# ddRAD sequencing-based genotyping for population structure analysis in cultivated tomato provides new insights into the genomic diversity of Mediterranean ‘da serbo’ type long shelf-life germplasm

**DOI:** 10.1038/s41438-020-00353-6

**Published:** 2020-09-01

**Authors:** Salvatore Esposito, Teodoro Cardi, Gabriele Campanelli, Sara Sestili, María José Díez, Salvador Soler, Jaime Prohens, Pasquale Tripodi

**Affiliations:** 1 CREA Research Centre for Vegetable and Ornamental Crops, Pontecagnano, (SA) Italy; 2 CREA Research Centre for Vegetable and Ornamental Crops, Monsampolo del Tronto (AP), Tronto, Italy; 3 Instituto de Conservación y Mejora de la Agrodiversidad Valenciana, Universitat Politècnica de València, 46022 Valencia, Spain

**Keywords:** Genetic markers, Genomics, Plant breeding

## Abstract

Double digest restriction-site associated sequencing (ddRAD-seq) is a flexible and cost-effective strategy for providing in-depth insights into the genetic architecture of germplasm collections. Using this methodology, we investigated the genomic diversity of a panel of 288 diverse tomato (*Solanum lycopersicum* L.) accessions enriched in ‘da serbo’ (called ‘de penjar’ in Spain) long shelf life (LSL) materials (152 accessions) mostly originating from Italy and Spain. The rest of the materials originate from different countries and include landraces for fresh consumption, elite cultivars, heirlooms, and breeding lines. Apart from their LSL trait, ‘da serbo’ landraces are of remarkable interest for their resilience. We identified 32,799 high-quality SNPs, which were used for model ancestry population structure and non-parametric hierarchical clustering. Six genetic subgroups were revealed, clearly separating most ‘da serbo’ landraces, but also the Spanish germplasm, suggesting a subdivision of the population based on type and geographical provenance. Linkage disequilibrium (LD) in the collection decayed very rapidly within <5 kb. We then investigated SNPs showing contrasted minor frequency allele (MAF) in ‘da serbo’ materials, resulting in the identification of high frequencies in this germplasm of several mutations in genes related to stress tolerance and fruit maturation such as *CTR1* and *JAR1*. Finally, a mini-core collection of 58 accessions encompassing most of the diversity was selected for further exploitation of key traits. Our findings suggest the presence of a genetic footprint of the ‘da serbo’ germplasm selected in the Mediterranean basin. Moreover, we provide novel insights on LSL ‘da serbo’ germplasm as a promising source of alleles for tolerance to stresses.

## Introduction

Tomato (*Solanum lycopersicum* L.) is the most economically important vegetable crop, being widely grown and appreciated for its organoleptic and nutritional properties. Its cultivation is spread on a global surface of 4.76 million hectares with a production of 182 million tons^1^. The domestication of tomato had its origins in the Andean region of South America, fitting a model in which a pre-domestication phase took place in the Andean region, with the domestication being completed in Mesoamerica^2,3^ and not so long before the discovery of America^4^. This process led to a series of morphological and physiological changes, the most important of which has been the increase of the size and weight of fruits and the variation of shape^3^. During the XVI century, tomato was introduced in Europe and mostly in Spain and Italy, where further domestication occurred, resulting in the development of a high diversity of local varieties. For this reason, the Mediterranean basin of Europe is recognized as a secondary center of diversification for tomato^5,6^.

The last century has been characterized by a boost of breeding activities aimed at introducing and selecting novel variation toward the development of various types of cultivars for different uses and destinations (e.g., fresh consumption or processing industry). The first tomato cultivars developed by growers through selection and propagation are known as heirloom varieties. Heirlooms are characterized by unique features related to their appearance (e.g. color, shape, etc.), flavor, and significance for specific local markets^6,7^. Similarly, landraces are traditional cultivars developed over time after adapting to natural and cultural environments. Although both have been selected and handed down by farmers, landraces are limited to specific ecogeographical habitats and therefore are adapted to certain pedoclimatic conditions, whereas heirlooms do not necessarily present this characteristic^7,8^. Furthermore, in several cases, heirlooms have been developed through cross-breeding activities toward the development of resilient cultivars adapted to low input farming^7^. Anyway, in crops with a high economic impact such as tomato, the release of numerous commercial true-breeding and hybrid varieties has become a common rule in the market. Generally, modern tomato varieties are uniform and highly productive, and are characterized by a reduced gene pool, having been bred for few traits of major interest for the growers and industry, with resulting drawbacks related to the lack of those quality traits positively perceived by consumers such as flavor^9^. The dynamic changes of the market, concerning the increasing attention to the environment and sustainability, have grown the interest in the ‘Zero’ concept in the food chain (e.g., zero kilometers, zero pesticides, etc.). At the same time, identity, ethical and cultural aspects attract the interest of customers who show greater appreciation for typical products and traditional cuisine. For this reason, in recent years there has been an increasing interest in local landraces, which, although not as productive as commercial cultivars and often vulnerable to major pests^8^, have a closer tie with the territory and local customs and are better adapted to the small-scale economies.

Among these, ‘da serbo’ (also called ‘de penjar’ or ‘de ramellet’ in Spain) tomato materials are typical long shelf life (LSL) tomatoes landraces that diversified in the Mediterranean region and which have been traditionally cultivated in Southern Italy and Spain. These LSL tomatoes are generally small, mostly round, although a wide range of shapes can be found, with thick cuticle, and different colors, from yellow to red with several shades. The extended shelf-life might have been the primary selection criteria^10^ and is associated with high resilience, manifested in the tolerance to different abiotic and biotic stresses, as well as to a high soluble solids content providing excellent organoleptic properties^11^. This syndrome of phenotypic characteristics of the LSL ‘da serbo’ landraces results in a clear differentiation from the standard fresh consumption tomatoes. The particular characteristics of the LSL ‘da serbo’ materials may be also used as a selective trait^10,12^, in fact, they have been selected in semiarid summer conditions with poor irrigation or rain-fed^10^. The greater attention to organic farming and sustainability in agriculture has sparked an increasing interest in the ‘da serbo’ LSL cultivars. The potential of these LSL materials and their diversity have not been extensively characterized and are not yet fully exploited in tomato breeding programs. Indeed, apart from the long shelf life and resilience characteristics, ‘da serbo’ LSL germplasm may represent a potential source of other traits of agricultural interest.

The diversity of local landraces and heirlooms of tomato has been investigated using PCR based fragment analysis or custom DNA arrays (Golden-Gate) involving from a few hundred markers^13–17^ to few thousand of markers (e.g., Illumina Infinium SolCAP)^18–20^. These studies revealed patterns of genetic diversity due to breeding activities, adaptation to the cultivation environment, market type and destination of use. Whole-genome sequencing and re-sequencing projects accelerated the development of high-throughput genotyping methods^21^. Double digest restriction-site associated DNA (ddRAD-seq) is a method based on the development of multiplexed libraries obtained through the enzymatic digestion of the whole genomic DNA followed by binding to specific adapters (reduced representation libraries). By reducing the portion of the sequenced genome, it generates a large set of SNP markers which can be used to infer very precisely the genetic diversity and population structure of large germplasm collections^22^. Moreover, the adoption of double restriction enables paired-end sequencing of identical loci across many samples ensuring higher accuracy in the mapping of reads with respect to genotyping by sequencing (GBS) or single RAD digestion (RAD-seq)^23^. The application of ddRAD-seq technology has been successful in many species including Solanaceae crops^24,25^; while, so far, its application to tomato for the investigation of germplasm collections has been limited. Indeed, to date, studies are addressed principally to ddRAD-seq in silico optimization protocols^23^.

A reference genome sequence of tomato has been available since 2012^26^. The first version, obtained by a combination of short-read 454 and a Sanger paired-end read sequencing, consisted of approximately 900 Mb and 34,727 protein-coding gene models. Versions SL2.50 and SL3.0 have been widely used as a reference for SNP calling in various tomato population studies. Very recently, a high quality *de novo* assembly of the tomato reference genome (SL4.0), obtained through a combination of long-read sequencing methodologies and optical mapping, has been released^27^. The new version reduced the number of the unknown bases and unplaced contigs by around 2,000 and 30-fold, respectively. Moreover, 4,794 novel genes have been reported compared to the previous version. The new genes information has been implemented by the sequencing of 13 diverse tomato accessions (Shatz and Lippman unpublished, https://solgenomics.net/projects/tomato13/). These novel resources allow a better exploitation of the genomic information on germplasm resources.

In the present study, we analyzed the genome-wide diversity of a collection of 288 tomato accessions using ddRAD-seq genotyping aiming to provide biological knowledge for biodiversity-based breeding. The represented tomato accessions included a wide range of landraces for fresh consumption and LSL ‘da serbo’ retrieved from the Mediterranean basin, as well as representative heirlooms and elite cultivars grown across the world. Population structure and diversity within subpopulations have been inferred by combining Bayesian and non-parametric approaches. Then we investigated in depth the genomic diversity of ‘da serbo’ materials, identifying those alleles subjected to selective sweeps and putatively responsible for the syndrome of phenotypic characteristics of LSL types. The collection studied, as well as a mini-core collection selected for genomic diversity, represents diversified panels to be exploited for future association mapping studies toward the determination of genomic regions responsible of traits underlying resilience and amenable to breeding.

## Materials and methods

### Plant material and DNA isolation

**Fig. 1:**
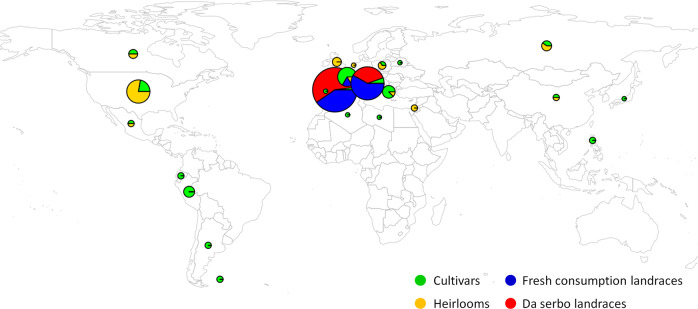
Geographical provenance of the 288 tomato accessions presented in this study. Circle colors define the type while their size is proportional to the number of samples

A diverse set of 288 cultivated tomato (*S. lycopersicum*) accessions were assembled from the germplasm collections from the Universitat Politècnica de València (UPV; Spain), the Research Center for Vegetable and Ornamental Crops (CREA; Italy), and the Tomato Genetics Resource Center (TGRC; USA). The germplasm panel included materials originating from 27 different countries of Europe, Asia, Africa and America (Fig. 1) and included 152 landraces (77 of the ‘da serbo’ typology and 75 of the standard typology for fresh consumption), mainly retrieved from Spain and Italy, 46 heirloom varieties, 76 elite cultivars and 14 breeding lines. Information about the accessions, including variety name and country of origin is in Supplementary Table 1. Genomic DNA was extracted from leaves of each accession using the DNeasy® Plant Mini Kit (QIAGEN, Germany). DNA quality parameters as well as concentration were measured by absorbance values at 260 nm and 280 nm, using a UV-Vis spectrophotometer (ND-1000; NanoDrop, Thermo Scientific, Wilmington, DE, USA).

### RADseq genotyping

In silico analysis on the tomato reference genome was used to select the best combination of two restriction enzymes and the best fragment size distribution to obtain the desired number of loci. Such analysis suggested that the *Mbo*I and *Sph*I enzyme pair is capable of producing a higher number of fragments, as already reported in other plant species^28^. Hence, this combination was used to generate fragments within the 288 tomato samples. Genomic DNA of each cultivar was double digested and incubated at 37 °C for 16–20 h. Fragmented DNA was then purified with AMPureXP beads (Agencourt) and ligated to barcoded adapters. Samples were pooled on multiplexing batches and bead purified. For each pool, targeted fragments distribution was collected on a BluePippin instrument (Sage Science Inc.). The gel eluted fraction was amplified with oligo primers that introduced TruSeq indexes and subsequently bead purified. The resulting libraries were checked with both Qubit 2.0 Fluorometer (Invitrogen, Carlsbad, CA) and Bioanalyzer DNA assay (Agilent Technologies, Santa Clara, CA). Libraries were finally processed with Illumina cBot for cluster generation on the flowcell, following the manufacturer’s instructions and sequenced with V4 chemistry paired end 125 bp mode on HiSeq2500 instrument (Illumina, San Diego, CA).

### SNP call, filtering and marker classification

Demultiplexing of raw Illumina reads was carried out using the process_radtags utility included in Stacks v2.0^29^. The quality distribution plot was generated using FASTQ and filtered data were then aligned to reference tomato genome (versions SL3.0 and SL.4.0) using BWA-MEM with default parameters^30^. Only uniquely aligned reads (i.e., reads with a mapping quality > 4) were selected for downstream analysis. Genomic sequences related to *S. lycopersicum* (versions SL3.0 and SL4.0) were obtained from Sol Genomics (https://solgenomics.net). Detection of all covered loci from the aligned reads was done using the gstacks program included in Stacks v2.0^29^, while filtering of detected loci was carried out using the included populations program with the option –*r* = 0.75, in order to retain only loci that are represented in at least 75% of the population. In addition, polymorphic homozygous markers were identified among the accessions using inhouse PERL scripts. Functional annotation of the identified variants associated genes was performed using SnpEff (v 3.1) (http://snpeff.sourceforge.net/). Gene diversity^31^ and Polymorphic Information Content (PIC)^32^, which can range from 0.0 to 0.5 for bi-allelic SNPs, were manually calculated.

### Population Structure and genetic diversity

Model-based ancestry estimation was obtained using the ADMIXTURE software^33^ with K ranging from 1 to 15. One thousand bootstrap replicates were run to estimate parameter standard errors. Ten-fold cross-validation (CV) procedure was performed and CV scores were used to determine the best K value. Non-parametric study of population structure was also carried out using AWclust^34^, with k-means clusters ranging from 1 to 40. The optimal number of clusters was inferred by the Bayesian Information Criterion statistics. Genetic relationships among individual accessions were finally assessed by the construction of a dendrogram based on the allele sharing distance and Ward’s clustering algorithm^34^. Polymorphic Information Content (PIC), Heterozygosity (H) and Gene Diversity were calculated using Power Marker software^35^. Multidimensional scaling (MDS) was performed using the ggscatter function implemented (https://rpkgs.datanovia.com/ggpubr/reference/ggscatter.html) in Rstudio^36^. Variants pruned for LD were used. The relationship between accessions was visualized by plotting scores for the first two PCs.

To identify genetically redundant accessions, a dissimilarity matrix was calculated using VCF2dis.1.0.py script. The dissimilarity index between two accessions was calculated from the entire VCF file as the proportion of unmatching alleles between two accessions: a null value (=0.00000) indicates redundant accessions.

### Development of a mini-core collection set and detection of private SNPs

To identify private SNPs in ‘da serbo’ landraces compared with the others, MAF values were computed at individual SNP loci in the two groups separately. Only SNPs that showed contrasting MAF values (> 0.4 in ‘da serbo’ and < 0.2 in the other cultivars) were further analyzed for their biological function using MapMan (http://mapman.gabipd.org/). Moreover, we implemented Core Hunter v. 2.0 to build a mini-core collection based on genotypic data^37^. The Core Hunter software was run in R with default parameters using the variants pruned for LD (http://www.r-project.org/), which allows choosing sampling intensity and the genetic measures to be used as selection criteria.

### Phenotypic analysis

Genotypes of the mini-core set were assessed for main qualitative and quantitative fruit traits. Plants were grown at the experimental organic field of the Research Centre for Vegetable and Ornamental Crops (CREA, Monsampolo Del Tronto located in the Tronto Valley of Marche Region). Traits analyzed included: (i) green shoulder (0, absent; 1, light green; 2, medium green; 3, dark green); (ii) external fruit color (1, yellow; 2, orange; 3, pink; 4, red; 5, purple; 6, brown; 7, green); (iii) fruit predominant shape (1, flat; 2, slightly flattened; 3, circular; 4, rectangular; 5, cylindrical; 6, elliptic; 7, heart shaped; 8, obovate; 9, ovate; 10, pear shaped; 11, pepper shaped); (iv) fruit firmness (1, very soft; 2, soft; 3, medium; 4, hard); (v) average fruit weight on a bulk of 18 fruits (in grams). Principal component analysis was performed using the computer package XLSTAT 2012.1.

## Results

### SNP discovery and functional annotation

The sequencing of RADs libraries in 288 tomato genotypes produced a total of 1,036,589,344 raw reads, corresponding to an average of 3.5 million read pairs per sample. We identified 277,335 SNPs in the previous annotated genome references (SL3.0), whereas 246,936 SNPs were identified using the new reference genome (SL4.0). The decreased total genome size of SL4.0 with respect to SL3.0 (from 828,076,956 bp to 782,520,033 bp), due to the shorter chromosomes length^27^, led to identifying ~26.000 SNP less compared with the previous annotation. Differences in length ranged from a minimum of 292,053 bp (Chr 7) to a maximum of 6,991,656 bp (Chr 3). The greatest difference in size was observed for the unlinked SNPs (chromosome 00) which were halved in the new annotation (~10.000 SNP less). Except for chromosomes 6 and 10, the SL4.0 allowed a higher percentage of SNPs distributed along each chromosome. The total number of SNPs and their distribution along chromosomes for SL3.0 and SL4.0 are reported in Supplementary Table 2, whereas gene localization, PIC and heterozygosity within SL4.0 are reported in Table 1. Most of the identified SNPs were localized in intergenic regions spanning the short and long arms of chromosomes (Supplementary Fig. 1). The average density resulted in one SNP every 3.48 kb across the twelve chromosomes, ranging from 1.75 kb (Chr 6) to 5.05 kb (Chr 11). Across the whole set, PIC values ranged from 0.003 to 0.375 (data not shown) with a mean of 0.044. The minimum average PIC values were encountered on chromosome 7 (0.020), while the maximum value was found in Chr 4 (0.083). On average, heterozygosity was 0.049 ranging from 0.003 to 0.500 (data not shown). The observed transitions/transversions ratio was 1.23, with a total of 2,081,361 transitions and 1,688,550 transversions events (data not shown). In particular, among transitions events, C > T and G > A were the most abundant, whereas G > T and C > A abounded within transversions events (Supplementary Fig. 2). Genotypes analyzed had in average a similar number of SNPs ranging from a minimum of 1,354 SNP up to 77,501.

**Table 1:** SNP number, distribution in intergenic and genic regions, average distance for each chromosome, polymorphic information content (PIC) and heterozygosity (H). Values based on SL4.0 annotation

Chromosome	SNP in intergenic regions	SNP in genic regions	Total	% genic SNP	Average SNP interdistance (Kb)^a^	Average PIC	Average H
Chr00	12,019	6551	18,570	35.277	0.519	0.053	0.063
Chr01	17,473	7028	24,501	28.685	3.709	0.031	0.035
Chr02	8774	5482	14,256	38.454	3.751	0.031	0.033
Chr03	12,285	4536	16,821	26.966	3.882	0.028	0.031
Chr04	13,600	4001	17,601	22.732	3.662	0.083	0.093
Chr05	13,198	3482	16,680	20.875	3.913	0.073	0.079
Chr06	20,176	6780	26,956	25.152	1.753	0.024	0.026
Chr07	16,436	3688	20,124	18.326	3.373	0.020	0.021
Chr08	13,313	4374	17,687	24.730	3.618	0.028	0.031
Chr09	17,496	4787	22,283	21.483	3.075	0.036	0.038
Chr10	11,572	4676	16,248	28.779	3.988	0.041	0.046
Chr11	9560	3836	13,396	28.635	4.059	0.064	0.071
Chr12	16,464	5349	21,813	24.522	3.057	0.055	0.064
Total	182,366	64,570	246,936				
Average				26.509	3.487	0.044	0.049

^a^Average values considered Chr 1–12

Three of them, harbored a higher number of variants (>50,000). In particular, the breeding line holding the greater number of SNPs (77,501) is LA2934 containing introgressions of the wild species *S. pimpinellifolium*. The two other Peruvian cultivars LA1313 and PI365927, held 54,166 and 68,247 SNPs, respectively. (Supplementary Fig. 3a). The observed average heterozygosity (in percentage) was also comparable within the germplasm collection with most genotypes showing values of ∼2% and only eight having values above 5% **(**Supplementary Fig. 3b). The SNP quality control procedure, based on successive filtering steps for call rate, minor allele frequency (MAF) (0.05) and percentage of missing values (20%) yielded a total of 32,779 high-quality SNPs in SL4.0. Raw sequence data were deposited at the National Center for Biotechnology Information Short Read Archive (http://www.ncbi.nlm.nih.gov/sra/) database under the accession number BioProject ID PRJNA638535.

Out of 246,936 SNPs, found using the latest tomato annotation (SL4.0), 10% (23.377 SNPs) were localized in 48% (2.297/4.794) of newly annotated genes. Most of these SNPs (21,991) have a possible modifier effect, followed by 721 with moderate impact, 608 with low impact and 57 with high impact. Most of these SNPs were localized in upstream gene regions (7,380), downstream gene regions (6,868), introns (5,917), 5′ UTR regions (1,006), 3′ UTR regions (820) (Supplementary Table 3). We further analyzed the genes showing sequence polymorphisms with high impact to get an interpretation of the effects of sequence changes. A total of 53 different genes were identified. Most of them had one SNP each (50 genes), whereas only three genes had two. Gene Ontology Enrichment Analysis (GOEA) was performed on this subset and no significant enrichment (FDR ≤ 0.05) was found (data not shown).

### Genomic diversity and population structure

**Fig. 2:**
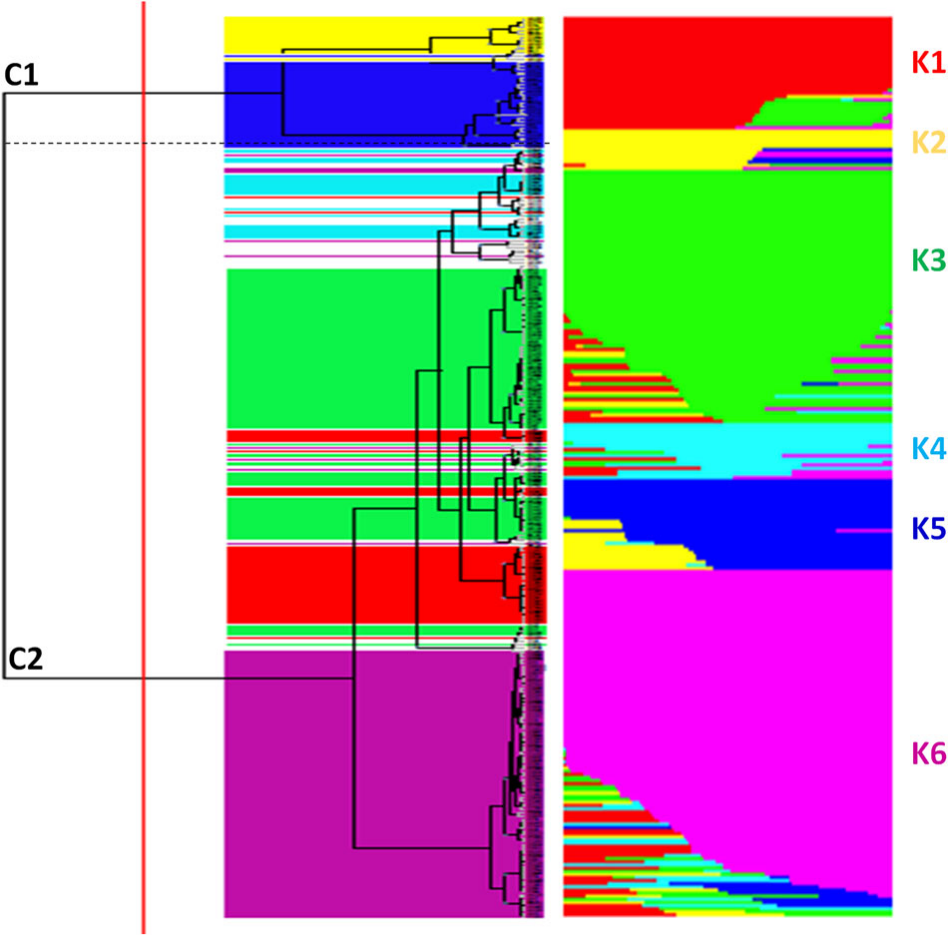
Estimate of genetic diversity in 288 tomato accessions using ddRAD. On the left, the dendrogram obtained through non-parametric hierarchical clustering. On the right, bar-plot describing the population admixture by the Bayesian approach. Each individual is represented by a thin horizontal line, which is partitioned into *K* colored segments whose length is proportional to the estimated membership coefficient (*q*). The population was divided into six (*K* = 6) groups according to the most informative *K* value (see Supplementary Fig. S4). Within the non-parametric model, the population was divided into two (*K* = 2) groups according to the most informative *K* value (see Supplementary Fig. S4). The colors indicate the accession membership to the groups identified with the Bayesian analysis

The admixture model was implemented to infer population structure. Using all SNP dataset, six different clusters were predicted (Supplementary Fig. 4a) and the population was accordingly divided in six sub-populations (K) (Fig. 2).

The first cluster (named “K1”), contained 10 cultivars from Peru, USA, Spain, France, Russia, Philippines and Falkland Islands, 23 Spanish landraces of which fourteen were ‘da serbo’, two heirlooms and one cultivar from Algeria, whereas 5 heirlooms, 4 landraces for fresh consumption (one from Italy and three from Spain), three breeding lines and one cultivar from Greece were placed in K2. The clusters K3 and K6 were the most abundant in terms of the number of genotypes, with 81 and 96, respectively. In particular, in cluster K3 most of the genotypes came from Italy (17 landraces of which 5 were ‘da serbo’), Spain (17 landraces of which 13 were ‘da serbo’) and France (8 cultivars and 3 landraces). Seventeen heirlooms, two breeding lines and 12 cultivars from various world regions were also included in K3. Similarly, 26 accessions from Italy (of which 10 were ‘da serbo’), 39 from Spain (of which 17 were ‘da serbo’), 11 heirloom, 14 cultivars, 5 unknown and one breeding line were included in K6. The cluster K4 grouped 11 ‘da serbo’ Spanish genotypes with 5 cultivars, one heirloom and a breeding line, whereas 7 landraces from Italy (2 ‘da serbo’ and 5 for fresh use), 10 cultivars, 7 heirlooms (five retrieved from the USA) and 4 breeding lines were included in K5. Nine accessions were classified as admixed, as they showed values for the highest cluster membership coefficient (*q*_*i*_) lower than 0.5.

**Fig. 3:**
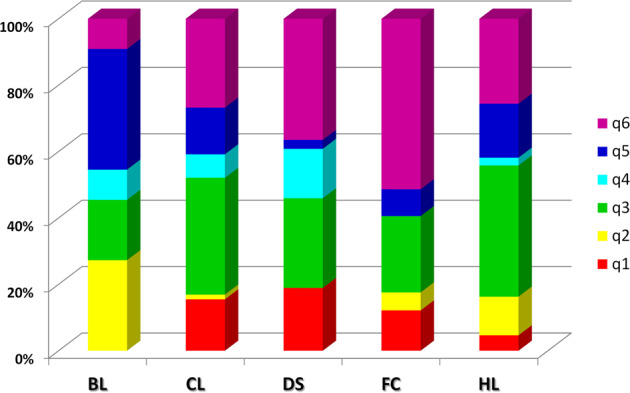
Stacked bar chart of the allele frequency based on *q* membership coefficient at *K* = 6. Groups of accessions characterized by a different biological status. *BL* breeding lines, *CL* cultivars, *DS* ‘da serbo’, *FC* fresh consumption, *HL* heirloom

The Fixation Index (*F*_*ST*_) values, measuring the population (K) differentiation based on SNP data, are reported in Table 2. The highest differentiation was found between K2 and other subpopulations, while the lowest divergence was found for clusters 3 and 6 (*F*_*ST*_ = 0.185). Considering the average *q*-value at *K* = 6 (Fig. 3), the analysis showed how breeding lines, ‘da serbo’ types and landraces for fresh consumption were not included in the clusters I, II and IV, respectively.

**Table 2:** *F*_*ST*_ divergences between populations inferred from a model-based ancestry estimation through the ADMIXTURE software^33^

	K1	K2	K3	K4	K5
**K2**	0.658				
**K3**	0.213	0.609			
**K4**	0.350	0.613	0.264		
**K5**	0.483	0.633	0.432	0.455	
**K6**	0.318	0.629	0.185	0.315	0.451

We complemented the investigation of genetic structure with the non-parametric method of k-means, which identifies clusters of individuals displaying the minimal within-group variance. Linkage disequilibrium-pruned SNPs were also used in this case, in order to avoid autocorrelation among linked loci. Based on the Bayesian Information Criterion, we found that genetic variation in the collection was efficiently summarized by gap statistic suggesting a minimum number of clusters (k) equal to 2 with 42 and 246 accessions, respectively (Supplementary Fig. 4b, Fig. 2).

Notably, a general overlap was observed between the clusters identified by the two-independent analysis. Indeed, sub-clusters at *K* = 6 were similar to the ones identified by Admixture (Fig. 2, Supplementary Table 4), except for the inclusion of accessions classified as admixed. The first branch of the dendrogram (C1, Supplementary Fig. 5; Supplementary Table 4) contained mostly heirloom and cultivars of diverse origin, and all the accessions belonging to K2 and K5 in the admixture analysis. The remaining K subpopulations and all admixed corresponded to the second branch (C2). Applying the Bayesian method, a better resolution of the genetic relationships of the accessions according to the geographical origin and the destination of use was observed. Indeed, different subgroups (Supplementary Table 4), were formed at the similarity level of 0.2 (Supplementary Fig. 5). In several cases, heirlooms tend to group mostly with cultivars and / or landraces for fresh use. Although not in a strict manner, it was possible to observe in many cases the grouping of accessions retrieved from the same region or a clustering according to biological status. As an example, French accessions tended to cluster together (e.g., C2_A1.2.1.1.1.2) as well as some heirlooms from North America and Central Europe (e.g., C2_B2.2) or the non-European germplasm (e.g., C1B2, C2_A1.1.1.1.2, C1_B1.1.3). Many American heirlooms tended to cluster with Italian landraces for fresh consumption. Homogeneous groups were made by Spanish ‘da serbo’ and/or other landraces (i.e., C2_A1.1.1.1.1.2, C2_A1.2.2.1, C2_A1.2.2.2). Overall, ‘da serbo’ landraces tended to cluster together while the remaining landraces grouped with commercial cultivars and heirlooms.

**Fig. 4:**
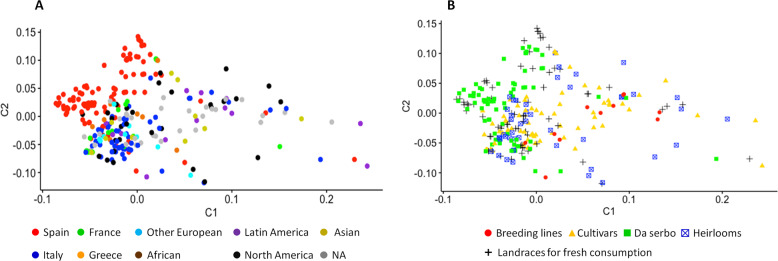
MDS clustering of 288 tomato accessions in the first two components based on 2454 SNP markers. **a** Diversity based on geographical provenance; the color of circles reflects the origin of accessions. Countries with more than 8 accessions were considered singularly, the remaining were grouped according to the main region of origin. **b** Diversity based on biological status. The color legends are in the figure

MDS was obtained after pruning the SNPs dataset for linkage disequilibrium. The resulting 2545 markers confirmed the best number of subpopulations (K) equal to 6. The score plot in the first two components (Fig. 4a) clearly separated Spanish accessions from the rest. The Italian germplasm tended to cluster closely, although more admixtures were found with germplasm from the other European countries and North American heirlooms. Interestingly, several close relationships were found between French and Greek accessions. As expected, Latin American cultivars and Asian ones clustered more distantly from European germplasm.

The second score plot based on the biological status of accessions (Fig. 4b), evidenced how the ‘da serbo’ accessions, in particular, those retrieved from Spain clustered together, while fresh consumption landraces made different tight close clusters. Heirlooms and cultivars were more dispersed than other categories, evidencing a higher variability. A close cluster including Italian landraces, heirlooms and cultivars was also observed.

**Fig. 5:**
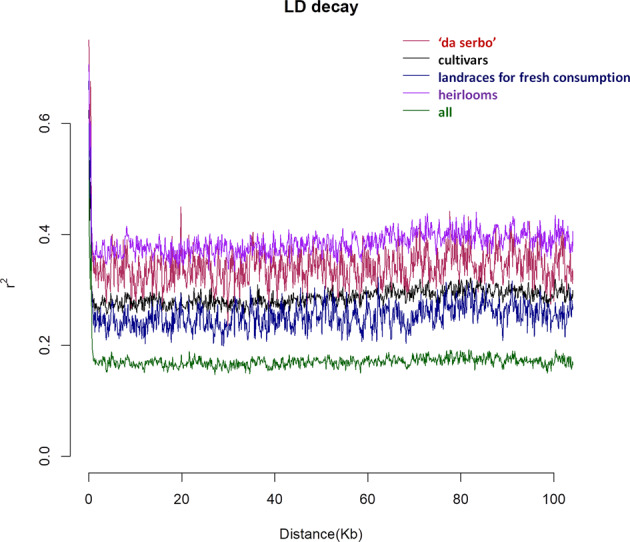
Linkage disequilibrium (LD) decay and comparison. The comparison between the different tomato populations. Different colors indicate the different group ‘da serbo’ landraces (red), cultivars (black), landraces for fresh consumption (blue), heirlooms (violet) and all together pooled (green). Distance within chromosomes is expressed in Kb

We calculated *r*^2^ between pairs of SNPs using PopLDdecay to estimate the linkage disequilibrium (LD) patterns in our collection and within the different major subgroups (‘da serbo’ landraces, fresh consumption landraces, cultivars and heirlooms). Linkage disequilibrium of the entire population decays very rapidly (within <5 kb). Landraces for fresh consumption and improved cultivars showed the lowest LD values (Fig. 5). By contrast, heirlooms showed the highest LD, with *r*^2^ near to 0.4. Overall, landraces showed a higher *r*^2^ range compared to the other typologies.

Non-redundant accessions were identified, although some of them were highly similar. For example, the Italian long shelf life accessions Piennolo 21 (DS8) and Pop25 (DS9) had a dissimilarity value of 0.00158, whereas the landraces Varrone (FC33) and Laura (FC34) showed a value of 0.00204 (Supplementary Table 5). The most different accessions were the BGV5592 (DS35) and LA2934 (BL1) with a dissimilarity index of 0.445.

In order to select the most diverse genotypes that represent most of the genetic variation with minimum redundancy, we selected a mini-core collection set from the original collection. To this end, a comparison of statistic and random sampling was implemented using the R software CoreHunter, which allowed us to sample 58 different genotypes that cover the highest genetic diversity of our collection (Supplementary Fig. 6). The mini-core collection set is highly heterogeneous comprising all accession biological statuses, in fact, a total of 23 cultivars, 12 heirlooms, 14 landraces (of which nine were of the ‘da serbo’ type) and 9 breeding lines were included. (Supplementary Table 6). Furthermore, all admixture clusters were represented. Indeed, out of 58 selected genotypes, 13 belonged to K5, whereas 13 were classified as admixed. As far as the remaining genotypes are concerned, the distribution was almost homogeneous in other clusters (K1 = 4; K2 = 8; K3 = 5; K4 = 6; K6 = 9).

**Fig. 6:**
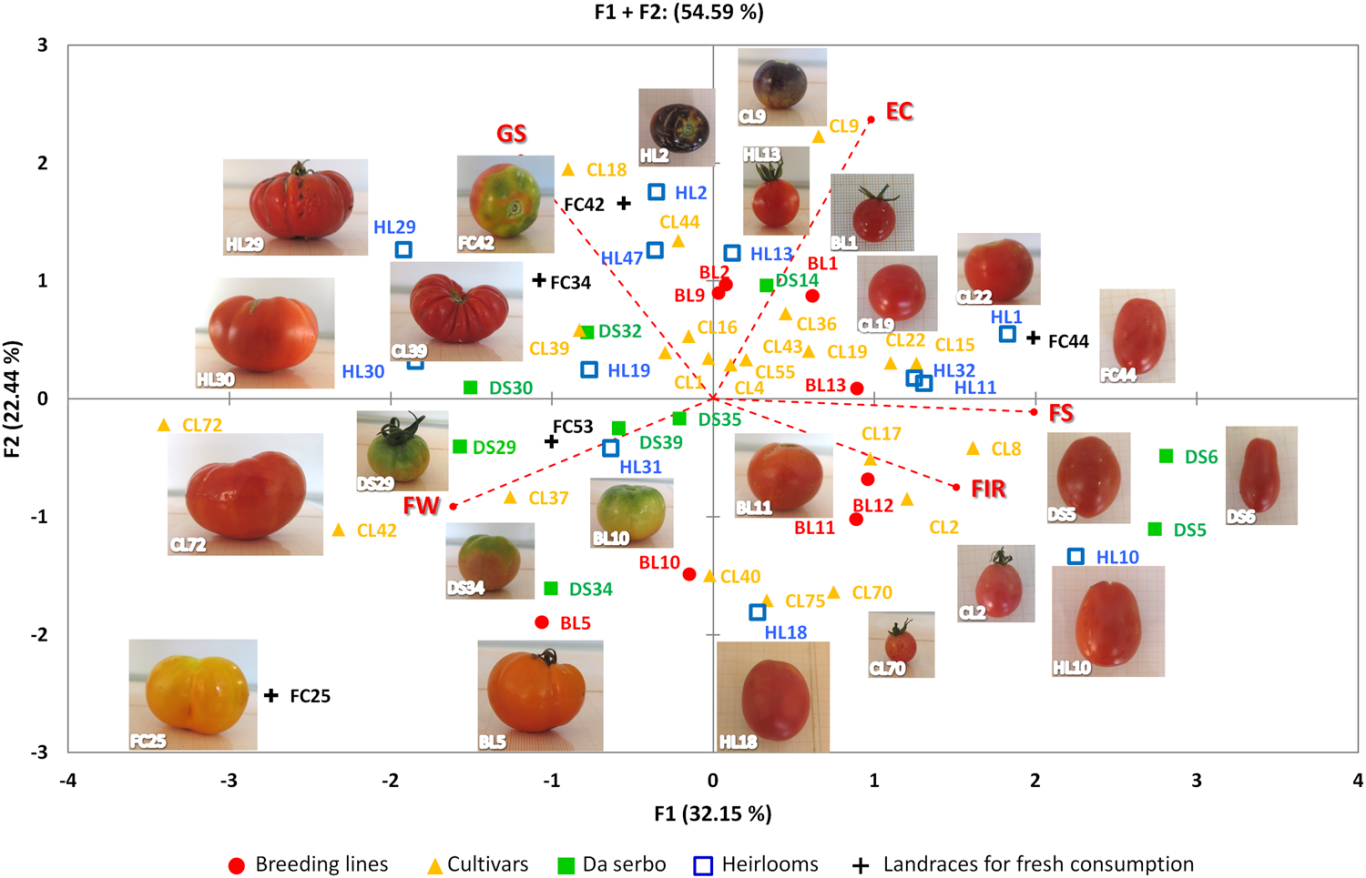
Loading plot of the first (F1) and second (F2) principal components showing the variation for main fruit traits in accessions of the mini-core set developed from ddRAD SNP data of 288 cultivated tomato genotypes. Code names are reported in Supplementary Table 1. *GS* green shoulder, *EC* external color, *FS* fruit shape, *FIR* fruit firmness, *FW* fruit weight

The group of selected accessions of the mini-core set was highly representative of the whole phenotypic variability of the collection. For the assessed fruit traits, the PCA in the first two components explained 54.59 % of the total variation (Fig. 6). The accessions were evenly distributed on the two axes of the biplot. The first component accounted for the 32.15% of the total variance and was positively correlated with external color, fruit shape, and fruit firmness and negatively correlated with the green shoulder and fruit weight. The second component accounted for the 22.44% of the variance being positively correlated only with green shoulder and external color.

### Genome-wide identification of private SNPs within ‘da serbo’ germplasm

In order to detect alleles differentiating ‘da serbo’ accessions from the rest of the germplasm collection, we computed the MAF values at individual SNP loci in both sets. A total of 53 SNPs showed contrasting values in the ‘da serbo’ group (MAF > 0.4) compared with other types (MAF < 0.2) (Supplementary Table 7). Five of them, of which one localized in the intergenic regions (chr 1) and four falling in genic regions (upstream region, downstream region, intron and 3’UTR) (chrs 4, 5 and 9) showed the most contrasting MAF values (MAF > 0.4 in ‘da serbo’ and <0.1 in other groups). Most of these SNPs were localized on chr 9 (24/53), followed by chr 10 (7/53). MapMan software was also used to further understand their biological role (Supplementary Table 8). However, since an input map file for the latest *S. lycopersicum* annotation is not available yet, we used Mercator online tool^38^ to associate the 34,071 tomato proteins to MapMan bins. Then, our list was mapped to MapMan bins for data visualization. Interestingly, on the long arm of chr 9, a high frequency for the SNP located in the Kinesin-14 annotated gene was found in ‘da serbo’ genotypes (Solyc09g075480.3, bin 20). This gene encodes for motor proteins acting in vesicular transport on cortical microtubules and regulates cell wall mechanics during cell elongation in plants^39^. As a cytoskeleton protein, it is likely involved in response to drought stress^40^. In addition, it has been recently shown that this class of kinesins enters into the nucleus in response to cold stress in rice, suggesting a role in osmotic stress conditions^41^.

The other genes with high MAF values in ‘da serbo’ genotypes were pre-pro-cysteine proteinase (Solyc04g080960.4, bin19), Cytochrome b561 (Solyc05g041910.3, bin 35) and Guanylate-binding protein (Solyc09g065810.3, bin 35). Cysteine proteinases are known to be involved in protein maturation, degradation, and protein re-built in response to different stress conditions^42^, whereas Cytochromes b561 (Cyt-b561) are important for plant growth, development, and damage prevention from light excess under drought condition^42^. By contrast, guanylate-binding proteins act as molecular switches to regulate diverse cellular processes, including bacterial and virus responses, by alternating between active (GTP-bound) and inactive conformation (GDP-bound)^43^. Investigating the biological role of the remaining genes harboring SNPs with contrasting MAF values, we noticed that the genes included in the bins 17 (Protein biosynthesis), 20 (Cytoskeleton organization) and 21 (Cell wall organization) also seem to have such function in biotic stress response^44^. One of them (Solyc10g083600.2, bin 17) is a component of ARPF2-ARRS1 assembly complex, which play an essential role in ribosome biogenesis and probably in ribosome stress response^45^, whereas Solyc11g013490.2 (bin 21) was identified as hexosyltransferase non-classical Arabinogalactan-protein, a family of hydroxyproline-rich glycoproteins ubiquitous in the plant kingdom and involved in several processes, from plant growth and development to reproduction^46^. Two other coding regions: Solyc10g083610.2 (bin 11) and Solyc05g050280.3-Solyc05g050290.3, bin (5) were implicated in phytohormone action. In particular, Solyc10g083610 encodes for a Raf-like serine/threonine (Ser/Thr) protein kinase annotated as *CTR1 (Constitutive triple response)*, whereas Solyc05g050280 transcribed for *Jasmonate Resistant 1* (*JAR1*). In tomato and Arabidopsis, *CTR1* has been reported to be involved in ethylene biosynthesis, being a negative regulator of signal transduction^47^. When the receptors perceive ethylene, *CTR1* kinase activity is shut off, thereby leading to responses. By contrast, *JAR1* encodes an enzyme that conjugates jasmonic acid (JA) to isoleucine, which was recently shown to be essential for pathogen defense^48^.

Single genes were involved in photosynthesis processes (bin 1), lipid metabolism (bin 5), chromatin organization (bin 12), DNA damage response (bin 14), RNA biosynthesis (bin 15), protein modification (bin 18), and solute transported (bin 24), whereas 15 genes did not show any functional description and were grouped in the group with not assigned function (bin 35) (Supplementary Table 8).

We also specifically looked for polymorphisms in *nac-nor* (*non ripening*) (Solyc10g006880), *rin* (*ripening inhibitor*) (Solyc05g012020) and *Cnr* (*Colorless non-ripening*) (Solyc02g077920), since they are related to a strong ripening impairment mainly related to ethylene insensitivity^49–51^. One polymorphism was found in the intergenic region of *nac-nor*, whereas two were detected in the *rin* region (one intergenic and one synonymous variant). All the variants detected passed the quality filtering. Regarding *Cnr*, four different variants, of which one passed quality filtering, were also identified. Only one was found in the upstream region and the minor allele was identified in 13 cultivars, 6 heirlooms, 4 unknown, 3 landraces and in one ‘da serbo’ genotype (data not shown).

## Discussion

### Genomic investigation of germplasm diversity

Tapping the diversity of crops is a prerequisite for efficient genetic improvement, conservation, and management of germplasm resources. The present work was aimed to study the genomic diversity of a diverse panel of cultivated tomato germplasm and to identify loci under selection and putatively responsible for the syndrome of differences related to LSL cultivars.

As a model species, tomato has been widely exploited for breeding, genomics and biology researches, being among the first vegetable crops having the whole genome sequence released. Within tomato populations genetics, efforts have been principally addressed toward the dissection of the genetic basis of advanced recombinant materials, elite germplasm, and wild species^52–55^. Approaches to investigate the diversity of traditional varieties including vintage and landraces have been performed with a low^13–17,56^, or medium marker-throughput^18–20^ in collections not larger than a hundred of accessions^13–15,17,19,20^ with few exceptions comprising a large proportion of improved cultivars^16,18,56^. These studies ascertained population stratification due to selection history, geographic adaptation and market destination^15–18,20^. Landraces were reported to have a lower allelic diversity compared to contemporary varieties, although the former may be richer in rare alleles^17^. In fact, modern varieties are developed through introgression breeding increasing the allelic assortment in established genetic backgrounds^18,54^. On the contrary, local varieties have been less exploited in terms of breeding and cultivation, having been replaced by high yielding varieties, particularly in intensive agricultural systems^57^. Therefore, the genetic composition of landraces is mainly due to selection driven by local practices in specific agricultural systems as well as environmental factors.

The gene pool could contribute to broadening and improving the genetic base of current modern varieties. A deeper exploration would give more insight to be exploited for tomato improvement. In this regard, we applied ddRAD sequencing protocol detecting 246,936 polymorphic sites and of 32,779 high-quality SNPs used to infer population structure and phylogenetic relationships using parametric and non-parametric computations. The abundance and whole-genome coverage provided by ddRAD based SNPs provide a high measure of genomic diversity in particular at the intra-specific level, where fewer polymorphisms occur compared to the inter-specific level^58^. The occurring heterozygosity across the whole collection is in agreement with previous evidence reporting values ranging from 1 to 4% in traditional varieties and higher values in the improved varieties^3^.

The results indicated a certain differentiation within the collection on the basis of biological status and provenance of accessions. Although a certain level of admixture was found between contemporary varieties, heirlooms, and landraces, the ‘da serbo’ LSL types were genetically more distinct. Moreover, a higher number of structured subgroups were found in landraces with respect to contemporary varieties. Indeed, although LSL and fresh consumption landraces represent 52% of the whole collection, fewer alleles were shared with the other typologies. Our observations are in agreement with previous researches that do not report subpopulation structures in varieties developed from advanced breeding programs such as cultivars and heirlooms, evidencing how the subpopulation structure is determined by natural selection and breeding history^15^. The number of K clusters identified can be considered consistent to define the population structure. Wang and colleagues^54^, and Bauchet and collaborators^56^, identified population structure within the typologies studied detecting 9 and 6 clusters in collections of 348 and 300 individuals, respectively. We also investigated the level of redundancy among accessions. The lowest values (<0.002 and <0.003) were found within landraces for fresh consumption as well within LSL, confirming how in situ conservation better maintains the genetic identity of tomato landraces due to reduced gene flow and genetic drift^59^. The MDS plot confirmed a low gene flow between LSL ‘da serbo’ materials and the other groups of accessions. Furthermore, allele exchange was observed between Italian landraces, heirlooms and elite cultivars, suggesting their greater exploitation in breeding programs. An LD decay within a few kilobases was observed in the 288 accessions studied. Robbins and colleagues evidenced an overall LD decay within a few cM with differences between the fresh market and processing market types^14^. The same trend was observed by Bauchet and collaborators reporting an LD fluctuating according to the genetic groups studied^54^. Both authors highlighted the breeding history and gene recombination as the main factors influencing the observed LD values. Moreover, the homozygosity level and mating system are two additional components affecting LD in crops. In fact, in outcrossing species, the LD can decay within hundreds of bp while for highly selfing species the LD may extend to 10 kb^60^. Moreover, accessions with different biological statuses can exhibit diverse LD values. As an example, in maize, the LD decay of landraces has been proven to be 100-fold less than in elite lines^61^. The same trend has been observed in heterogenous germplasm of barley^61^. The mini-core collection set of 58 tomato accessions was built with the objective to maximize genetic diversity with a reduced number of accessions. This mini-core collection represents the whole genetic and phenotypic diversity of the collection studied and could be useful to identify traits of agronomic and qualitative interest in tomato. Phenotyping is a major bottleneck to address in breeding due to costs and time required^62^. The establishment of a mini-core collection encompassing most of the diversity could be a valid solution to deeply explore the phenotypic diversity, overcoming the bottleneck of using very large collections to identify key traits for genetic improvement.

### Variants falling in gene regions characterizing ‘da serbo’ genotypes

Although the LSL are usually related to higher phenotypic performance and stress tolerance, the molecular mechanisms underlying the extended shelf-life and tolerance to drought cultivation conditions are not fully understood. To date, the LSL phenotype has been related to different mutations in genes such as *alc*, *nor* and *cnr*^12,63–66^. In particular, the *alc* mutation has been found in Spanish LSL ‘da serbo’ genotypes, which are commonly called ‘de penjar’ in Catalonia and Valencia and ‘de ramellet’ in the Balearic Islands^12,67^. In the ‘Alcobaça’ landrace the *alc* mutation was associated to a reduction of ethylene production (i.e., 25% less than ‘Rutgers’ cultivar^65^, and mutations *alc*-like have been suggested to play the same role in Italian ‘da serbo’ LSL landraces^10^. Similar results comparing the ethylene emission in ‘Ailsa Craig’ and four different ‘de penjar’ accessions, revealed that ethylene emission may be not only related to *alc* mutation^68^. A genetic screening within a collection of Italian ‘da serbo’ LSL landraces^20^, found high variation in ethylene-responsive genes, although no polymorphisms in the ethylene-dependent non-ripening (*nor*) transcription factor were found, endorsing the idea that genetic determinants of the LSL fruit phenotype may be different between Italian and Spanish landraces. In our study, we identified few polymorphisms in *nor-nac, rin* and *Cnr*, mainly localized in intergenic regions except for one variant detected in *Cnr*. Previously, Casals and collaborators^12^, identified a polymorphism in the second exon of the gene *nac-nor*, consisting of a replacement of thymine by adenine in the coding sequence responsible of the alcobaca (*alc*) mutation. In the same study^12^, the analysis of the *rin* region excluded its involvement as a cause of long shelf life in the Spanish ‘de penjar’ varietal types. Bota and colleagues^67^, also detected the presence of the *alc* mutation in a collection of Spanish ‘de ramellet” accessions from Majorca island. Our results suggest that, in addition to *alc*, other variants in *rin*, *nac-nor* and *Cnr* might be involved in the development of LSL ‘da serbo’ shelf life. We cannot exclude that our approach did not cover the region of *alc* previously studied^12^ due to the reduced genome representation occurring with ddRAD genotyping. Therefore, target sequencing may be used to verify the presence of such variants in this gene. Some SNPs were instead identified in ethylene signal transduction genes. Indeed, two SNPs were localized in intron regions of *Constitutive Triple Response 1* (*CTR1*), an important negative regulator of ethylene signaling. *CTR1* has a central position in the ethylene-response pathway, acting downstream of the ethylene receptors and upstream of *EIN2*, a membrane-integrated metal transporter-like protein^69^. In the presence of ethylene, the function of *CTR1* is inhibited and *EIN2* can translocate in the nucleus inducing the expression of ethylene-responsive genes^70^. By contrast, in absence of ethylene, *CTR1* can phosphorylate *EIN2*, which becomes inactive and does not induce its response.

In addition, it has been shown that *CTR1* is also involved in salt tolerance^71^. A mutant inactivating the allele-kinase in *CTR1* (ctr1-1), exhibited an increased salt tolerance during the germination and post-germination stages, suggesting a role of *CTR1* as negative regulator^71^. Therefore, the two variants identified in *CTR1* intron might play a dual role. On the one hand, they may act as enhancers, as already described in many plants such as Arabidopsis and rice^72,73^ In this scenario, *CTR1* might be expressed at a higher level even in the presence of ethylene, inhibiting the expression of related genes, which may lead to the extended shelf life of ‘da serbo’ accessions. On the other hand, intronic variants may induce aberrant splicing, resulting in defective protein products. In this case, *CTR1* might have an abnormal structure which can increase the tolerance to osmotic stresses such as salt and drought. However, since much remains unclear in respect to the signal transduction pathways from *CTR1* to *EIN2* in different plant species, other studies are ongoing to clarify the effect of the two variants identified.

Interesting variants were also identified in *JASMONATE RESISTANT 1* (*JAR1*), a coding-protein having a key role in the biosynthesis of jasmonate^48^. A variety of functions are associated with *JAR1* including resistance to biotic stress. Previous studies evidenced a correlation between jasmonate and reduced ethylene emission in ‘de Peniar’ accessions^68^, suggesting how the LSL fruit phenotype may be also related to non-ethylene mediated ripening regulation as in the non-ripening tomato mutants^10,74,75^.

In agreement with surveys of Tranchida-Lombardo and colleagues^20^, who identified a kinesin harboring a non-synonymous SNP in Italian ‘da serbo’, we found a SNP in the 3’-UTR region of Solyc09g075480.3. The underlying gene has high similarity to kinesin-14, which has a role in cell wall synthesis, acting in the deposition and orientation of cellulose microfibrils^76^. Other interesting variants were identified in cysteine proteinases (CP), cytochromes b561 (Cyt-b561) and guanylate-binding proteins (GBP). CP are mainly involved in protein rebuilt following external stimuli and in the protection against misfolded or damaged proteins^77^. The process could involve the rebuilding of cold and heat shock proteins, dehydration-induced proteins and pathogenesis-related proteins. Examples were reported in pea and *Arabidopsis* for which CP induced by water deficit are responsive to salt stress^78–80^. In tomato, CP are reported to have an active role in biotic and abiotic stress defense and suppression of autonecrosis^81,82^. Similarly, Cytochromes b561 are intrinsic membrane proteins involved in ascorbate regeneration known for its detoxification during aerobic metabolism and under stress conditions^83,84^. In watermelon, CYB561 has been reported to be induced by drought and high-light stress, suggesting a role for thermal dissipation of excess light energy, through functional interaction with apoplastic ASC oxidase^85^. In addition, through the modulation of levels of reactive oxygen species, ascorbate with its CYB561 activities are also implicated in the control of cell expansion, cell division, and programmed cell death^85^. Regarding GBP, it has been demonstrated how intravacuolar bacteria of infected cells stimulate the recruitment of antimicrobial guanylate binding proteins as part of a coordinated host defense program^86^.

Overall, these results support that mutations different from those previously known in genes such *nac-nor, rin* and *Cnr* may also be related to long shelf life and higher stress tolerance in Mediterranean ‘da serbo’ LSL tomato germplasm. Our results indicate putative genes under selection. The gained information suggests the possibility to further investigate specific genomic regions for the mining of novel alleles to be exploited for the improvement of long shelf life and resilient cultivars.

## Conclusions

Using ddRAD-seq for high throughput SNP discovery and the latest tomato genome version for annotation, we showed for the first-time new clues in a large fraction of Mediterranean ‘da serbo’ gene pool, providing furthermore, a list of 2297 newly and never studied target genes with different functions and harboring SNPs. The genomic analysis highlighted a geographical footprint distinguishing the landraces developed in the Mediterranean basin secondary center of diversity of tomato. We then investigated the level of variation underlying long shelf-life landraces, detecting SNPs falling in gene regions other than the previously known *alc*, *nac-nor* and *cnr*. Among the newly identified genes, those involved in biotic and abiotic stress conditions as well as in ethylene and jasmonate pathways may be worth being studied further. We finally provided breeders with a mini-core collection composed of the most diverse genotypes of our germplasm. Overall, the collections studied constitute a promising reservoir of genes for traits of interest, which could be further explored in genome-wide association studies and exploited in new tomato precision breeding programs.

## Supplementary information

Supplementary Table 1

Supplementary Table 2

Supplementary Table 3

Supplementary Table 4

Supplementary Table 5

Supplementary Table 6

Supplementary Table 7

Supplementary Table 8

Supplementary Figures
