# Supplementary material for: ddRAD sequencing-based genotyping for population structure analysis in cultivated tomato provides new insights into the genomic diversity of Mediterranean ‘da serbo’ type long shelf-life germplasm
Source: Hortic Res. 2020 Sep 1;7:134. doi: 10.1038/s41438-020-00353-6 (PMC7459340; doi:10.1038/s41438-020-00353-6)
Supplement: Supplementary file 1 — Supplementary Table 1 [file 41438_2020_353_MOESM1_ESM.pdf]

Supplementary Table 1: List of tomato accessions. Code: accession code; Name: name of the accession; Genebank code if available; Provenance: country of origin; Biological status: biological status of the accessions.

Code	Name	Genebank code	Provenance	Biological status/Tipology
BL1	LA2934 (*S. pimpinellifolum* introgression)		Perù	Breeding line
BL2	LA3668 (introgression of *S. lycopersicoides*)		*na*	Breeding line
BL3	LA1269(*S. pimpinellifolum* introgression)		Perù	Breeding line
BL4	NematodeResistent		*na*	Breeding line
BL5	LA0316 (Introgression from *S. habrochaites*)		*na*	Breeding line
BL6	LA1996 (Introgression from *S. chilense*)		*na*	Breeding line
BL7	LA2530		*na*	Breeding line
BL8	LA1033 (Introgression from *S. habrochaites*)		Perù	Breeding line
BL9	LA4425 (Introgression from *S. lycopersicoides*)		*na*	Breeding line
BL10	E 24		*na*	Breeding line
BL11	E 36		*na*	Breeding line
BL12	E 38		*na*	Breeding line
BL13	TY 172		*na*	Breeding line
BL14	7559		*na*	Breeding line
CL1	LA1051		*na*	Cultivars
CL2	LA3342 (Pto, Rio Grande background)		*na*	Cultivars
CL3	LA0059 (Improved line)		*na*	Cultivars
CL4	LA2460 (sft mutant tomato accession)		*na*	Cultivars
CL5	LA1051		*na*	Cultivars
CL6	LA0797 (cerasiforme type)		*na*	Cultivars
CL7	NC8776		*na*	Cultivars
CL8	Magliarosa		Italy	Cultivars
CL9	BlueP20		United States	Cultivars
CL10	Lyc2547		United States	Cultivars
CL11	NapoliVF		Italy	Cultivars
CL12	CLN2264F		*na*	Cultivars
CL13	nl91		*na*	Cultivars
CL14	LA1231		Ecuador	Cultivars
CL15	LA1459		Mexico	Cultivars
CL16	LA1463		*na*	Cultivars
CL17	Tomate Divisoria 2	BGV012514	Philippines	Cultivars
CL18	Tomate Nagcarlan	BGV012516	Philippines	Cultivars
CL19	Tomate Chang1	BGV012518	China	Cultivars
CL20	Tomate Heinz 1409	BGV012521	United States	Cultivars
CL21	Tomate Saladette	BGV012526	*na*	Cultivars
CL22	Tomate T95	BGV012527	Spain	Cultivars
CL23	Tomate Otawa 27	BGV012531	Canada	Cultivars
CL24	Tomate Campbell 35	BGV012535	United States	Cultivars
CL25	Edkawi 1987		Japan	Cultivars
CL26	v4654 (N 358)	BGV009538	Lybia	Cultivars
CL27	v3541	BGV009516	Algeria	Cultivars
CL28	v3475 (Cuyano)	BGV009511	Falkland Island	Cultivars
CL29	v3380 (Cuyano)	BGV009506	Falkland Island	Cultivars
CL30	v3291	BGV009503	*na*	Cultivars
CL31	v3092 (Marinadnyi 1)	BGV009498	Belarus	Cultivars
CL32	v2772	BGV009489	*na*	Cultivars
CL33	v2188	BGV009484	*na*	Cultivars
CL34	AFRL14		*na*	Cultivars
CL35	Catie10965	BGV008346	Costa rica	Cultivars
CL36	LA1313		Perù	Cultivars
CL37	LA2275		Perù	Cultivars

CL38	YL96		*na*	Cultivars
CL39	238-PI128639		Perù	Cultivars
CL40	c20249	BGV013135	*na*	Cultivars
CL41	PI365927		Perù	Cultivars
CL42	Uco Plata	BGV013683	Argentina	Cultivars
CL43	LA1421		Ecuador	Cultivars
CL44	LA2254		Perù	Cultivars
CL45	LA2208		Perù	Cultivars
CL46	Amelioree de Montlhery		France	Cultivars
CL47	Anabelle		France	Cultivars
CL48	NemausaeB		France	Cultivars
CL49	Areti		Greece	Cultivars
CL50	Makedonia		Greece	Cultivars
CL51	Santorini		Greece	Cultivars
CL52	Marmande		France	Cultivars
CL53	Outre Coeur de Boeuf		France	Cultivars
CL54	Abondance		France	Cultivars
CL55	Apedice		France	Cultivars
CL56	Chemin		France	Cultivars
CL57	Jaune Demi Lisse		France	Cultivars
CL58	Porphyre		France	Cultivars
CL59	Reinedes Precoces		France	Cultivars
CL60	Supermarmande		France	Cultivars
CL62	ARETI		Greece	Cultivars
CL62.1	Gran Saso Italian		Italy	Cultivars
CL63	Karampola		Greece	Cultivars
CL64	Laginati		Greece	Cultivars
CL65	Strogili Megali		Greece	Cultivars
CL66	Early Pak 7	BGV011426	United States	Cultivars
CL67	Hellfrucht Fruhstamm	BGV010796	Germany	Cultivars
CL68	Kumato	BGV013893	Spain	Cultivars
CL69	Lycoprea	BGV014202	Germany	Cultivars
CL70	Oregon T5-4	BGV011176	United States	Cultivars
CL71	Parteno	BGV011504	Poland	Cultivars
CL72	Platense	BGV010798	Argentina	Cultivars
CL73	Severianin	BGV014201	Russia	Cultivars
CL74	Sintra	BGV011512	Portugal	Cultivars
CL75	Sub Artic Plenty	BGV014206	Canada	Cultivars
CL76	Volgogradskii 595	BGV012603	Russia	Cultivars
DS1	Nocerino Nocera		Italy	Da serbo landraces
DS2	PiennoloPugli		Italy	Da serbo landraces
DS3	Regina		Italy	Da serbo landraces
DS4	Molese		Italy	Da serbo landraces
DS5	Molteno		Italy	Da serbo landraces
DS6	RedPear		Italy	Da serbo landraces
DS7	Nocerino		Italy	Da serbo landraces
DS8	Piennolo21		Italy	Da serbo landraces
DS9	Pop25		Italy	Da serbo landraces
DS10	SMGAR		Italy	Da serbo landraces
DS11	VesuvioPOP8		Italy	Da serbo landraces
DS12	VesuvioSMsmall		Italy	Da serbo landraces
DS12.1	99190		Italy	Da serbo landraces
DS13	SanMarzano113		Italy	Da serbo landraces
DS14	LSMPro		Italy	Da serbo landraces
DS15	CostFI		Italy	Da serbo landraces
DS16	SanMarzanoLPRO		Italy	Da serbo landraces

DS17	CampanoBal		Italy	Da serbo landraces
DS18	DelVesuvio25		Italy	Da serbo landraces
DS19	ReginaOstuni		Italy	Da serbo landraces
DS20	Tomate de penjar	BGV005528	Spain	Da serbo landraces
DS21	Tomate de penjar	BGV005514	Spain	Da serbo landraces
DS22	Tomate de penjar	BGV005494	Spain	Da serbo landraces
DS23	Tomate de penjar	BGV005502	Spain	Da serbo landraces
DS24	Tomate de penjar	BGV006058	Spain	Da serbo landraces
DS25	Tomate de penjar	BGV005449	Spain	Da serbo landraces
DS26	Tomate de penjar	BGV005569	Spain	Da serbo landraces
DS27	Tomate de penjar	BGV016059	Spain	Da serbo landraces
DS28	Tomate de penjar	BGV005490	Spain	Da serbo landraces
DS29	Tomate de penjar	BGV005413	Spain	Da serbo landraces
DS30	Tomate de penjar	BGV005509	Spain	Da serbo landraces
DS31	Tomate de penjar	BGV000569	Spain	Da serbo landraces
DS32	Tomate de penjar	BGV005485	Spain	Da serbo landraces
DS33	Tomate de penjar	BGV005413	Spain	Da serbo landraces
DS34	Tomate de penjar	BGV005454	Spain	Da serbo landraces
DS35	Tomate de penjar	BGV005592	Spain	Da serbo landraces
DS36	Tomate de penjar	BGV005511	Spain	Da serbo landraces
DS37	Tomate de penjar	BGV005482	Spain	Da serbo landraces
DS38	Tomate de penjar	BGV005660	Spain	Da serbo landraces
DS39	Tomate de penjar	BGV014803	Spain	Da serbo landraces
DS40	Tomate de penjar	BGV014806	Spain	Da serbo landraces
DS41	Tomate de penjar	BGV005661	Spain	Da serbo landraces
DS42	Tomate de penjar	BGV015050	Spain	Da serbo landraces
DS43	Tomate de penjar	BGV015051	Spain	Da serbo landraces
DS44	Tomate de penjar	BGV015056	Spain	Da serbo landraces
DS45	Tomate de penjar	BGV014802	Spain	Da serbo landraces
DS46	Tomate de penjar	BGV015356	Spain	Da serbo landraces
DS47	Tomate de penjar	BGV015880	Spain	Da serbo landraces
DS48	Tomate de penjar	BGV015881	Spain	Da serbo landraces
DS49	Tomate de penjar	BGV015944	Spain	Da serbo landraces
DS50	Tomate de penjar	BGV015945	Spain	Da serbo landraces
DS51	Tomate de penjar	BGV015968	Spain	Da serbo landraces
DS52	Tomate de penjar	BGV016251	Spain	Da serbo landraces
DS53	Tomate de penjar	BGV016200	Spain	Da serbo landraces
DS54	Tomate de penjar	BGV016202	Spain	Da serbo landraces
DS55	Tomate de penjar	BGV016203	Spain	Da serbo landraces
DS56	Tomate de penjar	BGV016207	Spain	Da serbo landraces
DS57	Tomate de penjar	BGV016208	Spain	Da serbo landraces
DS58	Tomate de penjar	BGV016210	Spain	Da serbo landraces
DS59	Tomate de penjar	BGV016211	Spain	Da serbo landraces
DS60	Tomate de penjar	BGV016068	Spain	Da serbo landraces
DS61	Tomate de penjar	BGV016073	Spain	Da serbo landraces
DS62	Tomate de penjar	BGV016075	Spain	Da serbo landraces
DS63	Tomate de penjar	BGV016078	Spain	Da serbo landraces
DS64	Tomate de penjar	BGV016084	Spain	Da serbo landraces
DS65	Tomate de penjar	BGV016309	Spain	Da serbo landraces
DS66	Tomate de penjar	BGV005460	Spain	Da serbo landraces
DS67	Tomate de penjar	BGV005505	Spain	Da serbo landraces
DS68	Tomate de penjar	BGV016375	Spain	Da serbo landraces
DS69	Tomate de penjar	BGV016374	Spain	Da serbo landraces
DS70	Tomate de penjar	BGV015056	Spain	Da serbo landraces
DS71	TRBA064		Spain	Da serbo landraces
DS72	TRBA084		Spain	Da serbo landraces

DS73	TRBA123		Spain	Da serbo landraces
DS74	LC391		Spain	Da serbo landraces
DS75	BGV005496		Spain	Da serbo landraces
DS76	SL Vistabella 1		Spain	Da serbo landraces
FC1	PomoLuca		Italy	Fresh consumption landraces
FC2	NeroPro		Italy	Fresh consumption landraces
FC3	RotondaLigure		Italy	Fresh consumption landraces
FC4	CherryPro		Italy	Fresh consumption landraces
FC5	NocCorbarino		Italy	Fresh consumption landraces
FC6	CostFiorentinoNovoli		Italy	Fresh consumption landraces
FC7	MarmandeFTR		France	Fresh consumption landraces
FC8	Genovese		Italy	Fresh consumption landraces
FC9	PomoMax		Italy	Fresh consumption landraces
FC10	Peraloc2		Italy	Fresh consumption landraces
FC11	VomanoAgrif		Italy	Fresh consumption landraces
FC12	Gaetani		Italy	Fresh consumption landraces
FC13	ValdasoBasili		Italy	Fresh consumption landraces
FC14	PeraAbr1		Italy	Fresh consumption landraces
FC15	delVesuvio		Italy	Fresh consumption landraces
FC16	PiennoloPro2		Italy	Fresh consumption landraces
FC17	PizzutelloPin		Italy	Fresh consumption landraces
FC18	Pisanello		Italy	Fresh consumption landraces
FC19	PomodoroStellaPisa		Italy	Fresh consumption landraces
FC20	BelmonteMax		Italy	Fresh consumption landraces
FC21	GiganteRR		Italy	Fresh consumption landraces
FC22	Ponderosa		Italy	Fresh consumption landraces
FC23	Rita2		Italy	Fresh consumption landraces
FC24	7536		Italy	Fresh consumption landraces
FC25	GialloPro		Italy	Fresh consumption landraces
FC26	PomNeroPro		Italy	Fresh consumption landraces
FC27	SanMarzano622		Italy	Fresh consumption landraces
FC28	Malareto		Italy	Fresh consumption landraces
FC29	PurRSUCilSardegna		Italy	Fresh consumption landraces
FC30	Gialloagrappoli		Italy	Fresh consumption landraces
FC31	MarmandeCap		France	Fresh consumption landraces
FC32	MarmandeCsmall		France	Fresh consumption landraces
FC33	Varrone		Italy	Fresh consumption landraces
FC34	Laura		Italy	Fresh consumption landraces
FC35	Fiaschettoamandorla		Italy	Fresh consumption landraces
FC36	EsquenaVerd	BGV002269	Spain	Fresh consumption landraces
FC37	Zaragozano	BGV010280	Spain	Fresh consumption landraces
FC38	Caqui	BGV000916	Spain	Fresh consumption landraces
FC39	Conserva de pruna	BGV005510	Spain	Fresh consumption landraces
FC40	Palosanto	BGV002205	Spain	Fresh consumption landraces
FC41	Poma	BGV002053	Spain	Fresh consumption landraces
FC42	Conserva de pera	BGV09775	Spain	Fresh consumption landraces
FC43	Flor de Baladre	BGV009787	Spain	Fresh consumption landraces
FC44	Huevo de Paloma	BGV009559	Spain	Fresh consumption landraces
FC45	Amarillo bombilla	BGV008369	Spain	Fresh consumption landraces
FC46	Beef redondo	BGV000094	Spain	Fresh consumption landraces
FC47	Elchero	BGV005432	Spain	Fresh consumption landraces
FC48	Corazon de Fitero	BGV012839	Spain	Fresh consumption landraces
FC49	Borracho de Aretxabaleta	BGV012843	Spain	Fresh consumption landraces
FC50	Moruno	BGV003859	Spain	Fresh consumption landraces
FC51	Tomate negro	BGV015830	Spain	Fresh consumption landraces
FC52	Rosa de Barbastro	BGV015832	Spain	Fresh consumption landraces

FC53	Cien flores	TRBA1730	Spain	Fresh consumption landraces
FC54	Cirereta Menorca	TRBA0500	Spain	Fresh consumption landraces
FC55	Rosadet	TRBA1590	Spain	Fresh consumption landraces
FC56	Tomate grande	TRBA1600	Spain	Fresh consumption landraces
FC57	Amarillo Ademuz	V-L-309/A	Spain	Fresh consumption landraces
FC58	Rosado Aretxabaleta		Spain	Fresh consumption landraces
FC59	Montserrat		Spain	Fresh consumption landraces
FC60	Pera Girona		Spain	Fresh consumption landraces
FC61	Del Pebre	SL-FONT-1	Spain	Fresh consumption landraces
FC62	De Borseta	SL-AMORADI-1	Spain	Fresh consumption landraces
FC63	ValencianaI	SL-CATARROJA-1	Spain	Fresh consumption landraces
FC64	ValencianaII	SL-CATARROJA-2	Spain	Fresh consumption landraces
FC65	ValencianaIII	SL-CATARROJA-3	Spain	Fresh consumption landraces
FC66	Muchamiel	SL-SANT-JOAN-2	Spain	Fresh consumption landraces
FC67	AG1	SL-AGULLENT-1	Spain	Fresh consumption landraces
FC68	AG3	SL-AGULLENT-3	Spain	Fresh consumption landraces
FC69	BOC1	SL-BOCAIRENT-1	Spain	Fresh consumption landraces
FC70	ONT1	SL-ONTINYENT-1	Spain	Fresh consumption landraces
FC71	Rosada d'Altea		Spain	Fresh consumption landraces
FC72	Morella Rosada		Spain	Fresh consumption landraces
FC73	Rosada d'Ademuz		Spain	Fresh consumption landraces
FC74	Belorado		Spain	Fresh consumption landraces
FC75	Cherry ecologico	SL-POBLA-2	Spain	Fresh consumption landraces
HL1	Artpinktiger		*na*	Heirloom
HL2	SunBlack		*na*	Heirloom
HL3	RutgersI		United States	Heirloom
HL4	AilsaCraigLA3174		Scotland	Heirloom
HL5	Blacktruffle		United States	Heirloom
HL6	Snowwhite		United States	Heirloom
HL7	LA3472 (Movione NIL of Ailsa Craig)		United States	Heirloom
HL8	Rehovot 13		Israel	Heirloom
HL9	LA2458 Ontario7710		Canada	Heirloom
HL10	LA4285 (CLN2264F salad type)		*na*	Heirloom
HL11	LA3151 (Mecline)		France	Heirloom
HL12	BlancheduQuebe		Canada	Heirloom
HL13	TomatodeGallina2		United States	Heirloom
HL14	VioletJasper		China	Heirloom
HL15	MichaelPollan (green zebra)		United States	Heirloom
HL16	Rosabe		*na*	Heirloom
HL17	Creamsausage		United States	Heirloom
HL18	Gajodemelon		United States	Heirloom
HL19	SiberienneRose		Russia	Heirloom
HL20	BlackCherry		United States	Heirloom
HL21	Geneva11		United States	Heirloom
HL22	BlackTula		Russia	Heirloom
HL23	MexicanRibbed		Mexico	Heirloom
HL24	ApricotRibbed		*na*	Heirloom
HL25	TangerineRibbed		United Kingdom	Heirloom
HL26	AilsaCraigLA3193		Scotland	Heirloom
HL27	LA2451_Manapal		United States	Heirloom
HL28	LA2009 (New yorker)		United States	Heirloom
HL29	LA2802 (Globonnie)		United States	Heirloom
HL30	LA3129		Israel	Heirloom
HL31	Babywine		United States	Heirloom
HL32	ABCPotatoleaf		United States	Heirloom
HL33	ROD271	BGV011178	Poland	Heirloom

HL34	Early Large Red USA	BGV004649	United States	Heirloom
HL35	Black Aisberg USA	BGV004675	Russia	Heirloom
HL36	Carbon USA	BGV004684	United States	Heirloom
HL38	v2620 (Rouge Glorie de Versalles)	BGV009488	France	Heirloom
HL39	v0085 (Princess of Wales)	BGV009480	United Kingdom	Heirloom
HL40	American Beauty USA	BGV004678	United States	Heirloom
HL41	Orange Strawberry USA	BGV004681	United States	Heirloom
HL42	Maria Agustina USA	BGV004630	United States	Heirloom
HL43	Lutescent USA	BGV004634	United States	Heirloom
HL44	Saint Pierre		France	Heirloom
HL45	Money Maker		Netherlands	Heirloom
HL46	Tomataki		Greece	Heirloom
HL47	Bubjekosoko	BGV012568	Poland	Heirloom

na = not available
