# Supplementary material for: ddRAD sequencing-based genotyping for population structure analysis in cultivated tomato provides new insights into the genomic diversity of Mediterranean ‘da serbo’ type long shelf-life germplasm
Source: Hortic Res. 2020 Sep 1;7:134. doi: 10.1038/s41438-020-00353-6 (PMC7459340; doi:10.1038/s41438-020-00353-6)
Supplement: Supplementary file 2 — Supplementary Table 2 [file 41438_2020_353_MOESM2_ESM.pdf]

Supplementary Table 2: SNP distribution along tomato chromosomes in SL3.0 and SL4.0. The chromosome length is also reported.

Chromosome	Length 3.0	Length 4.0	Number SNPs in SL3.0	Number SNPs in SL4.0	% SL3.0	% SL4.0
Chr00	20,852,292	9,643,250	28,909	18,570	10.571	7.520
Chr01	98,455,869	90,863,682	26,932	24,501	9.848	9.922
Chr02	55,977,580	53,473,368	15,660	14,256	5.726	5.773
Chr03	72,290,146	65,298,490	17,961	16,821	6.568	6.812
Chr04	66,557,038	64,459,972	18,502	17,601	6.765	7.128
Chr05	66,723,567	65,269,487	17,947	16,680	6.563	6.755
Chr06	49,794,276	47,258,699	29,961	26,956	10.956	10.916
Chr07	68,175,699	67,883,646	20,803	20,124	7.607	8.149
Chr08	65,987,440	63,995,357	17,742	17,687	6.488	7.163
Chr09	72,906,345	68,513,564	24,057	22,283	8.797	9.024
Chr10	65,633,393	64,792,705	18,409	16,248	6.731	6.580
Chr11	56,597,135	54,379,777	14,645	13,396	5.355	5.425
Chr12	68,126,176	66,688,036	21,948	21,813	8.026	8.833
Total	828,076,956	782,520,033	273.476	246,936		
