# Supplementary material for: ddRAD sequencing-based genotyping for population structure analysis in cultivated tomato provides new insights into the genomic diversity of Mediterranean ‘da serbo’ type long shelf-life germplasm
Source: Hortic Res. 2020 Sep 1;7:134. doi: 10.1038/s41438-020-00353-6 (PMC7459340; doi:10.1038/s41438-020-00353-6)
Supplement: Supplementary file 3 — Supplementary Table 3 [file 41438_2020_353_MOESM3_ESM.pdf]

Supplementary Table 3: Types and number of SNPs with potential effects on gene function within the germplasm collection.

Type of variants	Number of variants
**Impact MODIFIER**	
Upstream gene	7380
Downstream gene	6868
Intron	5917
5 prime UTR	1006
3 prime UTR	820
**Impact MODERATE**	
Missense	721
**Impact LOW**	
Synonymous	321
5 prime UTR premature start codon gain	226
Splice region	61
**Impact HIGH**	
Stop gained	37
Splice acceptor	8
Stop lost	5
Splice donor	4
Start lost	2
Stop retained	1
Total	23377
