# Supplementary material for: ddRAD sequencing-based genotyping for population structure analysis in cultivated tomato provides new insights into the genomic diversity of Mediterranean ‘da serbo’ type long shelf-life germplasm
Source: Hortic Res. 2020 Sep 1;7:134. doi: 10.1038/s41438-020-00353-6 (PMC7459340; doi:10.1038/s41438-020-00353-6)
Supplement: Supplementary file 4 — Supplementary Table 4 [file 41438_2020_353_MOESM4_ESM.pdf]

Supplementary Table 4: Distribution of the 288 tomato accessions according to Hierarchical cluster analysis and Bayesian model-based clustering. Using ADMIXTURE, the accessions were partitioned in six subgroups (K1 to K6), considering q≥0.50. The dendrogram showed the accessions divided in two main groups (C1 and C2) and relatively su-groups considering minimum variance cluster < 0.1.

Cod	Name	Type	Prov	Awclust	q1	q2	q3	q4	q5	q6	Admixture group	C1	C2
HL20	BlackCherry	Heirloom	United States/Russia	C1	0.00001	0.99995	0.00001	0.00001	0.00001	0.00001	K2		
FC75	Cherryecologico	Fresh consumption landraces	Spain	C1	0.00001	0.99995	0.00001	0.00001	0.00001	0.00001	K2		
FC4	CherryPro	Fresh consumption landraces	Italy	C1	0.00001	0.99995	0.00001	0.00001	0.00001	0.00001	K2	C1_A1.1	
HL47	Bubjekosoko	Heirloom	Poland	C1	0.00001	0.999944	0.000016	0.00001	0.00001	0.00001	K2		
BL9	LA4425	Breeding line	na	C1	0.000018	0.999938	0.000014	0.00001	0.00001	0.00001	K2		
HL32	ABCPotatoleaf	Heirloom	United States	C1	0.00001	0.99995	0.00001	0.00001	0.00001	0.00001	K2		
CL64	Laginati	Cultivars	Greece	C1	0.00001	0.586296	0.00001	0.00001	0.00001	0.413664	K2		
FC42	Conservadepera	Fresh consumption landraces	Spain	C1	0.00001	0.545686	0.00001	0.00001	0.00001	0.454274	K2	C1_A1.2	
HL33	ROD271	Heirloom	Poland	C1	0.00001	0.579302	0.00001	0.00001	0.00001	0.420658	K2		
BL13	TY	Breeding line	na	C1	0.067428	0.5555	0.377042	0.00001	0.00001	0.00001	K2		
BL1	LA2934	Breeding line	Perù	C1	0.00001	0.574199	0.000018	0.000013	0.42575	0.00001	K2		
FC44	HuevodePaloma	Fresh consumption landraces	Spain	C1	0.000012	0.559657	0.000017	0.00001	0.440295	0.00001	K2	C1_A2.1	
DS6	RedPear	Da serbo landraces	Italy	C1	0.00001	0.376201	0.00001	0.00001	0.623759	0.00001	K5		
HL10	LA4285 (CLN2264F salad type)	Heirloom	na	C1	0.000015	0.604827	0.00001	0.000011	0.395126	0.00001	K2		
CL75	SubArticPlenty	Cultivars	Canada	C1	0.00001	0.402636	0.00001	0.00001	0.597324	0.00001	K5		
BL14	7559	Breeding line	na	C1	0.00001	0.430392	0.00001	0.00001	0.569568	0.00001	K5		
CL70	OregonT5	Cultivars	United States	C1	0.00001	0.452658	0.00001	0.00001	0.547302	0.00001	K5	C1_A2.2	
FC24	7536	Fresh consumption landraces	Italy	C1	0.000011	0.393159	0.00001	0.000015	0.606795	0.00001	K5		
FC39	Conservadepruna	Fresh consumption landraces	Spain	C1	0.00001	0.400527	0.00001	0.00001	0.599433	0.00001	K5		
HL6	Snowwhite	Heirloom	United States	C1	0.00001	0.00001	0.00001	0.00001	0.99995	0.00001	K5		
FC19	PomodoroStellaPisa	Fresh consumption landraces	Italy	C1	0.00001	0.00001	0.00001	0.00001	0.99995	0.00001	K5		
DS5	Molteno	Da serbo landraces	Italy	C1	0.00001	0.00001	0.00001	0.00001	0.99995	0.00001	K5		
CL39	PI128639	Cultivars	Perù	C1	0.00001	0.00001	0.00001	0.00001	0.99995	0.00001	K5		
HL28	LA2009 (New yorker)	Heirloom	United States	C1	0.00001	0.00001	0.00001	0.00001	0.99995	0.00001	K5		
CL23	TomateOtawa	Cultivars	Canada	C1	0.00001	0.00001	0.017608	0.00001	0.982352	0.00001	K5	C1_B1.1.1	
BL7	LA2530	Breeding line	na	C1	0.00001	0.00001	0.00001	0.00001	0.99995	0.00001	K5		
BL3	LA1269	Breeding line	Perù	C1	0.00001	0.00001	0.00001	0.00001	0.99995	0.00001	K5		
HL18	Gajodemelon	Heirloom	United States	C1	0.00001	0.00001	0.00001	0.00001	0.99995	0.00001	K5		
HL17	Creamsausage	Heirloom	United States	C1	0.000017	0.00001	0.00001	0.00001	0.824159	0.175794	K5		
FC23	Rita2	Fresh consumption landraces	Italy	C1	0.00001	0.00001	0.00001	0.00001	0.99995	0.00001	K5		
FC18	Pisanello	Fresh consumption landraces	Italy	C1	0.00001	0.00001	0.00001	0.00001	0.99995	0.00001	K5		
CL12	CLN2264F	Cultivars	na	C1	0.00001	0.189551	0.00001	0.00001	0.810409	0.00001	K5	C1_B1.1.2	
HL11	LA3151 (Mecline)	Heirloom	France	C1	0.00001	0.00001	0.00001	0.00001	0.99995	0.00001	K5		
CL72	Platense	Cultivars	Argentina	C1	0.00001	0.00001	0.00001	0.00001	0.99995	0.00001	K5	C1_B1.1.3	
HL1	Artpinktiger	Heirloom	na	C1	0.00001	0.125885	0.00001	0.146946	0.727139	0.00001	K5		
FC9	PomoMax	Fresh consumption landraces	Italy	C1	0.00001	0.169068	0.00001	0.00001	0.830892	0.00001	K5		
BL4	NematodeResistent	Breeding line	na	C1	0.000011	0.175238	0.00001	0.00001	0.824721	0.00001	K5		
CL19	TomateChang	Cultivars	China	C1	0.00001	0.187483	0.00001	0.00001	0.812477	0.00001	K5	C1_B1.2	
HL21	Geneva11	Heirloom	United States	C1	0.00001	0.181449	0.00001	0.00001	0.818511	0.00001	K5		
CL9	BlueP20	Cultivars	United States	C1	0.000014	0.174217	0.00001	0.00001	0.82574	0.00001	K5		
CL41	PI365927	Cultivars	Perù	C1	0.00001	0.372341	0.00001	0.060089	0.567541	0.00001	K5	C1B2	
CL36	LA1313	Cultivars	Perù	C1	0.00001	0.362027	0.00001	0.00001	0.637933	0.00001	K5		
DS43	BGV15051	Da serbo landraces	Spain	C2	0.165484	0.00001	0.00001	0.526288	0.00001	0.308198	K4		
DS34	BGV5454	Da serbo landraces	Spain	C2	0.165353	0.00001	0.00001	0.527377	0.00001	0.30724	K4		
DS28	BGV5490	Da serbo landraces	Spain	C2	0.00001	0.00001	0.00001	0.479636	0.00001	0.520324	K6		
CL74	Sintra	Cultivars	Portugal	C2	0.00001	0.00001	0.00001	0.515351	0.00001	0.484609	K4		C2_A1.1.1.1.1.1
DS49	BGV15944	Da serbo landraces	Spain	C2	0.00001	0.00001	0.00001	0.661888	0.00001	0.338072	K4		
DS40	BGV14806	Da serbo landraces	Spain	C2	0.283052	0.00001	0.00001	0.400841	0.00001	0.316077	Admixted		
FC34	Laura	Fresh consumption landraces	Italy	C2	0.00001	0.00001	0.00001	0.377693	0.00001	0.622267	K6		
FC33	Varrone	Fresh consumption landraces	Italy	C2	0.00001	0.00001	0.00001	0.374705	0.00001	0.625255	K6		
DS76	SL	Da serbo landraces	Spain	C2	0.00001	0.00001	0.00001	0.99995	0.00001	0.00001	K4		
DS59	BGV16211	Da serbo landraces	Spain	C2	0.00001	0.00001	0.00001	0.99995	0.00001	0.00001	K4		
DS47	BGV15880	Da serbo landraces	Spain	C2	0.00001	0.00001	0.029568	0.970392	0.00001	0.00001	K4		
DS74	BGVLC391	Da serbo landraces	Spain	C2	0.12689	0.00001	0.00001	0.87307	0.00001	0.00001	K4		C2_A1.1.1.1.1.2
DS63	BGV16078	Da serbo landraces	Spain	C2	0.00001	0.00001	0.00001	0.99995	0.00001	0.00001	K4		
DS39	BGV14803	Da serbo landraces	Spain	C2	0.00001	0.00001	0.00001	0.99995	0.00001	0.00001	K4		
DS51	BGV15968	Da serbo landraces	Spain	C2	0.000011	0.00001	0.304935	0.695023	0.00001	0.00001	K4		
CL44	LA2254	Cultivars	Perù	C2	0.519942	0.00001	0.00001	0.00001	0.00001	0.480018	K1		
CL16	LA1463	Cultivars	na	C2	0.30546	0.00001	0.00001	0.111799	0.00001	0.582711	K6		
HL13	TomatodeGallina2	Heirloom	United States	C2	0.143937	0.00001	0.066964	0.415471	0.056003	0.317615	Admixted		
HL19	SiberienneRose	Heirloom	Russia	C2	0.286844	0.00001	0.310662	0.402465	0.00001	0.00001	Admixted		C2_A1.1.1.1.2
CL15	LA1459	Cultivars	Mexico	C2	0.341377	0.00001	0.00001	0.658583	0.00001	0.00001	K4		
CL18	TomateNagcarlan	Cultivars	Philippines	C2	0.639347	0.00001	0.203396	0.036482	0.00001	0.120755	K1		
CL17	TomateDivisoria	Cultivars	Philippines	C2	0.414428	0.00001	0.00001	0.585532	0.00001	0.00001	K4		
BL5	LA0316	Breeding line	na	C2	0.237463	0.00001	0.00001	0.411194	0.351313	0.00001	Admixted		
CL14	LA1231	Cultivars	Ecuador	C2	0.129183	0.00001	0.00001	0.185995	0.373527	0.311276	Admixted		
CL69	Lycoprea	Cultivars	Germany	C2	0.00001	0.00001	0.125092	0.593037	0.00001	0.281841	K4		
CL55	Apedice	Cultivars	France	C2	0.00001	0.00001	0.341929	0.62967	0.00001	0.028372	K4		
HL29	La2802 (Globonnie)	Heirloom	United States	C2	0.00001	0.00001	0.00001	0.99995	0.00001	0.00001	K4		
BL8	LA1033	Breeding line	Perù	C2	0.00001	0.00001	0.00001	0.922027	0.00001	0.077933	K4		C2_A1.1.1.2
DS61	BGV16073	Da serbo landraces	Spain	C2	0.000011	0.00001	0.00001	0.999949	0.00001	0.00001	K4		
CL4	LA2460	Cultivars	na	C2	0.00001	0.204478	0.00001	0.022288	0.00001	0.773205	K6		
DS14	LSMPro	Da serbo landraces	Italy	C2	0.00001	0.477063	0.130886	0.00001	0.00001	0.392021	Admixted		
DS30	BGV5509	Da serbo landraces	Spain	C2	0.197542	0.268432	0.00001	0.00001	0.386914	0.147092	Admixted		
BL2	LA3668	Breeding line	na	C2	0.00001	0.00001	0.190165	0.382399	0.427406	0.00001	Admixted		
FC53	Cienflores	Fresh consumption landraces	Spain	C2	0.00001	0.00001	0.00001	0.422842	0.349808	0.22732	Admixted		
CL43	LA1421	Cultivars	Ecuador	C2	0.00001	0.00001	0.00001	0.00001	0.326042	0.673918	K6		C2_A1.1.2.2

CL3	LA0059	Cultivars	na	C2	0.223217	0.00001	0.273643	0.00001	0.424085	0.079035	Admixted	
HL30	LA3129 (Rehovot 13)	Heirloom	Israel	C2	0.000019	0.243103	0.361566	0.00001	0.395293	0.00001	Admixted	
CL1	LA1051	Cultivars	na	C2	0.00001	0.00001	0.424995	0.082875	0.00001	0.4921	Admixted	
HL2	SunBlack	Heirloom	na	C2	0.00001	0.00001	0.992865	0.00001	0.00001	0.007095	K3	
CL5	LA1051	Cultivars	na	C2	0.00001	0.00001	0.961499	0.029043	0.00001	0.009428	K3	
HL5	Blacktruffle	Heirloom	United States	C2	0.007815	0.00001	0.992145	0.00001	0.00001	0.00001	K3	
HL35	BlackAisbergUSA	Heirloom	United States	C2	0.00001	0.00001	0.99995	0.00001	0.00001	0.00001	K3	
FC17	PizzutelloPin	Fresh consumption landraces	Italy	C2	0.00001	0.00001	0.99995	0.00001	0.00001	0.00001	K3	
FC15	delVesuvio	Fresh consumption landraces	Italy	C2	0.00001	0.00001	0.99995	0.00001	0.00001	0.00001	K3	C2_A1.2.1.1.1.1
FC5	NocCorbarino	Fresh consumption landraces	Italy	C2	0.00001	0.00001	0.99995	0.00001	0.00001	0.00001	K3	
DS4	Molese	Da serbo landraces	Italy	C2	0.00001	0.00001	0.99995	0.00001	0.00001	0.00001	K3	
CL67	HellfruchtFruhstamm	Cultivars	Germany	C2	0.00001	0.00001	0.99995	0.00001	0.00001	0.00001	K3	
CL56	Chemin	Cultivars	France	C2	0.00001	0.00001	0.99995	0.00001	0.00001	0.00001	K3	
HL26	AilsaCraigLA3193	Heirloom	Scotland	C2	0.00001	0.00001	0.99995	0.00001	0.00001	0.00001	K3	
HL4	AilsaCraigLA3174	Heirloom	Scotland	C2	0.00001	0.00001	0.99995	0.00001	0.00001	0.00001	K3	
HL38	v2620 (Rouge Glorie de Versalles)	Heirloom	France	C2	0.00001	0.00001	0.99995	0.00001	0.00001	0.00001	K3	
FC7	MarmandeFTR	Fresh consumption landraces	France	C2	0.00001	0.00001	0.99995	0.00001	0.00001	0.00001	K3	
CL25	Edkawi1987	Cultivars	Japan	C2	0.00001	0.00001	0.99995	0.00001	0.00001	0.00001	K3	
CL59	ReinedesPrecoces	Cultivars	France	C2	0.00001	0.00001	0.99995	0.00001	0.00001	0.00001	K3	
CL54	Abondance	Cultivars	France	C2	0.00001	0.00001	0.99995	0.00001	0.00001	0.00001	K3	
CL60	Supermarmande	Cultivars	France	C2	0.00001	0.00001	0.99995	0.00001	0.00001	0.00001	K3	
CL57	JauneDemiLisse	Cultivars	France	C2	0.00001	0.00001	0.99995	0.00001	0.00001	0.00001	K3	C2_A1.2.1.1.1.2
FC32	MarmandeCsmall	Fresh consumption landraces	France	C2	0.00001	0.00001	0.99995	0.00001	0.00001	0.00001	K3	
CL29	v3380 (Cuyano)	Cultivars	Falkland Island	C2	0.00001	0.00001	0.99995	0.00001	0.00001	0.00001	K3	
DS15	CostFI	Da serbo landraces	Italy	C2	0.00001	0.00001	0.99995	0.00001	0.00001	0.00001	K3	
CL13	nl91	Cultivars	na	C2	0.00001	0.00001	0.99995	0.00001	0.00001	0.00001	K3	
HL8	LA3129 (Rehovot 13)	Heirloom	Israel	C2	0.00001	0.00001	0.99995	0.00001	0.00001	0.00001	K3	
FC31	MarmandeCap	Fresh consumption landraces	France	C2	0.00001	0.00001	0.99995	0.00001	0.00001	0.00001	K3	
DS72	TRBA084	Da serbo landraces	Spain	C2	0.010282	0.00001	0.989678	0.00001	0.00001	0.00001	K3	
DS71	TRBA064	Da serbo landraces	Spain	C2	0.00001	0.00001	0.99995	0.00001	0.00001	0.00001	K3	
CL22	TomateT	Cultivars	Spain	C2	0.00001	0.00001	0.999947	0.000013	0.00001	0.00001	K3	
FC37	Zaragozano	Fresh consumption landraces	Spain	C2	0.101116	0.00001	0.898844	0.00001	0.00001	0.00001	K3	
DS56	BGV16207	Da serbo landraces	Spain	C2	0.311383	0.00001	0.688577	0.00001	0.00001	0.00001	K3	
FC74	Belorado	Fresh consumption landraces	Spain	C2	0.345869	0.00001	0.654091	0.00001	0.00001	0.00001	K3	
HL25	TangerineRibbed	Heirloom	United Kingdom	C2	0.193703	0.00001	0.806257	0.00001	0.00001	0.00001	K3	
HL24	ApricotRibbed	Heirloom	na	C2	0.200192	0.00001	0.799768	0.00001	0.00001	0.00001	K3	
DS75	BGV5496	Da serbo landraces	Spain	C2	0.453872	0.00001	0.546088	0.00001	0.00001	0.00001	K3	
DS69	BGV16374	Da serbo landraces	Spain	C2	0.335675	0.00001	0.664285	0.00001	0.00001	0.00001	K3	
HL22	BlackTula	Heirloom	Russia	C2	0.00001	0.00001	0.99995	0.00001	0.00001	0.00001	K3	
FC2	NeroPro	Fresh consumption landraces	Italy	C2	0.00001	0.00001	0.99995	0.00001	0.00001	0.00001	K3	
CL35	Catie10965	Cultivars	Costa rica	C2	0.00001	0.00001	0.99995	0.00001	0.00001	0.00001	K3	
FC13	ValdasoBasili	Fresh consumption landraces	Italy	C2	0.180876	0.00001	0.819084	0.00001	0.00001	0.00001	K3	C2_A1.2.1.1.2.1
DS27	BGV16059	Da serbo landraces	Spain	C2	0.031873	0.025854	0.843262	0.00001	0.00001	0.098991	K3	
DS73	TRBA123	Da serbo landraces	Spain	C2	0.00001	0.00001	0.638783	0.361177	0.00001	0.00001	K3	
FC26	PomNeroPro	Fresh consumption landraces	Italy	C2	0.00001	0.00001	0.99995	0.00001	0.00001	0.00001	K3	
HL40	AmericanBeautyUSA	Heirloom	United States	C2	0.017049	0.00001	0.982911	0.00001	0.00001	0.00001	K3	
HL23	MexicanRibbed	Heirloom	Mexico	C2	0.04659	0.00001	0.95337	0.00001	0.00001	0.00001	K3	
HL36	CarbonUSA	Heirloom	United States	C2	0.00001	0.00001	0.99995	0.00001	0.00001	0.00001	K3	
DS12	VesuvioSMsmall	Da serbo landraces	Italy	C2	0.00001	0.00001	0.976548	0.00001	0.00001	0.023412	K3	
DS2	PiennoloPugli	Da serbo landraces	Italy	C2	0.000013	0.00001	0.999947	0.00001	0.00001	0.00001	K3	
DS17	CampanoBal	Da serbo landraces	Italy	C2	0.115978	0.00001	0.883982	0.00001	0.00001	0.00001	K3	
FC28	Malareto	Fresh consumption landraces	Italy	C2	0.00001	0.00001	0.99995	0.00001	0.00001	0.00001	K3	
FC27	SanMarzano622	Fresh consumption landraces	Italy	C2	0.000019	0.00001	0.999941	0.00001	0.00001	0.00001	K3	
DS7	Nocerino	Da serbo landraces	Italy	C2	0.00001	0.00001	0.99995	0.00001	0.00001	0.00001	K3	
DS1	Nocerino	Da serbo landraces	Italy	C2	0.00001	0.00001	0.99995	0.00001	0.00001	0.00001	K3	
DS48	BGV15881	Da serbo landraces	Spain	C2	0.604447	0.00001	0.395513	0.00001	0.00001	0.00001	K1	
DS42	BGV15050	Da serbo landraces	Spain	C2	0.607153	0.00001	0.392807	0.00001	0.00001	0.00001	K1	C2_A1.2.1.1.2.2
DS46	BGV15356	Da serbo landraces	Spain	C2	0.596685	0.00001	0.403275	0.00001	0.00001	0.00001	K1	
DS58	BGV16210	Da serbo landraces	Spain	C2	0.593502	0.00001	0.406458	0.00001	0.00001	0.00001	K1	
BL12	E	Breeding line	na	C2	0.015616	0.031839	0.67106	0.00001	0.117155	0.164319	K3	
CL42	UcoPlata	Cultivars	Argentina	C2	0.00001	0.00001	0.00001	0.31918	0.00001	0.68078	K6	
DS53	BGV16200	Da serbo landraces	Spain	C2	0.550749	0.00001	0.390255	0.00001	0.00001	0.058966	K1	
FC72	MorellaRosada	Fresh consumption landraces	Spain	C2	0.366856	0.00001	0.633104	0.00001	0.00001	0.00001	K3	
FC12	Gaetani	Fresh consumption landraces	Italy	C2	0.483807	0.00001	0.516153	0.00001	0.00001	0.00001	K3	C2_A1.2.1.2.1.1
FC62	DeBorseta	Fresh consumption landraces	Spain	C2	0.122009	0.000014	0.302358	0.00001	0.00001	0.575599	K6	
CL11	NapoliVF	Cultivars	Italy	C2	0.00001	0.00001	0.612676	0.00001	0.00001	0.387284	K3	
CL26	v4654 (N 358)	Cultivars	Lybia	C2	0.00001	0.00001	0.99995	0.00001	0.00001	0.00001	K3	
DS33	BGV5413	Da serbo landraces	Spain	C2	0.287878	0.001111	0.19601	0.00001	0.00001	0.514981	K6	
DS25	BGV5449	Da serbo landraces	Spain	C2	0.323819	0.00001	0.676141	0.00001	0.00001	0.00001	K3	
DS24	BGV16058	Da serbo landraces	Spain	C2	0.114354	0.00001	0.59404	0.291575	0.00001	0.00001	K3	
DS60	BGV16068	Da serbo landraces	Spain	C2	0.00001	0.00001	0.814927	0.00001	0.00001	0.185033	K3	
DS52	BGV16251	Da serbo landraces	Spain	C2	0.00001	0.00001	0.813692	0.00001	0.00001	0.186268	K3	
DS57	BGV16208	Da serbo landraces	Spain	C2	0.020383	0.00001	0.795332	0.00001	0.00001	0.184255	K3	
CL76	Volgogradskii595	Cultivars	Russia	C2	0.574047	0.00001	0.418813	0.00001	0.00001	0.00711	K1	
HL42	MariaAgustinaUSA	Heirloom	United States	C2	0.581315	0.000012	0.404266	0.00001	0.00001	0.014386	K1	
DS29	BGV5413	Da serbo landraces	Spain	C2	0.574061	0.00001	0.397167	0.00001	0.00001	0.028742	K1	
CL66	EarlyPak7	Cultivars	United States	C2	0.00001	0.00001	0.99995	0.00001	0.00001	0.00001	K3	
HL34	EarlyLargeRedUSA	Heirloom	United States	C2	0.00001	0.00001	0.99995	0.00001	0.00001	0.00001	K3	C2_A1.2.1.2.1.2
CL65	StrogiliMegali	Cultivars	Greece	C2	0.00001	0.00001	0.99995	0.00001	0.00001	0.00001	K3	
CL47	Anabelle	Cultivars	France	C2	0.00001	0.00001	0.99995	0.00001	0.00001	0.00001	K3	
CL62	ARETI	Cultivars	Greece	C2	0.00001	0.00001	0.99995	0.00001	0.00001	0.00001	K3	
CL49	Areti	Cultivars	Greece	C2	0.00001	0.00001	0.99995	0.00001	0.00001	0.00001	K3	
CL53	OutreCoeurdeBoeuf	Cultivars	France	C2	0.00001	0.00001	0.99995	0.00001	0.00001	0.00001	K3	
FC67	AG1	Fresh consumption landraces	Spain	C2	0.014374	0.00001	0.928584	0.00001	0.00001	0.057012	K3	
HL41	OrangeStrawberryUSA	Heirloom	United States	C2	0.057261	0.00001	0.942699	0.00001	0.00001	0.00001	K3	
CL30	v3291	Cultivars	na	C2	0.00001	0.00001	0.99995	0.00001	0.00001	0.00001	K3	

HL16	Rosabe	Heirloom	na	C2	0.00001	0.00001	0.99995	0.00001	0.00001	0.00001	K3	
CL58	Porphyre	Cultivars	France	C2	0.00001	0.00001	0.99995	0.00001	0.00001	0.00001	K3	
BL10	E	Breeding line	na	C2	0.00001	0.184059	0.815901	0.00001	0.00001	0.00001	K3	
FC25	GialloPro	Fresh consumption landraces	Italy	C2	0.00001	0.181602	0.818358	0.00001	0.00001	0.00001	K3	C2_A1.2.1.2.2
FC47	Elchero	Fresh consumption landraces	Spain	C2	0.00001	0.166486	0.00001	0.00001	0.00001	0.833474	K6	
BL11	E	Breeding line	na	C2	0.066206	0.228711	0.475758	0.00001	0.179887	0.049429	Admixted	
CL45	LA2208	Cultivars	Perù	C2	0.985396	0.00001	0.014563	0.00001	0.00001	0.00001	K1	
CL37	LA2275	Cultivars	Perù	C2	0.99995	0.00001	0.00001	0.00001	0.00001	0.00001	K1	
DS41	BGV5661	Da serbo landraces	Spain	C2	0.99995	0.00001	0.00001	0.00001	0.00001	0.00001	K1	
DS38	BGV5660	Da serbo landraces	Spain	C2	0.99995	0.00001	0.00001	0.00001	0.00001	0.00001	K1	
CL68	Kumato	Cultivars	Spain	C2	0.99995	0.00001	0.00001	0.00001	0.00001	0.00001	K1	
DS62	BGV16075	Da serbo landraces	Spain	C2	0.99995	0.00001	0.00001	0.00001	0.00001	0.00001	K1	C2_A1.2.2.1
DS54	BGV16202	Da serbo landraces	Spain	C2	0.99995	0.00001	0.00001	0.00001	0.00001	0.00001	K1	
DS44	BGV15056	Da serbo landraces	Spain	C2	0.99995	0.00001	0.00001	0.00001	0.00001	0.00001	K1	
DS50	BGV15945	Da serbo landraces	Spain	C2	0.99995	0.00001	0.00001	0.00001	0.00001	0.00001	K1	
DS70	BGV15056	Da serbo landraces	Spain	C2	0.99995	0.00001	0.00001	0.00001	0.00001	0.00001	K1	
DS65	BGV16309	Da serbo landraces	Spain	C2	0.99995	0.00001	0.00001	0.00001	0.00001	0.00001	K1	
FC60	PeraGirona	Fresh consumption landraces	Spain	C2	0.99995	0.00001	0.00001	0.00001	0.00001	0.00001	K1	
FC36	EsquenaVerd	Fresh consumption landraces	Spain	C2	0.99995	0.00001	0.00001	0.00001	0.00001	0.00001	K1	
FC71	RosadadAltea	Fresh consumption landraces	Spain	C2	0.99995	0.00001	0.00001	0.00001	0.00001	0.00001	K1	
FC46	Beefredondo	Fresh consumption landraces	Spain	C2	0.99995	0.00001	0.00001	0.00001	0.00001	0.00001	K1	
FC38	Caqui	Fresh consumption landraces	Spain	C2	0.99995	0.00001	0.00001	0.00001	0.00001	0.00001	K1	
FC41	Poma	Fresh consumption landraces	Spain	C2	0.99995	0.00001	0.00001	0.00001	0.00001	0.00001	K1	
FC40	Palosanto	Fresh consumption landraces	Spain	C2	0.99995	0.00001	0.00001	0.00001	0.00001	0.00001	K1	C2_A1.2.2.2
FC50	Moruno	Fresh consumption landraces	Spain	C2	0.99995	0.00001	0.00001	0.00001	0.00001	0.00001	K1	
FC48	CorazondeFitero	Fresh consumption landraces	Spain	C2	0.99995	0.00001	0.00001	0.00001	0.00001	0.00001	K1	
CL46	AmelioreedeMontlhery	Cultivars	France	C2	0.99995	0.00001	0.00001	0.00001	0.00001	0.00001	K1	
HL44	SaintPierre	Heirloom	France	C2	0.99995	0.00001	0.00001	0.00001	0.00001	0.00001	K1	
CL50	Makedonia	Cultivars	Greece	C2	0.99995	0.00001	0.00001	0.00001	0.00001	0.00001	K1	
CL28	v3475 (Cuyano)	Cultivars	Falkland Island	C2	0.99995	0.00001	0.00001	0.00001	0.00001	0.00001	K1	
CL27	v3541	Cultivars	Algeria	C2	0.970639	0.00001	0.00001	0.00001	0.00001	0.029321	K1	
CL6	LA0797	Cultivars	na	C2	0.00001	0.367755	0.632205	0.00001	0.00001	0.00001	K3	
CL7	NC8776	Cultivars	na	C2	0.000011	0.375542	0.624417	0.00001	0.00001	0.00001	K3	
CL24	TomateCampbell35	Cultivars	United States	C2	0.00001	0.351347	0.648613	0.00001	0.00001	0.00001	K3	
HL27	LA2451Manapal	Heirloom	United States	C2	0.077953	0.348455	0.573562	0.00001	0.00001	0.00001	K3	
CL20	TomateHeinz1409	Cultivars	United States	C2	0.675124	0.324836	0.00001	0.00001	0.00001	0.00001	K1	C2_A2
CL8	Magliarosa	Cultivars	Italy	C2	0.344058	0.322683	0.333229	0.00001	0.00001	0.00001	Admixted	
DS21	BGV5514	Da serbo landraces	Spain	C2	0.029148	0.267063	0.703759	0.00001	0.00001	0.00001	K3	
DS35	BGV5592	Da serbo landraces	Spain	C2	0.00001	0.367077	0.00001	0.00001	0.00001	0.632883	K6	
CL40	c20249	Cultivars	na	C2	0.304679	0.246475	0.448816	0.00001	0.00001	0.00001	Admixted	
DS66	BGV5460	Da serbo landraces	Spain	C2	0.269461	0.00001	0.00001	0.000018	0.00001	0.730491	K6	
DS55	BGV16203	Da serbo landraces	Spain	C2	0.118309	0.00001	0.00001	0.00001	0.00001	0.881651	K6	
FC10	Peraloc2	Fresh consumption landraces	Italy	C2	0.00001	0.00001	0.021221	0.00001	0.00001	0.978739	K6	
FC1	PomoLuca	Fresh consumption landraces	Italy	C2	0.00001	0.00001	0.00001	0.00001	0.00001	0.99995	K6	
DS12.1	99190	Da serbo landraces	Italy	C2	0.00001	0.00001	0.00001	0.00001	0.00001	0.99995	K6	
CL62.1	Gran Saso Italian	Cultivars	Italy	C2	0.00001	0.00001	0.00001	0.00001	0.00001	0.99995	K6	
DS13	SanMarzano113	Da serbo landraces	Italy	C2	0.00001	0.00001	0.00001	0.00001	0.00001	0.99995	K6	
FC3	RotondaLigure	Fresh consumption landraces	Italy	C2	0.00001	0.00001	0.00001	0.00001	0.00001	0.99995	K6	
FC14	PeraAbr1	Fresh consumption landraces	Italy	C2	0.00001	0.00001	0.00001	0.00001	0.00001	0.99995	K6	
FC11	VomanoAgrif	Fresh consumption landraces	Italy	C2	0.00001	0.00001	0.00001	0.00001	0.00001	0.99995	K6	
FC22	Ponderosa	Fresh consumption landraces	Italy	C2	0.00001	0.00001	0.00001	0.00001	0.00001	0.99995	K6	
DS36	BGV5511	Da serbo landraces	Spain	C2	0.00001	0.00001	0.00001	0.00001	0.00001	0.99995	K6	
FC68	AG3	Fresh consumption landraces	Spain	C2	0.00001	0.00001	0.00001	0.00001	0.00001	0.99995	K6	
FC29	PurRSUCilSardegna	Fresh consumption landraces	Italy	C2	0.00001	0.00001	0.000019	0.00001	0.00001	0.999941	K6	
FC21	GiganteRR	Fresh consumption landraces	Italy	C2	0.00001	0.00001	0.00001	0.00001	0.00001	0.99995	K6	
DS68	BGV16375	Da serbo landraces	Spain	C2	0.00002	0.00001	0.00001	0.00001	0.00001	0.99994	K6	
FC20	BelmonteMax	Fresh consumption landraces	Italy	C2	0.00001	0.00001	0.00001	0.00001	0.00001	0.99995	K6	
DS31	BGV5569	Da serbo landraces	Spain	C2	0.007763	0.00001	0.00001	0.00001	0.00001	0.992197	K6	
DS26	BGV5569	Da serbo landraces	Spain	C2	0.00001	0.00001	0.00001	0.00001	0.00001	0.99995	K6	
CL73	Severianin (partenocarpic)	Cultivars	Russia	C2	0.00001	0.00001	0.00001	0.000011	0.00001	0.999949	K6	
CL71	Parteno (partenocarpic)	Cultivars	Poland	C2	0.00001	0.00001	0.00001	0.00001	0.00001	0.99995	K6	
CL2	LA3342	Cultivars	na	C2	0.00001	0.01214	0.00001	0.00001	0.00001	0.98782	K6	
CL34	AFRL14	Cultivars	na	C2	0.00001	0.00001	0.00001	0.000011	0.00001	0.999949	K6	
FC35	Fiaschettoamandorla	Fresh consumption landraces	Italy	C2	0.00001	0.000016	0.00001	0.00001	0.00001	0.999944	K6	
CL21	TomateSaladette	Cultivars	na	C2	0.00001	0.00001	0.00001	0.00001	0.00001	0.99995	K6	
DS67	BGV5505	Da serbo landraces	Spain	C2	0.000013	0.00001	0.00001	0.00001	0.00001	0.999947	K6	
DS64	BGV16084	Da serbo landraces	Spain	C2	0.00001	0.00001	0.00001	0.00001	0.00001	0.99995	K6	C2_B1.1
FC65	ValencianaIII	Fresh consumption landraces	Spain	C2	0.00001	0.00001	0.00001	0.000012	0.00001	0.999947	K6	
FC63	ValencianaI	Fresh consumption landraces	Spain	C2	0.00001	0.00001	0.00001	0.00001	0.00001	0.99995	K6	
FC64	ValencianaII	Fresh consumption landraces	Spain	C2	0.00001	0.00001	0.00001	0.00001	0.00001	0.99995	K6	
FC56	Tomategrande	Fresh consumption landraces	Spain	C2	0.006465	0.00001	0.00001	0.00001	0.00001	0.993495	K6	
FC52	RosadeBarbastro	Fresh consumption landraces	Spain	C2	0.00001	0.00001	0.00001	0.00001	0.00001	0.99995	K6	
CL10	Lyc2547	Cultivars	United States	C2	0.00001	0.00001	0.00001	0.00001	0.00001	0.99995	K6	
DS37	BGV5482	Da serbo landraces	Spain	C2	0.00001	0.00001	0.00001	0.00001	0.00001	0.99995	K6	
DS20	BGV5528	Da serbo landraces	Spain	C2	0.00001	0.00001	0.00001	0.005332	0.00001	0.994628	K6	
FC51	Tomatenegro	Fresh consumption landraces	Spain	C2	0.00001	0.00001	0.00001	0.00001	0.00001	0.99995	K6	
FC66	Muchamiel	Fresh consumption landraces	Spain	C2	0.00001	0.00001	0.00001	0.00001	0.00001	0.99995	K6	
FC59	Montserrat	Fresh consumption landraces	Spain	C2	0.000011	0.00001	0.000011	0.00001	0.00001	0.999947	K6	
DS16	SanMarzanoLPRO	Da serbo landraces	Italy	C2	0.00001	0.00001	0.006032	0.00001	0.00001	0.993928	K6	
DS10	SMGAR	Da serbo landraces	Italy	C2	0.00001	0.00001	0.000677	0.00001	0.00001	0.999283	K6	
FC58	RosadAretxabaleta	Fresh consumption landraces	Spain	C2	0.00001	0.00001	0.00001	0.00001	0.00001	0.99995	K6	
FC49	BorrachodAretxabaleta	Fresh consumption landraces	Spain	C2	0.00001	0.00001	0.00001	0.00001	0.00001	0.99995	K6	
FC61	DepPebre	Fresh consumption landraces	Spain	C2	0.00001	0.00001	0.00001	0.00001	0.00001	0.99995	K6	
CL52	Marmande	Cultivars	France	C2	0.00001	0.00001	0.00001	0.00001	0.00001	0.99995	K6	
CL48	NemausaeB	Cultivars	France	C2	0.00001	0.00001	0.00001	0.00001	0.00001	0.99995	K6	
CL63	Karampola	Cultivars	Greece	C2	0.057241	0.00001	0.00001	0.00001	0.00001	0.942719	K6	

DS22	BGV5494	Da serbo landraces	Spain	C2	0.120814	0.00001	0.00001	0.117476	0.00001	0.76168	K6		
FC45	Amarillobombilla	Fresh consumption landraces	Spain	C2	0.00001	0.00001	0.08255	0.00001	0.00001	0.91741	K6		
FC70	ONT1	Fresh consumption landraces	Spain	C2	0.00001	0.00001	0.151558	0.00001	0.00001	0.848402	K6		
FC69	BOC1	Fresh consumption landraces	Spain	C2	0.00001	0.00001	0.15979	0.00001	0.00001	0.84017	K6		
HL31	Babywine	Heirloom	United States	C2	0.00001	0.00001	0.170738	0.000011	0.00001	0.829221	K6		
DS32	BGV5485	Da serbo landraces	Spain	C2	0.00001	0.00001	0.00001	0.245787	0.00001	0.754173	K6		
DS23	BGV5502	Da serbo landraces	Spain	C2	0.246565	0.00001	0.00001	0.212976	0.00001	0.540428	K6		
FC8	Genovese	Fresh consumption landraces	Italy	C2	0.356533	0.00001	0.00001	0.00001	0.00001	0.643427	K6		
HL43	LutescentUSA	Heirloom	United States	C2	0.332617	0.000012	0.00001	0.00001	0.00001	0.667341	K6		
FC43	FlordeBaladre	Fresh consumption landraces	Spain	C2	0.382799	0.00001	0.00001	0.00001	0.00001	0.617161	K6		
HL9	LA2458Ontario7710	Heirloom	Canada	C2	0.290591	0.000018	0.00001	0.00001	0.00001	0.709361	K6		C2_B1.2
FC73	RosadadAdemuz	Fresh consumption landraces	Spain	C2	0.38488	0.00001	0.00001	0.00001	0.00001	0.61508	K6		
FC57	AmarilloAdemuz	Fresh consumption landraces	Spain	C2	0.387773	0.00001	0.00001	0.00001	0.00001	0.612187	K6		
CL33	v2188	Cultivars	na	C2	0.277458	0.00001	0.00001	0.00001	0.00001	0.722502	K6		
CL32	v2772	Cultivars	na	C2	0.261374	0.00001	0.00001	0.00001	0.00001	0.738586	K6		
DS18	DelVesuvio25	Da serbo landraces	Italy	C2	0.00001	0.00001	0.00001	0.00001	0.00001	0.99995	K6		
DS11	VesuvioPOP8	Da serbo landraces	Italy	C2	0.00001	0.00001	0.005861	0.00001	0.00001	0.994099	K6		
HL14	VioletJasper	Heirloom	China	C2	0.00001	0.00001	0.00001	0.00001	0.00001	0.99995	K6		
FC30	Gialloagrappoli	Fresh consumption landraces	Italy	C2	0.00001	0.00001	0.00001	0.00001	0.00001	0.99995	K6		
DS19	ReginaOstuni	Da serbo landraces	Italy	C2	0.00001	0.00001	0.00001	0.00001	0.00001	0.99995	K6		
DS3	Regina	Da serbo landraces	Italy	C2	0.00001	0.00001	0.00001	0.00001	0.00001	0.99995	K6		
FC16	PiennoloPro2	Fresh consumption landraces	Italy	C2	0.00001	0.00001	0.000012	0.00001	0.00001	0.999948	K6		
FC55	Rosadet	Fresh consumption landraces	Spain	C2	0.00001	0.00001	0.00001	0.007126	0.00001	0.992834	K6		C2_B2.1
FC54	CireretaMenorca	Fresh consumption landraces	Spain	C2	0.00001	0.00001	0.00001	0.00001	0.00001	0.99995	K6		
DS45	BGV14802	Da serbo landraces	Spain	C2	0.00001	0.00001	0.00001	0.00001	0.00001	0.99995	K6		
FC6	CostFiorentinoNovoli	Fresh consumption landraces	Italy	C2	0.00001	0.00001	0.00001	0.00001	0.00001	0.99995	K6		
CL38	YL96	Cultivars	na	C2	0.00001	0.00001	0.00001	0.000015	0.00001	0.999944	K6		
HL46	Tomataki	Heirloom	Greece	C2	0.00001	0.00001	0.00001	0.00001	0.00001	0.99995	K6		
CL51	Santorini	Cultivars	Greece	C2	0.00001	0.00001	0.00001	0.00001	0.00001	0.99995	K6		
DS9	Pop25	Da serbo landraces	Italy	C2	0.00001	0.00001	0.00001	0.00001	0.00001	0.99995	K6		
DS8	Piennolo21	Da serbo landraces	Italy	C2	0.00001	0.00001	0.00001	0.00001	0.00001	0.99995	K6		
HL15	MichaelPollan (green zebra)	Heirloom	United States	C2	0.00001	0.00001	0.203078	0.00001	0.00001	0.796882	K6		
HL7	LA3472 (Movione NIL of Ailsa Craig)	Heirloom	United States	C2	0.00001	0.00001	0.107695	0.00001	0.00001	0.892265	K6		
BL6	LA1996	Breeding line	na	C2	0.00001	0.00001	0.00001	0.00001	0.00001	0.99995	K6		
HL12	BlancheduQuebe	Heirloom	Canada	C2	0.00001	0.00001	0.00001	0.00001	0.00001	0.99995	K6		C2_B2.2
HL45	Moneymaker	Heirloom	Netherlands	C2	0.00001	0.00001	0.00001	0.00001	0.00001	0.99995	K6		
HL39	v0085 (Princess of Wales)	Heirloom	United Kingdom	C2	0.00001	0.00001	0.00001	0.00001	0.00001	0.99995	K6		
HL3	RutgersI	Heirloom	United States	C2	0.00001	0.00001	0.00001	0.00001	0.00001	0.99995	K6		
CL31	v3092 (Marinadnyi 1)	Cultivars	Belarus	C2	0.00001	0.00001	0.00001	0.00001	0.00001	0.99995	K6		
