# Supplementary material for: ddRAD sequencing-based genotyping for population structure analysis in cultivated tomato provides new insights into the genomic diversity of Mediterranean ‘da serbo’ type long shelf-life germplasm
Source: Hortic Res. 2020 Sep 1;7:134. doi: 10.1038/s41438-020-00353-6 (PMC7459340; doi:10.1038/s41438-020-00353-6)
Supplement: Supplementary file 5 — Supplementary Table 5 [file 41438_2020_353_MOESM5_ESM.pdf]

Supplementary Table 5: Dissimilarity matrix showing level of genetic redundancy for the 288 tomato accessions

	FC1	FC10	HL8	HL31	CL7	HL32	CL8	CL9	CL10	CL11	CL12	FC36	FC11	FC37	FC38	FC39	FC40	FC41	FC42	FC43	FC44	FC57	FC46	FC12	FC47	FC48	FC49	FC50	FC51	FC52	FC53	FC54	FC55	FC56	FC13	FC45	FC58	FC59	FC60	FC61	FC62	FC63	FC64	FC65	FC66	FC14	FC67	FC68
**FC1**																																																
**FC10**	0.0079																																															
**HL8**	0.0431	0.0443																																														
**HL31**	0.0131	0.0130	0.0447																																													
**CL7**	0.0356	0.0356	0.0324	0.0395																																												
**HL32**	0.1482	0.1495	0.1532	0.1482	0.1389																																											
**CL8**	0.0382	0.0395	0.0369	0.0410	0.0259	0.1369																																										
**CL9**	0.0674	0.0676	0.0381	0.0706	0.0427	0.1689	0.0566																																									
**CL10**	0.0102	0.0102	0.0445	0.0151	0.0351	0.1459	0.0381	0.0670																																								
**CL11**	0.0134	0.0147	0.0395	0.0173	0.0318	0.1485	0.0319	0.0650	0.0126																																							
**CL12**	0.0513	0.0525	0.0600	0.0531	0.0684	0.1615	0.0743	0.0565	0.0503	0.0485																																						
**FC36**	0.0190	0.0195	0.0414	0.0217	0.0371	0.1571	0.0356	0.0645	0.0181	0.0157	0.0535																																					
**FC11**	0.0086	0.0070	0.0450	0.0154	0.0343	0.1465	0.0377	0.0669	0.0089	0.0132	0.0521	0.0196																																				
**FC37**	0.0156	0.0162	0.0406	0.0178	0.0315	0.1506	0.0317	0.0630	0.0153	0.0108	0.0505	0.0141	0.0163																																			
**FC38**	0.0177	0.0187	0.0409	0.0212	0.0354	0.1551	0.0338	0.0631	0.0175	0.0137	0.0519	0.0076	0.0181	0.0119																																		
**FC39**	0.0675	0.0689	0.0795	0.0705	0.0905	0.1455	0.0952	0.0780	0.0664	0.0692	0.0323	0.0730	0.0672	0.0734	0.0718																																	
**FC40**	0.0180	0.0189	0.0409	0.0216	0.0357	0.1548	0.0341	0.0630	0.0173	0.0138	0.0521	0.0079	0.0184	0.0120	0.0028	0.0721																																
**FC41**	0.0179	0.0189	0.0406	0.0214	0.0354	0.1545	0.0339	0.0627	0.0174	0.0137	0.0516	0.0077	0.0183	0.0121	0.0026	0.0718	0.0025																															
**FC42**	0.0421	0.0438	0.0721	0.0439	0.0659	0.1226	0.0682	0.0980	0.0411	0.0439	0.0519	0.0504	0.0419	0.0482	0.0490	0.0361	0.0489	0.0488																														
**FC43**	0.0125	0.0132	0.0457	0.0171	0.0374	0.1489	0.0407	0.0658	0.0113	0.0147	0.0496	0.0147	0.0112	0.0178	0.0115	0.0661	0.0115	0.0114	0.0439																													
**FC44**	0.1413	0.1435	0.1117	0.1405	0.1222	0.1777	0.1307	0.1041	0.1417	0.1417	0.1019	0.1434	0.1433	0.1421	0.1433	0.0892	0.1432	0.1429	0.1104	0.1417																												
**FC57**	0.0142	0.0143	0.0443	0.0170	0.0403	0.1501	0.0428	0.0699	0.0142	0.0187	0.0570	0.0226	0.0144	0.0214	0.0227	0.0714	0.0230	0.0227	0.0453	0.0168	0.1421																											
**FC46**	0.0178	0.0188	0.0408	0.0212	0.0354	0.1549	0.0339	0.0631	0.0176	0.0139	0.0518	0.0077	0.0182	0.0120	0.0026	0.0717	0.0029	0.0027	0.0488	0.0117	0.1428	0.0227																										
**FC12**	0.0147	0.0143	0.0385	0.0205	0.0355	0.1560	0.0356	0.0657	0.0169	0.0143	0.0541	0.0125	0.0136	0.0131	0.0115	0.0733	0.0117	0.0115	0.0499	0.0167	0.1425	0.0205	0.0115																									
**FC47**	0.0178	0.0185	0.0378	0.0215	0.0228	0.1392	0.0305	0.0559	0.0185	0.0213	0.0600	0.0260	0.0184	0.0236	0.0248	0.0760	0.0251	0.0250	0.0491	0.0205	0.1317	0.0239	0.0249	0.0263																								
**FC48**	0.0183	0.0190	0.0414	0.0217	0.0362	0.1550	0.0337	0.0630	0.0175	0.0145	0.0521	0.0074	0.0184	0.0125	0.0047	0.0725	0.0047	0.0047	0.0495	0.0132	0.1437	0.0233	0.0047	0.0122	0.0252																							
**FC49**	0.0093	0.0095	0.0442	0.0129	0.0342	0.1454	0.0363	0.0653	0.0086	0.0124	0.0508	0.0165	0.0093	0.0142	0.0139	0.0665	0.0138	0.0137	0.0400	0.0103	0.1430	0.0150	0.0139	0.0170	0.0160	0.0148																						
**FC50**	0.0179	0.0187	0.0406	0.0214	0.0353	0.1545	0.0337	0.0626	0.0174	0.0136	0.0514	0.0077	0.0182	0.0119	0.0025	0.0716	0.0025	0.0025	0.0485	0.0114	0.1429	0.0228	0.0026	0.0116	0.0249	0.0045	0.0137																					
**FC51**	0.0105	0.0099	0.0443	0.0144	0.0348	0.1458	0.0377	0.0662	0.0074	0.0127	0.0510	0.0174	0.0090	0.0157	0.0171	0.0668	0.0170	0.0169	0.0409	0.0106	0.1421	0.0137	0.0171	0.0174	0.0177	0.0172	0.0084	0.0168																				
**FC52**	0.0103	0.0099	0.0441	0.0135	0.0336	0.1463	0.0373	0.0661	0.0078	0.0124	0.0504	0.0177	0.0086	0.0145	0.0159	0.0662	0.0163	0.0162	0.0416	0.0108	0.1428	0.0140	0.0160	0.0168	0.0179	0.0166	0.0074	0.0161	0.0077																			
**FC53**	0.0268	0.0272	0.0442	0.0309	0.0508	0.1576	0.0548	0.0595	0.0250	0.0301	0.0446	0.0336	0.0271	0.0319	0.0328	0.0606	0.0329	0.0328	0.0589	0.0277	0.1365	0.0318	0.0328	0.0339	0.0342	0.0331	0.0252	0.0326	0.0252	0.0251																		
**FC54**	0.0147	0.0135	0.0454	0.0167	0.0383	0.1470	0.0418	0.0652	0.0120	0.0178	0.0543	0.0225	0.0129	0.0184	0.0221	0.0713	0.0223	0.0222	0.0444	0.0159	0.1399	0.0156	0.0222	0.0213	0.0216	0.0224	0.0135	0.0222	0.0120	0.0129	0.0284																	
**FC55**	0.0132	0.0132	0.0500	0.0177	0.0419	0.1377	0.0445	0.0627	0.0112	0.0163	0.0469	0.0220	0.0122	0.0182	0.0212	0.0597	0.0214	0.0211	0.0390	0.0140	0.1305	0.0163	0.0214	0.0210	0.0220	0.0216	0.0129	0.0212	0.0109	0.0118	0.0247	0.0058																
**FC56**	0.0099	0.0102	0.0488	0.0147	0.0380	0.1437	0.0404	0.0654	0.0086	0.0128	0.0480	0.0193	0.0097	0.0162	0.0175	0.0626	0.0175	0.0174	0.0403	0.0111	0.1408	0.0154	0.0176	0.0187	0.0178	0.0179	0.0084	0.0173	0.0076	0.0080	0.0248	0.0138	0.0117															
**FC13**	0.0150	0.0149	0.0401	0.0186	0.0311	0.1515	0.0335	0.0619	0.0152	0.0117	0.0503	0.0127	0.0154	0.0082	0.0102	0.0718	0.0104	0.0104	0.0482	0.0163	0.1415	0.0205	0.0102	0.0125	0.0229	0.0116	0.0140	0.0101	0.0150	0.0145	0.0306	0.0180	0.0181	0.0153														
**FC45**	0.0147	0.0153	0.0481	0.0135	0.0401	0.1491	0.0404	0.0681	0.0139	0.0165	0.0506	0.0161	0.0146	0.0186	0.0147	0.0670	0.0148	0.0147	0.0436	0.0101	0.1429	0.0198	0.0149	0.0194	0.0222	0.0150	0.0110	0.0147	0.0138	0.0126	0.0298	0.0188	0.0167	0.0142	0.0202													
**FC58**	0.0092	0.0095	0.0443	0.0128	0.0343	0.1455	0.0364	0.0655	0.0085	0.0122	0.0508	0.0165	0.0091	0.0142	0.0139	0.0665	0.0139	0.0138	0.0399	0.0102	0.1428	0.0149	0.0139	0.0170	0.0159	0.0149	0.0022	0.0136	0.0083	0.0074	0.0252	0.0133	0.0123	0.0082	0.0140	0.0111												
**FC59**	0.0114	0.0109	0.0455	0.0128	0.0352	0.1470	0.0385	0.0663	0.0097	0.0145	0.0510	0.0172	0.0113	0.0149	0.0176	0.0686	0.0176	0.0174	0.0418	0.0130	0.1424	0.0156	0.0176	0.0179	0.0168	0.0179	0.0085	0.0175	0.0081	0.0095	0.0259	0.0120	0.0122	0.0087	0.0147	0.0143	0.0086											
**FC60**	0.0202	0.0209	0.0435	0.0231	0.0383	0.1575	0.0365	0.0658	0.0193	0.0165	0.0544	0.0091	0.0201	0.0150	0.0067	0.0743	0.0069	0.0068	0.0514	0.0146	0.1454	0.0241	0.0067	0.0135	0.0271	0.0080	0.0174	0.0066	0.0183	0.0182	0.0346	0.0229	0.0230	0.0201	0.0138	0.0173	0.0173	0.0193										
**FC61**	0.0092	0.0100	0.0449	0.0136	0.0351	0.1471	0.0359	0.0669	0.0083	0.0129	0.0508	0.0163	0.0095	0.0157	0.0162	0.0672	0.0165	0.0163	0.0407	0.0120	0.1428	0.0148	0.0163	0.0171	0.0163	0.0162	0.0069	0.0162	0.0084	0.0088	0.0258	0.0137	0.0131	0.0079	0.0144	0.0132	0.0069	0.0083	0.0177									
**FC62**	0.0134	0.0119	0.0426	0.0152	0.0320	0.1479	0.0354	0.0628	0.0117	0.0118	0.0495	0.0145	0.0125	0.0113	0.0136	0.0701	0.0138	0.0138	0.0449	0.0134	0.1410	0.0174	0.0137	0.0150	0.0184	0.0141	0.0110	0.0137	0.0107	0.0109	0.0274	0.0137	0.0140	0.0122	0.0098	0.0166	0.0109	0.0108	0.0152	0.0114								
**FC63**	0.0101	0.0095	0.0443	0.0137	0.0344	0.1454	0.0379	0.0661	0.0080	0.0131	0.0514	0.0166	0.0093	0.0151	0.0163	0.0657	0.0165	0.0164	0.0414	0.0109	0.1422	0.0136	0.0163	0.0165	0.0178	0.0168	0.0077	0.0164	0.0066	0.0075	0.0246	0.0122	0.0114	0.0080	0.0142	0.0135	0.0076	0.0088	0.0176	0.0082	0.0103							
**FC64**	0.0103	0.0099	0.0443	0.0139	0.0346	0.1459	0.0377	0.0659	0.0082	0.0127	0.0510	0.0159	0.0095	0.0155	0.0163	0.0668	0.0163	0.0162	0.0417	0.0101	0.1427	0.0139	0.0165	0.0173	0.0176	0.0166	0.0077	0.0162	0.0064	0.0068	0.0249	0.0122	0.0113	0.0086	0.0140	0.0128	0.0077	0.0088	0.0175	0.0081	0.0097	0.0052						
**FC65**	0.0104	0.0098	0.0439	0.0137	0.0343	0.1459	0.0378	0.0658	0.0079	0.0127	0.0511	0.0163	0.0093	0.0152	0.0163	0.0662	0.0162	0.0161	0.0415	0.0106	0.1425	0.0137	0.0163	0.0166	0.0178	0.0166	0.0074	0.0161	0.0063	0.0076	0.0247	0.0123	0.0114	0.0079	0.0141	0.0133	0.0074	0.0086	0.0176	0.0081	0.0104	0.0023	0.0050					
**FC66**	0.0105	0.0093	0.0456	0.0132	0.0343	0.1462	0.0385	0.0656	0.0079	0.0140	0.0500	0.0182	0.0093	0.0151	0.0183	0.0682	0.0182	0.0181	0.0425	0.0113	0.1412	0.0148	0.0183	0.0175	0.0163	0.0184	0.0088	0.0181	0.0075	0.0088	0.0251	0.0106	0.0108	0.0083	0.0138	0.0143	0.0089	0.0073	0.0189	0.0080	0.0089	0.0079	0.0077	0.0077				
**FC14**	0.0090	0.0072	0.0453	0.0154	0.0353	0.1463	0.0374	0.0665	0.0095	0.0136	0.0522	0.0194	0.0048	0.0160	0.0178	0.0670	0.0182	0.0181	0.0419	0.0120	0.1432	0.0149	0.0179	0.0147	0.0180	0.0180	0.0088	0.0179	0.0091	0.0091	0.0267	0.0125	0.0115	0.0093	0.0150	0.0147	0.0087	0.0111	0.0200	0.0091	0.0121	0.0093	0.0094	0.0096	0.0093			
**FC67**	0.0151	0.0159	0.0426	0.0185	0.0304	0.1520	0.0332	0.0609	0.0135	0.0118	0.0502	0.0151	0.0149	0.0118	0.0140	0.0728	0.0142	0.0140	0.0476	0.0164	0.1440	0.0195	0.0141	0.0158	0.0236	0.0125	0.0132	0.0140	0.0135	0.0133	0.0312	0.0182	0.0186	0.0145	0.0123	0.0176	0.0132	0.0154	0.0167	0.0127	0.0130	0.0134	0.0132	0.0131	0.0143	0.0141		
**FC68**	0.0106	0.0110	0.0453	0.0115	0.0351	0.1453	0.0367	0.0668	0.0092	0.0115	0.0494	0.0189	0.0096	0.0143	0.0175	0.0668	0.0178	0.0177	0.0413	0.0114	0.1421	0.0149	0.0176	0.0183	0.0187	0.0179	0.0087	0.0176	0.0090	0.0081	0.0268	0.0138	0.0130	0.0087	0.0156	0.0110	0.0086	0.0110	0.0200	0.0092	0.0121	0.0092	0.0091	0.0091	0.0098	0.0099	0.0125	
**FC69**	0.0106	0.0098	0.0424	0.0121	0.0375	0.1488	0.0399	0.0689	0.0102	0.0155	0.0531	0.0187	0.0112	0.0179	0.0189	0.0691	0.0192	0.0189	0.0438	0.0134	0.1404	0.0126	0.0188	0.0168	0.0202	0.0192	0.0108	0.0189	0.0086	0.0093	0.0276	0.0145	0.0147	0.0108	0.0168	0.0157	0.0108	0.0107	0.0199	0.0098	0.0131	0.0089	0.0090	0.0088	0.0095	0.0111	0.0156	0.0110
**FC70**	0.0105	0.0099	0.0425	0.0122	0.0374	0.1489	0.0396	0.0691	0.0101	0.0155	0.0532	0.0186	0.0111	0.0177	0.0187	0.0696	0.0190	0.0188	0.0441	0.0133	0.1407	0.0128	0.0187	0.0167	0.0201	0.0190	0.0108	0.0187	0.0085	0.0093	0.0277	0.0145	0.0146	0.0106	0.0167	0.0158	0.0107	0.0107	0.0197	0.0097	0.0132	0.0088	0.0090	0.0088	0.0095	0.0110	0.0155	0.0108
**FC71**	0.0179	0.0191	0.0411	0.0204	0.0356	0.1551	0.0324	0.0632	0.0178	0.0128	0.0511	0.0084	0.0183	0.0112	0.0053	0.0722	0.0057	0.0055	0.0494	0.0133	0.1435	0.0230	0.0054	0.0119	0.0255	0.0069	0.0151	0.0056	0.0174	0.0161	0.0332	0.0223	0.0215	0.0184	0.0117	0.0135	0.0152	0.0175	0.0087	0.0163	0.0145	0.0168	0.0167	0.0167	0.0184	0.0180	0.0134	0.0167
**FC72**	0.0171	0.0183	0.0418	0.0207	0.0309	0.1544	0.0359	0.0611	0.0174	0.0145	0.0516	0.0135	0.0182	0.0145	0.0118	0.0731	0.0118	0.0117	0.0481	0.0157	0.1426	0.0228	0.0118	0.0149	0.0231	0.0126	0.0144	0.0117	0.0168	0.0160	0.0316	0.0218	0.0209	0.0163	0.0137	0.0175	0.0144	0.0165	0.0145	0.0153	0.0148	0.0159	0.0155	0.0157	0.0169	0.0177	0.0112	0.0172
**FC73**	0.0148	0.0156	0.0483	0.0138	0.0401	0.1494	0.0406	0.0682	0.0140	0.0165	0.0509	0.0160	0.0151	0.0184	0.0151	0.0669	0.0152	0.0152	0.0440	0.0108	0.1434	0.0203	0.0152	0.0198	0.0224	0.0156	0.0113	0.0152	0.0143	0.0133	0.0299	0.0190	0.0173	0.0146	0.0202	0.0038	0.0113	0.0147	0.0177	0.0132	0.0168	0.0139	0.0134	0.0137	0.0150	0.0149	0.0180	0.0113
**FC74**	0.0149	0.0147	0.0391	0.0193	0.0315	0.1514	0.0315	0.0632	0.0134	0.0106	0.0521	0.0117	0.0141	0.0096	0.0094	0.0714	0.0096	0.0094	0.0466	0.0154	0.1428	0.0192	0.0097	0.0116	0.0227	0.0099	0.0121	0.0095	0.0123	0.0125	0.0304	0.0179	0.0178	0.0137	0.0079	0.0180	0.0120	0.0145	0.0127	0.0133	0.0109	0.0120	0.0124	0.0118	0.0139	0.0142	0.0095	0.0139
**FC75**	0.0748	0.0752	0.0730	0.0714	0.0630	0.1090	0.0485	0.0945	0.0753	0.0726	0.0846	0.0770	0.0758	0.0714	0.0765	0.0780	0.0763	0.0761	0.0528	0.0783	0.1017	0.0768	0.0763	0.0741	0.0687	0.0769	0.0758	0.0761	0.0756	0.0752	0.0928	0.0767	0.0767	0.0759	0.0722	0.0777	0.0757	0.0753	0.0789	0.0764	0.0727	0.0757	0.0759	0.0756	0.0740	0.0754	0.0722	0.0740
**DS20**	0.0122	0.0117	0.0477	0.0158	0.0379	0.1517	0.0398	0.0687	0.0092	0.0146	0.0540	0.0184	0.0113	0.0173	0.0185	0.0690	0.0185	0.0184	0.0450	0.0129	0.1450	0.0157	0.0185	0.0192	0.0201	0.0187	0.0102	0.0185	0.0081	0.0094	0.0274	0.0128	0.0113	0.0101	0.0165	0.0154	0.0101	0.0104	0.0202	0.0104	0.0124	0.0088	0.0082	0.0087	0.0101	0.0108	0.0155	0.0108
**DS7**	0.0190	0.0179	0.0393	0.0222	0.0345	0.1521	0.0350	0.0614	0.0170	0.0148	0.0551	0.0172	0.0172	0.0130	0.0165	0.0757	0.0166	0.0167	0.0487	0.0210	0.1394	0.0196	0.0165	0.0154	0.0267	0.0170	0.0186	0.0166	0.0176	0.0179	0.0345	0.0146	0.0164	0.0198	0.0116	0.0240	0.0186	0.0178	0.0180	0.0184	0.0137	0.0175	0.0178	0.0174	0.0163	0.0172	0.0158	0.0185
**DS21**	0.0283	0.0291	0.0354	0.0313	0.0133	0.1429	0.0265	0.0470	0.0277	0.0258	0.0646	0.0293	0.0284	0.0232	0.0276	0.0859	0.0277	0.0279	0.0605	0.0310	0.1287	0.0334	0.0276	0.0283	0.0194	0.0281	0.0272	0.0276	0.0283	0.0280	0.0441	0.0307	0.0328	0.0295	0.0243	0.0329	0.0271	0.0274	0.0302	0.0274	0.0246	0.0282	0.0285	0.0282	0.0270	0.0280	0.0234	0.0290
**DS22**	0.0128	0.0121	0.0439	0.0159	0.0364	0.1484	0.0395	0.0679	0.0123	0.0181	0.0558	0.0211	0.0130	0.0197	0.0212	0.0710	0.0213	0.0211	0.0457	0.0156	0.1413	0.0132	0.0212	0.0193	0.0210	0.0216	0.0125	0.0213	0.0118	0.0124	0.0299	0.0172	0.0182	0.0137	0.0190	0.0177	0.0123	0.0144	0.0229	0.0127	0.0153	0.0118	0.0120	0.0117	0.0127	0.0131	0.0180	0.0131
**DS23**	0.0144	0.0148	0.0478	0.0182	0.0385	0.1477	0.0407	0.0684	0.0144	0.0179	0.0539	0.0192	0.0149	0.0193	0.0170	0.0711	0.0172	0.0170	0.0460	0.0129	0.1454	0.0194	0.0169	0.0196	0.0214	0.0177	0.0124	0.0170	0.0139	0.0133	0.0239	0.0182	0.0170	0.0144	0.0193	0.0150	0.0123	0.0147	0.0191	0.0136	0.0154	0.0135	0.0130	0.0134	0.0141	0.0145	0.0188	0.0139
**DS24**	0.0183	0.0184	0.0444	0.0224	0.0321	0.1516	0.0368	0.0625	0.0173	0.0168	0.0547	0.0176	0.0184	0.0163	0.0170	0.0754	0.0170	0.0170	0.0508	0.0197	0.1449	0.0218	0.0172	0.0180	0.0241	0.0175	0.0164	0.0171	0.0165	0.0166	0.0291	0.0210	0.0211	0.0177	0.0145	0.0222	0.0162	0.0180	0.0188	0.0168	0.0149	0.0160	0.0159	0.0159	0.0165	0.0178	0.0117	0.0178
**DS25**	0.0177	0.0171	0.0426	0.0228	0.0290	0.1513	0.0340	0.0594	0.0146	0.0153	0.0535	0.0176	0.0159	0.0148	0.0166	0.0737	0.0167	0.0164	0.0484	0.0190	0.1417	0.0203	0.0167	0.0164	0.0245	0.0167	0.0157	0.0166	0.0149	0.0159	0.0330	0.0201	0.0212	0.0172	0.0144	0.0212	0.0156	0.0170	0.0186	0.0156	0.0148	0.0153	0.0157	0.0151	0.0161	0.0157	0.0102	0.0160
**DS26**	0.0111	0.0112	0.0457	0.0148	0.0357	0.1459	0.0359	0.0665	0.0089	0.0127	0.0511	0.0190	0.0092	0.0151	0.0176	0.0679	0.0177	0.0176	0.0427	0.0121	0.1432	0.0144	0.0176	0.0181	0.0190	0.0177	0.0093	0.0177	0.0089	0.0090	0.0267	0.0122	0.0114	0.0100	0.0160	0.0132	0.0092	0.0111	0.0200	0.0103	0.0121	0.0094	0.0092	0.0093	0.0100	0.0088	0.0129	0.0089
**DS27**	0.0187	0.0186	0.0426	0.0226	0.0322	0.1574	0.0363	0.0653	0.0176	0.0141	0.0545	0.0184	0.0188	0.0133	0.0166	0.0753	0.0166	0.0167	0.0517	0.0203	0.1450	0.0235	0.0168	0.0170	0.0248	0.0175	0.0185	0.0165	0.0186	0.0172	0.0348	0.0212	0.0211	0.0180	0.0105	0.0238	0.0183	0.0187	0.0199	0.0180	0.0148	0.0177	0.0182	0.0176	0.0173	0.0191	0.0173	0.0189
**DS28**	0.0152	0.0151	0.0516	0.0193	0.0391	0.1516	0.0420	0.0722	0.0150	0.0180	0.0584	0.0229	0.0149	0.0190	0.0223	0.0755	0.0225	0.0223	0.0499	0.0171	0.1541	0.0194	0.0222	0.0218	0.0233	0.0227	0.0144	0.0223	0.0138	0.0144	0.0232	0.0176	0.0170	0.0154	0.0192	0.0188	0.0142	0.0153	0.0241	0.0142	0.0154	0.0136	0.0134	0.0137	0.0138	0.0143	0.0186	0.0140
**DS29**	0.0180	0.0174	0.0441	0.0227	0.0318	0.1541	0.0378	0.0594	0.0166	0.0160	0.0508	0.0117	0.0167	0.0159	0.0115	0.0708	0.0115	0.0115	0.0495	0.0137	0.1421	0.0219	0.0114	0.0141	0.0258	0.0117	0.0162	0.0115	0.0151	0.0160	0.0318	0.0203	0.0196	0.0171	0.0150	0.0157	0.0162	0.0176	0.0131	0.0164	0.0150	0.0146	0.0152	0.0145	0.0167	0.0169	0.0114	0.0169
**DS30**	0.0394	0.0401	0.0505	0.0440	0.0588	0.1464	0.0608	0.0680	0.0382	0.0372	0.0294	0.0415	0.0389	0.0401	0.0406	0.0350	0.0407	0.0405	0.0387	0.0404	0.1125	0.0432	0.0405	0.0406	0.0470	0.0410	0.0386	0.0404	0.0378	0.0388	0.0376	0.0427	0.0388	0.0377	0.0392	0.0422	0.0384	0.0402	0.0426	0.0388	0.0376	0.0376	0.0385	0.0377	0.0393	0.0388	0.0404	0.0385
**FC15**	0.0174	0.0172	0.0384	0.0210	0.0350	0.1513	0.0345	0.0617	0.0161	0.0141	0.0542	0.0143	0.0169	0.0117	0.0143	0.0748	0.0144	0.0145	0.0482	0.0190	0.1397	0.0188	0.0143	0.0138	0.0248	0.0147	0.0165	0.0143	0.0157	0.0161	0.0334	0.0148	0.0159	0.0166	0.0100	0.0219	0.0165	0.0159	0.0159	0.0160	0.0126	0.0151	0.0155	0.0151	0.0145	0.0167	0.0138	0.0168
**DS26**	0.0107	0.0107	0.0453	0.0152	0.0353	0.1463	0.0365	0.0665	0.0087	0.0131	0.0516	0.0183	0.0089	0.0155	0.0170	0.0676	0.0171	0.0171	0.0422	0.0119	0.1427	0.0140	0.0170	0.0175	0.0184	0.0172	0.0090	0.0171	0.0087	0.0087	0.0264	0.0118	0.0109	0.0096	0.0152	0.0140	0.0089	0.0108	0.0193	0.0100	0.0113	0.0091	0.0088	0.0092	0.0098	0.0083	0.0135	0.0094
**DS32**	0.0158	0.0150	0.0484	0.0184	0.0402	0.1470	0.0431	0.0685	0.0123	0.0184	0.0550	0.0228	0.0151	0.0197	0.0226	0.0728	0.0224	0.0224	0.0458	0.0164	0.1440	0.0180	0.0225	0.0229	0.0220	0.0227	0.0140	0.0224	0.0124	0.0132	0.0222	0.0121	0.0116	0.0138	0.0189	0.0194	0.0140	0.0129	0.0238	0.0143	0.0150	0.0131	0.0128	0.0130	0.0119	0.0147	0.0190	0.0143
**DS29**	0.0173	0.0180	0.0451	0.0217	0.0372	0.1493	0.0400	0.0648	0.0165	0.0160	0.0518	0.0187	0.0173	0.0183	0.0172	0.0722	0.0172	0.0171	0.0465	0.0158	0.1435	0.0210	0.0171	0.0179	0.0255	0.0176	0.0156	0.0172	0.0158	0.0157	0.0328	0.0191	0.0170	0.0153	0.0189	0.0180	0.0156	0.0173	0.0190	0.0161	0.0162	0.0155	0.0159	0.0154	0.0171	0.0176	0.0166	0.0160
**DS34**	0.0204	0.0199	0.0535	0.0249	0.0417	0.1475	0.0456	0.0712	0.0191	0.0241	0.0590	0.0253	0.0193	0.0251	0.0239	0.0744	0.0241	0.0240	0.0516	0.0184	0.1486	0.0248	0.0243	0.0260	0.0270	0.0240	0.0185	0.0241	0.0189	0.0187	0.0219	0.0227	0.0210	0.0194	0.0244	0.0200	0.0183	0.0201	0.0263	0.0195	0.0212	0.0186	0.0187	0.0186	0.0191	0.0190	0.0247	0.0198

**DS35**	0.1088	0.1102	0.1093	0.1134	0.0947	0.2287	0.1096	0.1308	0.1099	0.1114	0.1580	0.1200	0.1096	0.1165	0.1191	0.1732	0.1191	0.1190	0.1440	0.1118	0.1952	0.1095	0.1192	0.1180	0.0973	0.1198	0.1084	0.1189	0.1097	0.1092	0.1287	0.1092	0.1053	0.1086	0.1163	0.1150	0.1086	0.1111	0.1222	0.1104	0.1121	0.1090	0.1090	0.1089	0.1099	0.1103	0.1161	0.1107
**DS36**	0.0095	0.0095	0.0445	0.0128	0.0355	0.1460	0.0364	0.0661	0.0086	0.0118	0.0497	0.0177	0.0089	0.0139	0.0161	0.0670	0.0164	0.0163	0.0417	0.0101	0.1428	0.0142	0.0163	0.0170	0.0175	0.0167	0.0079	0.0164	0.0076	0.0080	0.0255	0.0119	0.0110	0.0085	0.0144	0.0123	0.0076	0.0099	0.0185	0.0087	0.0107	0.0071	0.0072	0.0070	0.0082	0.0084	0.0120	0.0076
**DS37**	0.0120	0.0114	0.0459	0.0150	0.0368	0.1472	0.0383	0.0677	0.0092	0.0142	0.0526	0.0174	0.0108	0.0169	0.0178	0.0687	0.0179	0.0178	0.0430	0.0122	0.1443	0.0145	0.0179	0.0184	0.0193	0.0178	0.0095	0.0179	0.0081	0.0088	0.0259	0.0114	0.0106	0.0096	0.0157	0.0148	0.0094	0.0100	0.0182	0.0094	0.0113	0.0080	0.0077	0.0080	0.0093	0.0102	0.0141	0.0099
**DS38**	0.0194	0.0192	0.0418	0.0222	0.0358	0.1544	0.0336	0.0632	0.0171	0.0139	0.0518	0.0102	0.0183	0.0126	0.0092	0.0725	0.0095	0.0094	0.0503	0.0147	0.1420	0.0227	0.0094	0.0132	0.0270	0.0098	0.0175	0.0095	0.0173	0.0171	0.0337	0.0209	0.0205	0.0179	0.0131	0.0160	0.0174	0.0194	0.0114	0.0182	0.0152	0.0170	0.0173	0.0169	0.0180	0.0179	0.0137	0.0168
**DS39**	0.0247	0.0240	0.0469	0.0266	0.0420	0.1554	0.0429	0.0739	0.0235	0.0219	0.0626	0.0224	0.0252	0.0199	0.0218	0.0830	0.0219	0.0219	0.0573	0.0267	0.1481	0.0257	0.0220	0.0212	0.0329	0.0226	0.0241	0.0219	0.0231	0.0241	0.0274	0.0273	0.0272	0.0246	0.0188	0.0299	0.0238	0.0241	0.0241	0.0240	0.0207	0.0224	0.0233	0.0223	0.0232	0.0252	0.0212	0.0248
**DS40**	0.0192	0.0199	0.0533	0.0223	0.0446	0.1499	0.0464	0.0719	0.0190	0.0226	0.0567	0.0199	0.0196	0.0240	0.0180	0.0739	0.0180	0.0179	0.0501	0.0142	0.1501	0.0246	0.0181	0.0241	0.0262	0.0183	0.0162	0.0180	0.0181	0.0182	0.0206	0.0226	0.0200	0.0183	0.0234	0.0158	0.0161	0.0183	0.0203	0.0175	0.0196	0.0178	0.0174	0.0177	0.0184	0.0193	0.0233	0.0189
**FC16**	0.0123	0.0127	0.0433	0.0168	0.0383	0.1470	0.0408	0.0659	0.0100	0.0156	0.0535	0.0208	0.0114	0.0187	0.0209	0.0702	0.0211	0.0210	0.0433	0.0146	0.1406	0.0146	0.0209	0.0198	0.0208	0.0214	0.0123	0.0210	0.0110	0.0112	0.0283	0.0102	0.0094	0.0110	0.0171	0.0172	0.0121	0.0128	0.0222	0.0113	0.0145	0.0112	0.0114	0.0113	0.0113	0.0122	0.0171	0.0121
**DS41**	0.0196	0.0196	0.0422	0.0224	0.0362	0.1547	0.0341	0.0639	0.0171	0.0140	0.0519	0.0104	0.0186	0.0128	0.0094	0.0730	0.0095	0.0095	0.0505	0.0146	0.1426	0.0228	0.0096	0.0135	0.0271	0.0099	0.0176	0.0095	0.0174	0.0173	0.0339	0.0210	0.0207	0.0182	0.0135	0.0159	0.0174	0.0193	0.0116	0.0184	0.0154	0.0171	0.0173	0.0170	0.0181	0.0181	0.0138	0.0170
**DS42**	0.0179	0.0187	0.0399	0.0219	0.0330	0.1523	0.0315	0.0648	0.0170	0.0138	0.0564	0.0145	0.0173	0.0131	0.0131	0.0746	0.0132	0.0131	0.0476	0.0192	0.1429	0.0212	0.0132	0.0142	0.0242	0.0143	0.0161	0.0131	0.0151	0.0169	0.0342	0.0205	0.0210	0.0175	0.0127	0.0218	0.0158	0.0165	0.0149	0.0160	0.0145	0.0154	0.0163	0.0153	0.0171	0.0173	0.0146	0.0180
**DS43**	0.0202	0.0197	0.0532	0.0245	0.0416	0.1475	0.0455	0.0710	0.0188	0.0238	0.0588	0.0248	0.0190	0.0248	0.0237	0.0742	0.0236	0.0235	0.0515	0.0178	0.1485	0.0243	0.0237	0.0258	0.0266	0.0237	0.0180	0.0236	0.0184	0.0183	0.0218	0.0225	0.0214	0.0194	0.0241	0.0198	0.0178	0.0198	0.0259	0.0192	0.0209	0.0183	0.0183	0.0182	0.0187	0.0188	0.0243	0.0195
**DS44**	0.0190	0.0179	0.0416	0.0223	0.0348	0.1549	0.0351	0.0628	0.0163	0.0144	0.0519	0.0097	0.0174	0.0125	0.0090	0.0720	0.0092	0.0092	0.0498	0.0139	0.1421	0.0219	0.0092	0.0121	0.0263	0.0093	0.0171	0.0091	0.0150	0.0164	0.0325	0.0205	0.0195	0.0169	0.0120	0.0163	0.0170	0.0168	0.0107	0.0168	0.0135	0.0155	0.0163	0.0156	0.0161	0.0173	0.0140	0.0173
**DS45**	0.0134	0.0126	0.0448	0.0155	0.0378	0.1469	0.0408	0.0638	0.0103	0.0163	0.0522	0.0208	0.0128	0.0166	0.0205	0.0706	0.0207	0.0206	0.0437	0.0145	0.1380	0.0148	0.0206	0.0205	0.0198	0.0209	0.0122	0.0206	0.0109	0.0115	0.0275	0.0078	0.0086	0.0115	0.0159	0.0173	0.0120	0.0105	0.0222	0.0120	0.0126	0.0112	0.0112	0.0112	0.0090	0.0124	0.0171	0.0122
**DS46**	0.0178	0.0183	0.0396	0.0216	0.0328	0.1523	0.0315	0.0646	0.0168	0.0136	0.0564	0.0142	0.0169	0.0128	0.0129	0.0745	0.0131	0.0129	0.0477	0.0190	0.1432	0.0208	0.0130	0.0138	0.0239	0.0142	0.0159	0.0129	0.0149	0.0166	0.0341	0.0201	0.0213	0.0176	0.0123	0.0216	0.0157	0.0162	0.0146	0.0158	0.0141	0.0152	0.0161	0.0150	0.0168	0.0169	0.0144	0.0177
**DS47**	0.0229	0.0220	0.0473	0.0261	0.0386	0.1522	0.0402	0.0703	0.0202	0.0188	0.0593	0.0193	0.0219	0.0169	0.0189	0.0786	0.0189	0.0189	0.0542	0.0236	0.1503	0.0265	0.0190	0.0197	0.0301	0.0196	0.0204	0.0189	0.0197	0.0205	0.0222	0.0221	0.0213	0.0217	0.0157	0.0261	0.0203	0.0212	0.0201	0.0213	0.0174	0.0187	0.0197	0.0187	0.0203	0.0217	0.0188	0.0216
**DS48**	0.0182	0.0188	0.0398	0.0219	0.0330	0.1523	0.0316	0.0645	0.0170	0.0138	0.0564	0.0145	0.0173	0.0131	0.0132	0.0746	0.0133	0.0130	0.0477	0.0192	0.1431	0.0213	0.0133	0.0141	0.0241	0.0144	0.0160	0.0132	0.0151	0.0169	0.0343	0.0205	0.0215	0.0180	0.0127	0.0217	0.0158	0.0164	0.0149	0.0160	0.0144	0.0155	0.0163	0.0153	0.0170	0.0174	0.0145	0.0179
**DS49**	0.0199	0.0190	0.0527	0.0214	0.0459	0.1505	0.0490	0.0774	0.0191	0.0241	0.0616	0.0278	0.0205	0.0262	0.0279	0.0780	0.0279	0.0277	0.0525	0.0215	0.1479	0.0207	0.0279	0.0269	0.0285	0.0284	0.0191	0.0280	0.0182	0.0195	0.0227	0.0224	0.0214	0.0193	0.0251	0.0250	0.0189	0.0192	0.0297	0.0194	0.0216	0.0179	0.0185	0.0178	0.0184	0.0204	0.0242	0.0197
**DS50**	0.0193	0.0183	0.0416	0.0227	0.0348	0.1549	0.0352	0.0626	0.0164	0.0146	0.0520	0.0099	0.0176	0.0128	0.0091	0.0721	0.0092	0.0090	0.0498	0.0138	0.1420	0.0222	0.0091	0.0125	0.0263	0.0092	0.0172	0.0091	0.0150	0.0166	0.0326	0.0207	0.0199	0.0171	0.0124	0.0163	0.0172	0.0170	0.0107	0.0171	0.0137	0.0159	0.0165	0.0157	0.0163	0.0177	0.0141	0.0175
**FC17**	0.0177	0.0175	0.0386	0.0213	0.0353	0.1519	0.0345	0.0619	0.0165	0.0142	0.0544	0.0146	0.0173	0.0118	0.0144	0.0753	0.0146	0.0146	0.0483	0.0194	0.1400	0.0193	0.0144	0.0140	0.0252	0.0149	0.0169	0.0145	0.0160	0.0167	0.0338	0.0150	0.0160	0.0171	0.0102	0.0222	0.0167	0.0164	0.0161	0.0163	0.0129	0.0155	0.0159	0.0156	0.0149	0.0170	0.0140	0.0171
**DS51**	0.0236	0.0229	0.0489	0.0266	0.0364	0.1518	0.0426	0.0665	0.0221	0.0210	0.0587	0.0224	0.0221	0.0206	0.0223	0.0799	0.0223	0.0222	0.0545	0.0245	0.1493	0.0265	0.0224	0.0220	0.0309	0.0223	0.0214	0.0222	0.0211	0.0205	0.0251	0.0229	0.0226	0.0229	0.0196	0.0268	0.0214	0.0224	0.0233	0.0220	0.0185	0.0199	0.0199	0.0199	0.0210	0.0217	0.0157	0.0220
**DS52**	0.0155	0.0142	0.0427	0.0193	0.0296	0.1515	0.0353	0.0614	0.0113	0.0134	0.0513	0.0146	0.0140	0.0131	0.0148	0.0715	0.0148	0.0148	0.0470	0.0169	0.1430	0.0192	0.0148	0.0149	0.0228	0.0149	0.0128	0.0147	0.0127	0.0122	0.0291	0.0163	0.0172	0.0142	0.0124	0.0183	0.0128	0.0144	0.0163	0.0133	0.0118	0.0121	0.0125	0.0119	0.0129	0.0140	0.0091	0.0138
**DS53**	0.0163	0.0160	0.0413	0.0203	0.0325	0.1528	0.0344	0.0624	0.0139	0.0129	0.0514	0.0108	0.0162	0.0120	0.0097	0.0720	0.0099	0.0097	0.0477	0.0149	0.1430	0.0207	0.0097	0.0116	0.0235	0.0103	0.0136	0.0097	0.0145	0.0141	0.0303	0.0186	0.0185	0.0157	0.0118	0.0166	0.0135	0.0158	0.0119	0.0146	0.0121	0.0138	0.0142	0.0137	0.0154	0.0159	0.0115	0.0153
**DS54**	0.0192	0.0182	0.0417	0.0226	0.0350	0.1552	0.0352	0.0627	0.0166	0.0146	0.0522	0.0099	0.0176	0.0128	0.0091	0.0721	0.0093	0.0092	0.0500	0.0140	0.1422	0.0222	0.0092	0.0124	0.0265	0.0093	0.0173	0.0091	0.0152	0.0167	0.0326	0.0207	0.0195	0.0173	0.0123	0.0165	0.0173	0.0172	0.0108	0.0171	0.0137	0.0158	0.0166	0.0158	0.0164	0.0176	0.0142	0.0176
**DS55**	0.0101	0.0098	0.0443	0.0150	0.0341	0.1454	0.0367	0.0653	0.0089	0.0136	0.0515	0.0184	0.0098	0.0152	0.0172	0.0672	0.0173	0.0173	0.0421	0.0120	0.1421	0.0144	0.0174	0.0177	0.0174	0.0174	0.0085	0.0173	0.0082	0.0086	0.0258	0.0134	0.0123	0.0095	0.0153	0.0136	0.0082	0.0108	0.0192	0.0093	0.0116	0.0080	0.0082	0.0081	0.0095	0.0093	0.0140	0.0091
**DS56**	0.0157	0.0153	0.0394	0.0200	0.0319	0.1504	0.0318	0.0635	0.0134	0.0108	0.0528	0.0129	0.0145	0.0102	0.0118	0.0719	0.0119	0.0118	0.0462	0.0165	0.1417	0.0189	0.0119	0.0121	0.0230	0.0123	0.0135	0.0118	0.0119	0.0138	0.0308	0.0177	0.0176	0.0138	0.0096	0.0193	0.0133	0.0145	0.0138	0.0138	0.0114	0.0118	0.0128	0.0118	0.0135	0.0144	0.0110	0.0139
**DS57**	0.0157	0.0145	0.0427	0.0195	0.0297	0.1517	0.0354	0.0614	0.0113	0.0133	0.0514	0.0147	0.0143	0.0133	0.0147	0.0715	0.0148	0.0147	0.0469	0.0169	0.1428	0.0194	0.0149	0.0151	0.0229	0.0149	0.0129	0.0148	0.0128	0.0124	0.0291	0.0163	0.0170	0.0141	0.0127	0.0184	0.0129	0.0145	0.0165	0.0134	0.0119	0.0122	0.0126	0.0121	0.0131	0.0142	0.0092	0.0140
**DS58**	0.0175	0.0179	0.0393	0.0214	0.0320	0.1516	0.0310	0.0640	0.0164	0.0134	0.0560	0.0140	0.0166	0.0125	0.0131	0.0740	0.0131	0.0130	0.0470	0.0192	0.1429	0.0209	0.0131	0.0137	0.0235	0.0138	0.0150	0.0130	0.0150	0.0165	0.0338	0.0200	0.0210	0.0175	0.0124	0.0211	0.0149	0.0160	0.0151	0.0155	0.0142	0.0150	0.0157	0.0148	0.0167	0.0165	0.0141	0.0172
**DS59**	0.0228	0.0224	0.0471	0.0261	0.0393	0.1516	0.0396	0.0707	0.0191	0.0184	0.0592	0.0198	0.0219	0.0169	0.0183	0.0791	0.0184	0.0183	0.0536	0.0228	0.1493	0.0263	0.0185	0.0197	0.0301	0.0192	0.0207	0.0183	0.0191	0.0202	0.0240	0.0229	0.0224	0.0211	0.0160	0.0265	0.0206	0.0206	0.0201	0.0210	0.0175	0.0191	0.0201	0.0190	0.0194	0.0217	0.0174	0.0212
**DS60**	0.0155	0.0141	0.0428	0.0193	0.0297	0.1514	0.0353	0.0614	0.0113	0.0134	0.0512	0.0146	0.0142	0.0131	0.0147	0.0714	0.0148	0.0148	0.0472	0.0170	0.1426	0.0192	0.0148	0.0149	0.0230	0.0150	0.0130	0.0148	0.0128	0.0123	0.0291	0.0162	0.0171	0.0142	0.0124	0.0185	0.0129	0.0145	0.0165	0.0134	0.0118	0.0122	0.0126	0.0121	0.0130	0.0140	0.0091	0.0139
**DS2**	0.0174	0.0168	0.0416	0.0196	0.0329	0.1522	0.0329	0.0639	0.0172	0.0129	0.0522	0.0154	0.0165	0.0111	0.0155	0.0743	0.0158	0.0157	0.0491	0.0201	0.1425	0.0202	0.0156	0.0140	0.0248	0.0160	0.0175	0.0156	0.0161	0.0167	0.0338	0.0182	0.0191	0.0176	0.0096	0.0218	0.0175	0.0156	0.0167	0.0154	0.0125	0.0163	0.0161	0.0162	0.0150	0.0162	0.0136	0.0159
**DS61**	0.0358	0.0356	0.0657	0.0397	0.0543	0.1669	0.0578	0.0869	0.0346	0.0345	0.0733	0.0384	0.0350	0.0350	0.0370	0.0930	0.0369	0.0371	0.0680	0.0367	0.1305	0.0402	0.0370	0.0386	0.0432	0.0374	0.0336	0.0371	0.0338	0.0336	0.0373	0.0380	0.0386	0.0368	0.0347	0.0395	0.0333	0.0351	0.0394	0.0345	0.0338	0.0334	0.0335	0.0331	0.0345	0.0348	0.0337	0.0340
**DS62**	0.0191	0.0182	0.0417	0.0227	0.0348	0.1546	0.0355	0.0629	0.0165	0.0147	0.0523	0.0098	0.0175	0.0127	0.0091	0.0721	0.0092	0.0092	0.0497	0.0140	0.1419	0.0223	0.0092	0.0123	0.0263	0.0092	0.0174	0.0092	0.0152	0.0167	0.0325	0.0207	0.0196	0.0171	0.0123	0.0165	0.0172	0.0171	0.0108	0.0172	0.0136	0.0158	0.0167	0.0158	0.0164	0.0176	0.0143	0.0176
**DS63**	0.0246	0.0240	0.0466	0.0263	0.0419	0.1556	0.0426	0.0733	0.0234	0.0218	0.0628	0.0222	0.0253	0.0199	0.0217	0.0832	0.0218	0.0216	0.0576	0.0264	0.1479	0.0253	0.0218	0.0211	0.0328	0.0224	0.0240	0.0218	0.0229	0.0241	0.0272	0.0272	0.0269	0.0249	0.0188	0.0297	0.0238	0.0239	0.0240	0.0239	0.0207	0.0222	0.0232	0.0222	0.0230	0.0251	0.0211	0.0246
**DS64**	0.0090	0.0082	0.0442	0.0132	0.0340	0.1461	0.0378	0.0661	0.0084	0.0130	0.0513	0.0173	0.0078	0.0150	0.0165	0.0662	0.0167	0.0167	0.0410	0.0106	0.1426	0.0140	0.0165	0.0164	0.0170	0.0170	0.0070	0.0166	0.0071	0.0072	0.0249	0.0126	0.0115	0.0076	0.0138	0.0127	0.0071	0.0094	0.0183	0.0081	0.0104	0.0054	0.0063	0.0056	0.0078	0.0081	0.0137	0.0088
**DS65**	0.0186	0.0176	0.0417	0.0226	0.0347	0.1552	0.0354	0.0629	0.0164	0.0148	0.0523	0.0097	0.0169	0.0129	0.0091	0.0721	0.0092	0.0091	0.0502	0.0138	0.1422	0.0220	0.0091	0.0117	0.0263	0.0092	0.0172	0.0091	0.0151	0.0166	0.0325	0.0207	0.0197	0.0171	0.0117	0.0165	0.0172	0.0170	0.0107	0.0170	0.0135	0.0157	0.0165	0.0157	0.0162	0.0171	0.0144	0.0176
**DS66**	0.0116	0.0111	0.0456	0.0174	0.0357	0.1477	0.0384	0.0662	0.0098	0.0154	0.0523	0.0170	0.0103	0.0174	0.0160	0.0680	0.0162	0.0161	0.0438	0.0104	0.1429	0.0156	0.0160	0.0162	0.0193	0.0164	0.0100	0.0160	0.0095	0.0103	0.0270	0.0150	0.0137	0.0098	0.0164	0.0129	0.0100	0.0116	0.0179	0.0101	0.0134	0.0099	0.0103	0.0100	0.0105	0.0102	0.0156	0.0107
**DS67**	0.0092	0.0088	0.0449	0.0146	0.0348	0.1460	0.0373	0.0664	0.0075	0.0130	0.0510	0.0173	0.0079	0.0149	0.0162	0.0671	0.0165	0.0163	0.0411	0.0102	0.1430	0.0138	0.0162	0.0167	0.0176	0.0166	0.0076	0.0163	0.0075	0.0079	0.0257	0.0126	0.0114	0.0077	0.0139	0.0134	0.0073	0.0095	0.0186	0.0084	0.0112	0.0071	0.0079	0.0072	0.0084	0.0078	0.0128	0.0086
**DS68**	0.0101	0.0099	0.0459	0.0136	0.0359	0.1465	0.0372	0.0673	0.0091	0.0128	0.0506	0.0176	0.0094	0.0147	0.0163	0.0678	0.0169	0.0167	0.0424	0.0113	0.1437	0.0146	0.0166	0.0171	0.0185	0.0170	0.0088	0.0168	0.0087	0.0086	0.0265	0.0131	0.0120	0.0094	0.0138	0.0128	0.0087	0.0109	0.0182	0.0093	0.0110	0.0075	0.0077	0.0077	0.0092	0.0092	0.0131	0.0087
**DS69**	0.0156	0.0152	0.0397	0.0201	0.0326	0.1510	0.0320	0.0636	0.0133	0.0114	0.0531	0.0117	0.0145	0.0102	0.0113	0.0726	0.0115	0.0114	0.0472	0.0164	0.1419	0.0191	0.0113	0.0123	0.0231	0.0119	0.0135	0.0113	0.0129	0.0139	0.0312	0.0174	0.0172	0.0143	0.0088	0.0194	0.0134	0.0151	0.0132	0.0141	0.0110	0.0123	0.0127	0.0125	0.0140	0.0143	0.0113	0.0142
**DS44**	0.0188	0.0176	0.0419	0.0228	0.0349	0.1553	0.0355	0.0630	0.0166	0.0150	0.0524	0.0100	0.0170	0.0129	0.0092	0.0721	0.0096	0.0094	0.0502	0.0141	0.1422	0.0221	0.0094	0.0120	0.0265	0.0094	0.0174	0.0094	0.0152	0.0166	0.0328	0.0208	0.0197	0.0174	0.0119	0.0167	0.0174	0.0172	0.0108	0.0172	0.0136	0.0158	0.0166	0.0158	0.0164	0.0173	0.0145	0.0176
**FC2**	0.0141	0.0144	0.0390	0.0197	0.0316	0.1504	0.0320	0.0637	0.0099	0.0103	0.0515	0.0122	0.0129	0.0091	0.0110	0.0709	0.0113	0.0113	0.0458	0.0155	0.1430	0.0189	0.0111	0.0108	0.0229	0.0114	0.0128	0.0111	0.0126	0.0126	0.0301	0.0183	0.0180	0.0138	0.0083	0.0184	0.0128	0.0149	0.0137	0.0133	0.0110	0.0124	0.0131	0.0123	0.0135	0.0136	0.0100	0.0131
**FC18**	0.0345	0.0351	0.0444	0.0393	0.0618	0.1720	0.0628	0.0432	0.0327	0.0380	0.0322	0.0381	0.0346	0.0406	0.0383	0.0481	0.0386	0.0385	0.0663	0.0329	0.1178	0.0360	0.0384	0.0400	0.0434	0.0389	0.0334	0.0384	0.0321	0.0331	0.0279	0.0330	0.0281	0.0322	0.0387	0.0357	0.0332	0.0342	0.0400	0.0330	0.0361	0.0323	0.0326	0.0324	0.0337	0.0340	0.0384	0.0344
**DS71**	0.0184	0.0167	0.0411	0.0195	0.0354	0.1561	0.0376	0.0671	0.0180	0.0162	0.0561	0.0162	0.0185	0.0134	0.0165	0.0769	0.0168	0.0166	0.0516	0.0209	0.1405	0.0194	0.0166	0.0148	0.0268	0.0172	0.0183	0.0166	0.0172	0.0184	0.0345	0.0199	0.0209	0.0190	0.0118	0.0245	0.0182	0.0172	0.0182	0.0182	0.0141	0.0165	0.0178	0.0166	0.0163	0.0187	0.0159	0.0186
**DS72**	0.0154	0.0147	0.0393	0.0198	0.0307	0.1512	0.0320	0.0630	0.0143	0.0114	0.0523	0.0136	0.0136	0.0100	0.0127	0.0704	0.0129	0.0128	0.0457	0.0171	0.1418	0.0178	0.0128	0.0118	0.0230	0.0131	0.0147	0.0127	0.0137	0.0141	0.0308	0.0173	0.0171	0.0155	0.0090	0.0202	0.0146	0.0148	0.0149	0.0146	0.0115	0.0130	0.0142	0.0131	0.0139	0.0143	0.0120	0.0145
**DS73**	0.0192	0.0184	0.0429	0.0240	0.0350	0.1513	0.0353	0.0670	0.0170	0.0149	0.0563	0.0167	0.0176	0.0146	0.0162	0.0738	0.0164	0.0162	0.0486	0.0208	0.1457	0.0216	0.0162	0.0158	0.0269	0.0166	0.0181	0.0161	0.0170	0.0176	0.0272	0.0206	0.0203	0.0186	0.0133	0.0238	0.0181	0.0187	0.0179	0.0179	0.0152	0.0163	0.0173	0.0163	0.0180	0.0176	0.0153	0.0181
**DS74**	0.0226	0.0220	0.0467	0.0263	0.0391	0.1517	0.0394	0.0705	0.0205	0.0186	0.0597	0.0193	0.0215	0.0167	0.0184	0.0797	0.0186	0.0186	0.0547	0.0231	0.1499	0.0258	0.0186	0.0192	0.0299	0.0189	0.0207	0.0184	0.0196	0.0209	0.0247	0.0237	0.0227	0.0215	0.0151	0.0263	0.0206	0.0211	0.0201	0.0213	0.0172	0.0192	0.0197	0.0194	0.0198	0.0214	0.0187	0.0213
**DS75**	0.0149	0.0154	0.0394	0.0192	0.0329	0.1517	0.0310	0.0640	0.0142	0.0114	0.0535	0.0110	0.0152	0.0097	0.0092	0.0731	0.0093	0.0092	0.0466	0.0164	0.1432	0.0200	0.0092	0.0119	0.0223	0.0104	0.0119	0.0092	0.0142	0.0143	0.0318	0.0188	0.0184	0.0151	0.0084	0.0183	0.0119	0.0144	0.0123	0.0136	0.0117	0.0135	0.0140	0.0137	0.0153	0.0151	0.0120	0.0149
**DS76**	0.0226	0.0221	0.0474	0.0264	0.0393	0.1521	0.0401	0.0710	0.0196	0.0191	0.0598	0.0200	0.0214	0.0174	0.0185	0.0794	0.0189	0.0189	0.0543	0.0232	0.1501	0.0264	0.0188	0.0195	0.0300	0.0196	0.0211	0.0188	0.0195	0.0205	0.0242	0.0231	0.0229	0.0214	0.0159	0.0270	0.0210	0.0208	0.0203	0.0211	0.0177	0.0193	0.0205	0.0194	0.0197	0.0213	0.0180	0.0215
**CL13**	0.0174	0.0174	0.0322	0.0186	0.0361	0.1542	0.0358	0.0650	0.0173	0.0134	0.0561	0.0164	0.0179	0.0144	0.0158	0.0765	0.0162	0.0160	0.0470	0.0200	0.1386	0.0184	0.0160	0.0133	0.0274	0.0168	0.0182	0.0159	0.0170	0.0168	0.0353	0.0184	0.0201	0.0190	0.0133	0.0236	0.0182	0.0191	0.0182	0.0183	0.0162	0.0174	0.0176	0.0173	0.0186	0.0182	0.0150	0.0180
**CL14**	0.0312	0.0322	0.0549	0.0358	0.0538	0.1774	0.0545	0.0699	0.0311	0.0320	0.0568	0.0315	0.0323	0.0324	0.0310	0.0752	0.0311	0.0308	0.0659	0.0317	0.1340	0.0362	0.0312	0.0327	0.0401	0.0312	0.0306	0.0309	0.0312	0.0316	0.0412	0.0341	0.0355	0.0307	0.0319	0.0321	0.0303	0.0321	0.0322	0.0297	0.0316	0.0311	0.0308	0.0306	0.0317	0.0318	0.0322	0.0322
**CL15**	0.0396	0.0393	0.0644	0.0384	0.0581	0.1659	0.0617	0.0869	0.0401	0.0397	0.0738	0.0351	0.0406	0.0385	0.0357	0.0933	0.0359	0.0357	0.0707	0.0382	0.1590	0.0415	0.0358	0.0364	0.0490	0.0358	0.0399	0.0358	0.0397	0.0397	0.0466	0.0431	0.0441	0.0418	0.0371	0.0385	0.0399	0.0392	0.0373	0.0397	0.0377	0.0394	0.0398	0.0394	0.0392	0.0401	0.0375	0.0398
**CL16**	0.0253	0.0247	0.0531	0.0277	0.0500	0.1557	0.0522	0.0753	0.0258	0.0304	0.0646	0.0287	0.0256	0.0324	0.0294	0.0807	0.0294	0.0292	0.0579	0.0237	0.1451	0.0218	0.0293	0.0290	0.0341	0.0297	0.0263	0.0294	0.0253	0.0254	0.0407	0.0258	0.0266	0.0273	0.0308	0.0266	0.0261	0.0267	0.0306	0.0266	0.0282	0.0251	0.0253	0.0252	0.0256	0.0257	0.0308	0.0264
**FC19**	0.0395	0.0407	0.0388	0.0443	0.0588	0.1769	0.0571	0.0400	0.0385	0.0362	0.0337	0.0329	0.0394	0.0347	0.0327	0.0532	0.0330	0.0329	0.0711	0.0380	0.1179	0.0417	0.0329	0.0343	0.0486	0.0331	0.0388	0.0328	0.0378	0.0383	0.0331	0.0387	0.0344	0.0374	0.0329	0.0410	0.0387	0.0393	0.0341	0.0379	0.0361	0.0372	0.0377	0.0374	0.0382	0.0390	0.0358	0.0393
**HL33**	0.0471	0.0473	0.0815	0.0468	0.0705	0.1144	0.0730	0.1029	0.0451	0.0485	0.0581	0.0560	0.0470	0.0490	0.0544	0.0453	0.0548	0.0546	0.0238	0.0481	0.1191	0.0521	0.0546	0.0550	0.0555	0.0549	0.0462	0.0544	0.0461	0.0459	0.0631	0.0491	0.0412	0.0434	0.0498	0.0476	0.0462	0.0467	0.0571	0.0466	0.0476	0.0459	0.0458	0.0462	0.0454	0.0464	0.0500	0.0439
**CL17**	0.0350	0.0340	0.0516	0.0369	0.0488	0.1590	0.0492	0.0752	0.0346	0.0318	0.0689	0.0278	0.0341	0.0302	0.0277	0.0897	0.0281	0.0277	0.0669	0.0325	0.1519	0.0341	0.0280	0.0284	0.0423	0.0282	0.0350	0.0278	0.0345	0.0338	0.0411	0.0350	0.0373	0.0369	0.0288	0.0351	0.0348	0.0344	0.0299	0.0352	0.0312	0.0342	0.0347	0.0341	0.0341	0.0344	0.0321	0.0350
**CL18**	0.0255	0.0246	0.0471	0.0290	0.0405	0.1588	0.0421	0.0684	0.0252	0.0243	0.0608	0.0204	0.0245	0.0245	0.0207	0.0796	0.0209	0.0209	0.0582	0.0233	0.1448	0.0263	0.0207	0.0215	0.0328	0.0210	0.0253	0.0208	0.0245	0.0250	0.0410	0.0276	0.0298	0.0268	0.0225	0.0261	0.0253	0.0260	0.0224	0.0252	0.0235	0.0242	0.0247	0.0242	0.0253	0.0245	0.0228	0.0261
**CL19**	0.1382	0.1396	0.1205	0.1425	0.1278	0.2245	0.1359	0.1142	0.1389	0.1420	0.1355	0.1441	0.1385	0.1453	0.1443	0.1531	0.1448	0.1440	0.1758	0.1385	0.1875	0.1416	0.1441	0.1445	0.1281	0.1445	0.1390	0.1444	0.1387	0.1397	0.1329	0.1416	0.1579	0.1519	0.1446	0.1409	0.1389	0.1402	0.1469	0.1387	0.1412	0.1389	0.1385	0.1387	0.1387	0.1382	0.1435	0.1392
**CL20**	0.0365	0.0370	0.0302	0.0401	0.0161	0.1432	0.0246	0.0455	0.0365	0.0317	0.0695	0.0294	0.0356	0.0299	0.0271	0.0913	0.0274	0.0272	0.0674	0.0330	0.1240	0.0425	0.0270	0.0299	0.0256	0.0279	0.0350	0.0271	0.0369	0.0366	0.0524	0.0397	0.0426	0.0398	0.0300	0.0356	0.0351	0.0356	0.0303	0.0358	0.0331	0.0366	0.0378	0.0367	0.0363	0.0364	0.0348	0.0370
**CL21**	0.0105	0.0116	0.0453	0.0150	0.0336	0.1453	0.0361	0.0672	0.0099	0.0096	0.0504	0.0203	0.0100	0.0152	0.0186	0.0667	0.0189	0.0188	0.0424	0.0120	0.1424	0.0156	0.0187	0.0188	0.0184	0.0192	0.0102	0.0189	0.0104	0.0101	0.0271	0.0152	0.0132	0.0101	0.0156	0.0143	0.0101	0.0127	0.0212	0.0105	0.0133	0.0104	0.0103	0.0106	0.0113	0.0101	0.0141	0.0084
**CL22**	0.0248	0.0242	0.0481	0.0286	0.0392	0.1612	0.0416	0.0721	0.0234	0.0193	0.0596	0.0232	0.0237	0.0188	0.0219	0.0799	0.0222	0.0221	0.0557	0.0268	0.1460	0.0288	0.0220	0.0210	0.0323	0.0226	0.0237	0.0219	0.0236	0.0225	0.0399	0.0272	0.0247	0.0237	0.0178	0.0292	0.0237	0.0245	0.0233	0.0242	0.0206	0.0230	0.0235	0.0231	0.0237	0.0242	0.0222	0.0243
**CL23**	0.0374	0.0381	0.0377	0.0395	0.0571	0.1798	0.0574	0.0440	0.0391	0.0358	0.0331	0.0331	0.0394	0.0338	0.0320	0.0513	0.0322	0.0320	0.0721	0.0384	0.1186	0.0427	0.0320	0.0315	0.0476	0.0327	0.0376	0.0318	0.0394	0.0380	0.0335	0.0448	0.0405	0.0390	0.0336	0.0399	0.0377	0.0396	0.0346	0.0383	0.0370	0.0385	0.0394	0.0387	0.0402	0.0400	0.0376	0.0402
**CL24**	0.0331	0.0340	0.0300	0.0382	0.0104	0.1387	0.0240	0.0434	0.0341	0.0305	0.0713	0.0344	0.0331	0.0314	0.0323	0.0888	0.0326	0.0324	0.0640	0.0364	0.1251	0.0391	0.0323	0.0332	0.0213	0.0330	0.0312	0.0323	0.0335	0.0328	0.0499	0.0382	0.0421	0.0369	0.0308	0.0380	0.0313	0.0338	0.0353	0.0325	0.0320	0.0326	0.0334	0.0325	0.0343	0.0329	0.0286	0.0345
**CL25**	0.0170	0.0168	0.0324	0.0182	0.0363	0.1530	0.0364	0.0646	0.0175	0.0142	0.0557	0.0165	0.0174	0.0140	0.0153	0.0758	0.0156	0.0154	0.0470	0.0192	0.1380	0.0176	0.0153	0.0128	0.0274	0.0164	0.0179	0.0154	0.0169	0.0165	0.0352	0.0182	0.0203	0.0190	0.0128	0.0230	0.0179	0.0191	0.0178	0.0183	0.0160	0.0168	0.0172	0.0168	0.0183	0.0181	0.0151	0.0176
**FC20**	0.0092	0.0099	0.0454	0.0124	0.0360	0.1462	0.0356	0.0671	0.0100	0.0123	0.0505	0.0176	0.0093	0.0143	0.0158	0.0680	0.0163	0.0160	0.0418	0.0123	0.1439	0.0156	0.0159	0.0172	0.0176	0.0165	0.0073	0.0161	0.0097	0.0084	0.0268	0.0143	0.0129	0.0095	0.0149	0.0109	0.0073	0.0102	0.0183	0.0082	0.0121	0.0094	0.0089	0.0095	0.0102	0.0090	0.0128	0.0085
**HL34**	0.0146	0.0144	0.0424	0.0178	0.0272	0.1513	0.0344	0.0589	0.0151	0.0128	0.0494	0.0152	0.0149	0.0106	0.0133	0.0715	0.0135	0.0134	0.0464	0.0178	0.1418	0.0212	0.0134	0.0149	0.0215	0.0138	0.0127	0.0133	0.0146	0.0139	0.0300	0.0180	0.0180	0.0153	0.0101	0.0190	0.0128	0.0135	0.0157	0.0132	0.0117	0.0141	0.0146	0.0140	0.0139	0.0147	0.0092	0.0151
**HL35**	0.0175	0.0166	0.0370	0.0196	0.0321	0.1511	0.0348	0.0597	0.0158	0.0127	0.0523	0.0150	0.0164	0.0097	0.0138	0.0741	0.0143	0.0141	0.0477	0.0182	0.1395	0.0196	0.0139	0.0141	0.0251	0.0145	0.0158	0.0140	0.0160	0.0146	0.0326	0.0151	0.0162	0.0174	0.0087	0.0207	0.0158	0.0160	0.0165	0.0172	0.0122	0.0153	0.0155	0.0152	0.0158	0.0169	0.0137	0.0156
**HL36**	0.0149	0.0160	0.0393	0.0153	0.0309	0.1545	0.0352	0.0641	0.0171	0.0133	0.0521	0.0162	0.0170	0.0133	0.0151	0.0748	0.0155	0.0153	0.0486	0.0191	0.1419	0.0192	0.0152	0.0145	0.0249	0.0160	0.0148	0.0153	0.0172	0.0154	0.0340	0.0222	0.0225	0.0185	0.0143	0.0190	0.0149	0.0175	0.0178	0.0158	0.0157	0.0166	0.0165	0.0166	0.0182	0.0175	0.0102	0.0156
**CL26**	0.0140	0.0152	0.0386	0.0184	0.0299	0.1511	0.0320	0.0626	0.0136	0.0093	0.0506	0.0142	0.0140	0.0104	0.0121	0.0714	0.0127	0.0126	0.0443	0.0164	0.1423	0.0202	0.0124	0.0125	0.0219	0.0133	0.0126	0.0124	0.0140	0.0137	0.0309	0.0191	0.0186	0.0148	0.0111	0.0180	0.0126	0.0146	0.0149	0.0132	0.0127	0.0138	0.0143	0.0138	0.0150	0.0143	0.0099	0.0140
**CL27**	0.0166	0.0177	0.0407	0.0207	0.0352	0.1561	0.0341	0.0628	0.0171	0.0138	0.0511	0.0084	0.0175	0.0122	0.0035	0.0700	0.0039	0.0036	0.0483	0.0117	0.1436	0.0226	0.0034	0.0112	0.0244	0.0056	0.0141	0.0036	0.0166	0.0159	0.0316	0.0214	0.0202	0.0162	0.0103	0.0149	0.0139	0.0175	0.0078	0.0159	0.0137	0.0160	0.0162	0.0159	0.0180	0.0171	0.0146	0.0175
**CL28**	0.0176	0.0184	0.0410	0.0213	0.0355	0.1549	0.0339	0.0631	0.0178	0.0140	0.0520	0.0079	0.0180	0.0122	0.0029	0.0722	0.0031	0.0029	0.0491	0.0119	0.1431	0.0230	0.0029	0.0112	0.0252	0.0051	0.0142	0.0028	0.0172	0.0163	0.0329	0.0225	0.0214	0.0178	0.0100	0.0151	0.0142	0.0179	0.0070	0.0165	0.0140	0.0166	0.0167	0.0166	0.0186	0.0177	0.0144	0.0178
**CL29**	0.0173	0.0171	0.0321	0.0183	0.0365	0.1537	0.0362	0.0647	0.0174	0.0141	0.0564	0.0162	0.0176	0.0142	0.0154	0.0764	0.0158	0.0155	0.0468	0.0197	0.1388	0.0180	0.0154	0.0130	0.0278	0.0163	0.0182	0.0155	0.0170	0.0164	0.0356	0.0183	0.0203	0.0191	0.0130	0.0233	0.0182	0.0191	0.0181	0.0184	0.0161	0.0172	0.0175	0.0172	0.0184	0.0181	0.0150	0.0178
**CL30**	0.0147	0.0149	0.0420	0.0182	0.0291	0.1506	0.0338	0.0601	0.0149	0.0114	0.0500	0.0155	0.0144	0.0114	0.0137	0.0718	0.0141	0.0139	0.0473	0.0164	0.1428	0.0199	0.0140	0.0150	0.0230	0.0148	0.0133	0.0138	0.0136	0.0130	0.0308	0.0185	0.0181	0.0145	0.0112	0.0176	0.0133	0.0154	0.0165	0.0146	0.0126	0.0134	0.0128	0.0133	0.0150	0.0137	0.0074	0.0131
**CL31**	0.0141	0.0132	0.0457	0.0170	0.0378	0.1431	0.0418	0.0635	0.0131	0.0175	0.0536	0.0223	0.0138	0.0168	0.0207	0.0701	0.0208	0.0208	0.0450	0.0156	0.1382	0.0169	0.0206	0.0216	0.0214	0.0210	0.0120	0.0207	0.0129	0.0126	0.0294	0.0121	0.0106	0.0123	0.0155	0.0180	0.0119	0.0134	0.0232	0.0136	0.0138	0.0122	0.0125	0.0122	0.0122	0.0133	0.0177	0.0133
**CL32**	0.0145	0.0150	0.0442	0.0191	0.0390	0.1489	0.0430	0.0636	0.0138	0.0172	0.0522	0.0170	0.0139	0.0195	0.0151	0.0675	0.0153	0.0153	0.0453	0.0094	0.1395	0.0173	0.0150	0.0183	0.0232	0.0161	0.0130	0.0152	0.0133	0.0122	0.0296	0.0148	0.0129	0.0139	0.0185	0.0125	0.0129	0.0157	0.0179	0.0149	0.0164	0.0129	0.0130	0.0130	0.0149	0.0142	0.0192	0.0139
**FC21**	0.0087	0.0095	0.0455	0.0131	0.0358	0.1462	0.0362	0.0663	0.0080	0.0118	0.0500	0.0186	0.0084	0.0138	0.0172	0.0673	0.0176	0.0175	0.0422	0.0111	0.1429	0.0145	0.0173	0.0172	0.0183	0.0172	0.0083	0.0175	0.0088	0.0088	0.0261	0.0129	0.0116	0.0089	0.0138	0.0125	0.0083	0.0109	0.0197	0.0079	0.0117	0.0088	0.0086	0.0089	0.0088	0.0081	0.0111	0.0075
**HL38**	0.0189	0.0177	0.0371	0.0195	0.0367	0.1538	0.0381	0.0628	0.0191	0.0172	0.0564	0.0177	0.0199	0.0131	0.0169	0.0781	0.0172	0.0171	0.0510	0.0219	0.1356	0.0196	0.0170	0.0153	0.0275	0.0178	0.0194	0.0170	0.0189	0.0191	0.0351	0.0167	0.0191	0.0208	0.0121	0.0250	0.0193	0.0188	0.0193	0.0194	0.0147	0.0180	0.0186	0.0180	0.0173	0.0193	0.0166	0.0196

**CL33**	0.0151	0.0150	0.0442	0.0191	0.0390	0.1486	0.0423	0.0637	0.0133	0.0175	0.0521	0.0164	0.0141	0.0192	0.0150	0.0678	0.0153	0.0151	0.0458	0.0100	0.1403	0.0172	0.0150	0.0186	0.0236	0.0159	0.0129	0.0151	0.0139	0.0124	0.0296	0.0147	0.0132	0.0139	0.0185	0.0125	0.0129	0.0159	0.0177	0.0148	0.0163	0.0134	0.0135	0.0136	0.0150	0.0146	0.0190	0.0137
**HL39**	0.0130	0.0121	0.0436	0.0154	0.0357	0.1445	0.0412	0.0630	0.0123	0.0159	0.0519	0.0217	0.0122	0.0157	0.0196	0.0700	0.0199	0.0198	0.0436	0.0136	0.1383	0.0159	0.0195	0.0202	0.0203	0.0206	0.0110	0.0196	0.0121	0.0110	0.0282	0.0109	0.0106	0.0120	0.0156	0.0166	0.0110	0.0121	0.0226	0.0129	0.0128	0.0112	0.0116	0.0113	0.0109	0.0126	0.0175	0.0118
**CL34**	0.0117	0.0141	0.0457	0.0159	0.0347	0.1465	0.0376	0.0698	0.0120	0.0109	0.0490	0.0214	0.0125	0.0166	0.0195	0.0685	0.0197	0.0196	0.0406	0.0139	0.1422	0.0183	0.0196	0.0202	0.0199	0.0208	0.0106	0.0196	0.0128	0.0126	0.0298	0.0175	0.0147	0.0114	0.0174	0.0152	0.0107	0.0134	0.0223	0.0117	0.0162	0.0129	0.0128	0.0130	0.0139	0.0129	0.0170	0.0116
**CL35**	0.0150	0.0148	0.0394	0.0189	0.0300	0.1520	0.0335	0.0632	0.0146	0.0102	0.0495	0.0138	0.0148	0.0089	0.0124	0.0718	0.0127	0.0125	0.0479	0.0172	0.1416	0.0204	0.0125	0.0123	0.0236	0.0131	0.0152	0.0125	0.0151	0.0138	0.0309	0.0183	0.0183	0.0152	0.0057	0.0203	0.0152	0.0151	0.0152	0.0145	0.0113	0.0146	0.0150	0.0144	0.0139	0.0156	0.0132	0.0154
**CL36**	0.1700	0.1719	0.1604	0.1716	0.1664	0.2083	0.1689	0.1571	0.1696	0.1697	0.1456	0.1711	0.1703	0.1715	0.1715	0.1564	0.1714	0.1711	0.1730	0.1703	0.1250	0.1721	0.1710	0.1711	0.1680	0.1713	0.1711	0.1712	0.1704	0.1703	0.1642	0.1712	0.1638	0.1692	0.1699	0.1700	0.1710	0.1712	0.1734	0.1703	0.1702	0.1701	0.1708	0.1703	0.1707	0.1701	0.1707	0.1695
**CL37**	0.0545	0.0550	0.0762	0.0544	0.0711	0.1876	0.0679	0.0987	0.0525	0.0497	0.0876	0.0459	0.0533	0.0492	0.0454	0.1064	0.0457	0.0454	0.0837	0.0505	0.1758	0.0580	0.0455	0.0478	0.0621	0.0455	0.0538	0.0454	0.0529	0.0533	0.0699	0.0560	0.0566	0.0580	0.0497	0.0473	0.0536	0.0542	0.0457	0.0534	0.0516	0.0531	0.0534	0.0530	0.0544	0.0533	0.0503	0.0514
**CL38**	0.0125	0.0125	0.0425	0.0165	0.0379	0.1462	0.0404	0.0651	0.0109	0.0152	0.0532	0.0195	0.0118	0.0181	0.0195	0.0696	0.0197	0.0196	0.0424	0.0134	0.1410	0.0134	0.0195	0.0193	0.0201	0.0199	0.0112	0.0197	0.0092	0.0108	0.0276	0.0101	0.0101	0.0102	0.0159	0.0167	0.0111	0.0110	0.0209	0.0106	0.0133	0.0089	0.0095	0.0091	0.0106	0.0112	0.0159	0.0114
**CL39**	0.0926	0.0933	0.0907	0.0956	0.1130	0.2324	0.1122	0.0941	0.0928	0.0901	0.0852	0.0874	0.0934	0.0888	0.0867	0.1069	0.0873	0.0868	0.1251	0.0923	0.1671	0.0941	0.0867	0.0876	0.1029	0.0869	0.0926	0.0867	0.0930	0.0924	0.0877	0.0933	0.0907	0.0940	0.0878	0.0939	0.0926	0.0945	0.0891	0.0931	0.0906	0.0925	0.0924	0.0921	0.0933	0.0936	0.0900	0.0933
**CL40**	0.0394	0.0392	0.0442	0.0411	0.0315	0.1523	0.0364	0.0619	0.0404	0.0381	0.0746	0.0369	0.0404	0.0357	0.0356	0.0971	0.0358	0.0354	0.0731	0.0399	0.1356	0.0420	0.0353	0.0351	0.0365	0.0363	0.0401	0.0356	0.0404	0.0405	0.0559	0.0431	0.0440	0.0417	0.0338	0.0436	0.0403	0.0403	0.0385	0.0406	0.0359	0.0403	0.0405	0.0403	0.0393	0.0400	0.0365	0.0411
**FC22**	0.0092	0.0081	0.0450	0.0154	0.0347	0.1463	0.0386	0.0671	0.0096	0.0137	0.0519	0.0197	0.0059	0.0163	0.0178	0.0671	0.0180	0.0179	0.0423	0.0117	0.1432	0.0148	0.0177	0.0147	0.0191	0.0184	0.0090	0.0178	0.0098	0.0084	0.0270	0.0134	0.0123	0.0099	0.0152	0.0147	0.0091	0.0121	0.0199	0.0106	0.0127	0.0095	0.0095	0.0097	0.0103	0.0060	0.0153	0.0098
**CL41**	0.3020	0.3049	0.2960	0.3047	0.2973	0.2460	0.3003	0.2919	0.3002	0.3001	0.2821	0.3026	0.3015	0.3005	0.3007	0.2950	0.3000	0.2992	0.3075	0.3004	0.2633	0.3054	0.3001	0.3038	0.2988	0.2996	0.2994	0.2993	0.3003	0.3012	0.2940	0.3009	0.2841	0.3045	0.3010	0.3006	0.2993	0.3007	0.3002	0.3003	0.2998	0.3003	0.3010	0.3007	0.3010	0.3006	0.3024	0.3020
**CL42**	0.0400	0.0399	0.0717	0.0449	0.0633	0.1788	0.0664	0.0934	0.0388	0.0420	0.0808	0.0457	0.0399	0.0435	0.0453	0.0987	0.0455	0.0457	0.0720	0.0424	0.1524	0.0438	0.0456	0.0453	0.0487	0.0460	0.0384	0.0456	0.0385	0.0385	0.0557	0.0415	0.0339	0.0363	0.0428	0.0438	0.0383	0.0402	0.0469	0.0391	0.0390	0.0368	0.0379	0.0370	0.0381	0.0400	0.0422	0.0396
**CL43**	0.0270	0.0268	0.0446	0.0298	0.0497	0.1616	0.0546	0.0600	0.0259	0.0297	0.0473	0.0345	0.0261	0.0297	0.0332	0.0657	0.0333	0.0332	0.0577	0.0262	0.1333	0.0309	0.0331	0.0333	0.0347	0.0335	0.0256	0.0332	0.0259	0.0250	0.0274	0.0266	0.0234	0.0247	0.0302	0.0293	0.0254	0.0259	0.0358	0.0265	0.0271	0.0256	0.0257	0.0258	0.0246	0.0263	0.0312	0.0256
**HL40**	0.0153	0.0157	0.0365	0.0144	0.0349	0.1542	0.0319	0.0663	0.0172	0.0121	0.0544	0.0132	0.0179	0.0107	0.0118	0.0754	0.0123	0.0121	0.0486	0.0196	0.1423	0.0194	0.0120	0.0121	0.0244	0.0128	0.0143	0.0121	0.0169	0.0153	0.0333	0.0215	0.0223	0.0175	0.0110	0.0186	0.0143	0.0170	0.0150	0.0151	0.0137	0.0158	0.0160	0.0158	0.0176	0.0171	0.0122	0.0156
**HL41**	0.0154	0.0152	0.0427	0.0187	0.0304	0.1508	0.0333	0.0610	0.0144	0.0125	0.0499	0.0158	0.0146	0.0119	0.0133	0.0723	0.0136	0.0136	0.0479	0.0169	0.1435	0.0200	0.0132	0.0152	0.0236	0.0136	0.0140	0.0134	0.0142	0.0130	0.0309	0.0190	0.0186	0.0149	0.0117	0.0179	0.0139	0.0163	0.0158	0.0144	0.0129	0.0136	0.0137	0.0136	0.0150	0.0139	0.0071	0.0133
**HL42**	0.0167	0.0171	0.0431	0.0212	0.0324	0.1550	0.0365	0.0600	0.0166	0.0149	0.0506	0.0112	0.0165	0.0147	0.0090	0.0713	0.0093	0.0092	0.0486	0.0130	0.1426	0.0226	0.0091	0.0130	0.0242	0.0096	0.0148	0.0092	0.0160	0.0159	0.0321	0.0216	0.0209	0.0170	0.0141	0.0147	0.0147	0.0163	0.0119	0.0154	0.0156	0.0158	0.0166	0.0158	0.0172	0.0161	0.0111	0.0172
**HL43**	0.0119	0.0116	0.0465	0.0176	0.0377	0.1495	0.0399	0.0656	0.0104	0.0154	0.0502	0.0153	0.0105	0.0182	0.0133	0.0663	0.0137	0.0135	0.0440	0.0077	0.1425	0.0169	0.0135	0.0165	0.0197	0.0137	0.0108	0.0135	0.0104	0.0107	0.0268	0.0158	0.0139	0.0106	0.0170	0.0105	0.0106	0.0128	0.0158	0.0106	0.0139	0.0107	0.0109	0.0110	0.0111	0.0102	0.0158	0.0118
**CL44**	0.0723	0.0743	0.1051	0.0750	0.0982	0.1808	0.0964	0.1242	0.0730	0.0764	0.1131	0.0739	0.0736	0.0785	0.0742	0.1291	0.0742	0.0738	0.1068	0.0701	0.2018	0.0751	0.0738	0.0774	0.0807	0.0738	0.0722	0.0741	0.0727	0.0726	0.0890	0.0755	0.0808	0.0782	0.0789	0.0702	0.0719	0.0728	0.0741	0.0724	0.0760	0.0724	0.0727	0.0727	0.0738	0.0726	0.0770	0.0731
**CL45**	0.0609	0.0615	0.0821	0.0637	0.0779	0.1953	0.0738	0.1044	0.0614	0.0571	0.0959	0.0520	0.0609	0.0560	0.0522	0.1173	0.0523	0.0521	0.0950	0.0582	0.1850	0.0635	0.0521	0.0545	0.0699	0.0522	0.0611	0.0523	0.0612	0.0608	0.0770	0.0639	0.0677	0.0653	0.0554	0.0593	0.0608	0.0626	0.0526	0.0611	0.0591	0.0602	0.0610	0.0605	0.0619	0.0605	0.0573	0.0605
**HL44**	0.0176	0.0184	0.0412	0.0215	0.0356	0.1552	0.0338	0.0632	0.0179	0.0140	0.0521	0.0079	0.0180	0.0120	0.0027	0.0722	0.0031	0.0028	0.0491	0.0120	0.1431	0.0229	0.0026	0.0112	0.0253	0.0050	0.0143	0.0029	0.0173	0.0163	0.0328	0.0225	0.0211	0.0177	0.0099	0.0152	0.0141	0.0179	0.0070	0.0166	0.0140	0.0166	0.0168	0.0167	0.0187	0.0176	0.0143	0.0177
**FC23**	0.0316	0.0320	0.0464	0.0375	0.0585	0.1716	0.0600	0.0442	0.0319	0.0350	0.0290	0.0381	0.0311	0.0378	0.0365	0.0450	0.0369	0.0367	0.0647	0.0307	0.1195	0.0375	0.0366	0.0380	0.0416	0.0363	0.0310	0.0367	0.0315	0.0311	0.0255	0.0364	0.0292	0.0290	0.0368	0.0328	0.0310	0.0339	0.0393	0.0316	0.0346	0.0318	0.0318	0.0320	0.0323	0.0305	0.0356	0.0318
**HL45**	0.0125	0.0114	0.0436	0.0151	0.0359	0.1451	0.0402	0.0627	0.0120	0.0160	0.0516	0.0209	0.0122	0.0156	0.0190	0.0698	0.0194	0.0191	0.0428	0.0138	0.1381	0.0155	0.0191	0.0201	0.0193	0.0199	0.0107	0.0192	0.0113	0.0110	0.0274	0.0097	0.0095	0.0113	0.0149	0.0163	0.0105	0.0118	0.0215	0.0119	0.0117	0.0103	0.0104	0.0105	0.0099	0.0115	0.0163	0.0114
**CL46**	0.0177	0.0185	0.0413	0.0215	0.0358	0.1552	0.0341	0.0634	0.0181	0.0142	0.0522	0.0080	0.0181	0.0123	0.0029	0.0721	0.0033	0.0030	0.0490	0.0121	0.1433	0.0231	0.0030	0.0113	0.0254	0.0052	0.0144	0.0030	0.0176	0.0164	0.0331	0.0225	0.0211	0.0178	0.0101	0.0153	0.0144	0.0182	0.0071	0.0167	0.0140	0.0167	0.0169	0.0167	0.0188	0.0177	0.0145	0.0179
**CL47**	0.0174	0.0177	0.0446	0.0227	0.0286	0.1519	0.0362	0.0613	0.0166	0.0141	0.0536	0.0183	0.0174	0.0150	0.0160	0.0724	0.0161	0.0161	0.0495	0.0194	0.1431	0.0227	0.0160	0.0175	0.0246	0.0169	0.0153	0.0161	0.0163	0.0159	0.0325	0.0221	0.0200	0.0166	0.0133	0.0217	0.0151	0.0184	0.0189	0.0166	0.0155	0.0158	0.0164	0.0159	0.0178	0.0173	0.0112	0.0170
**CL48**	0.0087	0.0095	0.0449	0.0133	0.0350	0.1468	0.0362	0.0663	0.0084	0.0135	0.0516	0.0159	0.0092	0.0147	0.0152	0.0670	0.0155	0.0154	0.0409	0.0119	0.1439	0.0149	0.0153	0.0168	0.0167	0.0143	0.0067	0.0155	0.0087	0.0082	0.0255	0.0142	0.0124	0.0091	0.0140	0.0117	0.0066	0.0094	0.0178	0.0068	0.0112	0.0082	0.0084	0.0082	0.0094	0.0088	0.0125	0.0096
**CL49**	0.0175	0.0178	0.0451	0.0203	0.0285	0.1515	0.0359	0.0607	0.0170	0.0137	0.0508	0.0191	0.0178	0.0114	0.0175	0.0740	0.0179	0.0178	0.0503	0.0199	0.1402	0.0225	0.0176	0.0182	0.0243	0.0182	0.0174	0.0178	0.0169	0.0170	0.0331	0.0198	0.0194	0.0176	0.0125	0.0218	0.0173	0.0174	0.0200	0.0175	0.0142	0.0170	0.0170	0.0169	0.0165	0.0174	0.0102	0.0166
**CL50**	0.0175	0.0184	0.0411	0.0215	0.0356	0.1546	0.0338	0.0629	0.0179	0.0140	0.0519	0.0079	0.0179	0.0120	0.0028	0.0719	0.0032	0.0030	0.0490	0.0120	0.1429	0.0230	0.0028	0.0112	0.0251	0.0051	0.0143	0.0029	0.0173	0.0162	0.0327	0.0224	0.0213	0.0178	0.0099	0.0153	0.0142	0.0179	0.0069	0.0165	0.0138	0.0165	0.0168	0.0166	0.0186	0.0176	0.0144	0.0177
**CL51**	0.0132	0.0128	0.0435	0.0167	0.0383	0.1457	0.0401	0.0647	0.0108	0.0160	0.0534	0.0200	0.0124	0.0174	0.0198	0.0699	0.0200	0.0200	0.0429	0.0142	0.1404	0.0137	0.0198	0.0199	0.0212	0.0201	0.0117	0.0200	0.0093	0.0110	0.0282	0.0097	0.0096	0.0114	0.0165	0.0168	0.0115	0.0115	0.0212	0.0118	0.0136	0.0101	0.0106	0.0102	0.0113	0.0122	0.0162	0.0115
**CL52**	0.0088	0.0092	0.0438	0.0130	0.0327	0.1460	0.0366	0.0663	0.0087	0.0115	0.0502	0.0172	0.0078	0.0139	0.0153	0.0657	0.0155	0.0154	0.0399	0.0108	0.1428	0.0150	0.0152	0.0159	0.0163	0.0160	0.0063	0.0153	0.0086	0.0069	0.0258	0.0141	0.0123	0.0083	0.0142	0.0120	0.0062	0.0092	0.0180	0.0072	0.0113	0.0082	0.0083	0.0083	0.0096	0.0085	0.0146	0.0085
**CL53**	0.0146	0.0156	0.0425	0.0177	0.0304	0.1515	0.0329	0.0610	0.0145	0.0122	0.0507	0.0152	0.0154	0.0108	0.0132	0.0728	0.0137	0.0136	0.0470	0.0175	0.1438	0.0206	0.0134	0.0149	0.0232	0.0134	0.0124	0.0134	0.0150	0.0137	0.0315	0.0194	0.0190	0.0157	0.0116	0.0165	0.0123	0.0155	0.0160	0.0129	0.0136	0.0141	0.0143	0.0141	0.0160	0.0147	0.0066	0.0135
**CL54**	0.0176	0.0171	0.0322	0.0185	0.0366	0.1535	0.0361	0.0646	0.0175	0.0142	0.0563	0.0164	0.0177	0.0142	0.0156	0.0762	0.0158	0.0158	0.0466	0.0199	0.1386	0.0182	0.0156	0.0132	0.0278	0.0166	0.0183	0.0158	0.0171	0.0165	0.0357	0.0183	0.0205	0.0192	0.0134	0.0234	0.0182	0.0192	0.0181	0.0185	0.0161	0.0173	0.0177	0.0173	0.0186	0.0181	0.0151	0.0178
**FC24**	0.0721	0.0748	0.0767	0.0735	0.0855	0.1531	0.0906	0.0730	0.0720	0.0670	0.0299	0.0695	0.0735	0.0694	0.0681	0.0184	0.0682	0.0680	0.0409	0.0715	0.0879	0.0790	0.0679	0.0709	0.0801	0.0689	0.0705	0.0678	0.0725	0.0723	0.0666	0.0775	0.0677	0.0707	0.0691	0.0692	0.0708	0.0735	0.0705	0.0716	0.0713	0.0722	0.0722	0.0724	0.0739	0.0729	0.0665	0.0710
**CL55**	0.0342	0.0343	0.0617	0.0337	0.0537	0.1705	0.0587	0.0868	0.0358	0.0361	0.0738	0.0384	0.0356	0.0346	0.0375	0.0934	0.0377	0.0378	0.0680	0.0382	0.1206	0.0384	0.0376	0.0361	0.0434	0.0383	0.0341	0.0377	0.0356	0.0343	0.0523	0.0391	0.0395	0.0367	0.0342	0.0398	0.0340	0.0341	0.0397	0.0347	0.0346	0.0350	0.0356	0.0350	0.0349	0.0364	0.0359	0.0361
**CL56**	0.0186	0.0171	0.0369	0.0184	0.0365	0.1535	0.0374	0.0630	0.0192	0.0164	0.0557	0.0172	0.0199	0.0132	0.0158	0.0772	0.0162	0.0160	0.0503	0.0218	0.1368	0.0190	0.0160	0.0149	0.0271	0.0167	0.0186	0.0160	0.0186	0.0181	0.0349	0.0167	0.0188	0.0199	0.0116	0.0242	0.0185	0.0183	0.0184	0.0190	0.0142	0.0177	0.0183	0.0178	0.0174	0.0190	0.0160	0.0189
**CL57**	0.0175	0.0172	0.0321	0.0184	0.0364	0.1535	0.0358	0.0644	0.0175	0.0140	0.0563	0.0162	0.0178	0.0140	0.0153	0.0763	0.0157	0.0155	0.0467	0.0198	0.1386	0.0181	0.0155	0.0131	0.0277	0.0164	0.0183	0.0156	0.0171	0.0166	0.0356	0.0184	0.0203	0.0191	0.0131	0.0234	0.0181	0.0192	0.0181	0.0184	0.0161	0.0172	0.0177	0.0173	0.0187	0.0182	0.0150	0.0178
**CL58**	0.0161	0.0164	0.0436	0.0187	0.0291	0.1507	0.0354	0.0604	0.0152	0.0131	0.0496	0.0179	0.0158	0.0081	0.0160	0.0743	0.0164	0.0164	0.0491	0.0174	0.1412	0.0210	0.0162	0.0160	0.0245	0.0169	0.0155	0.0163	0.0158	0.0148	0.0322	0.0184	0.0182	0.0167	0.0113	0.0195	0.0154	0.0158	0.0189	0.0164	0.0132	0.0154	0.0157	0.0155	0.0151	0.0160	0.0089	0.0143
**CL59**	0.0173	0.0170	0.0324	0.0184	0.0363	0.1538	0.0360	0.0646	0.0173	0.0141	0.0562	0.0161	0.0175	0.0139	0.0153	0.0764	0.0157	0.0155	0.0467	0.0197	0.1389	0.0180	0.0154	0.0129	0.0276	0.0164	0.0180	0.0155	0.0169	0.0163	0.0355	0.0182	0.0200	0.0189	0.0130	0.0232	0.0180	0.0190	0.0179	0.0182	0.0159	0.0170	0.0174	0.0171	0.0183	0.0178	0.0149	0.0177
**CL60**	0.0177	0.0173	0.0323	0.0185	0.0366	0.1538	0.0361	0.0647	0.0177	0.0142	0.0564	0.0163	0.0179	0.0142	0.0156	0.0766	0.0159	0.0158	0.0470	0.0201	0.1387	0.0182	0.0156	0.0132	0.0279	0.0166	0.0186	0.0158	0.0172	0.0168	0.0358	0.0186	0.0202	0.0193	0.0133	0.0235	0.0184	0.0194	0.0183	0.0186	0.0163	0.0174	0.0177	0.0174	0.0188	0.0183	0.0151	0.0178
**CL61**	0.0173	0.0173	0.0449	0.0210	0.0279	0.1519	0.0369	0.0605	0.0165	0.0144	0.0518	0.0186	0.0173	0.0128	0.0174	0.0735	0.0176	0.0174	0.0494	0.0196	0.1407	0.0223	0.0174	0.0177	0.0236	0.0178	0.0167	0.0176	0.0162	0.0159	0.0323	0.0194	0.0189	0.0171	0.0121	0.0223	0.0165	0.0168	0.0197	0.0165	0.0136	0.0165	0.0163	0.0165	0.0158	0.0167	0.0112	0.0171
**CL62**	0.0090	0.0066	0.0453	0.0157	0.0347	0.1466	0.0385	0.0674	0.0097	0.0137	0.0521	0.0202	0.0050	0.0164	0.0183	0.0669	0.0185	0.0184	0.0422	0.0121	0.1433	0.0156	0.0183	0.0139	0.0191	0.0189	0.0097	0.0184	0.0100	0.0091	0.0275	0.0136	0.0123	0.0101	0.0156	0.0149	0.0095	0.0122	0.0206	0.0105	0.0131	0.0100	0.0104	0.0102	0.0105	0.0058	0.0160	0.0103
**CL63**	0.0095	0.0101	0.0441	0.0130	0.0350	0.1456	0.0367	0.0661	0.0100	0.0129	0.0508	0.0166	0.0099	0.0147	0.0129	0.0668	0.0131	0.0130	0.0404	0.0101	0.1427	0.0152	0.0129	0.0172	0.0169	0.0149	0.0061	0.0130	0.0093	0.0078	0.0259	0.0142	0.0128	0.0083	0.0132	0.0128	0.0060	0.0099	0.0162	0.0082	0.0108	0.0086	0.0084	0.0088	0.0102	0.0094	0.0143	0.0093
**CL64**	0.0437	0.0454	0.0776	0.0478	0.0646	0.1226	0.0705	0.1002	0.0420	0.0460	0.0509	0.0534	0.0432	0.0501	0.0522	0.0342	0.0524	0.0521	0.0097	0.0462	0.1098	0.0480	0.0521	0.0520	0.0522	0.0529	0.0437	0.0519	0.0436	0.0438	0.0607	0.0486	0.0419	0.0417	0.0486	0.0464	0.0433	0.0455	0.0548	0.0435	0.0473	0.0439	0.0443	0.0443	0.0447	0.0435	0.0496	0.0429
**FC25**	0.0385	0.0385	0.0513	0.0411	0.0313	0.1602	0.0435	0.0652	0.0389	0.0350	0.0734	0.0401	0.0393	0.0339	0.0390	0.0965	0.0396	0.0394	0.0719	0.0418	0.1470	0.0435	0.0394	0.0393	0.0310	0.0397	0.0383	0.0393	0.0388	0.0379	0.0543	0.0414	0.0406	0.0393	0.0340	0.0428	0.0382	0.0391	0.0417	0.0387	0.0351	0.0383	0.0380	0.0383	0.0378	0.0388	0.0318	0.0377
**CL65**	0.0141	0.0149	0.0415	0.0182	0.0289	0.1513	0.0338	0.0615	0.0130	0.0118	0.0511	0.0146	0.0132	0.0117	0.0127	0.0719	0.0132	0.0130	0.0457	0.0156	0.1427	0.0197	0.0129	0.0135	0.0221	0.0138	0.0115	0.0130	0.0134	0.0125	0.0310	0.0182	0.0180	0.0148	0.0118	0.0175	0.0114	0.0137	0.0155	0.0125	0.0129	0.0129	0.0140	0.0130	0.0143	0.0139	0.0083	0.0137
**HL46**	0.0132	0.0130	0.0437	0.0178	0.0383	0.1462	0.0397	0.0648	0.0117	0.0164	0.0544	0.0211	0.0120	0.0185	0.0203	0.0683	0.0206	0.0205	0.0416	0.0149	0.1402	0.0118	0.0204	0.0195	0.0216	0.0206	0.0120	0.0206	0.0105	0.0115	0.0289	0.0101	0.0101	0.0120	0.0177	0.0174	0.0117	0.0129	0.0217	0.0122	0.0149	0.0106	0.0112	0.0108	0.0122	0.0118	0.0168	0.0122
**BL10**	0.0737	0.0763	0.0903	0.0787	0.0677	0.2247	0.0843	0.1110	0.0750	0.0722	0.1180	0.0769	0.0752	0.0724	0.0765	0.1420	0.0769	0.0768	0.1147	0.0791	0.1869	0.0793	0.0761	0.0744	0.0711	0.0775	0.0764	0.0769	0.0759	0.0743	0.0948	0.0804	0.0779	0.0750	0.0712	0.0819	0.0756	0.0772	0.0790	0.0755	0.0731	0.0747	0.0750	0.0753	0.0757	0.0764	0.0698	0.0762
**BL11**	0.1015	0.1019	0.1070	0.1065	0.0943	0.2265	0.1042	0.1180	0.1021	0.1012	0.1320	0.1029	0.1024	0.1015	0.1023	0.1543	0.1022	0.1024	0.1344	0.1042	0.1837	0.1051	0.1024	0.1029	0.0938	0.1026	0.1013	0.1022	0.1019	0.1023	0.1158	0.1031	0.1003	0.1037	0.1000	0.1062	0.1014	0.1024	0.1046	0.1016	0.0996	0.1017	0.1019	0.1012	0.1020	0.1019	0.0997	0.1038
**BL12**	0.0581	0.0587	0.0796	0.0630	0.0695	0.1954	0.0770	0.0963	0.0584	0.0568	0.0896	0.0611	0.0591	0.0568	0.0594	0.1117	0.0596	0.0596	0.0902	0.0612	0.1735	0.0630	0.0594	0.0595	0.0634	0.0603	0.0582	0.0595	0.0584	0.0587	0.0716	0.0600	0.0555	0.0576	0.0560	0.0634	0.0581	0.0592	0.0621	0.0588	0.0562	0.0583	0.0584	0.0581	0.0582	0.0590	0.0560	0.0599
**BL13**	0.0924	0.0949	0.1191	0.0932	0.1093	0.1798	0.1099	0.1441	0.0913	0.0881	0.0999	0.0904	0.0946	0.0847	0.0893	0.0886	0.0892	0.0890	0.0639	0.0960	0.1446	0.0985	0.0890	0.0909	0.1011	0.0894	0.0911	0.0887	0.0926	0.0923	0.1092	0.0953	0.0778	0.0872	0.0857	0.0942	0.0911	0.0913	0.0910	0.0918	0.0881	0.0920	0.0925	0.0920	0.0921	0.0942	0.0903	0.0924
**BL14**	0.0804	0.0811	0.0776	0.0787	0.0982	0.1474	0.0977	0.0794	0.0809	0.0779	0.0477	0.0764	0.0827	0.0748	0.0745	0.0394	0.0746	0.0743	0.0590	0.0804	0.0903	0.0821	0.0745	0.0752	0.0904	0.0751	0.0810	0.0741	0.0814	0.0809	0.0755	0.0805	0.0699	0.0771	0.0730	0.0805	0.0809	0.0814	0.0774	0.0818	0.0769	0.0804	0.0813	0.0808	0.0807	0.0819	0.0787	0.0808
**HL47**	0.0787	0.0800	0.0720	0.0791	0.0677	0.1066	0.0585	0.0942	0.0799	0.0760	0.0921	0.0785	0.0802	0.0756	0.0769	0.0802	0.0770	0.0767	0.0563	0.0826	0.1044	0.0798	0.0768	0.0752	0.0740	0.0776	0.0799	0.0767	0.0800	0.0796	0.0972	0.0800	0.0785	0.0798	0.0741	0.0834	0.0798	0.0819	0.0801	0.0809	0.0772	0.0792	0.0801	0.0794	0.0807	0.0799	0.0778	0.0793
**CL66**	0.0153	0.0146	0.0423	0.0179	0.0278	0.1520	0.0357	0.0579	0.0139	0.0139	0.0500	0.0158	0.0151	0.0112	0.0148	0.0724	0.0151	0.0149	0.0476	0.0178	0.1416	0.0197	0.0149	0.0154	0.0226	0.0152	0.0136	0.0150	0.0144	0.0133	0.0291	0.0172	0.0172	0.0151	0.0100	0.0195	0.0135	0.0144	0.0170	0.0146	0.0108	0.0134	0.0140	0.0135	0.0134	0.0147	0.0089	0.0149
**CL67**	0.0170	0.0170	0.0357	0.0186	0.0345	0.1535	0.0373	0.0614	0.0183	0.0154	0.0544	0.0178	0.0182	0.0118	0.0159	0.0754	0.0162	0.0161	0.0494	0.0200	0.1359	0.0189	0.0158	0.0142	0.0267	0.0171	0.0183	0.0159	0.0185	0.0172	0.0342	0.0171	0.0182	0.0188	0.0114	0.0236	0.0182	0.0182	0.0190	0.0190	0.0144	0.0179	0.0184	0.0180	0.0169	0.0183	0.0157	0.0182
**FC26**	0.0144	0.0147	0.0364	0.0157	0.0344	0.1540	0.0331	0.0661	0.0141	0.0117	0.0528	0.0139	0.0159	0.0100	0.0132	0.0744	0.0136	0.0135	0.0490	0.0181	0.1417	0.0177	0.0134	0.0112	0.0248	0.0138	0.0153	0.0134	0.0155	0.0145	0.0328	0.0206	0.0222	0.0170	0.0102	0.0192	0.0154	0.0175	0.0157	0.0159	0.0134	0.0151	0.0150	0.0151	0.0166	0.0162	0.0116	0.0140
**CL68**	0.0185	0.0189	0.0420	0.0225	0.0363	0.1563	0.0341	0.0635	0.0173	0.0144	0.0518	0.0102	0.0180	0.0131	0.0094	0.0718	0.0098	0.0095	0.0502	0.0148	0.1435	0.0229	0.0094	0.0127	0.0271	0.0102	0.0179	0.0096	0.0175	0.0174	0.0336	0.0213	0.0199	0.0173	0.0128	0.0164	0.0177	0.0197	0.0115	0.0185	0.0155	0.0171	0.0178	0.0173	0.0184	0.0177	0.0142	0.0174
**CL69**	0.0359	0.0352	0.0625	0.0378	0.0543	0.1631	0.0573	0.0794	0.0352	0.0359	0.0732	0.0393	0.0351	0.0345	0.0379	0.0924	0.0380	0.0379	0.0667	0.0378	0.1188	0.0383	0.0379	0.0386	0.0436	0.0382	0.0343	0.0379	0.0341	0.0334	0.0508	0.0335	0.0335	0.0349	0.0332	0.0400	0.0340	0.0349	0.0395	0.0350	0.0330	0.0336	0.0340	0.0336	0.0338	0.0347	0.0345	0.0350
**CL70**	0.0942	0.0970	0.0993	0.0940	0.1141	0.1297	0.1124	0.1006	0.0935	0.0911	0.0611	0.0926	0.0959	0.0910	0.0912	0.0544	0.0910	0.0907	0.0738	0.0934	0.1176	0.0985	0.0908	0.0927	0.1049	0.0910	0.0947	0.0906	0.0943	0.0938	0.0836	0.0984	0.0891	0.0913	0.0908	0.0931	0.0946	0.0952	0.0931	0.0946	0.0928	0.0943	0.0949	0.0947	0.0948	0.0953	0.0909	0.0927
**CL71**	0.0133	0.0128	0.0474	0.0151	0.0356	0.1440	0.0399	0.0675	0.0122	0.0143	0.0499	0.0218	0.0129	0.0149	0.0201	0.0671	0.0203	0.0203	0.0445	0.0145	0.1414	0.0183	0.0201	0.0205	0.0216	0.0209	0.0123	0.0202	0.0127	0.0112	0.0291	0.0158	0.0125	0.0107	0.0153	0.0162	0.0121	0.0133	0.0228	0.0134	0.0138	0.0124	0.0121	0.0124	0.0123	0.0130	0.0167	0.0111
**CL72**	0.0498	0.0506	0.0544	0.0542	0.0708	0.1930	0.0736	0.0546	0.0492	0.0501	0.0473	0.0500	0.0503	0.0506	0.0489	0.0647	0.0490	0.0489	0.0816	0.0488	0.1007	0.0533	0.0488	0.0505	0.0589	0.0492	0.0485	0.0488	0.0489	0.0480	0.0435	0.0498	0.0470	0.0495	0.0492	0.0506	0.0484	0.0509	0.0514	0.0494	0.0489	0.0479	0.0482	0.0482	0.0503	0.0498	0.0494	0.0500
**CL73**	0.0100	0.0099	0.0453	0.0119	0.0333	0.1453	0.0374	0.0647	0.0090	0.0117	0.0483	0.0188	0.0101	0.0119	0.0170	0.0662	0.0171	0.0171	0.0428	0.0111	0.1417	0.0153	0.0169	0.0174	0.0183	0.0179	0.0088	0.0171	0.0096	0.0084	0.0254	0.0126	0.0107	0.0094	0.0119	0.0132	0.0086	0.0104	0.0198	0.0103	0.0104	0.0088	0.0090	0.0089	0.0091	0.0102	0.0137	0.0086
**CL74**	0.0161	0.0160	0.0521	0.0201	0.0414	0.1498	0.0448	0.0743	0.0142	0.0195	0.0583	0.0236	0.0152	0.0216	0.0241	0.0741	0.0241	0.0240	0.0491	0.0176	0.1515	0.0202	0.0239	0.0228	0.0244	0.0241	0.0154	0.0239	0.0127	0.0140	0.0192	0.0168	0.0149	0.0146	0.0201	0.0209	0.0153	0.0138	0.0249	0.0148	0.0161	0.0127	0.0134	0.0129	0.0130	0.0153	0.0207	0.0160
**CL75**	0.1177	0.1183	0.1281	0.1169	0.1464	0.1656	0.1488	0.1277	0.1162	0.1221	0.0884	0.1226	0.1177	0.1222	0.1226	0.0750	0.1227	0.1224	0.0960	0.1163	0.1451	0.1184	0.1223	0.1236	0.1267	0.1230	0.1173	0.1226	0.1176	0.1183	0.1126	0.1158	0.1360	0.1311	0.1210	0.1170	0.1170	0.1173	0.1248	0.1170	0.1179	0.1176	0.1166	0.1174	0.1157	0.1173	0.1231	0.1170
**CL76**	0.0165	0.0175	0.0431	0.0212	0.0318	0.1558	0.0364	0.0596	0.0165	0.0150	0.0510	0.0109	0.0167	0.0145	0.0092	0.0705	0.0096	0.0095	0.0485	0.0133	0.1430	0.0230	0.0094	0.0137	0.0240	0.0096	0.0146	0.0094	0.0160	0.0165	0.0314	0.0214	0.0201	0.0168	0.0139	0.0148	0.0145	0.0170	0.0118	0.0152	0.0151	0.0152	0.0161	0.0153	0.0171	0.0164	0.0109	0.0175
**FC27**	0.0167	0.0154	0.0403	0.0199	0.0311	0.1521	0.0329	0.0637	0.0153	0.0117	0.0523	0.0152	0.0145	0.0108	0.0141	0.0724	0.0143	0.0142	0.0475	0.0184	0.1419	0.0196	0.0142	0.0120	0.0248	0.0151	0.0166	0.0141	0.0155	0.0151	0.0326	0.0180	0.0185	0.0168	0.0094	0.0219	0.0165	0.0150	0.0160	0.0154	0.0116	0.0152	0.0158	0.0152	0.0144	0.0154	0.0141	0.0163
**FC3**	0.0086	0.0058	0.0451	0.0145	0.0340	0.1474	0.0388	0.0669	0.0092	0.0133	0.0515	0.0198	0.0058	0.0153	0.0179	0.0677	0.0182	0.0181	0.0432	0.0119	0.1440	0.0155	0.0180	0.0147	0.0182	0.0186	0.0091	0.0180	0.0096	0.0088	0.0268	0.0131	0.0122	0.0099	0.0145	0.0147	0.0092	0.0107	0.0204	0.0101	0.0117	0.0095	0.0099	0.0097	0.0091	0.0066	0.0157	0.0101
**FC28**	0.0164	0.0151	0.0406	0.0200	0.0313	0.1521	0.0326	0.0633	0.0154	0.0120	0.0518	0.0151	0.0144	0.0109	0.0141	0.0725	0.0144	0.0144	0.0475	0.0188	0.1419	0.0197	0.0143	0.0124	0.0242	0.0149	0.0166	0.0142	0.0156	0.0154	0.0322	0.0179	0.0180	0.0166	0.0088	0.0220	0.0165	0.0152	0.0161	0.0150	0.0118	0.0153	0.0161	0.0154	0.0142	0.0151	0.0140	0.0161
**FC29**	0.0098	0.0106	0.0450	0.0135	0.0359	0.1447	0.0368	0.0663	0.0089	0.0120	0.0501	0.0184	0.0095	0.0138	0.0171	0.0657	0.0175	0.0173	0.0416	0.0118	0.1422	0.0145	0.0173	0.0176	0.0182	0.0177	0.0091	0.0173	0.0086	0.0089	0.0262	0.0132	0.0117	0.0087	0.0142	0.0135	0.0090	0.0108	0.0193	0.0090	0.0121	0.0088	0.0087	0.0090	0.0099	0.0086	0.0126	0.0082
**FC30**	0.0133	0.0129	0.0445	0.0167	0.0384	0.1470	0.0409	0.0648	0.0128	0.0170	0.0533	0.0214	0.0121	0.0190	0.0215	0.0692	0.0219	0.0217	0.0426	0.0157	0.1403	0.0133	0.0217	0.0198	0.0211	0.0220	0.0131	0.0217	0.0117	0.0126	0.0298	0.0103	0.0110	0.0124	0.0170	0.0185	0.0129	0.0122	0.0227	0.0117	0.0138	0.0119	0.0120	0.0120	0.0110	0.0121	0.0180	0.0129
**FC31**	0.0172	0.0184	0.0323	0.0184	0.0371	0.1546	0.0359	0.0659	0.0190	0.0144	0.0568	0.0164	0.0190	0.0146	0.0155	0.0768	0.0156	0.0156	0.0460	0.0211	0.1393	0.0195	0.0154	0.0136	0.0276	0.0165	0.0182	0.0156	0.0184	0.0182	0.0362	0.0200	0.0212	0.0200	0.0143	0.0233	0.0182	0.0190	0.0183	0.0185	0.0174	0.0186	0.0190	0.0186	0.0200	0.0198	0.0167	0.0191
**FC32**	0.0172	0.0168	0.0320	0.0182	0.0363	0.1536	0.0359	0.0644	0.0171	0.0138	0.0562	0.0161	0.0173	0.0139	0.0153	0.0763	0.0156	0.0153	0.0467	0.0196	0.1388	0.0179	0.0153	0.0128	0.0276	0.0162	0.0179	0.0153	0.0168	0.0161	0.0354	0.0182	0.0203	0.0190	0.0129	0.0231	0.0180	0.0189	0.0178	0.0181	0.0158	0.0170	0.0173	0.0169	0.0181	0.0179	0.0147	0.0176
**FC33**	0.0166	0.0169	0.0525	0.0185	0.0419	0.1460	0.0422	0.0726	0.0169	0.0190	0.0570	0.0245	0.0170	0.0199	0.0229	0.0743	0.0233	0.0232	0.0491	0.0193	0.1504	0.0224	0.0233	0.0248	0.0239	0.0232	0.0142	0.0232	0.0166	0.0162	0.0235	0.0195	0.0181	0.0170	0.0207	0.0185	0.0141	0.0158	0.0256	0.0153	0.0177	0.0158	0.0156	0.0157	0.0163	0.0168	0.0202	0.0157
**FC34**	0.0164	0.0169	0.0524	0.0185	0.0418	0.1459	0.0422	0.0727	0.0170	0.0190	0.0569	0.0246	0.0172	0.0200	0.0230	0.0743	0.0233	0.0231	0.0491	0.0192	0.1503	0.0226	0.0232	0.0248	0.0240	0.0233	0.0142	0.0231	0.0167	0.0163	0.0234	0.0197	0.0181	0.0170	0.0207	0.0183	0.0142	0.0159	0.0257	0.0154	0.0177	0.0158	0.0157	0.0158	0.0165	0.0168	0.0202	0.0158
**FC35**	0.0102	0.0116	0.0456	0.0153	0.0340	0.1457	0.0365	0.0678	0.0097	0.0097	0.0502	0.0201	0.0100	0.0152	0.0187	0.0665	0.0191	0.0189	0.0429	0.0121	0.1420	0.0155	0.0188	0.0185	0.0186	0.0194	0.0103	0.0189	0.0103	0.0102	0.0274	0.0151	0.0136	0.0104	0.0154	0.0142	0.0102	0.0127	0.0212	0.0104	0.0133	0.0107	0.0101	0.0106	0.0111	0.0101	0.0144	0.0087
**DS3**	0.0120	0.0120	0.0436	0.0164	0.0385	0.1464	0.0403	0.0656	0.0101	0.0156	0.0532	0.0201	0.0111	0.0183	0.0200	0.0701	0.0203	0.0203	0.0429	0.0140	0.1402	0.0143	0.0201	0.0194	0.0205	0.0203	0.0113	0.0202	0.0104	0.0108	0.0279	0.0095	0.0098	0.0112	0.0166	0.0168	0.0112	0.0123	0.0215	0.0105	0.0137	0.0104	0.0103	0.0105	0.0105	0.0107	0.0162	0.0117
**DS4**	0.0174	0.0169	0.0378	0.0216	0.0351	0.1520	0.0344	0.0622	0.0154	0.0133	0.0549	0.0145	0.0163	0.0129	0.0144	0.0751	0.0147	0.0148	0.0479	0.0192	0.1409	0.0193	0.0147	0.0137	0.0246	0.0151	0.0162	0.0146	0.0151	0.0157	0.0329	0.0151	0.0161	0.0162	0.0112	0.0218	0.0163	0.0163	0.0158	0.0154	0.0132	0.0149	0.0151	0.0150	0.0147	0.0161	0.0134	0.0164

**FC4**	0.0723	0.0729	0.0723	0.0700	0.0611	0.1092	0.0468	0.0923	0.0733	0.0710	0.0834	0.0750	0.0731	0.0695	0.0745	0.0772	0.0745	0.0741	0.0523	0.0764	0.1025	0.0756	0.0743	0.0720	0.0665	0.0749	0.0738	0.0743	0.0734	0.0731	0.0908	0.0747	0.0745	0.0746	0.0698	0.0758	0.0739	0.0733	0.0766	0.0742	0.0706	0.0736	0.0738	0.0736	0.0720	0.0730	0.0707	0.0720
**DS5**	0.0592	0.0600	0.0574	0.0639	0.0736	0.1941	0.0764	0.0584	0.0587	0.0541	0.0487	0.0541	0.0598	0.0543	0.0536	0.0698	0.0538	0.0537	0.0885	0.0585	0.1085	0.0631	0.0537	0.0553	0.0667	0.0541	0.0591	0.0537	0.0582	0.0579	0.0521	0.0594	0.0498	0.0566	0.0523	0.0609	0.0590	0.0592	0.0558	0.0587	0.0555	0.0579	0.0586	0.0582	0.0584	0.0591	0.0563	0.0599
**DS6**	0.1553	0.1575	0.1606	0.1570	0.1730	0.2189	0.1728	0.1537	0.1540	0.1545	0.1183	0.1564	0.1560	0.1551	0.1561	0.1069	0.1553	0.1557	0.1287	0.1544	0.0990	0.1574	0.1557	0.1564	0.1649	0.1558	0.1554	0.1550	0.1547	0.1555	0.1495	0.1527	0.1230	0.1399	0.1534	0.1554	0.1558	0.1549	0.1575	0.1557	0.1541	0.1548	0.1553	0.1550	0.1544	0.1556	0.1560	0.1555
**DS7**	0.0190	0.0177	0.0389	0.0219	0.0344	0.1524	0.0357	0.0614	0.0172	0.0143	0.0544	0.0178	0.0171	0.0128	0.0166	0.0749	0.0168	0.0168	0.0481	0.0207	0.1395	0.0197	0.0167	0.0150	0.0271	0.0175	0.0189	0.0167	0.0179	0.0176	0.0343	0.0147	0.0163	0.0198	0.0118	0.0239	0.0187	0.0178	0.0184	0.0186	0.0142	0.0179	0.0182	0.0179	0.0168	0.0172	0.0162	0.0185
**DS8**	0.0137	0.0128	0.0443	0.0165	0.0373	0.1461	0.0408	0.0641	0.0122	0.0168	0.0536	0.0213	0.0125	0.0176	0.0213	0.0697	0.0215	0.0213	0.0429	0.0153	0.1394	0.0126	0.0212	0.0202	0.0212	0.0216	0.0131	0.0213	0.0109	0.0125	0.0292	0.0091	0.0101	0.0132	0.0163	0.0184	0.0129	0.0115	0.0227	0.0132	0.0134	0.0115	0.0116	0.0115	0.0113	0.0123	0.0178	0.0132
**DS9**	0.0131	0.0123	0.0445	0.0157	0.0372	0.1524	0.0408	0.0657	0.0116	0.0162	0.0557	0.0211	0.0126	0.0168	0.0207	0.0720	0.0210	0.0209	0.0443	0.0151	0.1455	0.0124	0.0207	0.0197	0.0210	0.0213	0.0127	0.0207	0.0104	0.0121	0.0300	0.0085	0.0092	0.0125	0.0154	0.0184	0.0125	0.0110	0.0224	0.0128	0.0127	0.0108	0.0110	0.0111	0.0109	0.0122	0.0175	0.0129
**DS10**	0.0102	0.0108	0.0445	0.0160	0.0359	0.1483	0.0381	0.0676	0.0096	0.0129	0.0516	0.0183	0.0098	0.0167	0.0180	0.0664	0.0182	0.0181	0.0416	0.0124	0.1434	0.0127	0.0180	0.0169	0.0189	0.0188	0.0105	0.0180	0.0096	0.0101	0.0271	0.0134	0.0129	0.0098	0.0133	0.0162	0.0104	0.0106	0.0197	0.0088	0.0122	0.0092	0.0090	0.0092	0.0098	0.0094	0.0155	0.0109
**DS11**	0.0129	0.0121	0.0444	0.0160	0.0375	0.1472	0.0411	0.0645	0.0108	0.0168	0.0523	0.0210	0.0125	0.0176	0.0211	0.0711	0.0212	0.0212	0.0442	0.0150	0.1402	0.0154	0.0210	0.0206	0.0200	0.0215	0.0124	0.0211	0.0113	0.0121	0.0279	0.0086	0.0092	0.0122	0.0155	0.0177	0.0123	0.0110	0.0226	0.0113	0.0124	0.0116	0.0113	0.0118	0.0092	0.0120	0.0172	0.0126
**DS12**	0.0167	0.0165	0.0414	0.0192	0.0331	0.1525	0.0331	0.0638	0.0166	0.0128	0.0519	0.0154	0.0161	0.0110	0.0154	0.0732	0.0157	0.0155	0.0478	0.0198	0.1420	0.0198	0.0155	0.0138	0.0245	0.0158	0.0172	0.0155	0.0156	0.0163	0.0329	0.0175	0.0178	0.0165	0.0094	0.0214	0.0170	0.0155	0.0165	0.0150	0.0121	0.0155	0.0158	0.0157	0.0147	0.0158	0.0134	0.0156
**HL1**	0.0796	0.0822	0.0636	0.0832	0.0705	0.1780	0.0569	0.0576	0.0806	0.0782	0.0747	0.0800	0.0803	0.0796	0.0784	0.0947	0.0788	0.0786	0.1127	0.0795	0.0983	0.0857	0.0785	0.0814	0.0726	0.0787	0.0786	0.0786	0.0800	0.0802	0.0646	0.0835	0.0829	0.0842	0.0802	0.0795	0.0788	0.0801	0.0813	0.0788	0.0798	0.0803	0.0800	0.0803	0.0803	0.0800	0.0788	0.0791
**HL2**	0.0340	0.0342	0.0573	0.0387	0.0487	0.1703	0.0545	0.0713	0.0335	0.0323	0.0708	0.0351	0.0338	0.0315	0.0337	0.0913	0.0339	0.0337	0.0649	0.0362	0.1587	0.0371	0.0337	0.0344	0.0423	0.0342	0.0327	0.0335	0.0330	0.0328	0.0494	0.0344	0.0328	0.0342	0.0306	0.0384	0.0326	0.0354	0.0360	0.0333	0.0318	0.0323	0.0325	0.0324	0.0341	0.0333	0.0279	0.0336
**FC5**	0.0178	0.0178	0.0392	0.0214	0.0356	0.1526	0.0350	0.0619	0.0158	0.0146	0.0541	0.0149	0.0175	0.0124	0.0148	0.0756	0.0151	0.0150	0.0488	0.0198	0.1395	0.0196	0.0150	0.0147	0.0259	0.0154	0.0175	0.0149	0.0163	0.0168	0.0333	0.0143	0.0153	0.0175	0.0102	0.0224	0.0174	0.0173	0.0165	0.0165	0.0134	0.0163	0.0165	0.0163	0.0153	0.0174	0.0146	0.0173
**HL3**	0.0118	0.0108	0.0432	0.0148	0.0359	0.1446	0.0394	0.0618	0.0109	0.0153	0.0513	0.0202	0.0116	0.0147	0.0186	0.0679	0.0190	0.0188	0.0426	0.0134	0.1386	0.0147	0.0186	0.0196	0.0192	0.0188	0.0100	0.0188	0.0109	0.0105	0.0269	0.0098	0.0098	0.0115	0.0136	0.0157	0.0098	0.0114	0.0212	0.0113	0.0114	0.0101	0.0103	0.0102	0.0098	0.0109	0.0153	0.0111
**HL4**	0.0189	0.0176	0.0373	0.0193	0.0367	0.1543	0.0376	0.0630	0.0190	0.0169	0.0563	0.0176	0.0199	0.0128	0.0169	0.0784	0.0171	0.0170	0.0511	0.0219	0.1362	0.0199	0.0168	0.0151	0.0275	0.0177	0.0194	0.0170	0.0188	0.0191	0.0351	0.0167	0.0187	0.0204	0.0119	0.0250	0.0192	0.0189	0.0192	0.0193	0.0147	0.0179	0.0186	0.0180	0.0174	0.0192	0.0163	0.0193
**HL5**	0.0168	0.0175	0.0371	0.0208	0.0354	0.1516	0.0320	0.0615	0.0156	0.0127	0.0540	0.0138	0.0165	0.0113	0.0121	0.0746	0.0124	0.0122	0.0461	0.0188	0.1410	0.0197	0.0121	0.0137	0.0247	0.0123	0.0150	0.0122	0.0157	0.0155	0.0331	0.0168	0.0174	0.0171	0.0110	0.0199	0.0147	0.0169	0.0145	0.0148	0.0138	0.0159	0.0161	0.0159	0.0168	0.0159	0.0127	0.0158
**HL6**	0.0513	0.0527	0.0510	0.0567	0.0695	0.1791	0.0654	0.0486	0.0511	0.0479	0.0451	0.0462	0.0516	0.0466	0.0447	0.0660	0.0450	0.0447	0.0837	0.0498	0.1102	0.0544	0.0449	0.0468	0.0605	0.0454	0.0505	0.0448	0.0506	0.0508	0.0457	0.0513	0.0411	0.0450	0.0447	0.0530	0.0505	0.0522	0.0476	0.0507	0.0485	0.0500	0.0507	0.0501	0.0513	0.0511	0.0480	0.0514
**HL7**	0.0104	0.0113	0.0384	0.0139	0.0370	0.1471	0.0399	0.0654	0.0119	0.0147	0.0530	0.0206	0.0118	0.0172	0.0189	0.0691	0.0192	0.0191	0.0408	0.0135	0.1384	0.0139	0.0191	0.0177	0.0198	0.0200	0.0104	0.0192	0.0115	0.0108	0.0282	0.0122	0.0125	0.0120	0.0172	0.0160	0.0102	0.0131	0.0217	0.0118	0.0143	0.0109	0.0112	0.0110	0.0125	0.0120	0.0170	0.0117
**HL8**	0.0169	0.0177	0.0314	0.0177	0.0365	0.1545	0.0349	0.0651	0.0180	0.0139	0.0566	0.0159	0.0185	0.0140	0.0148	0.0766	0.0153	0.0150	0.0459	0.0205	0.1387	0.0186	0.0150	0.0132	0.0263	0.0159	0.0173	0.0150	0.0179	0.0175	0.0355	0.0192	0.0212	0.0199	0.0138	0.0225	0.0171	0.0182	0.0176	0.0174	0.0165	0.0177	0.0180	0.0178	0.0190	0.0188	0.0158	0.0184
**BL1**	0.3873	0.3901	0.3648	0.3866	0.3750	0.2650	0.3641	0.3576	0.3866	0.3860	0.3488	0.3874	0.3873	0.3866	0.3873	0.3360	0.3871	0.3866	0.3617	0.3867	0.2737	0.3874	0.3870	0.3884	0.3804	0.3869	0.3871	0.3868	0.3869	0.3879	0.3752	0.3867	0.3955	0.4019	0.3873	0.3864	0.3863	0.3869	0.3872	0.3865	0.3863	0.3869	0.3870	0.3871	0.3867	0.3867	0.3880	0.3878
**HL9**	0.0150	0.0150	0.0485	0.0174	0.0388	0.1478	0.0405	0.0651	0.0146	0.0163	0.0483	0.0184	0.0148	0.0180	0.0164	0.0676	0.0167	0.0165	0.0456	0.0107	0.1415	0.0205	0.0164	0.0205	0.0228	0.0169	0.0142	0.0166	0.0142	0.0148	0.0292	0.0174	0.0151	0.0128	0.0178	0.0132	0.0141	0.0151	0.0189	0.0148	0.0158	0.0143	0.0147	0.0145	0.0138	0.0145	0.0187	0.0134
**CL1**	0.0453	0.0467	0.0727	0.0497	0.0679	0.1888	0.0684	0.0961	0.0451	0.0453	0.0874	0.0483	0.0461	0.0457	0.0470	0.1067	0.0471	0.0470	0.0778	0.0475	0.1602	0.0482	0.0467	0.0481	0.0535	0.0476	0.0435	0.0469	0.0438	0.0456	0.0628	0.0449	0.0365	0.0405	0.0456	0.0499	0.0435	0.0452	0.0490	0.0436	0.0449	0.0435	0.0440	0.0437	0.0450	0.0451	0.0449	0.0460
**BL2**	0.0568	0.0579	0.0634	0.0610	0.0699	0.1786	0.0759	0.0697	0.0567	0.0542	0.0689	0.0566	0.0577	0.0537	0.0558	0.0923	0.0558	0.0557	0.0883	0.0582	0.1568	0.0603	0.0559	0.0561	0.0649	0.0560	0.0559	0.0557	0.0562	0.0564	0.0446	0.0560	0.0466	0.0508	0.0516	0.0607	0.0559	0.0569	0.0579	0.0563	0.0545	0.0557	0.0563	0.0559	0.0562	0.0569	0.0507	0.0566
**FC6**	0.0114	0.0119	0.0428	0.0161	0.0374	0.1462	0.0391	0.0648	0.0103	0.0146	0.0529	0.0193	0.0107	0.0170	0.0182	0.0694	0.0184	0.0184	0.0424	0.0125	0.1407	0.0134	0.0182	0.0190	0.0199	0.0188	0.0101	0.0183	0.0094	0.0095	0.0274	0.0102	0.0101	0.0104	0.0154	0.0154	0.0100	0.0113	0.0205	0.0103	0.0133	0.0096	0.0098	0.0096	0.0107	0.0106	0.0150	0.0108
**HL10**	0.0870	0.0898	0.0623	0.0902	0.0645	0.1387	0.0774	0.0521	0.0867	0.0832	0.0436	0.0848	0.0876	0.0851	0.0840	0.0362	0.0845	0.0839	0.0581	0.0870	0.0709	0.0926	0.0840	0.0862	0.0780	0.0848	0.0871	0.0837	0.0879	0.0874	0.0807	0.0922	0.0870	0.0893	0.0831	0.0859	0.0869	0.0895	0.0870	0.0871	0.0861	0.0874	0.0877	0.0876	0.0883	0.0877	0.0816	0.0860
**BL3**	0.0415	0.0428	0.0466	0.0462	0.0546	0.1826	0.0602	0.0430	0.0412	0.0387	0.0339	0.0385	0.0414	0.0387	0.0368	0.0544	0.0370	0.0370	0.0745	0.0404	0.1175	0.0479	0.0368	0.0387	0.0498	0.0378	0.0394	0.0368	0.0414	0.0404	0.0360	0.0469	0.0413	0.0394	0.0386	0.0418	0.0393	0.0421	0.0395	0.0404	0.0402	0.0409	0.0414	0.0409	0.0426	0.0417	0.0363	0.0419
**HL11**	0.1213	0.1227	0.1298	0.1231	0.1418	0.2746	0.1472	0.1296	0.1219	0.1212	0.1139	0.1230	0.1228	0.1209	0.1223	0.1349	0.1223	0.1227	0.1565	0.1218	0.1563	0.1238	0.1225	0.1230	0.1323	0.1230	0.1226	0.1224	0.1230	0.1222	0.1160	0.1250	0.1210	0.1242	0.1205	0.1236	0.1227	0.1226	0.1247	0.1231	0.1207	0.1226	0.1225	0.1223	0.1215	0.1231	0.1220	0.1229
**HL12**	0.0126	0.0117	0.0435	0.0153	0.0358	0.1455	0.0396	0.0629	0.0102	0.0145	0.0515	0.0213	0.0111	0.0155	0.0194	0.0702	0.0198	0.0197	0.0427	0.0131	0.1392	0.0151	0.0195	0.0193	0.0202	0.0202	0.0113	0.0196	0.0109	0.0101	0.0278	0.0095	0.0097	0.0118	0.0153	0.0162	0.0111	0.0112	0.0219	0.0119	0.0125	0.0112	0.0110	0.0112	0.0103	0.0115	0.0157	0.0113
**HL13**	0.0415	0.0417	0.0652	0.0414	0.0603	0.1659	0.0629	0.0859	0.0419	0.0428	0.0769	0.0440	0.0416	0.0433	0.0441	0.0939	0.0442	0.0440	0.0715	0.0435	0.1540	0.0413	0.0440	0.0429	0.0498	0.0441	0.0422	0.0440	0.0419	0.0417	0.0544	0.0421	0.0450	0.0447	0.0417	0.0428	0.0418	0.0425	0.0461	0.0420	0.0414	0.0414	0.0421	0.0415	0.0416	0.0413	0.0420	0.0406
**HL14**	0.0122	0.0118	0.0441	0.0155	0.0372	0.1462	0.0405	0.0640	0.0114	0.0157	0.0515	0.0210	0.0115	0.0169	0.0202	0.0701	0.0205	0.0204	0.0426	0.0142	0.1398	0.0154	0.0203	0.0204	0.0198	0.0207	0.0117	0.0204	0.0111	0.0115	0.0278	0.0106	0.0102	0.0107	0.0151	0.0169	0.0115	0.0114	0.0222	0.0107	0.0128	0.0115	0.0111	0.0117	0.0098	0.0114	0.0163	0.0119
**HL15**	0.0124	0.0124	0.0403	0.0146	0.0396	0.1489	0.0423	0.0674	0.0112	0.0165	0.0556	0.0226	0.0131	0.0199	0.0217	0.0721	0.0221	0.0219	0.0446	0.0152	0.1383	0.0134	0.0219	0.0188	0.0229	0.0225	0.0136	0.0220	0.0124	0.0120	0.0299	0.0126	0.0139	0.0141	0.0192	0.0184	0.0136	0.0145	0.0238	0.0135	0.0162	0.0127	0.0126	0.0128	0.0135	0.0135	0.0180	0.0136
**HL16**	0.0159	0.0151	0.0429	0.0178	0.0281	0.1494	0.0347	0.0592	0.0160	0.0125	0.0484	0.0170	0.0158	0.0104	0.0148	0.0720	0.0152	0.0152	0.0474	0.0179	0.1420	0.0211	0.0148	0.0163	0.0238	0.0157	0.0145	0.0150	0.0153	0.0140	0.0314	0.0180	0.0178	0.0155	0.0103	0.0189	0.0143	0.0152	0.0178	0.0154	0.0122	0.0146	0.0145	0.0146	0.0149	0.0152	0.0084	0.0135
**HL17**	0.0358	0.0359	0.0516	0.0376	0.0578	0.1700	0.0592	0.0482	0.0354	0.0367	0.0348	0.0413	0.0362	0.0387	0.0401	0.0524	0.0405	0.0402	0.0685	0.0347	0.1218	0.0415	0.0401	0.0421	0.0427	0.0402	0.0350	0.0403	0.0351	0.0345	0.0310	0.0383	0.0321	0.0334	0.0384	0.0356	0.0349	0.0360	0.0423	0.0353	0.0356	0.0346	0.0345	0.0348	0.0344	0.0356	0.0391	0.0345
**HL18**	0.0475	0.0485	0.0616	0.0519	0.0734	0.1766	0.0702	0.0568	0.0469	0.0500	0.0432	0.0534	0.0467	0.0513	0.0514	0.0610	0.0517	0.0516	0.0808	0.0459	0.1147	0.0525	0.0515	0.0534	0.0568	0.0517	0.0469	0.0515	0.0464	0.0456	0.0416	0.0503	0.0379	0.0391	0.0523	0.0476	0.0466	0.0485	0.0538	0.0473	0.0498	0.0466	0.0465	0.0468	0.0475	0.0460	0.0506	0.0458
**FC7**	0.0169	0.0164	0.0332	0.0181	0.0368	0.1544	0.0359	0.0641	0.0172	0.0145	0.0565	0.0155	0.0173	0.0141	0.0152	0.0765	0.0154	0.0153	0.0474	0.0198	0.1396	0.0173	0.0152	0.0137	0.0266	0.0159	0.0174	0.0152	0.0159	0.0166	0.0348	0.0172	0.0195	0.0185	0.0128	0.0232	0.0173	0.0180	0.0172	0.0174	0.0151	0.0156	0.0162	0.0157	0.0173	0.0170	0.0142	0.0177
**HL19**	0.0259	0.0254	0.0501	0.0261	0.0422	0.1585	0.0470	0.0690	0.0277	0.0254	0.0585	0.0220	0.0277	0.0237	0.0211	0.0824	0.0215	0.0213	0.0610	0.0243	0.1484	0.0290	0.0212	0.0231	0.0358	0.0217	0.0267	0.0214	0.0273	0.0269	0.0323	0.0297	0.0305	0.0296	0.0234	0.0248	0.0267	0.0277	0.0207	0.0274	0.0245	0.0267	0.0267	0.0267	0.0269	0.0276	0.0204	0.0263
**HL20**	0.0743	0.0746	0.0733	0.0712	0.0630	0.1095	0.0483	0.0947	0.0753	0.0727	0.0851	0.0768	0.0753	0.0714	0.0760	0.0780	0.0764	0.0761	0.0532	0.0782	0.1020	0.0769	0.0761	0.0738	0.0686	0.0767	0.0760	0.0760	0.0755	0.0749	0.0929	0.0766	0.0763	0.0764	0.0717	0.0780	0.0759	0.0755	0.0785	0.0764	0.0725	0.0755	0.0758	0.0757	0.0741	0.0752	0.0724	0.0741
**HL21**	0.0586	0.0594	0.0347	0.0650	0.0358	0.1631	0.0499	0.0231	0.0586	0.0559	0.0508	0.0572	0.0582	0.0571	0.0556	0.0663	0.0561	0.0557	0.0904	0.0587	0.1025	0.0637	0.0559	0.0569	0.0485	0.0562	0.0579	0.0557	0.0583	0.0583	0.0515	0.0629	0.0602	0.0595	0.0557	0.0611	0.0580	0.0603	0.0584	0.0590	0.0572	0.0577	0.0588	0.0579	0.0593	0.0580	0.0538	0.0594
**HL22**	0.0141	0.0142	0.0387	0.0195	0.0313	0.1515	0.0315	0.0632	0.0099	0.0103	0.0513	0.0123	0.0129	0.0089	0.0110	0.0707	0.0114	0.0113	0.0454	0.0156	0.1427	0.0189	0.0111	0.0107	0.0228	0.0115	0.0128	0.0112	0.0125	0.0126	0.0298	0.0181	0.0180	0.0136	0.0083	0.0182	0.0126	0.0148	0.0137	0.0131	0.0110	0.0123	0.0133	0.0124	0.0134	0.0133	0.0098	0.0129
**HL23**	0.0165	0.0157	0.0384	0.0187	0.0359	0.1560	0.0357	0.0680	0.0168	0.0147	0.0562	0.0153	0.0169	0.0140	0.0149	0.0758	0.0151	0.0150	0.0510	0.0199	0.1424	0.0188	0.0149	0.0120	0.0272	0.0158	0.0171	0.0150	0.0173	0.0161	0.0347	0.0217	0.0225	0.0193	0.0129	0.0225	0.0171	0.0185	0.0168	0.0175	0.0150	0.0167	0.0174	0.0167	0.0178	0.0173	0.0153	0.0180
**HL24**	0.0153	0.0139	0.0399	0.0196	0.0312	0.1516	0.0314	0.0635	0.0137	0.0114	0.0525	0.0122	0.0138	0.0094	0.0108	0.0716	0.0111	0.0110	0.0472	0.0174	0.1426	0.0197	0.0108	0.0109	0.0232	0.0111	0.0133	0.0108	0.0132	0.0122	0.0304	0.0183	0.0175	0.0137	0.0083	0.0185	0.0131	0.0147	0.0131	0.0130	0.0107	0.0122	0.0131	0.0123	0.0141	0.0136	0.0113	0.0139
**HL25**	0.0155	0.0142	0.0399	0.0198	0.0314	0.1514	0.0313	0.0633	0.0138	0.0116	0.0530	0.0123	0.0140	0.0098	0.0111	0.0716	0.0115	0.0113	0.0472	0.0173	0.1425	0.0197	0.0112	0.0111	0.0232	0.0117	0.0134	0.0112	0.0134	0.0128	0.0307	0.0185	0.0179	0.0145	0.0085	0.0186	0.0131	0.0147	0.0136	0.0132	0.0108	0.0124	0.0131	0.0124	0.0143	0.0139	0.0114	0.0142
**BL4**	0.0778	0.0785	0.0595	0.0811	0.0665	0.2017	0.0793	0.0529	0.0794	0.0825	0.0712	0.0850	0.0787	0.0840	0.0841	0.0925	0.0850	0.0846	0.1135	0.0785	0.1348	0.0823	0.0846	0.0852	0.0683	0.0847	0.0777	0.0846	0.0794	0.0787	0.0712	0.0820	0.0869	0.0832	0.0818	0.0812	0.0778	0.0794	0.0872	0.0784	0.0793	0.0785	0.0787	0.0790	0.0771	0.0784	0.0848	0.0801
**DS12.1**	0.0087	0.0064	0.0452	0.0156	0.0348	0.1471	0.0383	0.0673	0.0096	0.0133	0.0521	0.0202	0.0050	0.0163	0.0180	0.0667	0.0183	0.0183	0.0422	0.0120	0.1438	0.0152	0.0181	0.0140	0.0190	0.0187	0.0094	0.0182	0.0099	0.0089	0.0274	0.0136	0.0121	0.0101	0.0153	0.0148	0.0092	0.0121	0.0206	0.0103	0.0129	0.0097	0.0101	0.0100	0.0103	0.0055	0.0160	0.0102
**DS13**	0.0091	0.0073	0.0449	0.0156	0.0339	0.1466	0.0386	0.0670	0.0093	0.0128	0.0511	0.0197	0.0068	0.0163	0.0181	0.0669	0.0184	0.0183	0.0422	0.0120	0.1429	0.0160	0.0182	0.0157	0.0184	0.0189	0.0093	0.0183	0.0100	0.0090	0.0263	0.0147	0.0126	0.0098	0.0156	0.0145	0.0091	0.0119	0.0205	0.0102	0.0128	0.0096	0.0100	0.0098	0.0099	0.0076	0.0157	0.0103
**FC8**	0.0125	0.0130	0.0466	0.0176	0.0383	0.1492	0.0400	0.0660	0.0116	0.0160	0.0503	0.0143	0.0124	0.0183	0.0133	0.0664	0.0136	0.0134	0.0450	0.0079	0.1416	0.0172	0.0134	0.0168	0.0211	0.0139	0.0116	0.0135	0.0112	0.0115	0.0274	0.0161	0.0145	0.0120	0.0170	0.0111	0.0114	0.0130	0.0150	0.0115	0.0135	0.0108	0.0105	0.0110	0.0116	0.0118	0.0165	0.0126
**DS14**	0.0390	0.0389	0.0568	0.0402	0.0468	0.1253	0.0410	0.0779	0.0397	0.0395	0.0558	0.0450	0.0399	0.0407	0.0443	0.0696	0.0445	0.0443	0.0452	0.0427	0.1196	0.0423	0.0443	0.0422	0.0372	0.0448	0.0408	0.0444	0.0398	0.0397	0.0562	0.0409	0.0401	0.0397	0.0388	0.0447	0.0404	0.0397	0.0461	0.0400	0.0374	0.0397	0.0399	0.0400	0.0384	0.0392	0.0417	0.0397
**DS15**	0.0175	0.0173	0.0325	0.0186	0.0364	0.1534	0.0361	0.0646	0.0178	0.0141	0.0564	0.0166	0.0179	0.0142	0.0158	0.0761	0.0162	0.0160	0.0467	0.0201	0.1385	0.0185	0.0159	0.0132	0.0280	0.0169	0.0185	0.0159	0.0175	0.0167	0.0356	0.0186	0.0200	0.0186	0.0133	0.0237	0.0184	0.0194	0.0184	0.0186	0.0165	0.0174	0.0180	0.0176	0.0189	0.0184	0.0153	0.0181
**DS16**	0.0123	0.0112	0.0467	0.0153	0.0362	0.1475	0.0390	0.0668	0.0108	0.0151	0.0513	0.0200	0.0108	0.0167	0.0196	0.0684	0.0198	0.0198	0.0433	0.0141	0.1413	0.0145	0.0197	0.0189	0.0198	0.0202	0.0125	0.0198	0.0110	0.0114	0.0274	0.0120	0.0121	0.0118	0.0137	0.0178	0.0122	0.0107	0.0210	0.0108	0.0119	0.0110	0.0109	0.0111	0.0096	0.0104	0.0170	0.0120
**DS17**	0.0170	0.0160	0.0409	0.0198	0.0326	0.1527	0.0328	0.0635	0.0163	0.0132	0.0526	0.0142	0.0159	0.0111	0.0141	0.0735	0.0144	0.0141	0.0483	0.0191	0.1418	0.0195	0.0142	0.0131	0.0243	0.0146	0.0171	0.0142	0.0156	0.0163	0.0328	0.0175	0.0184	0.0168	0.0080	0.0226	0.0169	0.0151	0.0152	0.0150	0.0108	0.0153	0.0152	0.0153	0.0140	0.0154	0.0140	0.0165
**FC15**	0.0131	0.0121	0.0442	0.0154	0.0371	0.1464	0.0404	0.0642	0.0116	0.0167	0.0530	0.0208	0.0125	0.0171	0.0208	0.0713	0.0211	0.0212	0.0438	0.0152	0.1395	0.0146	0.0208	0.0203	0.0195	0.0213	0.0125	0.0210	0.0110	0.0116	0.0280	0.0089	0.0093	0.0119	0.0158	0.0177	0.0122	0.0102	0.0220	0.0114	0.0124	0.0110	0.0111	0.0112	0.0087	0.0120	0.0168	0.0121
**DS19**	0.0119	0.0119	0.0436	0.0164	0.0383	0.1474	0.0399	0.0653	0.0103	0.0157	0.0533	0.0199	0.0113	0.0183	0.0201	0.0701	0.0204	0.0203	0.0430	0.0142	0.1411	0.0142	0.0201	0.0194	0.0203	0.0207	0.0116	0.0205	0.0104	0.0108	0.0280	0.0094	0.0099	0.0110	0.0167	0.0167	0.0113	0.0122	0.0216	0.0106	0.0138	0.0105	0.0103	0.0106	0.0106	0.0111	0.0162	0.0113
**HL26**	0.0189	0.0177	0.0374	0.0191	0.0369	0.1541	0.0379	0.0631	0.0193	0.0171	0.0563	0.0177	0.0200	0.0129	0.0169	0.0785	0.0173	0.0171	0.0511	0.0218	0.1359	0.0196	0.0170	0.0151	0.0275	0.0180	0.0195	0.0171	0.0189	0.0193	0.0353	0.0167	0.0189	0.0210	0.0121	0.0249	0.0194	0.0190	0.0194	0.0194	0.0148	0.0181	0.0188	0.0181	0.0175	0.0194	0.0166	0.0193
**CL2**	0.0118	0.0129	0.0460	0.0172	0.0347	0.1475	0.0382	0.0690	0.0113	0.0145	0.0499	0.0203	0.0118	0.0177	0.0187	0.0679	0.0190	0.0189	0.0394	0.0147	0.1429	0.0181	0.0187	0.0201	0.0195	0.0193	0.0104	0.0188	0.0118	0.0120	0.0290	0.0173	0.0146	0.0105	0.0170	0.0158	0.0102	0.0126	0.0211	0.0103	0.0152	0.0124	0.0126	0.0124	0.0127	0.0119	0.0174	0.0122
**CL3**	0.0230	0.0231	0.0348	0.0274	0.0424	0.1625	0.0458	0.0503	0.0252	0.0240	0.0393	0.0244	0.0250	0.0237	0.0233	0.0602	0.0236	0.0236	0.0580	0.0249	0.1289	0.0290	0.0234	0.0223	0.0319	0.0239	0.0244	0.0234	0.0254	0.0245	0.0239	0.0289	0.0275	0.0256	0.0235	0.0265	0.0244	0.0259	0.0253	0.0253	0.0228	0.0247	0.0251	0.0249	0.0252	0.0248	0.0238	0.0256
**CL4**	0.0486	0.0502	0.0861	0.0530	0.0740	0.1654	0.0779	0.1073	0.0493	0.0517	0.0710	0.0589	0.0502	0.0545	0.0571	0.0829	0.0572	0.0570	0.0581	0.0521	0.1541	0.0558	0.0570	0.0577	0.0571	0.0580	0.0479	0.0569	0.0496	0.0487	0.0641	0.0534	0.0413	0.0429	0.0539	0.0528	0.0477	0.0504	0.0597	0.0495	0.0512	0.0489	0.0496	0.0490	0.0497	0.0497	0.0546	0.0489
**FC9**	0.0579	0.0590	0.0403	0.0644	0.0461	0.1617	0.0567	0.0320	0.0584	0.0618	0.0528	0.0650	0.0576	0.0646	0.0630	0.0686	0.0633	0.0630	0.0894	0.0575	0.0996	0.0639	0.0629	0.0643	0.0488	0.0636	0.0566	0.0629	0.0585	0.0577	0.0510	0.0631	0.0568	0.0556	0.0628	0.0600	0.0566	0.0604	0.0657	0.0586	0.0605	0.0577	0.0580	0.0581	0.0588	0.0570	0.0642	0.0587
**HL27**	0.0341	0.0344	0.0296	0.0395	0.0118	0.1394	0.0221	0.0475	0.0339	0.0298	0.0732	0.0326	0.0331	0.0293	0.0312	0.0908	0.0317	0.0312	0.0651	0.0371	0.1240	0.0389	0.0313	0.0312	0.0217	0.0317	0.0332	0.0313	0.0330	0.0327	0.0503	0.0381	0.0405	0.0363	0.0282	0.0392	0.0333	0.0352	0.0339	0.0334	0.0306	0.0323	0.0330	0.0325	0.0342	0.0329	0.0316	0.0342
**BL5**	0.0491	0.0495	0.0730	0.0510	0.0679	0.1873	0.0717	0.0844	0.0488	0.0485	0.0717	0.0479	0.0493	0.0473	0.0461	0.0921	0.0462	0.0462	0.0823	0.0469	0.1274	0.0552	0.0459	0.0490	0.0569	0.0466	0.0467	0.0460	0.0491	0.0478	0.0587	0.0520	0.0497	0.0497	0.0475	0.0485	0.0466	0.0478	0.0484	0.0482	0.0469	0.0485	0.0488	0.0484	0.0482	0.0494	0.0492	0.0495
**CL1**	0.0344	0.0352	0.0543	0.0378	0.0547	0.1728	0.0534	0.0826	0.0355	0.0321	0.0747	0.0344	0.0363	0.0321	0.0333	0.0953	0.0337	0.0336	0.0652	0.0380	0.1566	0.0378	0.0335	0.0326	0.0436	0.0341	0.0345	0.0336	0.0347	0.0351	0.0524	0.0360	0.0303	0.0320	0.0322	0.0399	0.0344	0.0358	0.0359	0.0345	0.0336	0.0343	0.0349	0.0346	0.0360	0.0358	0.0333	0.0356
**CL6**	0.0347	0.0348	0.0327	0.0396	0.0066	0.1398	0.0257	0.0425	0.0341	0.0326	0.0691	0.0367	0.0342	0.0313	0.0352	0.0916	0.0358	0.0354	0.0653	0.0379	0.1217	0.0393	0.0355	0.0349	0.0226	0.0359	0.0347	0.0356	0.0340	0.0342	0.0506	0.0370	0.0407	0.0372	0.0303	0.0405	0.0346	0.0345	0.0379	0.0342	0.0316	0.0342	0.0346	0.0343	0.0335	0.0342	0.0297	0.0351
**BL6**	0.0115	0.0118	0.0426	0.0167	0.0363	0.1461	0.0403	0.0619	0.0107	0.0141	0.0526	0.0208	0.0110	0.0165	0.0189	0.0678	0.0191	0.0190	0.0418	0.0127	0.1400	0.0150	0.0189	0.0189	0.0205	0.0201	0.0102	0.0190	0.0112	0.0099	0.0279	0.0122	0.0108	0.0106	0.0158	0.0155	0.0100	0.0133	0.0217	0.0118	0.0136	0.0105	0.0106	0.0107	0.0121	0.0111	0.0165	0.0109
**BL7**	0.0410	0.0422	0.0462	0.0455	0.0538	0.1809	0.0597	0.0427	0.0405	0.0384	0.0339	0.0378	0.0409	0.0383	0.0366	0.0539	0.0370	0.0368	0.0734	0.0402	0.1168	0.0474	0.0367	0.0381	0.0487	0.0374	0.0389	0.0367	0.0406	0.0396	0.0357	0.0462	0.0401	0.0394	0.0385	0.0412	0.0390	0.0412	0.0389	0.0394	0.0394	0.0400	0.0404	0.0401	0.0417	0.0413	0.0362	0.0415
**BL8**	0.1074	0.1085	0.1349	0.1104	0.1258	0.1760	0.1284	0.1580	0.1071	0.1064	0.1472	0.1115	0.1062	0.1061	0.1097	0.1674	0.1095	0.1099	0.1400	0.1085	0.1992	0.1096	0.1101	0.1094	0.1156	0.1104	0.1060	0.1099	0.1064	0.1061	0.1171	0.1074	0.1078	0.1108	0.1069	0.1114	0.1059	0.1068	0.1122	0.1067	0.1064	0.1065	0.1068	0.1064	0.1067	0.1065	0.1066	0.1068
**HL28**	0.0429	0.0434	0.0444	0.0451	0.0539	0.1781	0.0571	0.0416	0.0422	0.0356	0.0301	0.0377	0.0436	0.0348	0.0362	0.0526	0.0364	0.0362	0.0741	0.0417	0.1181	0.0491	0.0364	0.0391	0.0493	0.0362	0.0415	0.0361	0.0424	0.0422	0.0354	0.0454	0.0387	0.0399	0.0346	0.0424	0.0416	0.0426	0.0390	0.0420	0.0380	0.0420	0.0414	0.0417	0.0416	0.0429	0.0385	0.0417
**HL29**	0.1399	0.1400	0.1666	0.1410	0.1597	0.2114	0.1628	0.1927	0.1397	0.1392	0.1807	0.1431	0.1389	0.1386	0.1423	0.2022	0.1420	0.1423	0.1731	0.1409	0.2331	0.1399	0.1425	0.1415	0.1481	0.1429	0.1383	0.1426	0.1392	0.1389	0.1484	0.1400	0.1534	0.1503	0.1394	0.1441	0.1384	0.1386	0.1450	0.1391	0.1389	0.1394	0.1388	0.1390	0.1382	0.1392	0.1386	0.1395
**BL9**	0.0743	0.0746	0.0778	0.0762	0.0626	0.1132	0.0724	0.0847	0.0728	0.0772	0.0897	0.0840	0.0746	0.0813	0.0829	0.0758	0.0828	0.0825	0.0521	0.0769	0.1138	0.0789	0.0828	0.0821	0.0642	0.0831	0.0732	0.0824	0.0736	0.0740	0.0905	0.0788	0.0722	0.0705	0.0805	0.0772	0.0732	0.0758	0.0857	0.0744	0.0766	0.0736	0.0739	0.0733	0.0739	0.0745	0.0801	0.0732

FC69	FC70	FC71	FC72	FC73	FC74	FC75	DS20	DS7	DS21	DS22	DS23	DS24	DS25	DS26	DS27	DS28	DS29	DS30	FC15	DS26	DS32	DS29	DS34	DS35	DS36	DS37	DS38	DS39	DS40	FC16	DS41	DS42	DS43	DS44	DS45	DS46	DS47	DS48	DS49	DS50	FC17	DS51	DS52	DS53	DS54	DS55	DS56
0.0024																																															
0.0193	0.0191																																														
0.0192	0.0190	0.0129																																													
0.0164	0.0164	0.0138	0.0171																																												
0.0145	0.0143	0.0103	0.0133	0.0183																																											
0.0739	0.0738	0.0753	0.0748	0.0777	0.0723																																										
0.0112	0.0111	0.0190	0.0182	0.0157	0.0144	0.0778																																									
0.0194	0.0193	0.0165	0.0192	0.0243	0.0129	0.0742	0.0190																																								
0.0302	0.0300	0.0280	0.0238	0.0325	0.0255	0.0638	0.0308	0.0271																																							
0.0108	0.0107	0.0210	0.0207	0.0179	0.0169	0.0743	0.0145	0.0201	0.0300																																						
0.0154	0.0154	0.0173	0.0177	0.0148	0.0180	0.0792	0.0154	0.0221	0.0309	0.0140																																					
0.0186	0.0184	0.0177	0.0128	0.0222	0.0134	0.0744	0.0181	0.0179	0.0253	0.0167	0.0177																																				
0.0174	0.0172	0.0167	0.0133	0.0211	0.0123	0.0725	0.0175	0.0154	0.0221	0.0116	0.0177	0.0095																																			
0.0115	0.0112	0.0164	0.0177	0.0131	0.0138	0.0743	0.0104	0.0180	0.0280	0.0128	0.0139	0.0171	0.0149																																		
0.0200	0.0200	0.0173	0.0187	0.0237	0.0129	0.0752	0.0203	0.0161	0.0267	0.0220	0.0234	0.0193	0.0187	0.0195																																	
0.0156	0.0156	0.0214	0.0224	0.0193	0.0186	0.0812	0.0165	0.0208	0.0331	0.0129	0.0102	0.0173	0.0164	0.0142	0.0240																																
0.0177	0.0177	0.0115	0.0105	0.0158	0.0129	0.0763	0.0175	0.0183	0.0254	0.0183	0.0177	0.0132	0.0111	0.0161	0.0192	0.0207																															
0.0403	0.0402	0.0409	0.0415	0.0419	0.0379	0.0726	0.0414	0.0410	0.0539	0.0384	0.0401	0.0404	0.0377	0.0385	0.0427	0.0441	0.0399																														
0.0172	0.0172	0.0145	0.0167	0.0224	0.0106	0.0733	0.0175	0.0095	0.0269	0.0201	0.0217	0.0156	0.0156	0.0172	0.0153	0.0207	0.0164	0.0411																													
0.0110	0.0109	0.0169	0.0173	0.0140	0.0136	0.0751	0.0095	0.0177	0.0276	0.0126	0.0137	0.0166	0.0149	0.0031	0.0191	0.0143	0.0159	0.0380	0.0168																												
0.0148	0.0149	0.0229	0.0214	0.0194	0.0184	0.0788	0.0128	0.0187	0.0327	0.0178	0.0135	0.0183	0.0207	0.0138	0.0223	0.0142	0.0211	0.0436	0.0177	0.0134																											
0.0180	0.0179	0.0175	0.0155	0.0180	0.0168	0.0778	0.0177	0.0204	0.0304	0.0201	0.0177	0.0193	0.0178	0.0171	0.0212	0.0224	0.0140	0.0406	0.0184	0.0170	0.0193																										
0.0211	0.0210	0.0234	0.0250	0.0203	0.0231	0.0835	0.0214	0.0267	0.0356	0.0166	0.0130	0.0195	0.0199	0.0188	0.0279	0.0106	0.0217	0.0449	0.0272	0.0187	0.0170	0.0244																									

0.1096	0.1094	0.1200	0.1170	0.1156	0.1149	0.1485	0.1135	0.1149	0.1033	0.1087	0.1141	0.1178	0.1162	0.1103	0.1046	0.1151	0.1197	0.1417	0.1140	0.1100	0.1134	0.1155	0.1186																								
0.0098	0.0097	0.0154	0.0161	0.0126	0.0126	0.0742	0.0092	0.0177	0.0281	0.0122	0.0122	0.0162	0.0156	0.0076	0.0187	0.0122	0.0154	0.0382	0.0156	0.0083	0.0122	0.0154	0.0187	0.1094																							
0.0101	0.0100	0.0183	0.0173	0.0147	0.0139	0.0763	0.0090	0.0187	0.0294	0.0132	0.0143	0.0167	0.0163	0.0095	0.0196	0.0147	0.0165	0.0394	0.0161	0.0090	0.0117	0.0167	0.0197	0.1109	0.0082																						
0.0196	0.0194	0.0075	0.0147	0.0157	0.0117	0.0738	0.0181	0.0148	0.0278	0.0195	0.0182	0.0170	0.0141	0.0156	0.0185	0.0202	0.0113	0.0393	0.0141	0.0162	0.0218	0.0183	0.0227	0.1191	0.0154	0.0180																					
0.0222	0.0221	0.0226	0.0241	0.0301	0.0175	0.0795	0.0212	0.0225	0.0356	0.0232	0.0231	0.0193	0.0223	0.0248	0.0240	0.0195	0.0231	0.0485	0.0208	0.0241	0.0211	0.0279	0.0204	0.1228	0.0233	0.0243	0.0217																				
0.0205	0.0205	0.0192	0.0210	0.0155	0.0223	0.0857	0.0193	0.0275	0.0368	0.0220	0.0127	0.0212	0.0255	0.0188	0.0279	0.0140	0.0200	0.0473	0.0267	0.0184	0.0156	0.0222	0.0109	0.1209	0.0175	0.0182	0.0216	0.0200																			
0.0134	0.0134	0.0211	0.0202	0.0173	0.0169	0.0779	0.0114	0.0153	0.0315	0.0162	0.0175	0.0202	0.0193	0.0125	0.0204	0.0175	0.0199	0.0417	0.0141	0.0122	0.0135	0.0177	0.0230	0.1077	0.0111	0.0120	0.0202	0.0268	0.0219																		
0.0197	0.0194	0.0076	0.0147	0.0154	0.0117	0.0743	0.0183	0.0152	0.0283	0.0198	0.0184	0.0170	0.0141	0.0156	0.0186	0.0204	0.0114	0.0398	0.0145	0.0162	0.0217	0.0182	0.0226	0.1199	0.0156	0.0181	0.0023	0.0219	0.0212	0.0204																	
0.0182	0.0179	0.0133	0.0159	0.0216	0.0106	0.0728	0.0176	0.0138	0.0259	0.0146	0.0183	0.0139	0.0096	0.0166	0.0172	0.0180	0.0158	0.0375	0.0138	0.0163	0.0213	0.0196	0.0219	0.1158	0.0160	0.0174	0.0128	0.0205	0.0250	0.0193	0.0128																
0.0208	0.0207	0.0232	0.0249	0.0199	0.0228	0.0834	0.0212	0.0263	0.0356	0.0163	0.0128	0.0191	0.0198	0.0183	0.0275	0.0106	0.0212	0.0445	0.0268	0.0183	0.0165	0.0241	0.0028	0.1192	0.0183	0.0195	0.0224	0.0203	0.0109	0.0228	0.0223	0.0218															
0.0178	0.0177	0.0088	0.0143	0.0164	0.0104	0.0754	0.0170	0.0153	0.0275	0.0193	0.0185	0.0166	0.0140	0.0165	0.0166	0.0209	0.0093	0.0391	0.0137	0.0163	0.0207	0.0171	0.0221	0.1197	0.0158	0.0164	0.0080	0.0197	0.0200	0.0193	0.0082	0.0123	0.0219														
0.0131	0.0129	0.0207	0.0194	0.0175	0.0163	0.0755	0.0117	0.0138	0.0293	0.0155	0.0166	0.0186	0.0184	0.0119	0.0193	0.0165	0.0194	0.0415	0.0123	0.0117	0.0094	0.0176	0.0215	0.1088	0.0104	0.0116	0.0195	0.0257	0.0209	0.0093	0.0195	0.0194	0.0213	0.0189													
0.0178	0.0177	0.0131	0.0159	0.0216	0.0106	0.0729	0.0176	0.0134	0.0258	0.0143	0.0183	0.0138	0.0096	0.0166	0.0170	0.0177	0.0157	0.0374	0.0135	0.0161	0.0213	0.0196	0.0221	0.1159	0.0157	0.0171	0.0126	0.0202	0.0251	0.0192	0.0128	0.0023	0.0218	0.0122	0.0191												
0.0223	0.0224	0.0195	0.0212	0.0262	0.0141	0.0799	0.0215	0.0185	0.0328	0.0242	0.0197	0.0158	0.0198	0.0216	0.0203	0.0156	0.0195	0.0450	0.0178	0.0210	0.0166	0.0243	0.0167	0.1236	0.0198	0.0196	0.0190	0.0103	0.0169	0.0233	0.0191	0.0177	0.0164	0.0171	0.0218	0.0174											
0.0182	0.0180	0.0133	0.0160	0.0217	0.0106	0.0729	0.0178	0.0138	0.0260	0.0147	0.0186	0.0139	0.0096	0.0167	0.0170	0.0180	0.0161	0.0376	0.0138	0.0164	0.0214	0.0198	0.0221	0.1159	0.0161	0.0174	0.0128	0.0207	0.0253	0.0195	0.0130	0.0023	0.0219	0.0125	0.0194	0.0024	0.0178										
0.0173	0.0173	0.0284	0.0272	0.0250	0.0229	0.0823	0.0164	0.0281	0.0391	0.0177	0.0180	0.0221	0.0253	0.0197	0.0284	0.0153	0.0259	0.0489	0.0263	0.0195	0.0159	0.0265	0.0153	0.1171	0.0180	0.0189	0.0276	0.0100	0.0149	0.0220	0.0273	0.0260	0.0151	0.0257	0.0207	0.0259	0.0154	0.0262									
0.0181	0.0179	0.0091	0.0142	0.0164	0.0105	0.0754	0.0173	0.0155	0.0276	0.0196	0.0187	0.0167	0.0140	0.0166	0.0168	0.0213	0.0094	0.0395	0.0140	0.0165	0.0207	0.0172	0.0221	0.1196	0.0162	0.0167	0.0082	0.0198	0.0198	0.0195	0.0082	0.0124	0.0217	0.0025	0.0192	0.0124	0.0173	0.0126	0.0257								
0.0176	0.0174	0.0147	0.0168	0.0227	0.0105	0.0735	0.0177	0.0096	0.0272	0.0204	0.0220	0.0157	0.0157	0.0175	0.0154	0.0210	0.0166	0.0413	0.0025	0.0171	0.0181	0.0186	0.0274	0.1140	0.0157	0.0164	0.0142	0.0210	0.0270	0.0144	0.0146	0.0138	0.0271	0.0138	0.0125	0.0136	0.0179	0.0139	0.0267	0.0141							
0.0230	0.0230	0.0226	0.0186	0.0269	0.0184	0.0803	0.0223	0.0212	0.0305	0.0248	0.0199	0.0130	0.0174	0.0203	0.0239	0.0164	0.0167	0.0474	0.0204	0.0198	0.0184	0.0231	0.0178	0.1232	0.0205	0.0205	0.0225	0.0152	0.0167	0.0241	0.0225	0.0211	0.0175	0.0209	0.0231	0.0212	0.0117	0.0212	0.0178	0.0210	0.0205						
0.0144	0.0143	0.0145	0.0118	0.0180	0.0103	0.0733	0.0134	0.0145	0.0234	0.0162	0.0180	0.0113	0.0097	0.0136	0.0162	0.0179	0.0104	0.0389	0.0135	0.0134	0.0168	0.0162	0.0228	0.1152	0.0122	0.0132	0.0134	0.0205	0.0229	0.0155	0.0137	0.0136	0.0225	0.0132	0.0147	0.0134	0.0158	0.0137	0.0230	0.0134	0.0138	0.0153					
0.0165	0.0163	0.0104	0.0103	0.0161	0.0102	0.0745	0.0152	0.0151	0.0248	0.0179	0.0155	0.0133	0.0120	0.0154	0.0165	0.0194	0.0098	0.0379	0.0134	0.0147	0.0186	0.0138	0.0229	0.1167	0.0133	0.0148	0.0105	0.0205	0.0204	0.0178	0.0107	0.0125	0.0224	0.0101	0.0170	0.0124	0.0166	0.0126	0.0248	0.0104	0.0134	0.0179	0.0076				
0.0181	0.0179	0.0090	0.0144	0.0165	0.0105	0.0754	0.0171	0.0155	0.0276	0.0195	0.0187	0.0168	0.0143	0.0168	0.0169	0.0213	0.0095	0.0394	0.0139	0.0164	0.0208	0.0173	0.0222	0.1197	0.0160	0.0167	0.0082	0.0199	0.0201	0.0196	0.0083	0.0126	0.0220	0.0024	0.0192	0.0125	0.0171	0.0127	0.0259	0.0025	0.0140	0.0210	0.0134	0.0102			
0.0106	0.0105	0.0170	0.0166	0.0135	0.0133	0.0750	0.0105	0.0177	0.0273	0.0084	0.0102	0.0141	0.0120	0.0090	0.0188	0.0108	0.0152	0.0353	0.0167	0.0089	0.0134	0.0159	0.0158	0.1085	0.0070	0.0094	0.0163	0.0240	0.0183	0.0124	0.0165	0.0139	0.0155	0.0160	0.0118	0.0137	0.0203	0.0139	0.0192	0.0163	0.0170	0.0215	0.0126	0.0135	0.0162		
0.0150	0.0148	0.0123	0.0135	0.0192	0.0072	0.0716	0.0145	0.0124	0.0254	0.0161	0.0186	0.0132	0.0109	0.0141	0.0139	0.0178	0.0123	0.0369	0.0104	0.0140	0.0179	0.0168	0.0235	0.1152	0.0126	0.0141	0.0110	0.0169	0.0231	0.0162	0.0110	0.0074	0.0232	0.0092	0.0161	0.0075	0.0141	0.0076	0.0225	0.0095	0.0104	0.0182	0.0104	0.0097	0.0094	0.0126	
0.0146	0.0145	0.0146	0.0117	0.0180	0.0102	0.0734	0.0135	0.0147	0.0232	0.0164	0.0181	0.0111	0.0096	0.0136	0.0162	0.0181	0.0103	0.0390	0.0138	0.0134	0.0167	0.0159	0.0227	0.1150	0.0124	0.0134	0.0135	0.0205	0.0227	0.0157	0.0135	0.0134	0.0225	0.0132	0.0148	0.0134	0.0159	0.0135	0.0229	0.0132	0.0139	0.0152	0.0025	0.0075	0.0134	0.0128	0.0102
0.0182	0.0180	0.0132	0.0155	0.0208	0.0103	0.0724	0.0177	0.0136	0.0252	0.0142	0.0181	0.0133	0.0090	0.0163	0.0168	0.0175	0.0155	0.0370	0.0132	0.0160	0.0210	0.0194	0.0216	0.1157	0.0155	0.0170	0.0121	0.0201	0.0249	0.0195	0.0121	0.0038	0.0212	0.0121	0.0191	0.0039	0.0170	0.0039	0.0255	0.0124	0.0134	0.0205	0.0130	0.0117	0.0124	0.0135	0.0075
0.0217	0.0214	0.0187	0.0210	0.0267	0.0138	0.0790	0.0210	0.0184	0.0327	0.0240	0.0195	0.0161	0.0190	0.0207	0.0207	0.0158	0.0196	0.0449	0.0175	0.0204	0.0160	0.0241	0.0171	0.1240	0.0193	0.0201	0.0183	0.0097	0.0165	0.0226	0.0182	0.0166	0.0168	0.0162	0.0213	0.0165	0.0064	0.0167	0.0150	0.0161	0.0177	0.0115	0.0161	0.0169	0.0164	0.0206	0.0137
0.0144	0.0143	0.0145	0.0120	0.0181	0.0103	0.0732	0.0134	0.0145	0.0233	0.0163	0.0182	0.0113	0.0098	0.0138	0.0160	0.0181	0.0105	0.0388	0.0136	0.0133	0.0169	0.0162	0.0230	0.1146	0.0122	0.0133	0.0133	0.0205	0.0231	0.0156	0.0136	0.0137	0.0225	0.0132	0.0146	0.0134	0.0160	0.0138	0.0231	0.0135	0.0138	0.0154	0.0023	0.0076	0.0134	0.0127	0.0104
0.0177	0.0176	0.0143	0.0175	0.0220	0.0119	0.0713	0.0181	0.0082	0.0262	0.0187	0.0206	0.0160	0.0143	0.0162	0.0152	0.0176	0.0168	0.0398	0.0114	0.0166	0.0202	0.0208	0.0252	0.1172	0.0154	0.0170	0.0127	0.0214	0.0266	0.0177	0.0130	0.0126	0.0250	0.0137	0.0174	0.0122	0.0183	0.0126	0.0268	0.0140	0.0117	0.0207	0.0145	0.0146	0.0141	0.0162	0.0109
0.0358	0.0357	0.0374	0.0364	0.0394	0.0331	0.0959	0.0359	0.0379	0.0491	0.0383	0.0326	0.0329	0.0361	0.0345	0.0394	0.0302	0.0364	0.0614	0.0373	0.0343	0.0316	0.0387	0.0305	0.1393	0.0335	0.0342	0.0377	0.0301	0.0300	0.0377	0.0380	0.0373	0.0302	0.0365	0.0366	0.0371	0.0272	0.0374	0.0304	0.0369	0.0376	0.0277	0.0336	0.0349	0.0370	0.0344	0.0340
0.0180	0.0178	0.0089	0.0143	0.0166	0.0106	0.0754	0.0170	0.0154	0.0276	0.0196	0.0187	0.0167	0.0143	0.0168	0.0170	0.0212	0.0094	0.0394	0.0137	0.0164	0.0208	0.0174	0.0221	0.1201	0.0162	0.0166	0.0081	0.0198	0.0200	0.0196	0.0083	0.0125	0.0219	0.0023	0.0191	0.0122	0.0171	0.0127	0.0259	0.0024	0.0139	0.0210	0.0133	0.0102	0.0024	0.0161	0.0094
0.0221	0.0220	0.0222	0.0241	0.0300	0.0172	0.0793	0.0209	0.0222	0.0356	0.0229	0.0231	0.0192	0.0223	0.0246	0.0237	0.0197	0.0232	0.0484	0.0204	0.0239	0.0208	0.0278	0.0203	0.1231	0.0231	0.0240	0.0216	0.0027	0.0198	0.0267	0.0218	0.0202	0.0201	0.0195	0.0255	0.0201	0.0100	0.0204	0.0096	0.0198	0.0207	0.0151	0.0204	0.0205	0.0197	0.0239	0.0168
0.0091	0.0090	0.0169	0.0156	0.0132	0.0122	0.0755	0.0093	0.0171	0.0276	0.0115	0.0130	0.0161	0.0153	0.0094	0.0179	0.0129	0.0152	0.0377	0.0153	0.0091	0.0135	0.0158	0.0179	0.1086	0.0070	0.0089	0.0168	0.0230	0.0173	0.0104	0.0171	0.0157	0.0176	0.0156	0.0114	0.0153	0.0191	0.0157	0.0181	0.0159	0.0156	0.0211	0.0120	0.0141	0.0159	0.0074	0.0121
0.0180	0.0179	0.0090	0.0147	0.0170	0.0108	0.0758	0.0172	0.0149	0.0279	0.0198	0.0190	0.0171	0.0146	0.0172	0.0171	0.0213	0.0098	0.0397	0.0132	0.0168	0.0212	0.0177	0.0224	0.1201	0.0164	0.0170	0.0084	0.0201	0.0204	0.0190	0.0088	0.0131	0.0222	0.0028	0.0195	0.0129	0.0174	0.0132	0.0263	0.0029	0.0135	0.0215	0.0137	0.0107	0.0029	0.0163	0.0099
0.0118	0.0116	0.0159	0.0163	0.0132	0.0151	0.0774	0.0117	0.0189	0.0282	0.0092	0.0107	0.0163	0.0124	0.0107	0.0207	0.0129	0.0140	0.0368	0.0173	0.0105	0.0157	0.0158	0.0159	0.1116	0.0100	0.0114	0.0151	0.0251	0.0176	0.0133	0.0154	0.0159	0.0156	0.0140	0.0135	0.0154	0.0224	0.0159	0.0203	0.0143	0.0176	0.0233	0.0141	0.0133	0.0143	0.0075	0.0139
0.0097	0.0096	0.0167	0.0168	0.0138	0.0125	0.0753	0.0091	0.0175	0.0276	0.0106	0.0133	0.0164	0.0137	0.0085	0.0177	0.0132	0.0153	0.0378	0.0147	0.0081	0.0129	0.0160	0.0181	0.1096	0.0066	0.0085	0.0164	0.0227	0.0179	0.0108	0.0167	0.0161	0.0176	0.0155	0.0107	0.0157	0.0198	0.0160	0.0175	0.0158	0.0150	0.0211	0.0121	0.0141	0.0158	0.0077	0.0127
0.0105	0.0105	0.0154	0.0174	0.0133	0.0135	0.0750	0.0102	0.0181	0.0284	0.0121	0.0128	0.0170	0.0157	0.0085	0.0185	0.0125	0.0163	0.0391	0.0156	0.0091	0.0139	0.0166	0.0189	0.1092	0.0053	0.0090	0.0159	0.0244	0.0183	0.0119	0.0161	0.0173	0.0186	0.0166	0.0115	0.0170	0.0208	0.0175	0.0192	0.0167	0.0157	0.0209	0.0128	0.0143	0.0168	0.0083	0.0142
0.0155	0.0154	0.0118	0.0140	0.0194	0.0084	0.0723	0.0140	0.0121	0.0254	0.0170	0.0189	0.0129	0.0117	0.0140	0.0145	0.0181	0.0132	0.0376	0.0093	0.0133	0.0183	0.0178	0.0245	0.1156	0.0131	0.0138	0.0104	0.0176	0.0236	0.0158	0.0109	0.0097	0.0241	0.0101	0.0164	0.0092	0.0150	0.0097	0.0235	0.0103	0.0096	0.0185	0.0105	0.0093	0.0103	0.0134	0.0053
0.0180	0.0180	0.0092	0.0149	0.0172	0.0110	0.0759	0.0173	0.0151	0.0278	0.0198	0.0190	0.0173	0.0147	0.0172	0.0172	0.0212	0.0099	0.0396	0.0135	0.0168	0.0214	0.0178	0.0226	0.1201	0.0164	0.0171	0.0085	0.0203	0.0207	0.0192	0.0088	0.0132	0.0224	0.0027	0.0196	0.0128	0.0175	0.0132	0.0264	0.0031	0.0136	0.0216	0.0138	0.0108	0.0030	0.0164	0.0099
0.0150	0.0149	0.0115	0.0140	0.0184	0.0075	0.0727	0.0148	0.0121	0.0243	0.0169	0.0191	0.0144	0.0116	0.0136	0.0128	0.0186	0.0133	0.0376	0.0098	0.0130	0.0187	0.0173	0.0237	0.1154	0.0133	0.0142	0.0113	0.0178	0.0232	0.0155	0.0118	0.0115	0.0233	0.0104	0.0166	0.0111	0.0152	0.0115	0.0238	0.0108	0.0101	0.0190	0.0101	0.0103	0.0106	0.0135	0.0076
0.0343	0.0345	0.0385	0.0404	0.0356	0.0382	0.1016	0.0341	0.0385	0.0539	0.0372	0.0377	0.0411	0.0403	0.0336	0.0440	0.0413	0.0381	0.0412	0.0355	0.0335	0.0360	0.0384	0.0426	0.1341	0.0326	0.0333	0.0383	0.0489	0.0405	0.0309	0.0388	0.0412	0.0421	0.0379	0.0316	0.0407	0.0458	0.0411	0.0438	0.0379	0.0358	0.0458	0.0380	0.0380	0.0380	0.0335	0.0381
0.0165	0.0164	0.0171	0.0189	0.0246	0.0122	0.0724	0.0151	0.0149	0.0288	0.0177	0.0235	0.0186	0.0169	0.0193	0.0171	0.0228	0.0176	0.0426	0.0134	0.0187	0.0213	0.0222	0.0279	0.1164	0.0176	0.0185	0.0163	0.0119	0.0280	0.0202	0.0167	0.0151	0.0277	0.0142	0.0186	0.0147	0.0168	0.0152	0.0168	0.0145	0.0137	0.0225	0.0148	0.0151	0.0144	0.0185	0.0114
0.0162	0.0161	0.0130	0.0152	0.0206	0.0086	0.0720	0.0157	0.0105	0.0247	0.0176	0.0192	0.0150	0.0127	0.0150	0.0133	0.0181	0.0139	0.0370	0.0112	0.0146	0.0187	0.0181	0.0235	0.1154	0.0141	0.0152	0.0125	0.0176	0.0240	0.0170	0.0129	0.0108	0.0232	0.0107	0.0169	0.0104	0.0147	0.0109	0.0236	0.0110	0.0115	0.0188	0.0114	0.0116	0.0109	0.0143	0.0083
0.0195	0.0192	0.0162	0.0186	0.0239	0.0121	0.0757	0.0185	0.0135	0.0287	0.0203	0.0177	0.0158	0.0157	0.0182	0.0175	0.0166	0.0170	0.0403	0.0144	0.0177	0.0165	0.0219	0.0204	0.1192	0.0178	0.0183	0.0157	0.0147	0.0211	0.0198	0.0161	0.0145	0.0202	0.0144	0.0200	0.0140	0.0109	0.0144	0.0201	0.0147	0.0148	0.0154	0.0140	0.0146	0.0146	0.0176	0.0117
0.0224	0.0223	0.0191	0.0211	0.0269	0.0154	0.0791	0.0212	0.0185	0.0321	0.0240	0.0198	0.0153	0.0191	0.0208	0.0209	0.0156	0.0199	0.0449	0.0164	0.0200	0.0178	0.0246	0.0172	0.1238	0.0203	0.0206	0.0181	0.0104	0.0167	0.0228	0.0185	0.0167	0.0171	0.0158	0.0228	0.0164	0.0082	0.0168	0.0166	0.0161	0.0168	0.0118	0.0181	0.0170	0.0161	0.0205	0.0124
0.0165	0.0164	0.0103	0.0130	0.0184	0.0083	0.0730	0.0154	0.0132	0.0251	0.0181	0.0183	0.0140	0.0130	0.0147	0.0147	0.0191	0.0142	0.0381	0.0107	0.0140	0.0194	0.0175	0.0249	0.1162	0.0138	0.0147	0.0119	0.0191	0.0227	0.0167	0.0123	0.0100	0.0244	0.0114	0.0176	0.0098	0.0163	0.0100	0.0251	0.0117	0.0108	0.0196	0.0117	0.0095	0.0117	0.0140	0.0073
0.0218	0.0218	0.0193	0.0218	0.0275	0.0147	0.0796	0.0210	0.0182	0.0326	0.0244	0.0198	0.0169	0.0198	0.0215	0.0212	0.0157	0.0202	0.0454	0.0173	0.0210	0.0169	0.0250	0.0175	0.1235	0.0199	0.0206	0.0189	0.0100	0.0172	0.0225	0.0193	0.0176	0.0174	0.0168	0.0220	0.0172	0.0068	0.0175	0.0156	0.0172	0.0176	0.0121	0.0167	0.0176	0.0171	0.0210	0.0146
0.0155	0.0155	0.0159	0.0177	0.0237	0.0122	0.0717	0.0189	0.0130	0.0298	0.0196	0.0236	0.0191	0.0180	0.0189	0.0169	0.0239	0.0188	0.0429	0.0109	0.0184	0.0212	0.0200	0.0297	0.1130	0.0173	0.0185	0.0159	0.0199	0.0283	0.0158	0.0163	0.0155	0.0295	0.0155	0.0173	0.0151	0.0203	0.0155	0.0254	0.0158	0.0110	0.0223	0.0158	0.0158	0.0157	0.0189	0.0121
0.0327	0.0330	0.0312	0.0327	0.0323	0.0317	0.0942	0.0343	0.0342	0.0484	0.0351	0.0349	0.0348	0.0343	0.0321	0.0372	0.0393	0.0319	0.0531	0.0314	0.0320	0.0354	0.0344	0.0406	0.1391	0.0308	0.0318	0.0331	0.0422	0.0367	0.0319	0.0335	0.0338	0.0409	0.0318	0.0324	0.0336	0.0384	0.0339	0.0417	0.0324	0.0319	0.0391	0.0322	0.0317	0.0316	0.0315	0.0315
0.0384	0.0383	0.0362	0.0377	0.0388	0.0373	0.0916	0.0424	0.0402	0.0510	0.0410	0.0378	0.0378	0.0396	0.0414	0.0424	0.0383	0.0354	0.0644	0.0387	0.0408	0.0396	0.0415	0.0373	0.1440	0.0402	0.0399	0.0369	0.0349	0.0350	0.0425	0.0371	0.0401	0.0369	0.0353	0.0418	0.0398	0.0347	0.0403	0.0375	0.0356	0.0391	0.0346	0.0375	0.0364	0.0355	0.0409	0.0377
0.0241	0.0241	0.0295	0.0316	0.0268	0.0303	0.0855	0.0229	0.0300	0.0435	0.0214	0.0259	0.0313	0.0288	0.0260	0.0354	0.0272	0.0284	0.0516	0.0297	0.0257	0.0277	0.0301	0.0283	0.1179	0.0250	0.0246	0.0283	0.0324	0.0303	0.0255	0.0283	0.0293	0.0278	0.0287	0.0257	0.0289	0.0360	0.0293	0.0274	0.0291	0.0301	0.0371	0.0297	0.0292	0.0289	0.0240	0.0306
0.0394	0.0391	0.0331	0.0375	0.0412	0.0324	0.0986	0.0396	0.0330	0.0515	0.0425	0.0426	0.0382	0.0376	0.0390	0.0393	0.0459	0.0352	0.0411	0.0290	0.0387	0.0411	0.0392	0.0476	0.1402	0.0376	0.0381	0.0329	0.0431	0.0457	0.0361	0.0335	0.0359	0.0473	0.0326	0.0365	0.0357	0.0402	0.0360	0.0488	0.0327	0.0291	0.0423	0.0362	0.0342	0.0327	0.0389	0.0325
0.0482	0.0484	0.0533	0.0546	0.0482	0.0511	0.0436	0.0477	0.0536	0.0629	0.0505	0.0511	0.0553	0.0535	0.0463	0.0535	0.0515	0.0544	0.0469	0.0524	0.0469	0.0503	0.0524	0.0561	0.1516	0.0449	0.0476	0.0540	0.0614	0.0553	0.0493	0.0544	0.0554	0.0558	0.0539	0.0473	0.0551	0.0586	0.0554	0.0567	0.0542	0.0526	0.0596	0.0514	0.0532	0.0541	0.0466	0.0515
0.0347	0.0348	0.0278	0.0328	0.0354	0.0291	0.0849	0.0366	0.0284	0.0426	0.0343	0.0313	0.0314	0.0318	0.0348	0.0334	0.0294	0.0300	0.0570	0.0286	0.0345	0.0331	0.0348	0.0293	0.1296	0.0345	0.0347	0.0277	0.0283	0.0299	0.0344	0.0277	0.0301	0.0292	0.0272	0.0341	0.0298	0.0269	0.0302	0.0336	0.0275	0.0287	0.0286	0.0322	0.0297	0.0275	0.0342	0.0295
0.0250	0.0250	0.0210	0.0232	0.0262	0.0222	0.0750	0.0263	0.0240	0.0337	0.0247	0.0267	0.0244	0.0222	0.0252	0.0275	0.0279	0.0201	0.0483	0.0237	0.0247	0.0294	0.0261	0.0295	0.1207	0.0246	0.0251	0.0204	0.0309	0.0298	0.0266	0.0206	0.0227	0.0293	0.0205	0.0273	0.0225	0.0293	0.0229	0.0335	0.0207	0.0239	0.0290	0.0227	0.0214	0.0207	0.0244	0.0223
0.1389	0.1386	0.1434	0.1450	0.1402	0.1440	0.1741	0.1466	0.1465	0.1349	0.1378	0.1414	0.1462	0.1437	0.1372	0.1544	0.1542	0.1438	0.1454	0.1464	0.1377	0.1437	0.1447	0.1456	0.1836	0.1374	0.1398	0.1423	0.1540	0.1465	0.1398	0.1427	0.1452	0.1454	0.1442	0.1409	0.1446	0.1522	0.1450	0.1482	0.1439	0.1467	0.1523	0.1426	0.1435	0.1441	0.1376	0.1441
0.0395	0.0393	0.0278	0.0319	0.0355	0.0308	0.0652	0.0396	0.0329	0.0191	0.0388	0.0373	0.0368	0.0329	0.0373	0.0325	0.0408	0.0306	0.0589	0.0330	0.0368	0.0416	0.0367	0.0404	0.1012	0.0371	0.0387	0.0282	0.0405	0.0395	0.0395	0.0286	0.0307	0.0405	0.0278	0.0387	0.0304	0.0385	0.0307	0.0472	0.0279	0.0330	0.0417	0.0347	0.0308	0.0279	0.0363	0.0308
0.0128	0.0126	0.0178	0.0179	0.0141	0.0154	0.0748	0.0123	0.0192	0.0262	0.0142	0.0153	0.0191	0.0173	0.0098	0.0179	0.0150	0.0181	0.0394	0.0172	0.0103	0.0157	0.0177	0.0206	0.1075	0.0090	0.0113	0.0182	0.0259	0.0199	0.0127	0.0184	0.0191	0.0204	0.0186	0.0134	0.0187	0.0229	0.0191	0.0211	0.0189	0.0175	0.0236	0.0155	0.0170	0.0190	0.0105	0.0153
0.0259	0.0260	0.0219	0.0232	0.0293	0.0179	0.0811	0.0229	0.0208	0.0346	0.0279	0.0288	0.0245	0.0235	0.0254	0.0221	0.0300	0.0233	0.0471	0.0205	0.0247	0.0282	0.0271	0.0336	0.1227	0.0244	0.0246	0.0226	0.0262	0.0340	0.0261	0.0229	0.0213	0.0333	0.0212	0.0264	0.0210	0.0246	0.0214	0.0321	0.0214	0.0209	0.0286	0.0207	0.0212	0.0214	0.0240	0.0183
0.0374	0.0373	0.0321	0.0363	0.0398	0.0333	0.0955	0.0413	0.0390	0.0504	0.0409	0.0431	0.0405	0.0395	0.0412	0.0392	0.0474	0.0361	0.0411	0.0366	0.0406	0.0454	0.0409	0.0477	0.1420	0.0395	0.0412	0.0347	0.0415	0.0451	0.0419	0.0352	0.0363	0.0475	0.0337	0.0428	0.0359	0.0415	0.0362	0.0478	0.0338	0.0367	0.0452	0.0366	0.0348	0.0339	0.0399	0.0343
0.0363	0.0360	0.0331	0.0280	0.0381	0.0301	0.0632	0.0363	0.0341	0.0134	0.0350	0.0366	0.0303	0.0272	0.0345	0.0337	0.0378	0.0304	0.0570	0.0337	0.0339	0.0399	0.0358	0.0414	0.0939	0.0336	0.0352	0.0342	0.0411	0.0428	0.0368	0.0347	0.0303	0.0415	0.0342	0.0375	0.0301	0.0380	0.0304	0.0449	0.0344	0.0338	0.0359	0.0284	0.0303	0.0343	0.0327	0.0305
0.0149	0.0149	0.0158	0.0180	0.0237	0.0115	0.0718	0.0191	0.0125	0.0299	0.0192	0.0233	0.0186	0.0177	0.0190	0.0172	0.0232	0.0185	0.0426	0.0103	0.0185	0.0210	0.0198	0.0293	0.1123	0.0170	0.0185	0.0160	0.0193	0.0282	0.0158	0.0164	0.0154	0.0290	0.0155	0.0168	0.0149	0.0196	0.0153	0.0249	0.0159	0.0106	0.0225	0.0159	0.0158	0.0157	0.0185	0.0119
0.0112	0.0112	0.0144	0.0162	0.0114	0.0134	0.0758	0.0110	0.0188	0.0283	0.0136	0.0131	0.0181	0.0172	0.0092	0.0196	0.0141	0.0176	0.0399	0.0171	0.0097	0.0153	0.0169	0.0197	0.1106	0.0077	0.0101	0.0176	0.0256	0.0173	0.0123	0.0179	0.0173	0.0193	0.0182	0.0131	0.0170	0.0221	0.0173	0.0206	0.0186	0.0174	0.0224	0.0145	0.0153	0.0185	0.0093	0.0150
0.0165	0.0164	0.0139	0.0102	0.0189	0.0112	0.0724	0.0166	0.0146	0.0198	0.0188	0.0186	0.0127	0.0114	0.0160	0.0147	0.0194	0.0117	0.0402	0.0140	0.0155	0.0193	0.0165	0.0240	0.1159	0.0148	0.0161	0.0158	0.0216	0.0226	0.0182	0.0162	0.0142	0.0237	0.0144	0.0166	0.0138	0.0182	0.0141	0.0253	0.0147	0.0142	0.0167	0.0091	0.0112	0.0146	0.0150	0.0125
0.0182	0.0182	0.0138	0.0164	0.0209	0.0103	0.0734	0.0178	0.0094	0.0262	0.0201	0.0211	0.0169	0.0159	0.0172	0.0133	0.0206	0.0160	0.0411	0.0078	0.0167	0.0182	0.0177	0.0255	0.1121	0.0159	0.0169	0.0151	0.0210	0.0251	0.0148	0.0155	0.0148	0.0252	0.0135	0.0136	0.0144	0.0172	0.0147	0.0263	0.0139	0.0081	0.0197	0.0137	0.0138	0.0139	0.0163	0.0111
0.0148	0.0147	0.0143	0.0125	0.0193	0.0132	0.0709	0.0185	0.0185	0.0245	0.0180	0.0209	0.0145	0.0141	0.0166	0.0185	0.0217	0.0145	0.0431	0.0164	0.0170	0.0228	0.0186	0.0275	0.1151	0.0155	0.0179	0.0165	0.0219	0.0255	0.0198	0.0169	0.0168	0.0274	0.0175	0.0204	0.0165	0.0215	0.0169	0.0249	0.0179	0.0165	0.0194	0.0120	0.0138	0.0179	0.0172	0.0150
0.0167	0.0166	0.0124	0.0111	0.0181	0.0100	0.0717	0.0156	0.0145	0.0226	0.0192	0.0190	0.0141	0.0124	0.0152	0.0149	0.0196	0.0134	0.0388	0.0129	0.0149	0.0199	0.0161	0.0255	0.1145	0.0138	0.0154	0.0136	0.0207	0.0234	0.0166	0.0141	0.0122	0.0253	0.0132	0.0177	0.0120	0.0177	0.0123	0.0253	0.0136	0.0131	0.0185	0.0107	0.0105	0.0135	0.0147	0.0096
0.0184	0.0183	0.0064	0.0120	0.0151	0.0098	0.0750	0.0181	0.0160	0.0277	0.0209	0.0166	0.0170	0.0167	0.0175	0.0170	0.0223	0.0114	0.0404	0.0138	0.0168	0.0219	0.0170	0.0231	0.1215	0.0163	0.0174	0.0098	0.0219	0.0173	0.0197	0.0101	0.0131	0.0230	0.0094	0.0200	0.0132	0.0189	0.0136	0.0274	0.0097	0.0139	0.0221	0.0146	0.0098	0.0095	0.0168	0.0121
0.0191	0.0188	0.0057	0.0121	0.0155	0.0099	0.0764	0.0186	0.0163	0.0277	0.0215	0.0172	0.0174	0.0169	0.0180	0.0171	0.0225	0.0119	0.0409	0.0141	0.0173	0.0229	0.0176	0.0243	0.1191	0.0166	0.0181	0.0096	0.0221	0.0184	0.0207	0.0099	0.0135	0.0240	0.0093	0.0209	0.0132	0.0192	0.0136	0.0283	0.0095	0.0142	0.0226	0.0151	0.0099	0.0095	0.0176	0.0123
0.0153	0.0153	0.0157	0.0181	0.0236	0.0121	0.0717	0.0188	0.0125	0.0299	0.0195	0.0235	0.0190	0.0179	0.0188	0.0168	0.0235	0.0187	0.0431	0.0105	0.0184	0.0211	0.0200	0.0298	0.1126	0.0171	0.0185	0.0159	0.0195	0.0280	0.0157	0.0162	0.0154	0.0295	0.0153	0.0171	0.0150	0.0203	0.0155	0.0252	0.0156	0.0107	0.0222	0.0158	0.0160	0.0156	0.0188	0.0121
0.0163	0.0162	0.0128	0.0111	0.0180	0.0103	0.0721	0.0153	0.0154	0.0230	0.0182	0.0188	0.0121	0.0112	0.0139	0.0164	0.0182	0.0110	0.0401	0.0136	0.0143	0.0193	0.0163	0.0241	0.1143	0.0122	0.0148	0.0146	0.0220	0.0236	0.0169	0.0149	0.0149	0.0238	0.0147	0.0172	0.0146	0.0184	0.0150	0.0249	0.0151	0.0137	0.0164	0.0092	0.0117	0.0151	0.0140	0.0113
0.0151	0.0151	0.0212	0.0199	0.0183	0.0172	0.0775	0.0147	0.0163	0.0300	0.0170	0.0178	0.0208	0.0200	0.0140	0.0199	0.0177	0.0205	0.0425	0.0145	0.0138	0.0151	0.0184	0.0220	0.1076	0.0123	0.0140	0.0219	0.0277	0.0222	0.0121	0.0221	0.0217	0.0218	0.0206	0.0101	0.0212	0.0240	0.0216	0.0233	0.0208	0.0148	0.0247	0.0176	0.0190	0.0208	0.0127	0.0177
0.0159	0.0157	0.0153	0.0184	0.0129	0.0177	0.0804	0.0153	0.0192	0.0338	0.0181	0.0159	0.0222	0.0217	0.0146	0.0221	0.0195	0.0156	0.0421	0.0167	0.0144	0.0173	0.0154	0.0204	0.1081	0.0128	0.0147	0.0169	0.0296	0.0176	0.0123	0.0171	0.0223	0.0201	0.0162	0.0132	0.0219	0.0255	0.0222	0.0250	0.0163	0.0171	0.0262	0.0184	0.0168	0.0163	0.0138	0.0189
0.0106	0.0106	0.0162	0.0169	0.0126	0.0135	0.0745	0.0103	0.0172	0.0275	0.0131	0.0138	0.0171	0.0156	0.0080	0.0183	0.0133	0.0168	0.0391	0.0152	0.0087	0.0139	0.0169	0.0190	0.1102	0.0066	0.0096	0.0161	0.0247	0.0185	0.0110	0.0164	0.0178	0.0186	0.0167	0.0111	0.0175	0.0215	0.0180	0.0198	0.0170	0.0155	0.0218	0.0134	0.0151	0.0170	0.0087	0.0139
0.0168	0.0167	0.0177	0.0193	0.0252	0.0136	0.0708	0.0201	0.0109	0.0290	0.0205	0.0244	0.0197	0.0190	0.0206	0.0180	0.0235	0.0196	0.0443	0.0101	0.0202	0.0203	0.0216	0.0298	0.1134	0.0183	0.0199	0.0175	0.0191	0.0288	0.0170	0.0179	0.0167	0.0296	0.0161	0.0149	0.0163	0.0194	0.0168	0.0252	0.0165	0.0102	0.0231	0.0163	0.0161	0.0164	0.0197	0.0130

0.0157	0.0157	0.0154	0.0188	0.0126	0.0177	0.0806	0.0152	0.0195	0.0331	0.0177	0.0156	0.0221	0.0212	0.0144	0.0222	0.0195	0.0157	0.0419	0.0169	0.0143	0.0174	0.0152	0.0202	0.1086	0.0133	0.0147	0.0162	0.0293	0.0176	0.0125	0.0164	0.0225	0.0198	0.0161	0.0134	0.0221	0.0254	0.0225	0.0248	0.0163	0.0172	0.0262	0.0178	0.0162	0.0162	0.0139	0.0194
0.0144	0.0144	0.0204	0.0199	0.0169	0.0163	0.0763	0.0135	0.0154	0.0300	0.0164	0.0164	0.0206	0.0204	0.0135	0.0189	0.0165	0.0206	0.0422	0.0145	0.0133	0.0142	0.0177	0.0219	0.1056	0.0111	0.0131	0.0217	0.0267	0.0205	0.0102	0.0219	0.0208	0.0217	0.0206	0.0095	0.0204	0.0234	0.0208	0.0219	0.0208	0.0150	0.0238	0.0171	0.0185	0.0207	0.0124	0.0170
0.0157	0.0156	0.0190	0.0186	0.0153	0.0177	0.0766	0.0147	0.0218	0.0286	0.0175	0.0173	0.0219	0.0202	0.0126	0.0203	0.0175	0.0212	0.0418	0.0200	0.0131	0.0183	0.0186	0.0234	0.1100	0.0111	0.0142	0.0205	0.0286	0.0213	0.0147	0.0207	0.0198	0.0234	0.0211	0.0158	0.0197	0.0252	0.0200	0.0235	0.0214	0.0202	0.0267	0.0183	0.0189	0.0214	0.0132	0.0179
0.0168	0.0167	0.0129	0.0147	0.0203	0.0089	0.0725	0.0169	0.0116	0.0244	0.0188	0.0203	0.0157	0.0149	0.0165	0.0082	0.0198	0.0152	0.0391	0.0109	0.0162	0.0194	0.0185	0.0245	0.1153	0.0154	0.0164	0.0142	0.0195	0.0246	0.0168	0.0146	0.0135	0.0242	0.0124	0.0163	0.0131	0.0161	0.0136	0.0254	0.0129	0.0112	0.0201	0.0125	0.0127	0.0128	0.0157	0.0102
0.1720	0.1720	0.1712	0.1708	0.1698	0.1709	0.1702	0.1723	0.1704	0.1669	0.1718	0.1714	0.1711	0.1698	0.1705	0.1747	0.1783	0.1698	0.1523	0.1699	0.1700	0.1713	0.1710	0.1739	0.2335	0.1701	0.1713	0.1694	0.1759	0.1751	0.1691	0.1702	0.1708	0.1740	0.1702	0.1701	0.1710	0.1751	0.1710	0.1762	0.1703	0.1702	0.1743	0.1702	0.1705	0.1703	0.1700	0.1699
0.0555	0.0554	0.0438	0.0509	0.0475	0.0485	0.1062	0.0565	0.0497	0.0661	0.0528	0.0530	0.0519	0.0479	0.0522	0.0569	0.0575	0.0475	0.0736	0.0503	0.0531	0.0574	0.0536	0.0558	0.1595	0.0522	0.0530	0.0429	0.0589	0.0568	0.0548	0.0431	0.0452	0.0558	0.0448	0.0556	0.0451	0.0549	0.0454	0.0640	0.0450	0.0506	0.0583	0.0507	0.0474	0.0450	0.0512	0.0478
0.0115	0.0114	0.0198	0.0188	0.0170	0.0155	0.0778	0.0109	0.0159	0.0307	0.0148	0.0161	0.0187	0.0179	0.0115	0.0201	0.0159	0.0181	0.0408	0.0129	0.0113	0.0130	0.0168	0.0217	0.1071	0.0097	0.0100	0.0192	0.0251	0.0213	0.0082	0.0196	0.0183	0.0213	0.0181	0.0090	0.0180	0.0223	0.0183	0.0199	0.0184	0.0132	0.0223	0.0153	0.0170	0.0185	0.0112	0.0151
0.0930	0.0932	0.0872	0.0908	0.0939	0.0872	0.1516	0.0965	0.0879	0.1062	0.0953	0.0963	0.0937	0.0926	0.0939	0.0955	0.1041	0.0896	0.0951	0.0855	0.0934	0.0956	0.0921	0.1023	0.1839	0.0924	0.0933	0.0868	0.0962	0.0991	0.0901	0.0875	0.0912	0.1020	0.0875	0.0916	0.0907	0.0953	0.0913	0.1016	0.0875	0.0858	0.0971	0.0905	0.0884	0.0875	0.0932	0.0877
0.0392	0.0390	0.0365	0.0357	0.0437	0.0358	0.0702	0.0418	0.0373	0.0297	0.0388	0.0412	0.0379	0.0352	0.0404	0.0387	0.0450	0.0365	0.0631	0.0373	0.0395	0.0443	0.0402	0.0460	0.1174	0.0402	0.0418	0.0361	0.0429	0.0465	0.0432	0.0365	0.0365	0.0459	0.0358	0.0418	0.0364	0.0436	0.0366	0.0475	0.0361	0.0377	0.0435	0.0367	0.0340	0.0361	0.0398	0.0363
0.0121	0.0121	0.0182	0.0181	0.0152	0.0141	0.0764	0.0113	0.0180	0.0290	0.0134	0.0142	0.0185	0.0168	0.0099	0.0187	0.0145	0.0173	0.0393	0.0176	0.0094	0.0155	0.0176	0.0196	0.1086	0.0091	0.0108	0.0187	0.0259	0.0194	0.0118	0.0189	0.0181	0.0193	0.0182	0.0132	0.0177	0.0221	0.0181	0.0210	0.0185	0.0180	0.0221	0.0144	0.0163	0.0185	0.0096	0.0154
0.3035	0.3050	0.3000	0.3002	0.3012	0.3010	0.3072	0.3097	0.3019	0.3068	0.3038	0.3007	0.3004	0.2998	0.3003	0.3163	0.3212	0.2994	0.2879	0.3007	0.3005	0.3006	0.2999	0.2997	0.4104	0.3015	0.3015	0.2995	0.3043	0.2995	0.3011	0.2993	0.2991	0.2995	0.2995	0.3006	0.2999	0.3008	0.3003	0.3034	0.2993	0.3014	0.3005	0.3004	0.3002	0.2998	0.3001	0.3007
0.0399	0.0400	0.0456	0.0446	0.0440	0.0414	0.1054	0.0401	0.0435	0.0560	0.0426	0.0433	0.0446	0.0436	0.0398	0.0406	0.0433	0.0438	0.0672	0.0420	0.0396	0.0420	0.0432	0.0489	0.0912	0.0379	0.0390	0.0454	0.0520	0.0484	0.0398	0.0458	0.0450	0.0489	0.0444	0.0399	0.0449	0.0486	0.0451	0.0493	0.0448	0.0419	0.0487	0.0408	0.0420	0.0446	0.0383	0.0414
0.0281	0.0283	0.0332	0.0330	0.0296	0.0307	0.0907	0.0280	0.0314	0.0444	0.0300	0.0297	0.0349	0.0337	0.0270	0.0341	0.0329	0.0328	0.0411	0.0304	0.0267	0.0280	0.0307	0.0349	0.1218	0.0253	0.0268	0.0340	0.0410	0.0337	0.0257	0.0344	0.0346	0.0347	0.0326	0.0248	0.0344	0.0380	0.0345	0.0359	0.0328	0.0306	0.0383	0.0312	0.0318	0.0327	0.0263	0.0308
0.0141	0.0139	0.0111	0.0141	0.0187	0.0099	0.0700	0.0178	0.0153	0.0267	0.0169	0.0202	0.0165	0.0158	0.0161	0.0165	0.0207	0.0161	0.0413	0.0133	0.0163	0.0219	0.0198	0.0272	0.1162	0.0150	0.0171	0.0131	0.0180	0.0246	0.0194	0.0135	0.0133	0.0267	0.0141	0.0195	0.0131	0.0183	0.0133	0.0238	0.0144	0.0133	0.0213	0.0136	0.0130	0.0144	0.0164	0.0109
0.0153	0.0151	0.0121	0.0121	0.0183	0.0103	0.0722	0.0157	0.0153	0.0231	0.0181	0.0183	0.0119	0.0108	0.0133	0.0169	0.0181	0.0117	0.0401	0.0137	0.0138	0.0197	0.0169	0.0245	0.1152	0.0122	0.0149	0.0137	0.0219	0.0234	0.0174	0.0141	0.0146	0.0241	0.0143	0.0176	0.0143	0.0186	0.0147	0.0252	0.0146	0.0139	0.0165	0.0086	0.0110	0.0145	0.0137	0.0116
0.0184	0.0183	0.0094	0.0094	0.0148	0.0124	0.0763	0.0182	0.0184	0.0245	0.0204	0.0180	0.0147	0.0129	0.0174	0.0192	0.0224	0.0073	0.0409	0.0165	0.0170	0.0218	0.0148	0.0228	0.1187	0.0158	0.0176	0.0120	0.0239	0.0187	0.0197	0.0122	0.0157	0.0225	0.0107	0.0197	0.0152	0.0208	0.0156	0.0269	0.0108	0.0166	0.0194	0.0116	0.0102	0.0109	0.0164	0.0136
0.0130	0.0128	0.0137	0.0161	0.0106	0.0155	0.0787	0.0123	0.0206	0.0303	0.0149	0.0134	0.0197	0.0179	0.0115	0.0209	0.0170	0.0126	0.0397	0.0188	0.0113	0.0162	0.0155	0.0170	0.1126	0.0102	0.0120	0.0143	0.0262	0.0142	0.0140	0.0144	0.0194	0.0169	0.0130	0.0142	0.0193	0.0233	0.0195	0.0212	0.0132	0.0191	0.0243	0.0158	0.0147	0.0131	0.0112	0.0159
0.0726	0.0726	0.0729	0.0770	0.0705	0.0776	0.1353	0.0763	0.0794	0.0937	0.0698	0.0717	0.0784	0.0749	0.0713	0.0868	0.0800	0.0749	0.0996	0.0798	0.0719	0.0769	0.0767	0.0752	0.1799	0.0718	0.0725	0.0730	0.0875	0.0757	0.0750	0.0731	0.0742	0.0750	0.0746	0.0748	0.0741	0.0848	0.0744	0.0820	0.0748	0.0802	0.0861	0.0773	0.0754	0.0748	0.0703	0.0780
0.0612	0.0608	0.0512	0.0581	0.0591	0.0547	0.1149	0.0641	0.0568	0.0737	0.0580	0.0602	0.0586	0.0550	0.0595	0.0627	0.0652	0.0551	0.0820	0.0570	0.0602	0.0657	0.0612	0.0631	0.1656	0.0596	0.0609	0.0497	0.0645	0.0646	0.0628	0.0499	0.0527	0.0628	0.0519	0.0633	0.0527	0.0624	0.0531	0.0700	0.0521	0.0571	0.0658	0.0580	0.0548	0.0521	0.0583	0.0552
0.0191	0.0188	0.0056	0.0116	0.0152	0.0096	0.0763	0.0185	0.0162	0.0279	0.0212	0.0169	0.0168	0.0165	0.0177	0.0167	0.0224	0.0113	0.0406	0.0140	0.0170	0.0225	0.0171	0.0240	0.1192	0.0163	0.0178	0.0092	0.0218	0.0179	0.0206	0.0093	0.0130	0.0236	0.0089	0.0205	0.0128	0.0187	0.0131	0.0278	0.0091	0.0141	0.0222	0.0147	0.0096	0.0090	0.0171	0.0118
0.0334	0.0336	0.0363	0.0385	0.0330	0.0357	0.0994	0.0329	0.0407	0.0508	0.0358	0.0354	0.0406	0.0387	0.0318	0.0419	0.0391	0.0364	0.0391	0.0387	0.0317	0.0369	0.0385	0.0400	0.1366	0.0309	0.0325	0.0369	0.0477	0.0381	0.0343	0.0374	0.0409	0.0398	0.0367	0.0346	0.0409	0.0443	0.0410	0.0428	0.0370	0.0390	0.0449	0.0368	0.0371	0.0369	0.0318	0.0375
0.0133	0.0133	0.0197	0.0189	0.0162	0.0153	0.0758	0.0127	0.0152	0.0291	0.0153	0.0156	0.0195	0.0190	0.0124	0.0190	0.0157	0.0192	0.0414	0.0134	0.0122	0.0133	0.0172	0.0207	0.1055	0.0100	0.0121	0.0201	0.0256	0.0195	0.0104	0.0202	0.0196	0.0206	0.0192	0.0085	0.0193	0.0222	0.0197	0.0208	0.0195	0.0137	0.0225	0.0157	0.0170	0.0193	0.0113	0.0159
0.0193	0.0189	0.0057	0.0120	0.0153	0.0096	0.0764	0.0183	0.0164	0.0277	0.0214	0.0171	0.0171	0.0165	0.0178	0.0167	0.0223	0.0116	0.0407	0.0142	0.0172	0.0227	0.0173	0.0243	0.1192	0.0165	0.0180	0.0094	0.0220	0.0181	0.0208	0.0095	0.0132	0.0238	0.0092	0.0207	0.0130	0.0190	0.0133	0.0279	0.0093	0.0142	0.0223	0.0149	0.0097	0.0093	0.0175	0.0119
0.0186	0.0184	0.0165	0.0130	0.0217	0.0125	0.0756	0.0183	0.0186	0.0225	0.0200	0.0215	0.0142	0.0125	0.0176	0.0174	0.0220	0.0129	0.0413	0.0164	0.0170	0.0223	0.0187	0.0262	0.1137	0.0166	0.0176	0.0178	0.0241	0.0260	0.0203	0.0182	0.0115	0.0258	0.0168	0.0203	0.0173	0.0207	0.0175	0.0275	0.0171	0.0164	0.0191	0.0113	0.0138	0.0171	0.0165	0.0140
0.0103	0.0103	0.0154	0.0152	0.0120	0.0121	0.0769	0.0106	0.0185	0.0273	0.0121	0.0131	0.0169	0.0157	0.0097	0.0181	0.0148	0.0161	0.0387	0.0162	0.0092	0.0144	0.0162	0.0181	0.1101	0.0083	0.0094	0.0182	0.0243	0.0165	0.0119	0.0184	0.0161	0.0179	0.0167	0.0124	0.0159	0.0208	0.0162	0.0198	0.0169	0.0165	0.0218	0.0132	0.0143	0.0169	0.0085	0.0142
0.0193	0.0190	0.0167	0.0133	0.0213	0.0143	0.0719	0.0187	0.0166	0.0193	0.0213	0.0215	0.0150	0.0132	0.0165	0.0175	0.0211	0.0145	0.0423	0.0155	0.0169	0.0211	0.0198	0.0269	0.1146	0.0157	0.0180	0.0163	0.0239	0.0263	0.0200	0.0167	0.0177	0.0267	0.0162	0.0179	0.0175	0.0211	0.0177	0.0275	0.0166	0.0155	0.0193	0.0126	0.0149	0.0167	0.0172	0.0145
0.0190	0.0188	0.0056	0.0118	0.0152	0.0097	0.0763	0.0184	0.0161	0.0277	0.0213	0.0169	0.0170	0.0165	0.0177	0.0168	0.0223	0.0114	0.0405	0.0140	0.0170	0.0224	0.0171	0.0240	0.1195	0.0164	0.0178	0.0092	0.0218	0.0179	0.0205	0.0095	0.0131	0.0236	0.0091	0.0205	0.0129	0.0187	0.0131	0.0280	0.0091	0.0141	0.0223	0.0147	0.0096	0.0091	0.0172	0.0118
0.0121	0.0119	0.0197	0.0198	0.0166	0.0158	0.0774	0.0110	0.0155	0.0299	0.0144	0.0164	0.0188	0.0174	0.0111	0.0203	0.0164	0.0180	0.0405	0.0129	0.0107	0.0127	0.0165	0.0216	0.1081	0.0105	0.0100	0.0190	0.0260	0.0207	0.0093	0.0193	0.0184	0.0210	0.0176	0.0088	0.0182	0.0228	0.0185	0.0208	0.0179	0.0131	0.0227	0.0156	0.0169	0.0179	0.0111	0.0151
0.0104	0.0103	0.0153	0.0149	0.0118	0.0130	0.0760	0.0104	0.0184	0.0269	0.0125	0.0127	0.0174	0.0161	0.0098	0.0169	0.0144	0.0160	0.0380	0.0168	0.0094	0.0146	0.0148	0.0181	0.1078	0.0083	0.0101	0.0176	0.0246	0.0169	0.0116	0.0178	0.0157	0.0178	0.0167	0.0123	0.0155	0.0206	0.0158	0.0196	0.0170	0.0171	0.0219	0.0126	0.0139	0.0169	0.0087	0.0139
0.0168	0.0167	0.0124	0.0101	0.0167	0.0107	0.0729	0.0162	0.0162	0.0222	0.0183	0.0186	0.0119	0.0105	0.0140	0.0166	0.0188	0.0114	0.0407	0.0140	0.0145	0.0200	0.0163	0.0245	0.1167	0.0130	0.0150	0.0144	0.0225	0.0227	0.0177	0.0147	0.0144	0.0242	0.0147	0.0173	0.0143	0.0191	0.0145	0.0258	0.0151	0.0139	0.0166	0.0096	0.0112	0.0150	0.0141	0.0119
0.0154	0.0152	0.0158	0.0181	0.0235	0.0120	0.0717	0.0187	0.0126	0.0300	0.0194	0.0235	0.0188	0.0179	0.0186	0.0169	0.0235	0.0186	0.0427	0.0106	0.0181	0.0209	0.0199	0.0296	0.1127	0.0171	0.0184	0.0158	0.0195	0.0279	0.0157	0.0161	0.0152	0.0292	0.0152	0.0170	0.0149	0.0201	0.0153	0.0250	0.0155	0.0107	0.0220	0.0157	0.0159	0.0153	0.0186	0.0120
0.0753	0.0756	0.0673	0.0661	0.0695	0.0697	0.0727	0.0750	0.0746	0.0797	0.0776	0.0758	0.0707	0.0697	0.0731	0.0744	0.0809	0.0662	0.0418	0.0720	0.0733	0.0784	0.0726	0.0817	0.1807	0.0713	0.0743	0.0690	0.0813	0.0785	0.0755	0.0694	0.0725	0.0815	0.0700	0.0758	0.0723	0.0773	0.0723	0.0843	0.0703	0.0719	0.0755	0.0681	0.0682	0.0700	0.0731	0.0700
0.0338	0.0338	0.0374	0.0353	0.0399	0.0350	0.0906	0.0381	0.0384	0.0482	0.0367	0.0398	0.0390	0.0382	0.0373	0.0389	0.0408	0.0383	0.0631	0.0377	0.0366	0.0404	0.0396	0.0457	0.1361	0.0356	0.0365	0.0390	0.0422	0.0440	0.0379	0.0395	0.0374	0.0454	0.0378	0.0374	0.0373	0.0421	0.0376	0.0427	0.0384	0.0379	0.0433	0.0351	0.0362	0.0382	0.0363	0.0359
0.0166	0.0165	0.0165	0.0185	0.0242	0.0123	0.0708	0.0196	0.0111	0.0289	0.0198	0.0230	0.0186	0.0184	0.0197	0.0169	0.0223	0.0186	0.0433	0.0095	0.0193	0.0199	0.0206	0.0287	0.1131	0.0177	0.0192	0.0170	0.0190	0.0275	0.0169	0.0172	0.0161	0.0285	0.0156	0.0148	0.0157	0.0187	0.0162	0.0246	0.0160	0.0096	0.0220	0.0154	0.0153	0.0159	0.0188	0.0126
0.0155	0.0152	0.0156	0.0177	0.0232	0.0118	0.0715	0.0188	0.0126	0.0297	0.0193	0.0232	0.0186	0.0177	0.0187	0.0169	0.0236	0.0183	0.0427	0.0106	0.0181	0.0209	0.0195	0.0294	0.1128	0.0170	0.0182	0.0156	0.0193	0.0277	0.0159	0.0159	0.0150	0.0291	0.0150	0.0169	0.0146	0.0198	0.0151	0.0250	0.0153	0.0106	0.0219	0.0157	0.0155	0.0153	0.0185	0.0118
0.0180	0.0179	0.0153	0.0126	0.0195	0.0127	0.0710	0.0174	0.0153	0.0216	0.0198	0.0204	0.0140	0.0125	0.0156	0.0162	0.0196	0.0136	0.0422	0.0142	0.0159	0.0198	0.0179	0.0258	0.1159	0.0142	0.0168	0.0159	0.0227	0.0247	0.0181	0.0163	0.0167	0.0256	0.0158	0.0166	0.0164	0.0203	0.0167	0.0261	0.0161	0.0142	0.0182	0.0115	0.0138	0.0159	0.0159	0.0131
0.0153	0.0150	0.0156	0.0177	0.0233	0.0118	0.0717	0.0187	0.0125	0.0297	0.0193	0.0232	0.0184	0.0176	0.0184	0.0167	0.0233	0.0183	0.0427	0.0104	0.0179	0.0207	0.0196	0.0293	0.1127	0.0168	0.0181	0.0157	0.0192	0.0276	0.0156	0.0159	0.0150	0.0289	0.0149	0.0168	0.0146	0.0198	0.0151	0.0248	0.0153	0.0105	0.0218	0.0154	0.0155	0.0151	0.0185	0.0117
0.0156	0.0153	0.0159	0.0180	0.0234	0.0121	0.0718	0.0188	0.0128	0.0299	0.0194	0.0234	0.0187	0.0178	0.0186	0.0167	0.0235	0.0185	0.0428	0.0108	0.0181	0.0210	0.0198	0.0296	0.1123	0.0170	0.0183	0.0158	0.0196	0.0277	0.0159	0.0161	0.0152	0.0292	0.0153	0.0169	0.0150	0.0202	0.0154	0.0250	0.0156	0.0108	0.0220	0.0157	0.0158	0.0154	0.0187	0.0121
0.0185	0.0185	0.0172	0.0129	0.0220	0.0137	0.0726	0.0181	0.0163	0.0185	0.0203	0.0214	0.0145	0.0126	0.0173	0.0165	0.0212	0.0136	0.0419	0.0152	0.0168	0.0204	0.0190	0.0260	0.1152	0.0165	0.0174	0.0173	0.0235	0.0257	0.0197	0.0176	0.0173	0.0257	0.0160	0.0177	0.0170	0.0205	0.0174	0.0268	0.0162	0.0153	0.0186	0.0119	0.0143	0.0160	0.0169	0.0140
0.0125	0.0123	0.0184	0.0184	0.0148	0.0143	0.0762	0.0116	0.0184	0.0292	0.0135	0.0148	0.0186	0.0167	0.0100	0.0187	0.0151	0.0173	0.0389	0.0181	0.0097	0.0157	0.0175	0.0193	0.1092	0.0095	0.0113	0.0187	0.0262	0.0195	0.0126	0.0189	0.0181	0.0190	0.0182	0.0133	0.0179	0.0223	0.0182	0.0211	0.0186	0.0183	0.0224	0.0142	0.0162	0.0185	0.0099	0.0152
0.0110	0.0108	0.0148	0.0140	0.0126	0.0128	0.0754	0.0105	0.0185	0.0270	0.0128	0.0119	0.0167	0.0163	0.0100	0.0181	0.0146	0.0171	0.0388	0.0166	0.0094	0.0145	0.0161	0.0195	0.1091	0.0084	0.0096	0.0177	0.0245	0.0163	0.0127	0.0178	0.0158	0.0192	0.0176	0.0125	0.0154	0.0210	0.0158	0.0196	0.0180	0.0168	0.0218	0.0138	0.0138	0.0178	0.0092	0.0144
0.0457	0.0458	0.0523	0.0516	0.0464	0.0484	0.0531	0.0464	0.0521	0.0618	0.0467	0.0487	0.0526	0.0496	0.0440	0.0522	0.0510	0.0512	0.0383	0.0511	0.0435	0.0495	0.0507	0.0529	0.1485	0.0439	0.0452	0.0520	0.0596	0.0539	0.0467	0.0522	0.0516	0.0530	0.0516	0.0472	0.0514	0.0563	0.0518	0.0545	0.0519	0.0513	0.0577	0.0484	0.0502	0.0519	0.0438	0.0487
0.0407	0.0405	0.0380	0.0345	0.0428	0.0357	0.0802	0.0409	0.0376	0.0261	0.0409	0.0420	0.0354	0.0340	0.0381	0.0350	0.0409	0.0358	0.0636	0.0370	0.0384	0.0427	0.0410	0.0472	0.0898	0.0370	0.0398	0.0380	0.0456	0.0485	0.0416	0.0386	0.0385	0.0472	0.0386	0.0402	0.0383	0.0426	0.0386	0.0491	0.0388	0.0373	0.0402	0.0334	0.0356	0.0389	0.0374	0.0362
0.0151	0.0150	0.0129	0.0103	0.0171	0.0100	0.0725	0.0151	0.0154	0.0220	0.0182	0.0185	0.0118	0.0103	0.0143	0.0163	0.0198	0.0113	0.0396	0.0132	0.0138	0.0191	0.0155	0.0241	0.1152	0.0133	0.0143	0.0142	0.0211	0.0222	0.0162	0.0147	0.0127	0.0238	0.0137	0.0169	0.0126	0.0178	0.0127	0.0240	0.0140	0.0133	0.0165	0.0083	0.0102	0.0138	0.0140	0.0105
0.0128	0.0127	0.0205	0.0203	0.0173	0.0165	0.0774	0.0123	0.0149	0.0305	0.0140	0.0165	0.0188	0.0171	0.0114	0.0213	0.0160	0.0184	0.0396	0.0138	0.0112	0.0140	0.0172	0.0214	0.1078	0.0111	0.0111	0.0194	0.0268	0.0216	0.0100	0.0196	0.0183	0.0209	0.0188	0.0100	0.0180	0.0236	0.0184	0.0219	0.0191	0.0140	0.0230	0.0162	0.0173	0.0189	0.0110	0.0155
0.0753	0.0754	0.0761	0.0708	0.0816	0.0722	0.1248	0.0769	0.0763	0.0650	0.0760	0.0802	0.0718	0.0703	0.0766	0.0640	0.0812	0.0718	0.1048	0.0747	0.0763	0.0815	0.0786	0.0856	0.0478	0.0750	0.0774	0.0763	0.0800	0.0873	0.0787	0.0768	0.0751	0.0857	0.0758	0.0788	0.0747	0.0806	0.0752	0.0838	0.0763	0.0746	0.0782	0.0691	0.0722	0.0757	0.0750	0.0728
0.1035	0.1035	0.1028	0.0992	0.1067	0.0999	0.1443	0.1054	0.1006	0.0946	0.1034	0.1049	0.1015	0.0988	0.1024	0.0962	0.1080	0.1005	0.1243	0.1008	0.1020	0.1045	0.1025	0.1094	0.0734	0.1014	0.1035	0.1020	0.1089	0.1098	0.1034	0.1029	0.1004	0.1099	0.1023	0.1021	0.1005	0.1081	0.1005	0.1110	0.1023	0.1009	0.1067	0.0991	0.0994	0.1022	0.1009	0.1008
0.0606	0.0606	0.0596	0.0564	0.0633	0.0569	0.1155	0.0618	0.0578	0.0659	0.0618	0.0619	0.0586	0.0570	0.0590	0.0549	0.0644	0.0577	0.0806	0.0568	0.0587	0.0612	0.0585	0.0673	0.0939	0.0583	0.0602	0.0595	0.0668	0.0674	0.0599	0.0603	0.0593	0.0673	0.0592	0.0585	0.0592	0.0645	0.0592	0.0693	0.0595	0.0568	0.0635	0.0560	0.0564	0.0592	0.0580	0.0573
0.0952	0.0955	0.0890	0.0904	0.0944	0.0868	0.0960	0.0955	0.0901	0.1035	0.0976	0.0967	0.0936	0.0922	0.0936	0.0836	0.1027	0.0927	0.0856	0.0884	0.0934	0.0968	0.0950	0.1024	0.1111	0.0929	0.0934	0.0904	0.0957	0.1001	0.0952	0.0909	0.0894	0.1029	0.0896	0.0935	0.0897	0.0938	0.0897	0.1018	0.0898	0.0884	0.0976	0.0898	0.0886	0.0898	0.0929	0.0873
0.0796	0.0800	0.0749	0.0793	0.0810	0.0751	0.0669	0.0846	0.0749	0.0936	0.0825	0.0845	0.0817	0.0809	0.0826	0.0800	0.0906	0.0777	0.0556	0.0727	0.0819	0.0831	0.0792	0.0888	0.1848	0.0808	0.0823	0.0760	0.0820	0.0863	0.0798	0.0766	0.0791	0.0889	0.0750	0.0777	0.0792	0.0823	0.0794	0.0879	0.0751	0.0726	0.0849	0.0789	0.0768	0.0749	0.0815	0.0755
0.0784	0.0782	0.0776	0.0793	0.0836	0.0734	0.0278	0.0830	0.0737	0.0702	0.0786	0.0837	0.0789	0.0772	0.0808	0.0780	0.0863	0.0802	0.0749	0.0726	0.0802	0.0837	0.0799	0.0881	0.1480	0.0795	0.0812	0.0776	0.0811	0.0900	0.0786	0.0783	0.0749	0.0879	0.0780	0.0791	0.0750	0.0818	0.0749	0.0872	0.0782	0.0725	0.0841	0.0777	0.0771	0.0780	0.0791	0.0737
0.0166	0.0166	0.0152	0.0108	0.0194	0.0112	0.0720	0.0157	0.0138	0.0197	0.0180	0.0186	0.0115	0.0103	0.0152	0.0152	0.0192	0.0110	0.0403	0.0127	0.0146	0.0183	0.0165	0.0234	0.1161	0.0142	0.0149	0.0152	0.0213	0.0228	0.0171	0.0156	0.0147	0.0233	0.0138	0.0155	0.0145	0.0176	0.0151	0.0247	0.0141	0.0129	0.0154	0.0087	0.0114	0.0140	0.0142	0.0114
0.0166	0.0166	0.0166	0.0184	0.0239	0.0124	0.0700	0.0204	0.0113	0.0292	0.0202	0.0236	0.0193	0.0186	0.0197	0.0167	0.0230	0.0192	0.0434	0.0097	0.0190	0.0202	0.0204	0.0283	0.1147	0.0180	0.0195	0.0170	0.0190	0.0272	0.0159	0.0174	0.0160	0.0285	0.0157	0.0149	0.0159	0.0197	0.0163	0.0250	0.0160	0.0100	0.0223	0.0163	0.0163	0.0158	0.0190	0.0126
0.0131	0.0130	0.0124	0.0158	0.0192	0.0102	0.0698	0.0174	0.0147	0.0273	0.0160	0.0205	0.0164	0.0149	0.0152	0.0157	0.0204	0.0159	0.0405	0.0122	0.0155	0.0210	0.0196	0.0263	0.1158	0.0143	0.0166	0.0127	0.0179	0.0257	0.0177	0.0130	0.0128	0.0260	0.0136	0.0185	0.0140	0.0180	0.0144	0.0234	0.0140	0.0122	0.0213	0.0132	0.0130	0.0139	0.0156	0.0108
0.0197	0.0194	0.0078	0.0149	0.0161	0.0121	0.0743	0.0186	0.0148	0.0281	0.0198	0.0186	0.0174	0.0145	0.0164	0.0190	0.0205	0.0114	0.0396	0.0139	0.0166	0.0222	0.0184	0.0225	0.1207	0.0159	0.0183	0.0024	0.0219	0.0212	0.0196	0.0026	0.0128	0.0226	0.0082	0.0198	0.0129	0.0192	0.0134	0.0278	0.0086	0.0141	0.0227	0.0137	0.0107	0.0084	0.0166	0.0113
0.0366	0.0366	0.0378	0.0364	0.0404	0.0339	0.0928	0.0365	0.0327	0.0482	0.0388	0.0391	0.0372	0.0365	0.0353	0.0388	0.0394	0.0367	0.0622	0.0323	0.0350	0.0363	0.0359	0.0441	0.1344	0.0338	0.0345	0.0373	0.0443	0.0438	0.0336	0.0377	0.0377	0.0438	0.0367	0.0317	0.0377	0.0405	0.0380	0.0448	0.0372	0.0323	0.0404	0.0340	0.0354	0.0372	0.0344	0.0344
0.0946	0.0948	0.0901	0.0933	0.0936	0.0915	0.0825	0.1019	0.0956	0.1128	0.0975	0.0956	0.0927	0.0940	0.0943	0.1023	0.1054	0.0913	0.0719	0.0928	0.0947	0.0959	0.0917	0.0961	0.2100	0.0937	0.0952	0.0901	0.0933	0.0941	0.0968	0.0909	0.0943	0.0965	0.0908	0.0960	0.0945	0.0920	0.0949	0.0962	0.0907	0.0930	0.0929	0.0920	0.0915	0.0908	0.0952	0.0914
0.0145	0.0143	0.0191	0.0202	0.0160	0.0167	0.0756	0.0141	0.0198	0.0292	0.0161	0.0167	0.0208	0.0196	0.0124	0.0181	0.0162	0.0199	0.0406	0.0189	0.0129	0.0166	0.0175	0.0215	0.1114	0.0113	0.0135	0.0201	0.0273	0.0211	0.0151	0.0204	0.0213	0.0213	0.0202	0.0137	0.0211	0.0239	0.0215	0.0227	0.0204	0.0190	0.0248	0.0170	0.0185	0.0204	0.0124	0.0178
0.0510	0.0508	0.0486	0.0500	0.0507	0.0485	0.1136	0.0524	0.0492	0.0656	0.0531	0.0524	0.0519	0.0514	0.0502	0.0567	0.0582	0.0477	0.0558	0.0475	0.0494	0.0523	0.0510	0.0566	0.1548	0.0489	0.0498	0.0486	0.0597	0.0552	0.0470	0.0493	0.0522	0.0569	0.0487	0.0480	0.0524	0.0558	0.0528	0.0599	0.0484	0.0476	0.0558	0.0483	0.0484	0.0486	0.0490	0.0486
0.0119	0.0117	0.0164	0.0168	0.0131	0.0133	0.0731	0.0113	0.0169	0.0268	0.0136	0.0138	0.0176	0.0168	0.0095	0.0161	0.0136	0.0169	0.0390	0.0152	0.0099	0.0135	0.0161	0.0187	0.1086	0.0079	0.0105	0.0172	0.0243	0.0181	0.0119	0.0174	0.0180	0.0185	0.0172	0.0104	0.0178	0.0204	0.0182	0.0196	0.0175	0.0155	0.0220	0.0140	0.0154	0.0174	0.0092	0.0144
0.0145	0.0144	0.0243	0.0239	0.0213	0.0192	0.0822	0.0148	0.0228	0.0352	0.0188	0.0145	0.0189	0.0223	0.0155	0.0245	0.0119	0.0220	0.0459	0.0211	0.0149	0.0112	0.0225	0.0121	0.1199	0.0139	0.0135	0.0234	0.0164	0.0111	0.0163	0.0237	0.0220	0.0122	0.0208	0.0161	0.0222	0.0130	0.0225	0.0114	0.0213	0.0216	0.0139	0.0186	0.0210	0.0211	0.0151	0.0193
0.1171	0.1172	0.1229	0.1253	0.1171	0.1227	0.1181	0.1272	0.1203	0.1444	0.1198	0.1211	0.1268	0.1266	0.1188	0.1347	0.1367	0.1230	0.0981	0.1199	0.1185	0.1192	0.1204	0.1267	0.1973	0.1171	0.1187	0.1234	0.1312	0.1241	0.1154	0.1240	0.1276	0.1270	0.1224	0.1137	0.1267	0.1297	0.1272	0.1258	0.1225	0.1198	0.1309	0.1222	0.1227	0.1225	0.1181	0.1235
0.0186	0.0184	0.0096	0.0090	0.0145	0.0129	0.0763	0.0184	0.0184	0.0245	0.0203	0.0181	0.0142	0.0130	0.0175	0.0190	0.0222	0.0069	0.0408	0.0165	0.0169	0.0220	0.0149	0.0220	0.1194	0.0161	0.0175	0.0118	0.0238	0.0181	0.0193	0.0119	0.0153	0.0219	0.0106	0.0197	0.0153	0.0206	0.0156	0.0271	0.0110	0.0166	0.0189	0.0112	0.0098	0.0107	0.0164	0.0132
0.0173	0.0172	0.0145	0.0172	0.0222	0.0103	0.0722	0.0176	0.0071	0.0253	0.0183	0.0202	0.0163	0.0132	0.0167	0.0128	0.0187	0.0159	0.0385	0.0113	0.0162	0.0203	0.0194	0.0245	0.1159	0.0164	0.0168	0.0131	0.0203	0.0259	0.0177	0.0134	0.0114	0.0242	0.0128	0.0175	0.0111	0.0172	0.0115	0.0262	0.0130	0.0115	0.0200	0.0134	0.0137	0.0131	0.0160	0.0101
0.0119	0.0119	0.0182	0.0183	0.0150	0.0141	0.0759	0.0114	0.0174	0.0285	0.0137	0.0144	0.0186	0.0170	0.0106	0.0179	0.0151	0.0174	0.0397	0.0172	0.0103	0.0148	0.0175	0.0195	0.1102	0.0095	0.0112	0.0192	0.0257	0.0194	0.0122	0.0195	0.0184	0.0193	0.0181	0.0125	0.0181	0.0219	0.0184	0.0207	0.0183	0.0175	0.0226	0.0143	0.0163	0.0183	0.0097	0.0152
0.0173	0.0174	0.0144	0.0170	0.0222	0.0103	0.0719	0.0176	0.0070	0.0251	0.0182	0.0201	0.0159	0.0131	0.0163	0.0127	0.0187	0.0159	0.0383	0.0112	0.0159	0.0199	0.0193	0.0242	0.1159	0.0162	0.0169	0.0130	0.0200	0.0256	0.0173	0.0133	0.0114	0.0240	0.0127	0.0171	0.0110	0.0171	0.0114	0.0260	0.0131	0.0116	0.0203	0.0134	0.0136	0.0130	0.0156	0.0100
0.0109	0.0108	0.0164	0.0165	0.0136	0.0139	0.0745	0.0103	0.0177	0.0278	0.0134	0.0139	0.0176	0.0159	0.0087	0.0189	0.0136	0.0167	0.0382	0.0154	0.0092	0.0139	0.0161	0.0192	0.1094	0.0074	0.0098	0.0162	0.0246	0.0189	0.0114	0.0165	0.0172	0.0191	0.0169	0.0115	0.0170	0.0213	0.0174	0.0197	0.0171	0.0157	0.0223	0.0141	0.0154	0.0171	0.0090	0.0136
0.0138	0.0137	0.0218	0.0212	0.0187	0.0173	0.0774	0.0129	0.0138	0.0310	0.0159	0.0178	0.0203	0.0195	0.0130	0.0207	0.0176	0.0203	0.0412	0.0138	0.0127	0.0145	0.0185	0.0229	0.1087	0.0119	0.0125	0.0213	0.0277	0.0227	0.0090	0.0216	0.0197	0.0227	0.0202	0.0096	0.0195	0.0245	0.0198	0.0228	0.0204	0.0141	0.0241	0.0173	0.0190	0.0205	0.0129	0.0173
0.0168	0.0168	0.0158	0.0176	0.0235	0.0135	0.0728	0.0201	0.0139	0.0297	0.0210	0.0242	0.0204	0.0194	0.0205	0.0181	0.0246	0.0200	0.0438	0.0119	0.0199	0.0226	0.0206	0.0311	0.1131	0.0187	0.0199	0.0173	0.0209	0.0280	0.0170	0.0178	0.0154	0.0308	0.0166	0.0187	0.0151	0.0215	0.0156	0.0267	0.0170	0.0119	0.0236	0.0173	0.0165	0.0169	0.0201	0.0133
0.0151	0.0150	0.0156	0.0179	0.0235	0.0118	0.0716	0.0187	0.0123	0.0296	0.0194	0.0234	0.0187	0.0178	0.0187	0.0167	0.0234	0.0186	0.0429	0.0103	0.0181	0.0208	0.0198	0.0295	0.1127	0.0170	0.0182	0.0158	0.0194	0.0279	0.0155	0.0161	0.0152	0.0292	0.0152	0.0169	0.0148	0.0199	0.0152	0.0251	0.0156	0.0104	0.0220	0.0156	0.0156	0.0152	0.0186	0.0119
0.0186	0.0185	0.0218	0.0226	0.0187	0.0209	0.0807	0.0181	0.0237	0.0335	0.0207	0.0157	0.0220	0.0240	0.0158	0.0250	0.0128	0.0241	0.0462	0.0233	0.0163	0.0165	0.0229	0.0163	0.1182	0.0150	0.0173	0.0240	0.0221	0.0144	0.0193	0.0242	0.0241	0.0161	0.0245	0.0184	0.0238	0.0185	0.0241	0.0174	0.0248	0.0234	0.0192	0.0209	0.0219	0.0247	0.0163	0.0215
0.0186	0.0186	0.0218	0.0228	0.0188	0.0209	0.0806	0.0181	0.0238	0.0336	0.0208	0.0158	0.0220	0.0240	0.0160	0.0249	0.0127	0.0241	0.0462	0.0232	0.0165	0.0166	0.0229	0.0164	0.1181	0.0151	0.0175	0.0240	0.0222	0.0144	0.0193	0.0242	0.0241	0.0162	0.0245	0.0186	0.0239	0.0185	0.0242	0.0175	0.0247	0.0235	0.0193	0.0210	0.0220	0.0249	0.0165	0.0216
0.0128	0.0126	0.0179	0.0181	0.0141	0.0155	0.0751	0.0123	0.0187	0.0268	0.0144	0.0154	0.0193	0.0178	0.0101	0.0182	0.0152	0.0186	0.0394	0.0173	0.0104	0.0158	0.0177	0.0208	0.1070	0.0089	0.0114	0.0184	0.0263	0.0200	0.0126	0.0185	0.0192	0.0204	0.0188	0.0134	0.0188	0.0229	0.0192	0.0213	0.0192	0.0175	0.0239	0.0156	0.0171	0.0191	0.0105	0.0155
0.0122	0.0121	0.0202	0.0195	0.0169	0.0162	0.0775	0.0112	0.0141	0.0308	0.0154	0.0166	0.0189	0.0189	0.0118	0.0204	0.0168	0.0194	0.0415	0.0133	0.0116	0.0128	0.0174	0.0220	0.1076	0.0101	0.0112	0.0199	0.0262	0.0211	0.0060	0.0203	0.0194	0.0218	0.0192	0.0080	0.0190	0.0224	0.0194	0.0211	0.0194	0.0134	0.0227	0.0147	0.0169	0.0195	0.0117	0.0160
0.0173	0.0172	0.0148	0.0161	0.0218	0.0108	0.0746	0.0163	0.0095	0.0277	0.0204	0.0217	0.0156	0.0157	0.0168	0.0159	0.0210	0.0160	0.0411	0.0073	0.0164	0.0179	0.0186	0.0274	0.1135	0.0151	0.0161	0.0137	0.0205	0.0264	0.0121	0.0141	0.0138	0.0270	0.0134	0.0133	0.0133	0.0176	0.0137	0.0262	0.0136	0.0074	0.0199	0.0127	0.0132	0.0135	0.0165	0.0102

0.0725	0.0726	0.0733	0.0730	0.0761	0.0706	0.0055	0.0764	0.0716	0.0623	0.0733	0.0775	0.0728	0.0708	0.0727	0.0733	0.0790	0.0742	0.0713	0.0708	0.0731	0.0770	0.0761	0.0816	0.1469	0.0724	0.0743	0.0722	0.0783	0.0838	0.0752	0.0727	0.0712	0.0818	0.0734	0.0735	0.0711	0.0781	0.0712	0.0812	0.0737	0.0709	0.0781	0.0712	0.0725	0.0736	0.0731	0.0700
0.0607	0.0609	0.0535	0.0564	0.0610	0.0535	0.1163	0.0583	0.0538	0.0684	0.0634	0.0628	0.0592	0.0583	0.0595	0.0572	0.0680	0.0551	0.0595	0.0522	0.0591	0.0611	0.0589	0.0670	0.1463	0.0582	0.0596	0.0533	0.0619	0.0651	0.0575	0.0540	0.0567	0.0668	0.0526	0.0576	0.0565	0.0600	0.0566	0.0675	0.0527	0.0527	0.0627	0.0560	0.0548	0.0527	0.0590	0.0535
0.1572	0.1578	0.1561	0.1566	0.1565	0.1558	0.1394	0.1556	0.1514	0.1675	0.1607	0.1595	0.1594	0.1565	0.1558	0.1490	0.1627	0.1549	0.1298	0.1522	0.1561	0.1570	0.1556	0.1632	0.1672	0.1556	0.1562	0.1551	0.1647	0.1624	0.1533	0.1561	0.1577	0.1636	0.1546	0.1512	0.1581	0.1629	0.1582	0.1650	0.1549	0.1521	0.1624	0.1559	0.1555	0.1548	0.1562	0.1557
0.0197	0.0196	0.0167	0.0193	0.0242	0.0131	0.0745	0.0192	0.0038	0.0274	0.0211	0.0223	0.0184	0.0163	0.0184	0.0155	0.0215	0.0188	0.0409	0.0101	0.0180	0.0190	0.0201	0.0274	0.1142	0.0181	0.0188	0.0159	0.0229	0.0277	0.0152	0.0163	0.0148	0.0271	0.0157	0.0141	0.0144	0.0189	0.0149	0.0282	0.0160	0.0103	0.0214	0.0150	0.0156	0.0160	0.0181	0.0130
0.0138	0.0138	0.0214	0.0214	0.0187	0.0174	0.0767	0.0130	0.0132	0.0295	0.0156	0.0174	0.0202	0.0184	0.0126	0.0204	0.0167	0.0198	0.0412	0.0129	0.0122	0.0137	0.0184	0.0219	0.1088	0.0120	0.0125	0.0205	0.0269	0.0223	0.0106	0.0208	0.0194	0.0215	0.0193	0.0088	0.0191	0.0236	0.0195	0.0223	0.0196	0.0132	0.0233	0.0172	0.0186	0.0198	0.0126	0.0169
0.0129	0.0130	0.0210	0.0210	0.0189	0.0165	0.0781	0.0132	0.0127	0.0297	0.0151	0.0172	0.0196	0.0180	0.0122	0.0198	0.0170	0.0196	0.0425	0.0118	0.0117	0.0132	0.0179	0.0219	0.1106	0.0115	0.0119	0.0203	0.0265	0.0223	0.0098	0.0207	0.0190	0.0215	0.0191	0.0080	0.0185	0.0231	0.0189	0.0219	0.0193	0.0121	0.0231	0.0166	0.0180	0.0190	0.0119	0.0163
0.0114	0.0113	0.0186	0.0169	0.0163	0.0147	0.0757	0.0110	0.0157	0.0281	0.0136	0.0151	0.0173	0.0162	0.0105	0.0174	0.0151	0.0179	0.0384	0.0158	0.0099	0.0149	0.0179	0.0206	0.1100	0.0101	0.0104	0.0186	0.0256	0.0202	0.0119	0.0189	0.0178	0.0204	0.0180	0.0122	0.0174	0.0224	0.0177	0.0212	0.0185	0.0161	0.0222	0.0150	0.0165	0.0185	0.0104	0.0150
0.0134	0.0133	0.0213	0.0203	0.0178	0.0170	0.0770	0.0124	0.0129	0.0298	0.0164	0.0171	0.0202	0.0194	0.0131	0.0194	0.0170	0.0205	0.0424	0.0137	0.0128	0.0124	0.0182	0.0226	0.1083	0.0115	0.0126	0.0209	0.0263	0.0214	0.0073	0.0211	0.0203	0.0223	0.0196	0.0075	0.0200	0.0226	0.0204	0.0217	0.0199	0.0141	0.0237	0.0152	0.0178	0.0198	0.0126	0.0169
0.0173	0.0172	0.0144	0.0170	0.0215	0.0115	0.0713	0.0176	0.0082	0.0259	0.0184	0.0200	0.0157	0.0139	0.0158	0.0149	0.0175	0.0161	0.0391	0.0110	0.0161	0.0194	0.0199	0.0244	0.1167	0.0150	0.0164	0.0125	0.0209	0.0255	0.0167	0.0129	0.0119	0.0243	0.0134	0.0165	0.0120	0.0178	0.0124	0.0260	0.0137	0.0112	0.0201	0.0140	0.0141	0.0136	0.0156	0.0105
0.0825	0.0823	0.0771	0.0799	0.0799	0.0799	0.0994	0.0840	0.0823	0.0725	0.0820	0.0768	0.0796	0.0794	0.0782	0.0859	0.0792	0.0792	0.0845	0.0823	0.0789	0.0801	0.0829	0.0755	0.1601	0.0789	0.0810	0.0783	0.0811	0.0753	0.0828	0.0789	0.0796	0.0755	0.0797	0.0824	0.0798	0.0781	0.0797	0.0815	0.0796	0.0824	0.0776	0.0808	0.0799	0.0798	0.0791	0.0802
0.0355	0.0356	0.0339	0.0293	0.0382	0.0305	0.0932	0.0369	0.0310	0.0434	0.0369	0.0380	0.0309	0.0296	0.0342	0.0376	0.0408	0.0300	0.0593	0.0292	0.0337	0.0367	0.0338	0.0432	0.1342	0.0328	0.0344	0.0341	0.0411	0.0424	0.0325	0.0344	0.0342	0.0430	0.0337	0.0322	0.0341	0.0377	0.0344	0.0444	0.0338	0.0298	0.0347	0.0283	0.0306	0.0338	0.0330	0.0305
0.0183	0.0182	0.0151	0.0176	0.0229	0.0112	0.0742	0.0176	0.0082	0.0270	0.0208	0.0225	0.0169	0.0160	0.0176	0.0153	0.0219	0.0172	0.0419	0.0058	0.0174	0.0178	0.0193	0.0280	0.1150	0.0167	0.0173	0.0145	0.0213	0.0271	0.0133	0.0150	0.0148	0.0278	0.0142	0.0123	0.0145	0.0178	0.0147	0.0271	0.0145	0.0059	0.0214	0.0132	0.0138	0.0145	0.0175	0.0113
0.0127	0.0127	0.0190	0.0182	0.0156	0.0149	0.0759	0.0124	0.0143	0.0278	0.0143	0.0151	0.0185	0.0177	0.0113	0.0177	0.0154	0.0181	0.0401	0.0130	0.0112	0.0129	0.0160	0.0198	0.1078	0.0100	0.0116	0.0195	0.0256	0.0193	0.0099	0.0197	0.0195	0.0195	0.0183	0.0079	0.0192	0.0221	0.0196	0.0209	0.0186	0.0132	0.0222	0.0152	0.0167	0.0186	0.0105	0.0157
0.0167	0.0166	0.0174	0.0189	0.0248	0.0132	0.0707	0.0199	0.0108	0.0288	0.0201	0.0241	0.0193	0.0187	0.0203	0.0175	0.0233	0.0192	0.0440	0.0100	0.0197	0.0199	0.0213	0.0295	0.1141	0.0179	0.0195	0.0172	0.0188	0.0284	0.0170	0.0175	0.0164	0.0292	0.0159	0.0147	0.0160	0.0192	0.0165	0.0249	0.0163	0.0101	0.0226	0.0159	0.0157	0.0161	0.0194	0.0126
0.0172	0.0170	0.0125	0.0153	0.0202	0.0099	0.0748	0.0173	0.0106	0.0263	0.0197	0.0204	0.0166	0.0147	0.0161	0.0155	0.0210	0.0160	0.0404	0.0089	0.0157	0.0191	0.0170	0.0265	0.1144	0.0155	0.0164	0.0139	0.0210	0.0241	0.0153	0.0142	0.0128	0.0262	0.0133	0.0152	0.0126	0.0181	0.0129	0.0266	0.0136	0.0090	0.0202	0.0133	0.0123	0.0135	0.0163	0.0106
0.0525	0.0528	0.0450	0.0480	0.0531	0.0448	0.1047	0.0525	0.0455	0.0633	0.0551	0.0550	0.0510	0.0498	0.0510	0.0506	0.0586	0.0476	0.0537	0.0427	0.0509	0.0534	0.0499	0.0595	0.1547	0.0499	0.0515	0.0453	0.0550	0.0572	0.0484	0.0459	0.0481	0.0592	0.0446	0.0488	0.0480	0.0527	0.0483	0.0610	0.0447	0.0428	0.0552	0.0488	0.0469	0.0446	0.0512	0.0446
0.0117	0.0117	0.0194	0.0180	0.0156	0.0161	0.0753	0.0132	0.0173	0.0304	0.0144	0.0158	0.0201	0.0197	0.0130	0.0201	0.0175	0.0200	0.0413	0.0152	0.0128	0.0152	0.0167	0.0227	0.1044	0.0106	0.0123	0.0209	0.0257	0.0200	0.0098	0.0212	0.0195	0.0223	0.0202	0.0112	0.0191	0.0238	0.0195	0.0206	0.0206	0.0156	0.0238	0.0166	0.0174	0.0205	0.0118	0.0164
0.0161	0.0160	0.0153	0.0163	0.0226	0.0126	0.0716	0.0197	0.0134	0.0290	0.0200	0.0234	0.0193	0.0184	0.0196	0.0176	0.0245	0.0193	0.0433	0.0115	0.0189	0.0218	0.0198	0.0302	0.1136	0.0180	0.0190	0.0166	0.0200	0.0268	0.0163	0.0170	0.0146	0.0300	0.0158	0.0178	0.0142	0.0208	0.0147	0.0258	0.0161	0.0115	0.0230	0.0167	0.0157	0.0160	0.0192	0.0122
0.3873	0.3877	0.3866	0.3867	0.3873	0.3877	0.3284	0.4009	0.3872	0.3897	0.3875	0.3877	0.3877	0.3881	0.3875	0.4052	0.4124	0.3876	0.3615	0.3870	0.3874	0.3874	0.3856	0.3873	0.4458	0.3880	0.3880	0.3865	0.3884	0.3880	0.3864	0.3871	0.3868	0.3875	0.3864	0.3869	0.3865	0.3883	0.3871	0.3876	0.3865	0.3874	0.3877	0.3875	0.3875	0.3866	0.3869	0.3874
0.0162	0.0160	0.0160	0.0192	0.0127	0.0192	0.0766	0.0157	0.0213	0.0307	0.0184	0.0158	0.0230	0.0215	0.0137	0.0216	0.0181	0.0164	0.0420	0.0207	0.0143	0.0183	0.0174	0.0205	0.1157	0.0129	0.0153	0.0161	0.0292	0.0167	0.0172	0.0162	0.0222	0.0202	0.0159	0.0154	0.0222	0.0263	0.0226	0.0238	0.0162	0.0209	0.0269	0.0194	0.0176	0.0161	0.0145	0.0191
0.0466	0.0466	0.0472	0.0458	0.0500	0.0449	0.1097	0.0485	0.0445	0.0632	0.0497	0.0501	0.0478	0.0471	0.0459	0.0528	0.0523	0.0477	0.0741	0.0425	0.0458	0.0475	0.0478	0.0567	0.1522	0.0437	0.0453	0.0485	0.0550	0.0541	0.0435	0.0489	0.0458	0.0567	0.0476	0.0431	0.0454	0.0520	0.0457	0.0559	0.0477	0.0425	0.0522	0.0448	0.0452	0.0477	0.0455	0.0435
0.0585	0.0586	0.0560	0.0521	0.0608	0.0534	0.1141	0.0584	0.0534	0.0631	0.0609	0.0559	0.0511	0.0530	0.0571	0.0573	0.0538	0.0523	0.0643	0.0511	0.0571	0.0538	0.0554	0.0562	0.1589	0.0556	0.0573	0.0561	0.0543	0.0553	0.0551	0.0567	0.0574	0.0560	0.0556	0.0543	0.0569	0.0508	0.0574	0.0572	0.0559	0.0512	0.0484	0.0517	0.0531	0.0559	0.0567	0.0536
0.0115	0.0115	0.0184	0.0188	0.0157	0.0145	0.0773	0.0110	0.0153	0.0299	0.0146	0.0158	0.0185	0.0178	0.0102	0.0193	0.0164	0.0187	0.0408	0.0130	0.0100	0.0130	0.0165	0.0212	0.1073	0.0096	0.0103	0.0187	0.0255	0.0204	0.0081	0.0190	0.0184	0.0206	0.0182	0.0091	0.0181	0.0225	0.0184	0.0207	0.0185	0.0134	0.0228	0.0151	0.0167	0.0184	0.0109	0.0155
0.0901	0.0901	0.0833	0.0817	0.0858	0.0844	0.0627	0.0924	0.0880	0.0715	0.0892	0.0899	0.0838	0.0813	0.0867	0.0886	0.0979	0.0813	0.0564	0.0868	0.0873	0.0937	0.0883	0.0939	0.1603	0.0865	0.0893	0.0832	0.0964	0.0947	0.0894	0.0837	0.0864	0.0941	0.0852	0.0906	0.0863	0.0928	0.0864	0.0997	0.0851	0.0867	0.0899	0.0821	0.0831	0.0850	0.0865	0.0852
0.0440	0.0439	0.0369	0.0353	0.0416	0.0378	0.1009	0.0441	0.0438	0.0502	0.0457	0.0447	0.0394	0.0380	0.0428	0.0442	0.0502	0.0346	0.0445	0.0418	0.0423	0.0474	0.0413	0.0494	0.1467	0.0414	0.0427	0.0392	0.0494	0.0466	0.0442	0.0397	0.0407	0.0495	0.0385	0.0452	0.0404	0.0457	0.0406	0.0527	0.0385	0.0420	0.0441	0.0360	0.0366	0.0385	0.0417	0.0388
0.1231	0.1229	0.1215	0.1214	0.1237	0.1223	0.1846	0.1277	0.1224	0.1390	0.1245	0.1258	0.1259	0.1245	0.1221	0.1193	0.1330	0.1222	0.1270	0.1246	0.1224	0.1273	0.1264	0.1307	0.1177	0.1220	0.1242	0.1213	0.1302	0.1294	0.1245	0.1223	0.1263	0.1313	0.1224	0.1240	0.1260	0.1300	0.1265	0.1303	0.1224	0.1243	0.1300	0.1222	0.1222	0.1226	0.1230	0.1239
0.0134	0.0134	0.0195	0.0194	0.0160	0.0156	0.0759	0.0124	0.0138	0.0289	0.0159	0.0163	0.0202	0.0187	0.0117	0.0181	0.0167	0.0196	0.0414	0.0141	0.0115	0.0129	0.0168	0.0218	0.1084	0.0106	0.0118	0.0198	0.0260	0.0204	0.0092	0.0202	0.0200	0.0214	0.0189	0.0080	0.0196	0.0233	0.0199	0.0218	0.0191	0.0142	0.0229	0.0162	0.0178	0.0190	0.0119	0.0163
0.0419	0.0419	0.0442	0.0443	0.0431	0.0416	0.0954	0.0443	0.0410	0.0563	0.0397	0.0431	0.0413	0.0401	0.0418	0.0483	0.0446	0.0437	0.0643	0.0412	0.0413	0.0432	0.0454	0.0451	0.1388	0.0416	0.0420	0.0433	0.0462	0.0472	0.0414	0.0436	0.0406	0.0447	0.0436	0.0414	0.0402	0.0456	0.0408	0.0460	0.0441	0.0416	0.0451	0.0416	0.0426	0.0440	0.0402	0.0417
0.0130	0.0131	0.0205	0.0195	0.0169	0.0160	0.0768	0.0130	0.0141	0.0298	0.0156	0.0164	0.0200	0.0189	0.0121	0.0187	0.0167	0.0198	0.0418	0.0140	0.0121	0.0132	0.0177	0.0213	0.1090	0.0108	0.0122	0.0204	0.0259	0.0199	0.0090	0.0205	0.0197	0.0211	0.0190	0.0088	0.0195	0.0235	0.0200	0.0212	0.0192	0.0142	0.0231	0.0165	0.0181	0.0193	0.0122	0.0163
0.0106	0.0105	0.0217	0.0209	0.0186	0.0180	0.0759	0.0139	0.0168	0.0332	0.0142	0.0187	0.0217	0.0207	0.0141	0.0219	0.0192	0.0213	0.0435	0.0167	0.0137	0.0152	0.0189	0.0247	0.1073	0.0125	0.0137	0.0221	0.0250	0.0232	0.0100	0.0223	0.0216	0.0244	0.0212	0.0107	0.0212	0.0253	0.0219	0.0203	0.0215	0.0169	0.0254	0.0173	0.0195	0.0214	0.0138	0.0182
0.0170	0.0170	0.0141	0.0118	0.0188	0.0113	0.0710	0.0168	0.0145	0.0219	0.0189	0.0191	0.0128	0.0117	0.0148	0.0154	0.0182	0.0119	0.0405	0.0140	0.0152	0.0193	0.0165	0.0246	0.1162	0.0136	0.0160	0.0150	0.0222	0.0239	0.0182	0.0154	0.0162	0.0242	0.0149	0.0165	0.0159	0.0187	0.0161	0.0253	0.0152	0.0142	0.0166	0.0098	0.0123	0.0151	0.0149	0.0126
0.0375	0.0376	0.0387	0.0405	0.0356	0.0397	0.0977	0.0376	0.0425	0.0507	0.0387	0.0378	0.0429	0.0421	0.0345	0.0438	0.0424	0.0387	0.0444	0.0413	0.0352	0.0392	0.0405	0.0420	0.1377	0.0333	0.0359	0.0388	0.0502	0.0409	0.0381	0.0391	0.0438	0.0419	0.0388	0.0363	0.0437	0.0463	0.0439	0.0452	0.0390	0.0415	0.0468	0.0395	0.0398	0.0391	0.0348	0.0404
0.0483	0.0485	0.0503	0.0524	0.0477	0.0518	0.1067	0.0473	0.0560	0.0665	0.0511	0.0504	0.0555	0.0538	0.0453	0.0566	0.0541	0.0512	0.0545	0.0541	0.0462	0.0513	0.0511	0.0550	0.1519	0.0447	0.0468	0.0509	0.0626	0.0535	0.0489	0.0512	0.0554	0.0550	0.0513	0.0492	0.0552	0.0597	0.0556	0.0575	0.0514	0.0543	0.0597	0.0517	0.0518	0.0514	0.0467	0.0520
0.0146	0.0144	0.0154	0.0173	0.0235	0.0116	0.0725	0.0179	0.0118	0.0293	0.0186	0.0228	0.0180	0.0169	0.0177	0.0173	0.0229	0.0177	0.0424	0.0097	0.0174	0.0200	0.0194	0.0287	0.1131	0.0161	0.0173	0.0149	0.0190	0.0274	0.0153	0.0154	0.0148	0.0285	0.0149	0.0160	0.0144	0.0192	0.0148	0.0244	0.0152	0.0100	0.0213	0.0147	0.0149	0.0151	0.0177	0.0114
0.0251	0.0252	0.0203	0.0209	0.0249	0.0236	0.0804	0.0289	0.0269	0.0349	0.0276	0.0238	0.0216	0.0238	0.0266	0.0290	0.0236	0.0183	0.0522	0.0256	0.0270	0.0264	0.0266	0.0239	0.1289	0.0256	0.0274	0.0212	0.0217	0.0202	0.0292	0.0216	0.0279	0.0237	0.0213	0.0284	0.0277	0.0209	0.0282	0.0245	0.0215	0.0259	0.0196	0.0222	0.0217	0.0214	0.0270	0.0244
0.0739	0.0737	0.0751	0.0751	0.0781	0.0725	0.0040	0.0774	0.0734	0.0637	0.0744	0.0792	0.0746	0.0727	0.0747	0.0754	0.0817	0.0765	0.0728	0.0727	0.0750	0.0790	0.0780	0.0837	0.1486	0.0743	0.0763	0.0740	0.0795	0.0857	0.0773	0.0746	0.0728	0.0836	0.0755	0.0755	0.0728	0.0800	0.0731	0.0824	0.0757	0.0729	0.0800	0.0732	0.0745	0.0754	0.0751	0.0718
0.0612	0.0610	0.0559	0.0529	0.0607	0.0551	0.0887	0.0617	0.0589	0.0398	0.0598	0.0611	0.0550	0.0517	0.0586	0.0594	0.0663	0.0516	0.0577	0.0585	0.0582	0.0642	0.0597	0.0637	0.1239	0.0584	0.0598	0.0556	0.0663	0.0654	0.0613	0.0563	0.0560	0.0638	0.0557	0.0621	0.0559	0.0624	0.0562	0.0699	0.0556	0.0586	0.0602	0.0530	0.0544	0.0554	0.0572	0.0554
0.0150	0.0147	0.0114	0.0137	0.0179	0.0071	0.0721	0.0146	0.0121	0.0243	0.0166	0.0187	0.0141	0.0114	0.0132	0.0128	0.0188	0.0130	0.0376	0.0099	0.0126	0.0185	0.0170	0.0233	0.1161	0.0131	0.0140	0.0110	0.0176	0.0226	0.0153	0.0114	0.0110	0.0231	0.0100	0.0163	0.0108	0.0151	0.0112	0.0234	0.0106	0.0101	0.0185	0.0098	0.0102	0.0103	0.0132	0.0072
0.0151	0.0151	0.0152	0.0175	0.0225	0.0113	0.0713	0.0188	0.0157	0.0292	0.0179	0.0224	0.0178	0.0170	0.0183	0.0174	0.0226	0.0175	0.0426	0.0140	0.0178	0.0223	0.0209	0.0282	0.1173	0.0169	0.0173	0.0160	0.0196	0.0269	0.0191	0.0163	0.0152	0.0276	0.0150	0.0203	0.0149	0.0194	0.0153	0.0246	0.0153	0.0142	0.0217	0.0143	0.0144	0.0153	0.0178	0.0123
0.0145	0.0143	0.0107	0.0138	0.0185	0.0066	0.0729	0.0149	0.0124	0.0249	0.0172	0.0181	0.0139	0.0125	0.0145	0.0134	0.0182	0.0127	0.0379	0.0100	0.0138	0.0190	0.0168	0.0229	0.1154	0.0133	0.0138	0.0116	0.0182	0.0235	0.0165	0.0120	0.0112	0.0228	0.0103	0.0170	0.0111	0.0147	0.0114	0.0243	0.0107	0.0101	0.0182	0.0100	0.0101	0.0104	0.0134	0.0079
0.0151	0.0149	0.0108	0.0138	0.0187	0.0065	0.0727	0.0151	0.0125	0.0252	0.0172	0.0184	0.0139	0.0124	0.0142	0.0136	0.0185	0.0125	0.0378	0.0102	0.0139	0.0190	0.0171	0.0229	0.1161	0.0134	0.0139	0.0114	0.0183	0.0235	0.0168	0.0118	0.0113	0.0224	0.0102	0.0170	0.0111	0.0147	0.0114	0.0243	0.0106	0.0103	0.0183	0.0102	0.0101	0.0105	0.0137	0.0079
0.0792	0.0789	0.0846	0.0845	0.0813	0.0841	0.1185	0.0863	0.0858	0.0722	0.0774	0.0810	0.0869	0.0842	0.0799	0.0858	0.0879	0.0839	0.0855	0.0863	0.0791	0.0842	0.0860	0.0845	0.0957	0.0784	0.0809	0.0850	0.0936	0.0858	0.0817	0.0857	0.0859	0.0851	0.0842	0.0807	0.0858	0.0925	0.0860	0.0885	0.0843	0.0866	0.0928	0.0838	0.0843	0.0842	0.0779	0.0852
0.0122	0.0121	0.0181	0.0181	0.0148	0.0141	0.0764	0.0115	0.0182	0.0290	0.0133	0.0145	0.0183	0.0166	0.0100	0.0186	0.0150	0.0172	0.0389	0.0180	0.0095	0.0156	0.0173	0.0192	0.1095	0.0092	0.0110	0.0188	0.0260	0.0194	0.0123	0.0189	0.0179	0.0190	0.0179	0.0131	0.0176	0.0220	0.0181	0.0208	0.0183	0.0181	0.0223	0.0143	0.0160	0.0179	0.0096	0.0149
0.0123	0.0121	0.0186	0.0173	0.0143	0.0140	0.0766	0.0115	0.0189	0.0282	0.0141	0.0150	0.0186	0.0173	0.0110	0.0181	0.0156	0.0176	0.0394	0.0179	0.0107	0.0153	0.0172	0.0200	0.1090	0.0093	0.0112	0.0189	0.0255	0.0191	0.0121	0.0191	0.0188	0.0197	0.0180	0.0132	0.0184	0.0219	0.0189	0.0206	0.0182	0.0182	0.0230	0.0140	0.0161	0.0182	0.0101	0.0148
0.0127	0.0126	0.0138	0.0162	0.0113	0.0158	0.0782	0.0126	0.0204	0.0306	0.0155	0.0134	0.0191	0.0184	0.0123	0.0220	0.0167	0.0136	0.0402	0.0172	0.0120	0.0167	0.0167	0.0185	0.1126	0.0108	0.0113	0.0142	0.0266	0.0151	0.0141	0.0143	0.0197	0.0184	0.0132	0.0146	0.0195	0.0238	0.0198	0.0224	0.0133	0.0177	0.0240	0.0161	0.0144	0.0134	0.0118	0.0166
0.0400	0.0399	0.0445	0.0434	0.0447	0.0410	0.0365	0.0411	0.0398	0.0416	0.0412	0.0433	0.0428	0.0418	0.0397	0.0426	0.0453	0.0439	0.0430	0.0412	0.0394	0.0425	0.0429	0.0490	0.1283	0.0393	0.0402	0.0434	0.0491	0.0492	0.0410	0.0437	0.0424	0.0488	0.0434	0.0398	0.0420	0.0476	0.0424	0.0483	0.0437	0.0412	0.0480	0.0411	0.0416	0.0436	0.0392	0.0403
0.0158	0.0156	0.0160	0.0181	0.0235	0.0121	0.0718	0.0188	0.0129	0.0298	0.0196	0.0236	0.0189	0.0181	0.0189	0.0170	0.0237	0.0186	0.0429	0.0108	0.0184	0.0211	0.0198	0.0295	0.1127	0.0172	0.0184	0.0160	0.0194	0.0277	0.0157	0.0163	0.0152	0.0293	0.0153	0.0171	0.0151	0.0203	0.0154	0.0251	0.0157	0.0109	0.0220	0.0159	0.0159	0.0154	0.0188	0.0120
0.0129	0.0127	0.0203	0.0190	0.0177	0.0164	0.0753	0.0117	0.0134	0.0281	0.0146	0.0158	0.0185	0.0174	0.0113	0.0182	0.0153	0.0192	0.0396	0.0167	0.0108	0.0138	0.0192	0.0210	0.1118	0.0112	0.0113	0.0196	0.0258	0.0200	0.0119	0.0198	0.0183	0.0206	0.0185	0.0108	0.0180	0.0221	0.0184	0.0211	0.0189	0.0168	0.0225	0.0154	0.0171	0.0187	0.0116	0.0156
0.0170	0.0169	0.0148	0.0160	0.0224	0.0106	0.0717	0.0171	0.0072	0.0252	0.0179	0.0197	0.0149	0.0129	0.0162	0.0139	0.0182	0.0160	0.0387	0.0107	0.0155	0.0193	0.0201	0.0247	0.1176	0.0158	0.0158	0.0126	0.0202	0.0251	0.0171	0.0128	0.0111	0.0245	0.0125	0.0163	0.0108	0.0173	0.0113	0.0258	0.0129	0.0107	0.0195	0.0133	0.0131	0.0128	0.0155	0.0096
0.0127	0.0126	0.0208	0.0194	0.0176	0.0170	0.0758	0.0123	0.0142	0.0297	0.0160	0.0167	0.0193	0.0192	0.0125	0.0197	0.0160	0.0197	0.0420	0.0125	0.0123	0.0122	0.0177	0.0222	0.1088	0.0110	0.0114	0.0201	0.0260	0.0209	0.0085	0.0203	0.0194	0.0218	0.0189	0.0072	0.0192	0.0231	0.0196	0.0215	0.0193	0.0126	0.0232	0.0163	0.0179	0.0193	0.0122	0.0160
0.0126	0.0126	0.0200	0.0191	0.0164	0.0159	0.0775	0.0111	0.0147	0.0312	0.0151	0.0164	0.0190	0.0186	0.0117	0.0201	0.0172	0.0192	0.0413	0.0137	0.0115	0.0127	0.0172	0.0221	0.1078	0.0103	0.0111	0.0193	0.0259	0.0208	0.0062	0.0195	0.0192	0.0217	0.0191	0.0083	0.0189	0.0224	0.0192	0.0208	0.0193	0.0139	0.0228	0.0145	0.0167	0.0194	0.0117	0.0159
0.0167	0.0166	0.0174	0.0191	0.0248	0.0134	0.0706	0.0200	0.0111	0.0292	0.0202	0.0242	0.0196	0.0188	0.0203	0.0178	0.0234	0.0194	0.0441	0.0102	0.0199	0.0199	0.0213	0.0295	0.1139	0.0180	0.0197	0.0170	0.0187	0.0283	0.0170	0.0173	0.0164	0.0292	0.0159	0.0147	0.0161	0.0192	0.0166	0.0249	0.0162	0.0102	0.0229	0.0161	0.0158	0.0161	0.0196	0.0126
0.0137	0.0137	0.0191	0.0185	0.0156	0.0165	0.0781	0.0138	0.0212	0.0288	0.0155	0.0165	0.0211	0.0185	0.0129	0.0200	0.0174	0.0198	0.0404	0.0195	0.0125	0.0175	0.0180	0.0220	0.1125	0.0119	0.0132	0.0203	0.0277	0.0203	0.0151	0.0205	0.0190	0.0218	0.0195	0.0155	0.0188	0.0244	0.0192	0.0227	0.0199	0.0197	0.0259	0.0165	0.0173	0.0198	0.0122	0.0171
0.0262	0.0260	0.0234	0.0233	0.0263	0.0235	0.0834	0.0269	0.0267	0.0353	0.0265	0.0258	0.0260	0.0249	0.0260	0.0284	0.0307	0.0228	0.0322	0.0263	0.0254	0.0292	0.0252	0.0322	0.1263	0.0243	0.0261	0.0243	0.0330	0.0307	0.0276	0.0246	0.0262	0.0319	0.0237	0.0273	0.0259	0.0308	0.0263	0.0344	0.0241	0.0264	0.0310	0.0235	0.0215	0.0237	0.0235	0.0244
0.0518	0.0521	0.0575	0.0563	0.0532	0.0539	0.0956	0.0505	0.0582	0.0661	0.0539	0.0524	0.0572	0.0572	0.0506	0.0499	0.0537	0.0578	0.0581	0.0566	0.0506	0.0527	0.0562	0.0577	0.0917	0.0482	0.0506	0.0579	0.0627	0.0557	0.0521	0.0585	0.0577	0.0577	0.0578	0.0517	0.0578	0.0596	0.0580	0.0578	0.0582	0.0563	0.0610	0.0544	0.0547	0.0581	0.0494	0.0548
0.0612	0.0611	0.0632	0.0628	0.0598	0.0632	0.0960	0.0608	0.0665	0.0503	0.0597	0.0604	0.0655	0.0622	0.0585	0.0654	0.0647	0.0622	0.0627	0.0661	0.0583	0.0642	0.0642	0.0635	0.1237	0.0578	0.0599	0.0633	0.0744	0.0648	0.0610	0.0639	0.0645	0.0634	0.0637	0.0620	0.0646	0.0709	0.0647	0.0699	0.0637	0.0664	0.0709	0.0629	0.0631	0.0635	0.0568	0.0636
0.0357	0.0353	0.0314	0.0318	0.0391	0.0273	0.0635	0.0362	0.0314	0.0147	0.0345	0.0374	0.0328	0.0299	0.0338	0.0309	0.0382	0.0326	0.0562	0.0305	0.0333	0.0395	0.0375	0.0406	0.0938	0.0331	0.0349	0.0308	0.0384	0.0439	0.0364	0.0312	0.0281	0.0404	0.0307	0.0371	0.0279	0.0350	0.0282	0.0449	0.0308	0.0306	0.0385	0.0303	0.0304	0.0307	0.0322	0.0272
0.0515	0.0516	0.0462	0.0476	0.0485	0.0479	0.1095	0.0536	0.0515	0.0634	0.0537	0.0509	0.0524	0.0516	0.0500	0.0547	0.0575	0.0477	0.0701	0.0503	0.0496	0.0535	0.0511	0.0559	0.1578	0.0488	0.0497	0.0483	0.0591	0.0524	0.0515	0.0485	0.0509	0.0555	0.0473	0.0504	0.0507	0.0556	0.0510	0.0596	0.0474	0.0505	0.0564	0.0490	0.0474	0.0474	0.0492	0.0491
0.0356	0.0353	0.0335	0.0341	0.0400	0.0314	0.0939	0.0365	0.0315	0.0474	0.0387	0.0401	0.0368	0.0358	0.0365	0.0354	0.0404	0.0363	0.0610	0.0292	0.0362	0.0383	0.0367	0.0469	0.1353	0.0345	0.0359	0.0348	0.0401	0.0439	0.0336	0.0351	0.0326	0.0466	0.0341	0.0346	0.0323	0.0388	0.0327	0.0449	0.0343	0.0293	0.0406	0.0339	0.0330	0.0343	0.0359	0.0303
0.0367	0.0364	0.0357	0.0305	0.0404	0.0313	0.0620	0.0373	0.0330	0.0119	0.0355	0.0380	0.0310	0.0272	0.0343	0.0328	0.0390	0.0309	0.0582	0.0330	0.0339	0.0387	0.0371	0.0409	0.0961	0.0347	0.0359	0.0340	0.0409	0.0441	0.0373	0.0346	0.0319	0.0407	0.0334	0.0358	0.0317	0.0385	0.0320	0.0446	0.0335	0.0332	0.0362	0.0292	0.0319	0.0335	0.0334	0.0310
0.0134	0.0132	0.0191	0.0184	0.0156	0.0153	0.0779	0.0130	0.0162	0.0300	0.0149	0.0159	0.0192	0.0186	0.0120	0.0191	0.0167	0.0187	0.0406	0.0144	0.0117	0.0143	0.0163	0.0205	0.1067	0.0102	0.0122	0.0197	0.0264	0.0202	0.0096	0.0199	0.0195	0.0203	0.0193	0.0097	0.0193	0.0228	0.0196	0.0217	0.0195	0.0147	0.0233	0.0153	0.0170	0.0194	0.0109	0.0162
0.0432	0.0432	0.0366	0.0346	0.0408	0.0375	0.0997	0.0433	0.0431	0.0496	0.0448	0.0438	0.0386	0.0372	0.0422	0.0437	0.0494	0.0339	0.0440	0.0411	0.0419	0.0465	0.0405	0.0488	0.1462	0.0408	0.0420	0.0386	0.0489	0.0464	0.0436	0.0392	0.0399	0.0487	0.0377	0.0443	0.0398	0.0451	0.0401	0.0523	0.0379	0.0412	0.0431	0.0355	0.0358	0.0377	0.0409	0.0380
0.1084	0.1084	0.1097	0.1084	0.1118	0.1066	0.1668	0.1146	0.1054	0.1236	0.1065	0.1051	0.1055	0.1046	0.1070	0.1167	0.1085	0.1104	0.1334	0.1065	0.1069	0.1056	0.1108	0.1053	0.2097	0.1064	0.1076	0.1090	0.1088	0.1085	0.1069	0.1093	0.1044	0.1055	0.1101	0.1068	0.1044	0.1061	0.1045	0.1090	0.1104	0.1068	0.1056	0.1075	0.1083	0.1104	0.1052	0.1064
0.0450	0.0451	0.0349	0.0395	0.0424	0.0363	0.0980	0.0443	0.0398	0.0491	0.0468	0.0456	0.0429	0.0417	0.0415	0.0398	0.0482	0.0387	0.0431	0.0388	0.0424	0.0466	0.0441	0.0498	0.1461	0.0409	0.0436	0.0359	0.0474	0.0481	0.0457	0.0363	0.0407	0.0496	0.0362	0.0436	0.0406	0.0436	0.0407	0.0534	0.0363	0.0388	0.0469	0.0398	0.0383	0.0363	0.0423	0.0371
0.1393	0.1389	0.1427	0.1408	0.1439	0.1385	0.1986	0.1449	0.1382	0.1526	0.1367	0.1370	0.1374	0.1372	0.1393	0.1453	0.1335	0.1424	0.1668	0.1395	0.1395	0.1378	0.1433	0.1360	0.2166	0.1381	0.1403	0.1417	0.1375	0.1392	0.1398	0.1417	0.1372	0.1359	0.1423	0.1393	0.1364	0.1370	0.1369	0.1376	0.1428	0.1399	0.1364	0.1397	0.1410	0.1431	0.1376	0.1389
0.0746	0.0747	0.0833	0.0820	0.0776	0.0787	0.0452	0.0763	0.0835	0.0654	0.0739	0.0782	0.0816	0.0787	0.0749	0.0806	0.0806	0.0822	0.0749	0.0826	0.0747	0.0800	0.0815	0.0815	0.1422	0.0743	0.0759	0.0834	0.0885	0.0840	0.0780	0.0836	0.0806	0.0811	0.0833	0.0781	0.0806	0.0865	0.0805	0.0831	0.0834	0.0831	0.0870	0.0784	0.0806	0.0834	0.0736	0.0794

DS57	DS58	DS59	DS60	DS2	DS61	DS62	DS63	DS64	DS65	DS66	DS67	DS68	DS69	DS44	FC2	FC18	DS71	DS72	DS73	DS74	DS75	DS76	CL13	CL14	CL15	CL16	FC19	HL33	CL17	CL18	CL19	CL20	CL21	CL22	CL23	CL24	CL25	FC20	HL34	HL35	HL36	CL26	CL27	CL28	CL29	CL30	CL31	CL32

0.0127																																																
0.0161	0.0169																																															
0.0024	0.0129	0.0162																																														
0.0148	0.0123	0.0178	0.0144																																													
0.0337	0.0372	0.0267	0.0337	0.0367																																												
0.0133	0.0124	0.0163	0.0133	0.0141	0.0365																																											
0.0205	0.0199	0.0093	0.0205	0.0211	0.0300	0.0196																																										
0.0122	0.0152	0.0197	0.0121	0.0160	0.0340	0.0158	0.0229																																									
0.0138	0.0128	0.0168	0.0136	0.0135	0.0370	0.0027	0.0199	0.0150																																								
0.0144	0.0150	0.0220	0.0142	0.0176	0.0360	0.0143	0.0251	0.0088	0.0135																																							
0.0123	0.0154	0.0192	0.0121	0.0165	0.0333	0.0158	0.0226	0.0063	0.0150	0.0075																																						
0.0131	0.0169	0.0207	0.0128	0.0154	0.0346	0.0168	0.0241	0.0075	0.0164	0.0097	0.0057																																					
0.0107	0.0090	0.0149	0.0106	0.0108	0.0343	0.0102	0.0174	0.0124	0.0096	0.0134	0.0119	0.0135																																				
0.0140	0.0129	0.0169	0.0139	0.0136	0.0372	0.0029	0.0202	0.0152	0.0023	0.0135	0.0153	0.0164	0.0096																																			
0.0103	0.0110	0.0145	0.0101	0.0112	0.0337	0.0106	0.0177	0.0121	0.0099	0.0136	0.0111	0.0133	0.0071	0.0104																																		
0.0382	0.0413	0.0454	0.0379	0.0401	0.0595	0.0380	0.0486	0.0324	0.0374	0.0331	0.0322	0.0331	0.0368	0.0376	0.0373																																	
0.0150	0.0147	0.0172	0.0147	0.0136	0.0378	0.0144	0.0118	0.0164	0.0138	0.0185	0.0161	0.0180	0.0113	0.0139	0.0116	0.0421																																
0.0116	0.0105	0.0148	0.0114	0.0091	0.0332	0.0110	0.0176	0.0128	0.0102	0.0148	0.0130	0.0145	0.0082	0.0104	0.0078	0.0385	0.0109																															
0.0141	0.0138	0.0109	0.0140	0.0127	0.0304	0.0145	0.0145	0.0162	0.0138	0.0175	0.0159	0.0176	0.0112	0.0140	0.0111	0.0420	0.0145	0.0088																														
0.0183	0.0164	0.0076	0.0181	0.0170	0.0279	0.0159	0.0103	0.0189	0.0152	0.0205	0.0192	0.0206	0.0108	0.0155	0.0144	0.0444	0.0174	0.0144	0.0113																													
0.0120	0.0094	0.0160	0.0117	0.0119	0.0351	0.0116	0.0189	0.0130	0.0110	0.0147	0.0129	0.0141	0.0049	0.0111	0.0079	0.0378	0.0130	0.0092	0.0125	0.0124																												
0.0169	0.0177	0.0028	0.0167	0.0175	0.0270	0.0170	0.0098	0.0192	0.0164	0.0214	0.0188	0.0204	0.0146	0.0167	0.0144	0.0453	0.0168	0.0143	0.0104	0.0068	0.0158																											
0.0160	0.0151	0.0195	0.0157	0.0153	0.0383	0.0156	0.0197	0.0170	0.0152	0.0196	0.0168	0.0179	0.0121	0.0153	0.0108	0.0385	0.0133	0.0122	0.0160	0.0190	0.0126	0.0191																										
0.0321	0.0337	0.0389	0.0322	0.0331	0.0411	0.0319	0.0424	0.0310	0.0316	0.0317	0.0300	0.0307	0.0315	0.0319	0.0311	0.0378	0.0362	0.0326	0.0360	0.0381	0.0311	0.0389	0.0342																									
0.0375	0.0400	0.0345	0.0374	0.0384	0.0506	0.0355	0.0347	0.0390	0.0347	0.0392	0.0394	0.0396	0.0373	0.0351	0.0370	0.0613	0.0371	0.0375	0.0360	0.0337	0.0372	0.0340	0.0383	0.0540																								
0.0299	0.0291	0.0367	0.0299	0.0306	0.0508	0.0289	0.0319	0.0246	0.0281	0.0231	0.0247	0.0252	0.0299	0.0284	0.0301	0.0439	0.0254	0.0297	0.0326	0.0362	0.0307	0.0360	0.0292	0.0443	0.0404																							
0.0360	0.0356	0.0395	0.0358	0.0347	0.0595	0.0327	0.0428	0.0378	0.0322	0.0383	0.0374	0.0383	0.0319	0.0324	0.0323	0.0127	0.0367	0.0334	0.0367	0.0390	0.0330	0.0395	0.0325	0.0388	0.0588	0.0491																						
0.0517	0.0550	0.0578	0.0515	0.0513	0.0711	0.0542	0.0615	0.0455	0.0535	0.0479	0.0448	0.0448	0.0509	0.0538	0.0488	0.0714	0.0541	0.0510	0.0543	0.0576	0.0515	0.0578	0.0546	0.0686	0.0731	0.0628	0.0759																					
0.0323	0.0300	0.0262	0.0321	0.0284	0.0452	0.0272	0.0281	0.0340	0.0267	0.0325	0.0339	0.0342	0.0290	0.0269	0.0289	0.0534	0.0305	0.0281	0.0269	0.0252	0.0289	0.0256	0.0289	0.0494	0.0347	0.0363	0.0483	0.0706																				
0.0228	0.0227	0.0288	0.0226	0.0226	0.0450	0.0205	0.0306	0.0241	0.0199	0.0229	0.0241	0.0245	0.0218	0.0200	0.0217	0.0454	0.0241	0.0215	0.0250	0.0281	0.0221	0.0284	0.0243	0.0395	0.0384	0.0294	0.0430	0.0620	0.0188																			
0.1429	0.1442	0.1514	0.1424	0.1437	0.1675	0.1444	0.1532	0.1378	0.1435	0.1376	0.1379	0.1374	0.1431	0.1439	0.1441	0.1161	0.1463	0.1431	0.1467	0.1512	0.1439	0.1500	0.1465	0.1512	0.1613	0.1436	0.1221	0.1779	0.1544	0.1440																		
0.0347	0.0299	0.0381	0.0347	0.0316	0.0567	0.0279	0.0404	0.0361	0.0272	0.0337	0.0356	0.0375	0.0305	0.0273	0.0297	0.0591	0.0335	0.0295	0.0339	0.0369	0.0296	0.0376	0.0341	0.0506	0.0545	0.0464	0.0535	0.0723	0.0424	0.0364	0.1277																	
0.0156	0.0185	0.0226	0.0154	0.0168	0.0350	0.0190	0.0258	0.0095	0.0184	0.0114	0.0090	0.0089	0.0150	0.0185	0.0131	0.0350	0.0196	0.0150	0.0182	0.0221	0.0157	0.0223	0.0173	0.0316	0.0413	0.0271	0.0398	0.0447	0.0355	0.0262	0.1361	0.0360																
0.0209	0.0208	0.0253	0.0208	0.0197	0.0440	0.0213	0.0260	0.0227	0.0208	0.0254	0.0239	0.0243	0.0182	0.0209	0.0174	0.0492	0.0192	0.0167	0.0201	0.0249	0.0189	0.0251	0.0212	0.0406	0.0460	0.0359	0.0431	0.0602	0.0378	0.0315	0.1498	0.0388	0.0239															
0.0368	0.0357	0.0416	0.0366	0.0375	0.0600	0.0337	0.0414	0.0380	0.0332	0.0398	0.0388	0.0400	0.0341	0.0334	0.0327	0.0190	0.0353	0.0341	0.0384	0.0412	0.0330	0.0414	0.0331	0.0399	0.0563	0.0499	0.0136	0.0766	0.0498	0.0426	0.1208	0.0508	0.0400	0.0424														
0.0285	0.0293	0.0383	0.0286	0.0329	0.0544	0.0343	0.0412	0.0315	0.0336	0.0334	0.0328	0.0340	0.0306	0.0338	0.0302	0.0596	0.0348	0.0298	0.0332	0.0375	0.0293	0.0376	0.0343	0.0511	0.0568	0.0481	0.0565	0.0711	0.0480	0.0382	0.1253	0.0149	0.0319	0.0390	0.0544													
0.0160	0.0151	0.0189	0.0160	0.0149	0.0382	0.0156	0.0190	0.0161	0.0150	0.0192	0.0165	0.0179	0.0117	0.0153	0.0107	0.0378	0.0126	0.0122	0.0161	0.0187	0.0126	0.0186	0.0046	0.0340	0.0377	0.0286	0.0321	0.0539	0.0286	0.0238	0.1465	0.0343	0.0181	0.0219	0.0327	0.0343												
0.0147	0.0168	0.0221	0.0146	0.0163	0.0349	0.0185	0.0254	0.0084	0.0180	0.0111	0.0089	0.0081	0.0146	0.0181	0.0140	0.0336	0.0193	0.0155	0.0191	0.0218	0.0132	0.0218	0.0187	0.0307	0.0402	0.0266	0.0390	0.0451	0.0352	0.0259	0.1381	0.0366	0.0096	0.0241	0.0383	0.0328	0.0183											
0.0094	0.0135	0.0186	0.0092	0.0135	0.0338	0.0146	0.0217	0.0130	0.0140	0.0157	0.0136	0.0149	0.0122	0.0141	0.0111	0.0397	0.0144	0.0110	0.0150	0.0186	0.0113	0.0182	0.0159	0.0321	0.0365	0.0314	0.0370	0.0500	0.0320	0.0232	0.1439	0.0304	0.0151	0.0199	0.0349	0.0248	0.0160	0.0138										
0.0139	0.0145	0.0172	0.0138	0.0119	0.0356	0.0138	0.0208	0.0149	0.0131	0.0175	0.0148	0.0159	0.0105	0.0133	0.0083	0.0365	0.0132	0.0102	0.0142	0.0170	0.0108	0.0170	0.0097	0.0316	0.0383	0.0301	0.0310	0.0503	0.0277	0.0239	0.1465	0.0309	0.0157	0.0184	0.0350	0.0327	0.0093	0.0164	0.0122									
0.0122	0.0164	0.0217	0.0120	0.0161	0.0371	0.0179	0.0217	0.0157	0.0172	0.0187	0.0161	0.0153	0.0143	0.0172	0.0131	0.0417	0.0155	0.0145	0.0185	0.0214	0.0131	0.0213	0.0128	0.0333	0.0359	0.0303	0.0385	0.0524	0.0322	0.0234	0.1438	0.0338	0.0158	0.0229	0.0326	0.0287	0.0128	0.0140	0.0097	0.0148								
0.0110	0.0116	0.0175	0.0107	0.0133	0.0346	0.0135	0.0206	0.0129	0.0130	0.0157	0.0133	0.0144	0.0097	0.0131	0.0083	0.0392	0.0143	0.0101	0.0138	0.0165	0.0090	0.0173	0.0109	0.0322	0.0370	0.0315	0.0350	0.0502	0.0307	0.0229	0.1448	0.0290	0.0134	0.0187	0.0332	0.0265	0.0120	0.0133	0.0087	0.0117	0.0100							
0.0147	0.0135	0.0184	0.0146	0.0154	0.0371	0.0092	0.0219	0.0156	0.0091	0.0150	0.0153	0.0158	0.0111	0.0090	0.0107	0.0369	0.0163	0.0124	0.0158	0.0179	0.0090	0.0183	0.0154	0.0312	0.0356	0.0287	0.0323	0.0529	0.0274	0.0202	0.1472	0.0267	0.0180	0.0219	0.0307	0.0318	0.0149	0.0154	0.0130	0.0132	0.0148	0.0122						
0.0151	0.0135	0.0187	0.0150	0.0153	0.0373	0.0095	0.0220	0.0162	0.0088	0.0156	0.0159	0.0162	0.0110	0.0090	0.0110	0.0380	0.0162	0.0124	0.0158	0.0181	0.0088	0.0184	0.0154	0.0307	0.0354	0.0288	0.0326	0.0542	0.0274	0.0204	0.1433	0.0266	0.0184	0.0216	0.0315	0.0319	0.0149	0.0157	0.0130	0.0136	0.0148	0.0121	0.0031					
0.0160	0.0151	0.0193	0.0159	0.0151	0.0386	0.0155	0.0194	0.0164	0.0149	0.0195	0.0165	0.0179	0.0120	0.0149	0.0109	0.0384	0.0131	0.0122	0.0161	0.0189	0.0126	0.0189	0.0033	0.0337	0.0378	0.0289	0.0324	0.0542	0.0287	0.0239	0.1460	0.0343	0.0180	0.0218	0.0329	0.0345	0.0038	0.0186	0.0162	0.0095	0.0130	0.0119	0.0149	0.0150				
0.0095	0.0146	0.0185	0.0094	0.0127	0.0339	0.0150	0.0218	0.0126	0.0145	0.0155	0.0130	0.0128	0.0111	0.0146	0.0107	0.0383	0.0157	0.0118	0.0153	0.0182	0.0113	0.0184	0.0146	0.0311	0.0374	0.0304	0.0352	0.0492	0.0317	0.0225	0.1429	0.0338	0.0132	0.0204	0.0361	0.0270	0.0142	0.0120	0.0087	0.0117	0.0093	0.0092	0.0132	0.0134	0.0145			
0.0177	0.0211	0.0241	0.0175	0.0191	0.0377	0.0209	0.0276	0.0113	0.0200	0.0140	0.0117	0.0125	0.0170	0.0202	0.0164	0.0333	0.0201	0.0174	0.0216	0.0233	0.0174	0.0237	0.0184	0.0338	0.0435	0.0274	0.0381	0.0467	0.0355	0.0283	0.1428	0.0389	0.0136	0.0265	0.0422	0.0369	0.0176	0.0133	0.0160	0.0131	0.0207	0.0182	0.0193	0.0203	0.0182	0.0167		
0.0184	0.0218	0.0259	0.0184	0.0220	0.0389	0.0161	0.0294	0.0119	0.0154	0.0126	0.0123	0.0129	0.0184	0.0155	0.0174	0.0311	0.0229	0.0192	0.0230	0.0253	0.0184	0.0254	0.0178	0.0312	0.0399	0.0235	0.0358	0.0497	0.0323	0.0241	0.1406	0.0347	0.0141	0.0276	0.0385	0.0378	0.0173	0.0139	0.0190	0.0150	0.0198	0.0182	0.0141	0.0147	0.0177	0.0169	0.0114	
0.0136	0.0170	0.0210	0.0134	0.0154	0.0345	0.0170	0.0245	0.0078	0.0165	0.0099	0.0075	0.0070	0.0133	0.0167	0.0121	0.0325	0.0182	0.0147	0.0180	0.0205	0.0138	0.0208	0.0175	0.0310	0.0398	0.0255	0.0380	0.0437	0.0342	0.0248	0.1370	0.0364	0.0082	0.0246	0.0394	0.0335	0.0174	0.0073	0.0143	0.0157	0.0152	0.0130	0.0166	0.0171	0.0174	0.0124	0.0117	0.0135
0.0164	0.0165	0.0193	0.0163	0.0143	0.0392	0.0163	0.0190	0.0177	0.0157	0.0206	0.0178	0.0190	0.0121	0.0158	0.0131	0.0388	0.0116	0.0132	0.0168	0.0187	0.0136	0.0190	0.0100	0.0351	0.0382	0.0295	0.0333	0.0541	0.0294	0.0252	0.1460	0.0342	0.0205	0.0228	0.0351	0.0360	0.0092	0.0199	0.0157	0.0102	0.0159	0.0152	0.0164	0.0166	0.0095	0.0160	0.0162	0.0183

0.0179	0.0221	0.0260	0.0177	0.0222	0.0388	0.0162	0.0292	0.0125	0.0155	0.0122	0.0125	0.0129	0.0182	0.0155	0.0168	0.0306	0.0229	0.0192	0.0227	0.0254	0.0180	0.0254	0.0182	0.0312	0.0400	0.0235	0.0357	0.0497	0.0321	0.0245	0.1399	0.0348	0.0142	0.0278	0.0389	0.0380	0.0178	0.0138	0.0189	0.0144	0.0194	0.0184	0.0137	0.0147	0.0181	0.0175	0.0122	0.0048
0.0172	0.0204	0.0237	0.0171	0.0183	0.0367	0.0207	0.0267	0.0101	0.0199	0.0137	0.0108	0.0114	0.0164	0.0201	0.0158	0.0326	0.0193	0.0164	0.0208	0.0227	0.0165	0.0232	0.0167	0.0324	0.0419	0.0260	0.0378	0.0464	0.0343	0.0281	0.1415	0.0371	0.0124	0.0250	0.0417	0.0362	0.0160	0.0117	0.0161	0.0119	0.0189	0.0166	0.0182	0.0192	0.0163	0.0160	0.0089	0.0097
0.0185	0.0193	0.0252	0.0183	0.0197	0.0381	0.0214	0.0285	0.0116	0.0208	0.0143	0.0113	0.0112	0.0175	0.0210	0.0161	0.0374	0.0220	0.0177	0.0215	0.0245	0.0163	0.0250	0.0202	0.0334	0.0434	0.0296	0.0425	0.0465	0.0379	0.0288	0.1392	0.0360	0.0084	0.0261	0.0411	0.0335	0.0205	0.0108	0.0167	0.0188	0.0168	0.0142	0.0187	0.0190	0.0205	0.0160	0.0161	0.0164
0.0127	0.0131	0.0165	0.0125	0.0099	0.0342	0.0127	0.0193	0.0140	0.0120	0.0165	0.0139	0.0149	0.0100	0.0123	0.0083	0.0396	0.0121	0.0085	0.0126	0.0159	0.0100	0.0162	0.0125	0.0326	0.0369	0.0308	0.0341	0.0499	0.0284	0.0226	0.1448	0.0293	0.0144	0.0170	0.0337	0.0309	0.0127	0.0157	0.0098	0.0086	0.0137	0.0104	0.0120	0.0121	0.0125	0.0119	0.0167	0.0185
0.1702	0.1706	0.1740	0.1702	0.1706	0.1579	0.1700	0.1758	0.1701	0.1695	0.1699	0.1695	0.1708	0.1691	0.1701	0.1692	0.1522	0.1718	0.1695	0.1706	0.1740	0.1700	0.1738	0.1704	0.1585	0.1828	0.1733	0.1523	0.1714	0.1765	0.1717	0.1811	0.1676	0.1696	0.1732	0.1549	0.1659	0.1707	0.1711	0.1697	0.1698	0.1723	0.1698	0.1705	0.1706	0.1706	0.1697	0.1705	0.1699
0.0506	0.0455	0.0547	0.0504	0.0476	0.0738	0.0450	0.0588	0.0522	0.0445	0.0497	0.0526	0.0523	0.0473	0.0450	0.0475	0.0745	0.0519	0.0478	0.0508	0.0548	0.0473	0.0548	0.0518	0.0634	0.0643	0.0593	0.0696	0.0868	0.0606	0.0527	0.1790	0.0622	0.0536	0.0547	0.0701	0.0683	0.0515	0.0521	0.0513	0.0505	0.0519	0.0488	0.0455	0.0450	0.0513	0.0497	0.0569	0.0516
0.0154	0.0181	0.0216	0.0152	0.0173	0.0363	0.0183	0.0250	0.0094	0.0176	0.0120	0.0092	0.0100	0.0145	0.0179	0.0151	0.0295	0.0183	0.0158	0.0191	0.0214	0.0160	0.0211	0.0149	0.0314	0.0412	0.0241	0.0343	0.0481	0.0337	0.0254	0.1396	0.0391	0.0116	0.0258	0.0415	0.0353	0.0143	0.0116	0.0170	0.0136	0.0187	0.0160	0.0185	0.0193	0.0148	0.0151	0.0109	0.0114
0.0904	0.0907	0.0942	0.0901	0.0898	0.1141	0.0875	0.0960	0.0911	0.0871	0.0931	0.0916	0.0928	0.0870	0.0870	0.0868	0.0676	0.0901	0.0880	0.0914	0.0944	0.0874	0.0941	0.0851	0.0953	0.1067	0.1009	0.0625	0.1299	0.0998	0.0946	0.1302	0.1079	0.0943	0.0932	0.0653	0.1107	0.0846	0.0928	0.0907	0.0856	0.0904	0.0886	0.0872	0.0865	0.0847	0.0901	0.0913	0.0898
0.0369	0.0361	0.0431	0.0366	0.0362	0.0602	0.0359	0.0428	0.0397	0.0352	0.0379	0.0392	0.0405	0.0354	0.0356	0.0351	0.0639	0.0355	0.0347	0.0390	0.0421	0.0360	0.0426	0.0366	0.0575	0.0570	0.0482	0.0598	0.0747	0.0476	0.0402	0.1405	0.0303	0.0405	0.0441	0.0567	0.0319	0.0362	0.0414	0.0346	0.0365	0.0353	0.0354	0.0344	0.0350	0.0363	0.0362	0.0415	0.0422
0.0145	0.0174	0.0223	0.0145	0.0171	0.0353	0.0186	0.0258	0.0082	0.0180	0.0111	0.0089	0.0094	0.0152	0.0181	0.0138	0.0350	0.0194	0.0145	0.0183	0.0222	0.0153	0.0220	0.0184	0.0322	0.0403	0.0259	0.0399	0.0471	0.0340	0.0247	0.1388	0.0365	0.0104	0.0237	0.0395	0.0332	0.0179	0.0090	0.0152	0.0163	0.0168	0.0144	0.0168	0.0176	0.0179	0.0142	0.0140	0.0135
0.2999	0.2995	0.3006	0.3001	0.3023	0.2829	0.2993	0.3036	0.3004	0.2994	0.3009	0.3015	0.3017	0.2999	0.3005	0.3006	0.2882	0.3047	0.3005	0.3006	0.3020	0.3008	0.3021	0.3047	0.2824	0.3060	0.3045	0.2887	0.3024	0.3047	0.3043	0.2968	0.2972	0.3020	0.3013	0.2903	0.2967	0.3032	0.3003	0.2996	0.3008	0.3041	0.3008	0.3014	0.2996	0.3035	0.2999	0.2994	0.3008
0.0407	0.0447	0.0488	0.0407	0.0446	0.0475	0.0447	0.0518	0.0372	0.0445	0.0399	0.0379	0.0380	0.0411	0.0444	0.0405	0.0616	0.0457	0.0422	0.0457	0.0483	0.0421	0.0488	0.0444	0.0535	0.0681	0.0542	0.0640	0.0767	0.0619	0.0532	0.1720	0.0657	0.0404	0.0513	0.0678	0.0614	0.0439	0.0392	0.0425	0.0415	0.0447	0.0420	0.0448	0.0452	0.0440	0.0415	0.0401	0.0412
0.0313	0.0342	0.0376	0.0311	0.0325	0.0505	0.0328	0.0408	0.0249	0.0325	0.0266	0.0248	0.0261	0.0305	0.0326	0.0296	0.0291	0.0339	0.0307	0.0345	0.0371	0.0310	0.0376	0.0329	0.0408	0.0546	0.0396	0.0343	0.0592	0.0478	0.0408	0.1327	0.0502	0.0264	0.0391	0.0366	0.0510	0.0321	0.0259	0.0305	0.0279	0.0335	0.0305	0.0320	0.0327	0.0322	0.0301	0.0253	0.0257
0.0138	0.0128	0.0181	0.0136	0.0128	0.0367	0.0143	0.0178	0.0151	0.0141	0.0178	0.0157	0.0151	0.0106	0.0141	0.0103	0.0409	0.0124	0.0116	0.0149	0.0178	0.0094	0.0180	0.0095	0.0318	0.0362	0.0301	0.0351	0.0524	0.0296	0.0230	0.1433	0.0326	0.0162	0.0204	0.0302	0.0313	0.0099	0.0135	0.0131	0.0126	0.0089	0.0105	0.0115	0.0116	0.0094	0.0116	0.0200	0.0208
0.0088	0.0145	0.0182	0.0086	0.0132	0.0340	0.0145	0.0218	0.0130	0.0141	0.0155	0.0133	0.0123	0.0111	0.0142	0.0102	0.0384	0.0159	0.0120	0.0153	0.0185	0.0116	0.0181	0.0155	0.0317	0.0376	0.0306	0.0354	0.0498	0.0318	0.0225	0.1424	0.0345	0.0142	0.0210	0.0369	0.0282	0.0151	0.0124	0.0090	0.0132	0.0096	0.0104	0.0130	0.0130	0.0152	0.0069	0.0175	0.0183
0.0117	0.0153	0.0203	0.0117	0.0172	0.0364	0.0107	0.0239	0.0152	0.0104	0.0147	0.0147	0.0164	0.0139	0.0106	0.0125	0.0379	0.0177	0.0142	0.0178	0.0206	0.0124	0.0201	0.0176	0.0305	0.0345	0.0285	0.0350	0.0536	0.0295	0.0198	0.1440	0.0285	0.0172	0.0235	0.0335	0.0283	0.0172	0.0153	0.0101	0.0152	0.0123	0.0109	0.0088	0.0088	0.0176	0.0096	0.0198	0.0143
0.0158	0.0190	0.0228	0.0157	0.0194	0.0364	0.0130	0.0261	0.0096	0.0127	0.0088	0.0091	0.0107	0.0162	0.0128	0.0143	0.0322	0.0203	0.0165	0.0200	0.0230	0.0166	0.0226	0.0199	0.0306	0.0381	0.0233	0.0377	0.0480	0.0324	0.0219	0.1369	0.0327	0.0114	0.0259	0.0377	0.0356	0.0200	0.0109	0.0165	0.0183	0.0193	0.0160	0.0124	0.0131	0.0199	0.0152	0.0148	0.0097
0.0773	0.0744	0.0851	0.0773	0.0777	0.1002	0.0746	0.0872	0.0723	0.0745	0.0689	0.0719	0.0715	0.0774	0.0748	0.0779	0.0934	0.0810	0.0780	0.0808	0.0852	0.0768	0.0852	0.0808	0.0808	0.0941	0.0743	0.0993	0.1102	0.0880	0.0780	0.1729	0.0929	0.0729	0.0853	0.0990	0.0948	0.0804	0.0709	0.0780	0.0805	0.0770	0.0779	0.0749	0.0735	0.0804	0.0769	0.0769	0.0721
0.0581	0.0528	0.0630	0.0581	0.0542	0.0818	0.0521	0.0642	0.0603	0.0518	0.0573	0.0594	0.0596	0.0541	0.0521	0.0548	0.0817	0.0582	0.0549	0.0583	0.0621	0.0548	0.0631	0.0577	0.0638	0.0734	0.0606	0.0761	0.0978	0.0651	0.0579	0.1820	0.0699	0.0609	0.0632	0.0766	0.0764	0.0573	0.0604	0.0592	0.0577	0.0581	0.0569	0.0531	0.0517	0.0572	0.0575	0.0646	0.0599
0.0147	0.0130	0.0183	0.0146	0.0153	0.0368	0.0090	0.0216	0.0163	0.0087	0.0156	0.0159	0.0163	0.0110	0.0089	0.0109	0.0383	0.0162	0.0124	0.0159	0.0182	0.0089	0.0183	0.0155	0.0309	0.0355	0.0290	0.0326	0.0543	0.0274	0.0202	0.1439	0.0266	0.0183	0.0215	0.0318	0.0321	0.0149	0.0158	0.0129	0.0135	0.0148	0.0120	0.0029	0.0026	0.0151	0.0134	0.0203	0.0149
0.0369	0.0406	0.0442	0.0367	0.0397	0.0570	0.0370	0.0476	0.0311	0.0365	0.0317	0.0308	0.0318	0.0369	0.0366	0.0358	0.0108	0.0416	0.0373	0.0405	0.0442	0.0374	0.0443	0.0412	0.0380	0.0599	0.0453	0.0164	0.0684	0.0542	0.0449	0.1143	0.0567	0.0320	0.0464	0.0168	0.0566	0.0409	0.0304	0.0372	0.0387	0.0391	0.0367	0.0347	0.0364	0.0413	0.0359	0.0357	0.0331
0.0157	0.0191	0.0225	0.0155	0.0176	0.0358	0.0194	0.0255	0.0096	0.0191	0.0130	0.0098	0.0105	0.0154	0.0190	0.0156	0.0318	0.0184	0.0160	0.0200	0.0221	0.0163	0.0225	0.0167	0.0320	0.0417	0.0255	0.0368	0.0462	0.0342	0.0273	0.1407	0.0372	0.0122	0.0253	0.0418	0.0356	0.0159	0.0110	0.0150	0.0121	0.0192	0.0165	0.0178	0.0188	0.0165	0.0150	0.0086	0.0103
0.0148	0.0132	0.0185	0.0149	0.0155	0.0371	0.0092	0.0217	0.0164	0.0090	0.0158	0.0161	0.0163	0.0111	0.0091	0.0110	0.0384	0.0163	0.0125	0.0160	0.0183	0.0090	0.0185	0.0156	0.0309	0.0354	0.0290	0.0327	0.0543	0.0275	0.0203	0.1437	0.0269	0.0184	0.0215	0.0318	0.0323	0.0151	0.0158	0.0131	0.0137	0.0149	0.0122	0.0032	0.0026	0.0152	0.0135	0.0206	0.0148
0.0113	0.0172	0.0211	0.0113	0.0174	0.0360	0.0169	0.0242	0.0153	0.0167	0.0175	0.0151	0.0166	0.0138	0.0167	0.0121	0.0415	0.0183	0.0141	0.0172	0.0208	0.0139	0.0209	0.0173	0.0330	0.0400	0.0334	0.0383	0.0530	0.0348	0.0255	0.1439	0.0349	0.0139	0.0217	0.0384	0.0265	0.0178	0.0166	0.0103	0.0145	0.0129	0.0122	0.0156	0.0156	0.0179	0.0101	0.0193	0.0203
0.0133	0.0157	0.0208	0.0132	0.0173	0.0342	0.0168	0.0242	0.0069	0.0165	0.0099	0.0070	0.0083	0.0139	0.0166	0.0120	0.0333	0.0184	0.0145	0.0178	0.0209	0.0129	0.0207	0.0185	0.0302	0.0390	0.0258	0.0383	0.0456	0.0348	0.0251	0.1387	0.0358	0.0097	0.0235	0.0375	0.0319	0.0180	0.0068	0.0126	0.0154	0.0151	0.0134	0.0144	0.0151	0.0182	0.0133	0.0120	0.0131
0.0127	0.0173	0.0205	0.0126	0.0146	0.0367	0.0167	0.0239	0.0161	0.0163	0.0183	0.0158	0.0154	0.0144	0.0163	0.0129	0.0412	0.0169	0.0140	0.0177	0.0206	0.0148	0.0206	0.0178	0.0338	0.0395	0.0333	0.0384	0.0506	0.0339	0.0257	0.1427	0.0342	0.0134	0.0229	0.0398	0.0275	0.0180	0.0164	0.0091	0.0144	0.0129	0.0123	0.0170	0.0174	0.0182	0.0104	0.0180	0.0213
0.0148	0.0131	0.0183	0.0147	0.0153	0.0369	0.0091	0.0216	0.0163	0.0088	0.0157	0.0159	0.0162	0.0110	0.0090	0.0109	0.0380	0.0162	0.0124	0.0159	0.0182	0.0089	0.0183	0.0156	0.0307	0.0355	0.0290	0.0325	0.0543	0.0275	0.0204	0.1437	0.0266	0.0182	0.0216	0.0316	0.0320	0.0151	0.0156	0.0130	0.0136	0.0149	0.0120	0.0032	0.0027	0.0153	0.0135	0.0204	0.0147
0.0158	0.0185	0.0220	0.0156	0.0178	0.0365	0.0179	0.0257	0.0103	0.0176	0.0118	0.0096	0.0106	0.0145	0.0176	0.0146	0.0299	0.0189	0.0163	0.0195	0.0211	0.0157	0.0219	0.0154	0.0317	0.0415	0.0245	0.0349	0.0474	0.0337	0.0262	0.1403	0.0389	0.0119	0.0260	0.0421	0.0366	0.0153	0.0118	0.0171	0.0131	0.0193	0.0167	0.0188	0.0198	0.0155	0.0159	0.0105	0.0122
0.0128	0.0150	0.0211	0.0127	0.0171	0.0335	0.0168	0.0245	0.0069	0.0165	0.0098	0.0079	0.0085	0.0141	0.0165	0.0124	0.0341	0.0183	0.0138	0.0176	0.0212	0.0130	0.0210	0.0170	0.0300	0.0395	0.0261	0.0389	0.0454	0.0345	0.0251	0.1384	0.0337	0.0085	0.0216	0.0354	0.0306	0.0169	0.0074	0.0113	0.0146	0.0134	0.0114	0.0146	0.0151	0.0171	0.0127	0.0125	0.0118
0.0095	0.0139	0.0190	0.0095	0.0140	0.0348	0.0150	0.0224	0.0136	0.0149	0.0158	0.0135	0.0132	0.0114	0.0148	0.0105	0.0386	0.0165	0.0129	0.0163	0.0186	0.0102	0.0191	0.0156	0.0313	0.0371	0.0315	0.0359	0.0497	0.0321	0.0232	0.1441	0.0327	0.0141	0.0217	0.0351	0.0266	0.0154	0.0112	0.0080	0.0128	0.0085	0.0083	0.0128	0.0131	0.0157	0.0072	0.0170	0.0184
0.0158	0.0148	0.0191	0.0157	0.0154	0.0384	0.0153	0.0191	0.0166	0.0151	0.0197	0.0165	0.0179	0.0121	0.0152	0.0111	0.0384	0.0133	0.0124	0.0164	0.0190	0.0126	0.0191	0.0035	0.0340	0.0379	0.0289	0.0322	0.0543	0.0289	0.0240	0.1464	0.0347	0.0181	0.0218	0.0330	0.0346	0.0040	0.0187	0.0165	0.0097	0.0131	0.0121	0.0151	0.0154	0.0027	0.0147	0.0183	0.0177
0.0684	0.0717	0.0775	0.0680	0.0719	0.0944	0.0702	0.0813	0.0717	0.0697	0.0738	0.0723	0.0715	0.0696	0.0697	0.0687	0.0523	0.0747	0.0703	0.0737	0.0771	0.0687	0.0776	0.0730	0.0735	0.0899	0.0867	0.0497	0.0500	0.0877	0.0782	0.1573	0.0875	0.0703	0.0786	0.0477	0.0825	0.0730	0.0702	0.0653	0.0718	0.0663	0.0652	0.0668	0.0677	0.0733	0.0650	0.0759	0.0733
0.0352	0.0373	0.0422	0.0352	0.0372	0.0229	0.0379	0.0421	0.0340	0.0378	0.0372	0.0350	0.0355	0.0360	0.0379	0.0346	0.0610	0.0351	0.0348	0.0399	0.0424	0.0352	0.0420	0.0349	0.0417	0.0570	0.0487	0.0603	0.0702	0.0534	0.0446	0.1665	0.0551	0.0369	0.0425	0.0544	0.0528	0.0346	0.0348	0.0318	0.0353	0.0317	0.0339	0.0375	0.0371	0.0348	0.0351	0.0344	0.0389
0.0154	0.0159	0.0189	0.0153	0.0139	0.0388	0.0158	0.0188	0.0174	0.0156	0.0203	0.0177	0.0182	0.0124	0.0157	0.0132	0.0387	0.0117	0.0128	0.0168	0.0188	0.0130	0.0189	0.0094	0.0336	0.0378	0.0290	0.0327	0.0542	0.0290	0.0246	0.1454	0.0345	0.0197	0.0219	0.0348	0.0356	0.0089	0.0188	0.0149	0.0094	0.0150	0.0152	0.0152	0.0155	0.0089	0.0150	0.0159	0.0187
0.0157	0.0147	0.0188	0.0155	0.0153	0.0382	0.0152	0.0190	0.0166	0.0151	0.0198	0.0167	0.0180	0.0120	0.0151	0.0110	0.0384	0.0134	0.0124	0.0164	0.0191	0.0127	0.0189	0.0036	0.0336	0.0380	0.0291	0.0322	0.0543	0.0289	0.0241	0.1462	0.0343	0.0181	0.0219	0.0329	0.0346	0.0040	0.0185	0.0163	0.0097	0.0130	0.0118	0.0150	0.0151	0.0026	0.0147	0.0183	0.0179
0.0117	0.0165	0.0196	0.0115	0.0134	0.0354	0.0160	0.0227	0.0149	0.0158	0.0173	0.0144	0.0145	0.0127	0.0157	0.0109	0.0401	0.0157	0.0128	0.0170	0.0192	0.0131	0.0196	0.0162	0.0332	0.0380	0.0319	0.0373	0.0481	0.0323	0.0245	0.1442	0.0324	0.0143	0.0214	0.0376	0.0293	0.0158	0.0147	0.0090	0.0117	0.0112	0.0106	0.0155	0.0158	0.0161	0.0086	0.0163	0.0187
0.0155	0.0146	0.0188	0.0153	0.0151	0.0381	0.0151	0.0190	0.0163	0.0149	0.0194	0.0163	0.0178	0.0118	0.0149	0.0109	0.0381	0.0131	0.0121	0.0162	0.0187	0.0125	0.0187	0.0035	0.0338	0.0379	0.0290	0.0321	0.0543	0.0289	0.0240	0.1464	0.0343	0.0180	0.0217	0.0330	0.0346	0.0038	0.0184	0.0161	0.0095	0.0130	0.0118	0.0148	0.0151	0.0026	0.0144	0.0181	0.0176
0.0158	0.0149	0.0192	0.0156	0.0154	0.0384	0.0154	0.0192	0.0168	0.0152	0.0199	0.0167	0.0180	0.0122	0.0153	0.0112	0.0385	0.0134	0.0124	0.0166	0.0192	0.0129	0.0191	0.0036	0.0336	0.0382	0.0292	0.0324	0.0545	0.0291	0.0242	0.1462	0.0347	0.0182	0.0220	0.0332	0.0347	0.0042	0.0186	0.0165	0.0098	0.0131	0.0120	0.0149	0.0154	0.0027	0.0148	0.0183	0.0180
0.0117	0.0168	0.0201	0.0118	0.0152	0.0357	0.0160	0.0233	0.0155	0.0159	0.0175	0.0152	0.0162	0.0139	0.0159	0.0125	0.0413	0.0164	0.0135	0.0169	0.0202	0.0146	0.0202	0.0174	0.0346	0.0386	0.0330	0.0385	0.0507	0.0336	0.0251	0.1433	0.0339	0.0138	0.0223	0.0394	0.0272	0.0178	0.0172	0.0087	0.0137	0.0139	0.0123	0.0168	0.0170	0.0180	0.0107	0.0178	0.0207
0.0143	0.0174	0.0224	0.0142	0.0176	0.0355	0.0185	0.0260	0.0086	0.0182	0.0112	0.0091	0.0098	0.0154	0.0182	0.0139	0.0354	0.0196	0.0148	0.0186	0.0225	0.0155	0.0222	0.0188	0.0320	0.0407	0.0260	0.0405	0.0473	0.0342	0.0249	0.1389	0.0360	0.0107	0.0236	0.0396	0.0332	0.0183	0.0096	0.0153	0.0167	0.0173	0.0142	0.0172	0.0181	0.0186	0.0145	0.0141	0.0139
0.0138	0.0156	0.0208	0.0136	0.0176	0.0339	0.0178	0.0243	0.0075	0.0175	0.0103	0.0080	0.0085	0.0134	0.0176	0.0137	0.0335	0.0185	0.0150	0.0180	0.0209	0.0121	0.0207	0.0177	0.0304	0.0397	0.0260	0.0384	0.0460	0.0347	0.0252	0.1384	0.0352	0.0100	0.0235	0.0380	0.0305	0.0172	0.0075	0.0119	0.0161	0.0146	0.0126	0.0125	0.0125	0.0176	0.0131	0.0125	0.0131
0.0485	0.0510	0.0561	0.0486	0.0506	0.0697	0.0518	0.0598	0.0428	0.0516	0.0444	0.0417	0.0434	0.0486	0.0516	0.0461	0.0694	0.0533	0.0472	0.0498	0.0564	0.0491	0.0561	0.0521	0.0689	0.0726	0.0603	0.0745	0.0221	0.0693	0.0600	0.1755	0.0698	0.0429	0.0568	0.0745	0.0661	0.0518	0.0443	0.0484	0.0506	0.0512	0.0478	0.0509	0.0519	0.0524	0.0487	0.0478	0.0485
0.0337	0.0381	0.0431	0.0333	0.0348	0.0586	0.0389	0.0456	0.0374	0.0385	0.0394	0.0381	0.0370	0.0359	0.0385	0.0349	0.0637	0.0383	0.0350	0.0389	0.0424	0.0363	0.0424	0.0392	0.0569	0.0611	0.0532	0.0611	0.0728	0.0539	0.0451	0.1429	0.0386	0.0351	0.0440	0.0615	0.0314	0.0393	0.0376	0.0306	0.0356	0.0336	0.0337	0.0389	0.0388	0.0395	0.0311	0.0395	0.0430
0.0083	0.0121	0.0176	0.0083	0.0142	0.0339	0.0138	0.0211	0.0123	0.0136	0.0150	0.0124	0.0139	0.0105	0.0138	0.0088	0.0380	0.0148	0.0110	0.0148	0.0178	0.0097	0.0175	0.0144	0.0314	0.0367	0.0308	0.0352	0.0506	0.0318	0.0228	0.1440	0.0311	0.0143	0.0202	0.0340	0.0264	0.0143	0.0124	0.0075	0.0124	0.0088	0.0073	0.0123	0.0126	0.0146	0.0085	0.0176	0.0174
0.0162	0.0182	0.0231	0.0162	0.0172	0.0372	0.0190	0.0267	0.0105	0.0188	0.0120	0.0102	0.0109	0.0152	0.0189	0.0156	0.0304	0.0203	0.0147	0.0183	0.0218	0.0164	0.0229	0.0166	0.0328	0.0426	0.0247	0.0358	0.0486	0.0338	0.0262	0.1400	0.0393	0.0125	0.0258	0.0430	0.0361	0.0163	0.0123	0.0181	0.0145	0.0200	0.0173	0.0195	0.0202	0.0167	0.0167	0.0114	0.0125
0.0694	0.0748	0.0810	0.0691	0.0730	0.1005	0.0764	0.0802	0.0745	0.0760	0.0760	0.0748	0.0739	0.0727	0.0755	0.0709	0.1055	0.0725	0.0718	0.0763	0.0803	0.0733	0.0806	0.0739	0.1027	0.1013	0.0892	0.1012	0.1169	0.0912	0.0813	0.1773	0.0768	0.0726	0.0786	0.0991	0.0688	0.0743	0.0765	0.0679	0.0726	0.0683	0.0693	0.0763	0.0763	0.0743	0.0683	0.0781	0.0795
0.0989	0.1003	0.1080	0.0989	0.1015	0.1231	0.1025	0.1089	0.1006	0.1018	0.1024	0.1011	0.1013	0.1003	0.1019	0.1004	0.1172	0.1021	0.1000	0.1037	0.1075	0.1003	0.1073	0.1032	0.1155	0.1200	0.1109	0.1160	0.1415	0.1146	0.1058	0.1780	0.0977	0.1021	0.1073	0.1176	0.0929	0.1025	0.1020	0.0972	0.0995	0.1009	0.0989	0.1030	0.1016	0.1025	0.0986	0.1007	0.1027
0.0559	0.0589	0.0643	0.0559	0.0590	0.0784	0.0595	0.0669	0.0576	0.0591	0.0590	0.0578	0.0579	0.0567	0.0590	0.0558	0.0769	0.0598	0.0570	0.0604	0.0639	0.0572	0.0642	0.0590	0.0747	0.0802	0.0718	0.0759	0.0954	0.0740	0.0661	0.1785	0.0735	0.0582	0.0652	0.0770	0.0698	0.0590	0.0589	0.0537	0.0556	0.0578	0.0550	0.0597	0.0592	0.0592	0.0549	0.0581	0.0595
0.0895	0.0892	0.0939	0.0897	0.0886	0.1134	0.0899	0.0962	0.0925	0.0899	0.0949	0.0923	0.0919	0.0869	0.0898	0.0856	0.1196	0.0888	0.0868	0.0899	0.0945	0.0861	0.0942	0.0909	0.1158	0.1128	0.1080	0.1136	0.0716	0.1078	0.1017	0.2351	0.1087	0.0920	0.0917	0.1123	0.1082	0.0907	0.0912	0.0865	0.0862	0.0911	0.0857	0.0890	0.0887	0.0906	0.0891	0.0937	0.0975
0.0789	0.0792	0.0820	0.0790	0.0771	0.1016	0.0749	0.0821	0.0803	0.0750	0.0822	0.0805	0.0808	0.0750	0.0749	0.0745	0.0584	0.0753	0.0756	0.0796	0.0822	0.0753	0.0825	0.0717	0.0845	0.0941	0.0885	0.0529	0.0488	0.0875	0.0840	0.1690	0.0935	0.0815	0.0837	0.0526	0.0987	0.0708	0.0813	0.0773	0.0715	0.0767	0.0766	0.0736	0.0740	0.0714	0.0773	0.0763	0.0763
0.0778	0.0749	0.0815	0.0777	0.0760	0.1012	0.0778	0.0810	0.0789	0.0774	0.0815	0.0794	0.0797	0.0733	0.0778	0.0734	0.1017	0.0754	0.0738	0.0773	0.0809	0.0740	0.0814	0.0699	0.0993	0.0969	0.0880	0.0961	0.0510	0.0868	0.0824	0.1791	0.0685	0.0790	0.0827	0.0967	0.0658	0.0692	0.0804	0.0776	0.0723	0.0753	0.0751	0.0766	0.0763	0.0698	0.0764	0.0761	0.0795
0.0087	0.0146	0.0180	0.0087	0.0133	0.0340	0.0139	0.0214	0.0127	0.0138	0.0155	0.0133	0.0142	0.0114	0.0139	0.0098	0.0386	0.0142	0.0117	0.0156	0.0175	0.0118	0.0181	0.0157	0.0314	0.0363	0.0305	0.0361	0.0496	0.0312	0.0222	0.1446	0.0324	0.0151	0.0204	0.0367	0.0271	0.0155	0.0145	0.0071	0.0108	0.0116	0.0105	0.0138	0.0146	0.0156	0.0083	0.0154	0.0185
0.0164	0.0161	0.0191	0.0164	0.0142	0.0383	0.0157	0.0189	0.0172	0.0156	0.0200	0.0173	0.0189	0.0123	0.0156	0.0115	0.0380	0.0122	0.0125	0.0170	0.0185	0.0129	0.0190	0.0092	0.0343	0.0381	0.0295	0.0324	0.0520	0.0287	0.0251	0.1489	0.0326	0.0188	0.0220	0.0327	0.0349	0.0082	0.0188	0.0149	0.0088	0.0145	0.0136	0.0149	0.0155	0.0085	0.0145	0.0156	0.0173
0.0134	0.0140	0.0176	0.0132	0.0119	0.0358	0.0138	0.0177	0.0147	0.0133	0.0166	0.0142	0.0142	0.0099	0.0135	0.0064	0.0395	0.0115	0.0109	0.0142	0.0174	0.0104	0.0176	0.0088	0.0330	0.0366	0.0289	0.0345	0.0505	0.0290	0.0226	0.1432	0.0330	0.0143	0.0199	0.0310	0.0330	0.0085	0.0147	0.0139	0.0102	0.0099	0.0109	0.0130	0.0129	0.0088	0.0113	0.0186	0.0196
0.0137	0.0125	0.0188	0.0137	0.0126	0.0388	0.0083	0.0219	0.0166	0.0080	0.0148	0.0161	0.0157	0.0102	0.0081	0.0112	0.0378	0.0159	0.0122	0.0154	0.0178	0.0115	0.0187	0.0156	0.0335	0.0367	0.0277	0.0325	0.0536	0.0271	0.0199	0.1446	0.0280	0.0180	0.0227	0.0336	0.0340	0.0157	0.0176	0.0154	0.0150	0.0163	0.0134	0.0091	0.0091	0.0155	0.0143	0.0215	0.0166
0.0338	0.0377	0.0408	0.0338	0.0359	0.0219	0.0368	0.0444	0.0328	0.0367	0.0358	0.0339	0.0339	0.0342	0.0368	0.0342	0.0555	0.0372	0.0341	0.0385	0.0407	0.0349	0.0408	0.0346	0.0404	0.0601	0.0485	0.0550	0.0686	0.0522	0.0447	0.1675	0.0563	0.0361	0.0431	0.0601	0.0535	0.0344	0.0344	0.0326	0.0312	0.0370	0.0346	0.0376	0.0375	0.0347	0.0331	0.0288	0.0342
0.0922	0.0948	0.0916	0.0921	0.0923	0.1081	0.0909	0.0937	0.0944	0.0908	0.0955	0.0935	0.0933	0.0911	0.0908	0.0902	0.0758	0.0935	0.0915	0.0917	0.0921	0.0919	0.0917	0.0939	0.1048	0.1033	0.1049	0.0732	0.0619	0.0998	0.0954	0.1851	0.1112	0.0941	0.1007	0.0714	0.1134	0.0924	0.0935	0.0914	0.0925	0.0912	0.0910	0.0927	0.0905	0.0927	0.0897	0.0969	0.0950
0.0171	0.0210	0.0238	0.0169	0.0176	0.0359	0.0203	0.0273	0.0119	0.0202	0.0140	0.0118	0.0112	0.0173	0.0202	0.0157	0.0373	0.0202	0.0172	0.0209	0.0239	0.0175	0.0239	0.0206	0.0339	0.0422	0.0289	0.0415	0.0390	0.0365	0.0282	0.1396	0.0372	0.0110	0.0252	0.0412	0.0367	0.0204	0.0114	0.0157	0.0153	0.0179	0.0169	0.0191	0.0198	0.0206	0.0149	0.0118	0.0148
0.0483	0.0521	0.0565	0.0482	0.0519	0.0405	0.0485	0.0598	0.0473	0.0484	0.0491	0.0484	0.0489	0.0483	0.0482	0.0483	0.0258	0.0536	0.0493	0.0530	0.0558	0.0486	0.0565	0.0485	0.0399	0.0732	0.0606	0.0255	0.0866	0.0648	0.0569	0.1418	0.0692	0.0502	0.0588	0.0300	0.0684	0.0483	0.0491	0.0492	0.0465	0.0512	0.0489	0.0482	0.0484	0.0483	0.0476	0.0477	0.0457
0.0142	0.0178	0.0207	0.0140	0.0150	0.0337	0.0174	0.0243	0.0083	0.0170	0.0113	0.0085	0.0080	0.0141	0.0172	0.0123	0.0339	0.0170	0.0142	0.0183	0.0205	0.0142	0.0207	0.0177	0.0307	0.0396	0.0261	0.0385	0.0413	0.0337	0.0253	0.1394	0.0341	0.0078	0.0225	0.0381	0.0336	0.0173	0.0085	0.0129	0.0121	0.0150	0.0135	0.0158	0.0167	0.0177	0.0121	0.0088	0.0112
0.0187	0.0224	0.0121	0.0186	0.0212	0.0271	0.0211	0.0162	0.0133	0.0208	0.0162	0.0134	0.0142	0.0194	0.0208	0.0189	0.0389	0.0222	0.0184	0.0159	0.0126	0.0204	0.0117	0.0233	0.0386	0.0361	0.0307	0.0437	0.0516	0.0306	0.0301	0.1521	0.0430	0.0164	0.0298	0.0454	0.0405	0.0234	0.0156	0.0200	0.0209	0.0231	0.0205	0.0226	0.0236	0.0231	0.0198	0.0183	0.0197
0.1226	0.1269	0.1299	0.1219	0.1232	0.1451	0.1223	0.1313	0.1155	0.1212	0.1174	0.1156	0.1162	0.1220	0.1216	0.1215	0.0927	0.1227	0.1225	0.1264	0.1291	0.1226	0.1281	0.1207	0.1319	0.1382	0.1233	0.0981	0.0831	0.1344	0.1269	0.1263	0.1427	0.1177	0.1320	0.1007	0.1455	0.1195	0.1171	0.1215	0.1186	0.1230	0.1230	0.1268	0.1219	0.1199	0.1221	0.1146	0.1125
0.0110	0.0152	0.0204	0.0112	0.0174	0.0370	0.0107	0.0239	0.0148	0.0105	0.0149	0.0154	0.0166	0.0135	0.0106	0.0123	0.0377	0.0182	0.0143	0.0178	0.0202	0.0123	0.0205	0.0177	0.0306	0.0347	0.0288	0.0346	0.0534	0.0298	0.0195	0.1447	0.0284	0.0170	0.0232	0.0330	0.0278	0.0178	0.0155	0.0095	0.0153	0.0124	0.0107	0.0087	0.0091	0.0180	0.0100	0.0193	0.0143
0.0134	0.0112	0.0171	0.0133	0.0064	0.0358	0.0130	0.0201	0.0152	0.0125	0.0168	0.0153	0.0162	0.0102	0.0128	0.0093	0.0403	0.0131	0.0079	0.0116	0.0166	0.0109	0.0168	0.0137	0.0337	0.0375	0.0305	0.0348	0.0513	0.0281	0.0227	0.1461	0.0291	0.0164	0.0177	0.0355	0.0316	0.0135	0.0169	0.0123	0.0104	0.0157	0.0116	0.0137	0.0139	0.0134	0.0134	0.0188	0.0205
0.0145	0.0178	0.0220	0.0143	0.0166	0.0352	0.0183	0.0256	0.0083	0.0176	0.0112	0.0086	0.0097	0.0152	0.0177	0.0133	0.0351	0.0184	0.0145	0.0185	0.0217	0.0152	0.0217	0.0182	0.0325	0.0401	0.0262	0.0403	0.0466	0.0338	0.0250	0.1398	0.0357	0.0106	0.0228	0.0393	0.0335	0.0178	0.0091	0.0142	0.0154	0.0168	0.0138	0.0171	0.0178	0.0181	0.0144	0.0129	0.0135
0.0134	0.0111	0.0169	0.0133	0.0059	0.0355	0.0129	0.0199	0.0150	0.0125	0.0169	0.0152	0.0164	0.0101	0.0127	0.0094	0.0402	0.0130	0.0075	0.0118	0.0163	0.0108	0.0168	0.0140	0.0333	0.0378	0.0305	0.0347	0.0518	0.0281	0.0223	0.1450	0.0295	0.0166	0.0182	0.0356	0.0318	0.0137	0.0170	0.0122	0.0107	0.0162	0.0118	0.0138	0.0140	0.0136	0.0134	0.0187	0.0209
0.0143	0.0168	0.0207	0.0141	0.0152	0.0342	0.0170	0.0245	0.0081	0.0166	0.0101	0.0081	0.0078	0.0133	0.0168	0.0125	0.0325	0.0181	0.0144	0.0177	0.0203	0.0140	0.0206	0.0174	0.0304	0.0404	0.0258	0.0377	0.0445	0.0348	0.0244	0.1380	0.0371	0.0078	0.0240	0.0385	0.0333	0.0170	0.0080	0.0146	0.0152	0.0157	0.0133	0.0163	0.0169	0.0173	0.0117	0.0109	0.0123
0.0175	0.0197	0.0241	0.0173	0.0153	0.0382	0.0204	0.0275	0.0115	0.0199	0.0140	0.0116	0.0121	0.0170	0.0201	0.0173	0.0313	0.0200	0.0157	0.0190	0.0236	0.0181	0.0238	0.0176	0.0334	0.0420	0.0252	0.0366	0.0491	0.0333	0.0263	0.1410	0.0395	0.0137	0.0261	0.0434	0.0374	0.0172	0.0133	0.0179	0.0154	0.0208	0.0182	0.0205	0.0213	0.0176	0.0174	0.0118	0.0133
0.0175	0.0151	0.0210	0.0173	0.0164	0.0396	0.0168	0.0206	0.0181	0.0164	0.0210	0.0183	0.0196	0.0135	0.0165	0.0123	0.0396	0.0146	0.0135	0.0173	0.0204	0.0127	0.0206	0.0049	0.0337	0.0387	0.0305	0.0336	0.0555	0.0304	0.0257	0.1474	0.0340	0.0190	0.0227	0.0329	0.0340	0.0055	0.0185	0.0156	0.0111	0.0127	0.0114	0.0151	0.0153	0.0044	0.0159	0.0198	0.0192
0.0157	0.0149	0.0191	0.0155	0.0150	0.0383	0.0153	0.0192	0.0164	0.0148	0.0195	0.0164	0.0178	0.0118	0.0150	0.0107	0.0380	0.0130	0.0121	0.0162	0.0188	0.0125	0.0189	0.0032	0.0339	0.0379	0.0289	0.0321	0.0542	0.0289	0.0240	0.1462	0.0345	0.0181	0.0217	0.0328	0.0343	0.0037	0.0183	0.0162	0.0095	0.0130	0.0117	0.0149	0.0149	0.0023	0.0145	0.0181	0.0176
0.0211	0.0237	0.0187	0.0209	0.0214	0.0309	0.0248	0.0220	0.0149	0.0242	0.0179	0.0157	0.0155	0.0210	0.0244	0.0204	0.0406	0.0248	0.0212	0.0201	0.0178	0.0200	0.0183	0.0255	0.0366	0.0344	0.0326	0.0460	0.0496	0.0275	0.0311	0.1463	0.0419	0.0160	0.0304	0.0450	0.0396	0.0253	0.0135	0.0192	0.0211	0.0215	0.0200	0.0221	0.0228	0.0256	0.0192	0.0173	0.0199
0.0212	0.0237	0.0188	0.0211	0.0214	0.0309	0.0249	0.0219	0.0150	0.0243	0.0181	0.0158	0.0154	0.0209	0.0246	0.0203	0.0406	0.0248	0.0212	0.0199	0.0177	0.0199	0.0183	0.0256	0.0365	0.0341	0.0325	0.0460	0.0497	0.0273	0.0309	0.1458	0.0420	0.0159	0.0304	0.0450	0.0396	0.0252	0.0135	0.0191	0.0211	0.0214	0.0201	0.0222	0.0228	0.0256	0.0190	0.0174	0.0199
0.0158	0.0185	0.0231	0.0156	0.0166	0.0353	0.0192	0.0263	0.0096	0.0187	0.0116	0.0093	0.0089	0.0154	0.0188	0.0134	0.0347	0.0200	0.0153	0.0186	0.0225	0.0159	0.0227	0.0178	0.0315	0.0416	0.0273	0.0399	0.0450	0.0356	0.0263	0.1362	0.0366	0.0029	0.0239	0.0401	0.0364	0.0185	0.0094	0.0154	0.0160	0.0161	0.0138	0.0181	0.0185	0.0186	0.0134	0.0138	0.0141
0.0149	0.0189	0.0222	0.0148	0.0170	0.0362	0.0194	0.0259	0.0100	0.0190	0.0125	0.0099	0.0106	0.0153	0.0191	0.0155	0.0303	0.0198	0.0166	0.0189	0.0224	0.0166	0.0220	0.0157	0.0318	0.0417	0.0250	0.0353	0.0482	0.0340	0.0261	0.1396	0.0399	0.0125	0.0261	0.0421	0.0364	0.0157	0.0115	0.0173	0.0147	0.0192	0.0164	0.0191	0.0199	0.0156	0.0159	0.0110	0.0119
0.0128	0.0130	0.0170	0.0127	0.0112	0.0365	0.0135	0.0204	0.0148	0.0130	0.0172	0.0149	0.0156	0.0094	0.0132	0.0101	0.0357	0.0141	0.0110	0.0140	0.0166	0.0108	0.0167	0.0102	0.0320	0.0389	0.0300	0.0302	0.0538	0.0286	0.0231	0.1457	0.0337	0.0175	0.0207	0.0366	0.0332	0.0101	0.0168	0.0145	0.0092	0.0164	0.0120	0.0143	0.0144	0.0100	0.0132	0.0159	0.0168

0.0716	0.0709	0.0773	0.0715	0.0686	0.0943	0.0735	0.0783	0.0729	0.0731	0.0747	0.0728	0.0727	0.0698	0.0733	0.0701	0.0988	0.0704	0.0693	0.0732	0.0768	0.0705	0.0772	0.0701	0.0929	0.0897	0.0834	0.0959	0.0431	0.0824	0.0725	0.1724	0.0629	0.0724	0.0787	0.0936	0.0607	0.0701	0.0731	0.0699	0.0711	0.0691	0.0694	0.0727	0.0738	0.0699	0.0697	0.0750	0.0778
0.0559	0.0565	0.0602	0.0555	0.0548	0.0791	0.0548	0.0527	0.0617	0.0578	0.0524	0.0591	0.0582	0.0589	0.0532	0.0523	0.0531	0.0348	0.0551	0.0535	0.0566	0.0604	0.0540	0.0604	0.0535	0.0601	0.0786	0.0678	0.0299	0.0943	0.0689	0.0635	0.1404	0.0721	0.0579	0.0588	0.0331	0.0733	0.0540	0.0595	0.0550	0.0518	0.0589	0.0548	0.0529	0.0536	0.0557	0.0586	0.0571
0.1556	0.1582	0.1626	0.1556	0.1548	0.1438	0.1548	0.1650	0.1557	0.1549	0.1567	0.1551	0.1559	0.1551	0.1548	0.1541	0.1302	0.1570	0.1531	0.1569	0.1632	0.1561	0.1634	0.1558	0.1441	0.1751	0.1633	0.1302	0.1219	0.1699	0.1637	0.2470	0.1745	0.1555	0.1589	0.1347	0.1747	0.1547	0.1562	0.1544	0.1516	0.1589	0.1550	0.1530	0.1555	0.1549	0.1547	0.1500	0.1515
0.0151	0.0146	0.0188	0.0149	0.0090	0.0382	0.0160	0.0226	0.0175	0.0155	0.0193	0.0177	0.0183	0.0128	0.0155	0.0123	0.0384	0.0155	0.0107	0.0137	0.0189	0.0133	0.0185	0.0127	0.0342	0.0400	0.0305	0.0332	0.0535	0.0283	0.0246	0.1466	0.0328	0.0191	0.0206	0.0389	0.0345	0.0121	0.0188	0.0148	0.0092	0.0182	0.0142	0.0159	0.0166	0.0123	0.0153	0.0167	0.0187
0.0173	0.0189	0.0235	0.0172	0.0155	0.0374	0.0196	0.0266	0.0116	0.0189	0.0132	0.0112	0.0117	0.0161	0.0192	0.0164	0.0307	0.0192	0.0145	0.0183	0.0222	0.0175	0.0231	0.0173	0.0329	0.0418	0.0248	0.0367	0.0482	0.0333	0.0264	0.1410	0.0381	0.0134	0.0258	0.0435	0.0369	0.0170	0.0135	0.0170	0.0134	0.0209	0.0181	0.0202	0.0211	0.0173	0.0172	0.0105	0.0133
0.0168	0.0185	0.0232	0.0165	0.0149	0.0376	0.0192	0.0263	0.0110	0.0186	0.0130	0.0106	0.0111	0.0154	0.0188	0.0155	0.0316	0.0185	0.0139	0.0178	0.0220	0.0167	0.0229	0.0163	0.0330	0.0427	0.0247	0.0372	0.0488	0.0335	0.0265	0.1457	0.0382	0.0129	0.0255	0.0436	0.0368	0.0158	0.0130	0.0164	0.0124	0.0197	0.0174	0.0198	0.0203	0.0162	0.0164	0.0095	0.0129
0.0151	0.0172	0.0225	0.0151	0.0134	0.0354	0.0184	0.0252	0.0096	0.0178	0.0112	0.0094	0.0097	0.0142	0.0180	0.0140	0.0337	0.0189	0.0128	0.0160	0.0212	0.0145	0.0220	0.0182	0.0323	0.0404	0.0259	0.0387	0.0473	0.0337	0.0241	0.1385	0.0368	0.0101	0.0233	0.0407	0.0332	0.0182	0.0108	0.0156	0.0170	0.0177	0.0147	0.0174	0.0177	0.0183	0.0148	0.0142	0.0151
0.0155	0.0201	0.0219	0.0151	0.0155	0.0370	0.0198	0.0261	0.0108	0.0191	0.0136	0.0111	0.0121	0.0165	0.0194	0.0165	0.0313	0.0189	0.0166	0.0197	0.0225	0.0176	0.0216	0.0170	0.0320	0.0414	0.0253	0.0368	0.0478	0.0336	0.0266	0.1404	0.0386	0.0135	0.0261	0.0430	0.0371	0.0167	0.0127	0.0165	0.0137	0.0205	0.0175	0.0199	0.0209	0.0167	0.0172	0.0106	0.0134
0.0142	0.0121	0.0173	0.0141	0.0028	0.0361	0.0136	0.0206	0.0155	0.0135	0.0175	0.0161	0.0150	0.0106	0.0134	0.0110	0.0394	0.0134	0.0091	0.0129	0.0168	0.0118	0.0174	0.0149	0.0328	0.0381	0.0303	0.0345	0.0508	0.0283	0.0225	0.1449	0.0317	0.0163	0.0196	0.0372	0.0329	0.0147	0.0160	0.0135	0.0115	0.0159	0.0130	0.0148	0.0152	0.0148	0.0128	0.0184	0.0213
0.0807	0.0793	0.0773	0.0806	0.0797	0.0583	0.0797	0.0807	0.0799	0.0793	0.0790	0.0797	0.0795	0.0797	0.0794	0.0799	0.0604	0.0844	0.0788	0.0777	0.0769	0.0790	0.0770	0.0842	0.0692	0.0907	0.0906	0.0608	0.1158	0.0811	0.0835	0.1335	0.0680	0.0781	0.0896	0.0597	0.0687	0.0845	0.0776	0.0782	0.0812	0.0805	0.0785	0.0774	0.0782	0.0844	0.0783	0.0829	0.0811
0.0282	0.0339	0.0381	0.0282	0.0339	0.0541	0.0338	0.0411	0.0316	0.0337	0.0344	0.0324	0.0330	0.0304	0.0337	0.0293	0.0543	0.0353	0.0316	0.0350	0.0376	0.0309	0.0382	0.0311	0.0516	0.0548	0.0484	0.0517	0.0701	0.0496	0.0413	0.1444	0.0532	0.0334	0.0405	0.0560	0.0459	0.0305	0.0335	0.0273	0.0282	0.0306	0.0289	0.0338	0.0334	0.0307	0.0268	0.0319	0.0335
0.0134	0.0143	0.0180	0.0134	0.0116	0.0378	0.0145	0.0212	0.0162	0.0139	0.0179	0.0157	0.0165	0.0105	0.0142	0.0105	0.0362	0.0141	0.0118	0.0143	0.0176	0.0116	0.0178	0.0114	0.0324	0.0393	0.0300	0.0298	0.0529	0.0289	0.0242	0.1456	0.0337	0.0178	0.0209	0.0372	0.0344	0.0112	0.0178	0.0146	0.0087	0.0171	0.0137	0.0143	0.0147	0.0113	0.0141	0.0156	0.0177
0.0153	0.0191	0.0219	0.0151	0.0171	0.0351	0.0186	0.0254	0.0095	0.0184	0.0122	0.0096	0.0101	0.0154	0.0184	0.0142	0.0308	0.0184	0.0158	0.0194	0.0219	0.0159	0.0222	0.0161	0.0310	0.0417	0.0255	0.0360	0.0440	0.0336	0.0263	0.1408	0.0372	0.0111	0.0249	0.0403	0.0349	0.0157	0.0108	0.0139	0.0110	0.0186	0.0160	0.0174	0.0185	0.0161	0.0146	0.0060	0.0101
0.0160	0.0159	0.0189	0.0156	0.0140	0.0388	0.0160	0.0187	0.0178	0.0158	0.0206	0.0179	0.0188	0.0122	0.0160	0.0129	0.0390	0.0119	0.0132	0.0168	0.0188	0.0136	0.0188	0.0102	0.0349	0.0383	0.0294	0.0332	0.0543	0.0296	0.0254	0.1463	0.0343	0.0203	0.0227	0.0352	0.0360	0.0094	0.0198	0.0156	0.0103	0.0160	0.0151	0.0160	0.0166	0.0099	0.0160	0.0163	0.0187
0.0133	0.0127	0.0171	0.0133	0.0133	0.0356	0.0134	0.0208	0.0154	0.0133	0.0171	0.0145	0.0155	0.0104	0.0134	0.0083	0.0360	0.0150	0.0111	0.0143	0.0173	0.0097	0.0173	0.0101	0.0309	0.0391	0.0309	0.0304	0.0525	0.0294	0.0242	0.1459	0.0317	0.0161	0.0212	0.0349	0.0320	0.0101	0.0146	0.0126	0.0079	0.0146	0.0108	0.0118	0.0119	0.0102	0.0129	0.0156	0.0169
0.0485	0.0482	0.0522	0.0485	0.0478	0.0717	0.0446	0.0550	0.0500	0.0447	0.0511	0.0497	0.0512	0.0442	0.0446	0.0444	0.0263	0.0494	0.0457	0.0494	0.0516	0.0450	0.0526	0.0455	0.0529	0.0713	0.0614	0.0203	0.0884	0.0604	0.0554	0.1342	0.0651	0.0521	0.0540	0.0262	0.0686	0.0449	0.0511	0.0488	0.0435	0.0511	0.0468	0.0446	0.0446	0.0451	0.0474	0.0470	0.0473
0.0166	0.0193	0.0241	0.0167	0.0198	0.0368	0.0204	0.0254	0.0101	0.0203	0.0138	0.0109	0.0113	0.0165	0.0203	0.0154	0.0318	0.0196	0.0170	0.0207	0.0236	0.0162	0.0239	0.0120	0.0315	0.0411	0.0248	0.0369	0.0480	0.0343	0.0268	0.1404	0.0379	0.0114	0.0257	0.0383	0.0346	0.0123	0.0107	0.0166	0.0138	0.0159	0.0143	0.0181	0.0188	0.0124	0.0159	0.0116	0.0110
0.0166	0.0142	0.0202	0.0165	0.0159	0.0388	0.0160	0.0198	0.0171	0.0158	0.0204	0.0176	0.0190	0.0126	0.0159	0.0114	0.0389	0.0140	0.0129	0.0168	0.0196	0.0119	0.0201	0.0044	0.0337	0.0383	0.0300	0.0331	0.0549	0.0298	0.0251	0.1472	0.0332	0.0182	0.0221	0.0322	0.0333	0.0051	0.0176	0.0152	0.0105	0.0123	0.0105	0.0146	0.0147	0.0043	0.0154	0.0189	0.0184
0.3872	0.3871	0.3880	0.3869	0.3872	0.3668	0.3863	0.3877	0.3867	0.3860	0.3871	0.3867	0.3874	0.3859	0.3863	0.3869	0.3626	0.3876	0.3864	0.3873	0.3878	0.3866	0.3870	0.3869	0.3715	0.3866	0.3863	0.3626	0.3485	0.3876	0.3864	0.2900	0.3761	0.3860	0.3847	0.3631	0.3752	0.3863	0.3865	0.3857	0.3872	0.3874	0.3867	0.3916	0.3861	0.3866	0.3863	0.3839	0.3867
0.0195	0.0222	0.0257	0.0194	0.0193	0.0388	0.0161	0.0290	0.0136	0.0160	0.0129	0.0130	0.0131	0.0191	0.0160	0.0180	0.0349	0.0217	0.0191	0.0226	0.0254	0.0197	0.0258	0.0226	0.0328	0.0398	0.0261	0.0399	0.0466	0.0337	0.0254	0.1392	0.0329	0.0128	0.0285	0.0415	0.0387	0.0230	0.0138	0.0176	0.0194	0.0211	0.0186	0.0156	0.0161	0.0231	0.0182	0.0162	0.0130
0.0450	0.0454	0.0520	0.0448	0.0474	0.0513	0.0478	0.0548	0.0438	0.0477	0.0466	0.0440	0.0446	0.0430	0.0475	0.0443	0.0660	0.0491	0.0451	0.0488	0.0511	0.0434	0.0526	0.0451	0.0605	0.0723	0.0605	0.0664	0.0838	0.0647	0.0561	0.1852	0.0676	0.0462	0.0547	0.0703	0.0636	0.0444	0.0437	0.0440	0.0433	0.0467	0.0430	0.0488	0.0468	0.0448	0.0437	0.0434	0.0451
0.0518	0.0570	0.0508	0.0517	0.0549	0.0669	0.0558	0.0542	0.0559	0.0555	0.0573	0.0556	0.0564	0.0533	0.0555	0.0525	0.0544	0.0567	0.0536	0.0525	0.0502	0.0534	0.0508	0.0536	0.0683	0.0658	0.0692	0.0512	0.0924	0.0587	0.0639	0.1647	0.0740	0.0550	0.0620	0.0572	0.0696	0.0539	0.0569	0.0496	0.0499	0.0535	0.0521	0.0551	0.0555	0.0546	0.0498	0.0546	0.0562
0.0154	0.0184	0.0223	0.0152	0.0175	0.0358	0.0184	0.0252	0.0095	0.0178	0.0117	0.0090	0.0101	0.0146	0.0180	0.0141	0.0283	0.0190	0.0156	0.0193	0.0216	0.0152	0.0219	0.0150	0.0308	0.0412	0.0243	0.0348	0.0478	0.0336	0.0257	0.1399	0.0382	0.0116	0.0256	0.0407	0.0354	0.0144	0.0106	0.0161	0.0134	0.0174	0.0156	0.0173	0.0179	0.0148	0.0150	0.0102	0.0107
0.0823	0.0859	0.0931	0.0823	0.0846	0.1096	0.0853	0.0962	0.0871	0.0848	0.0872	0.0869	0.0864	0.0843	0.0847	0.0833	0.0673	0.0898	0.0846	0.0880	0.0927	0.0845	0.0931	0.0882	0.0920	0.1062	0.0986	0.0646	0.0680	0.0989	0.0894	0.1375	0.0685	0.0851	0.0937	0.0647	0.0641	0.0879	0.0865	0.0811	0.0862	0.0824	0.0818	0.0833	0.0837	0.0884	0.0802	0.0909	0.0881
0.0360	0.0403	0.0461	0.0359	0.0426	0.0618	0.0384	0.0494	0.0403	0.0384	0.0420	0.0407	0.0417	0.0389	0.0384	0.0371	0.0219	0.0437	0.0393	0.0432	0.0463	0.0377	0.0463	0.0424	0.0434	0.0613	0.0551	0.0191	0.0785	0.0563	0.0470	0.1266	0.0552	0.0417	0.0461	0.0156	0.0529	0.0422	0.0406	0.0345	0.0396	0.0357	0.0347	0.0361	0.0367	0.0427	0.0349	0.0454	0.0412
0.1223	0.1257	0.1303	0.1219	0.1206	0.1096	0.1224	0.1303	0.1221	0.1222	0.1234	0.1222	0.1216	0.1225	0.1220	0.1209	0.1014	0.1220	0.1196	0.1241	0.1303	0.1235	0.1297	0.1240	0.1158	0.1436	0.1309	0.1019	0.1589	0.1359	0.1271	0.1917	0.1411	0.1218	0.1269	0.0987	0.1424	0.1239	0.1221	0.1205	0.1212	0.1208	0.1220	0.1229	0.1221	0.1240	0.1208	0.1239	0.1224
0.0163	0.0195	0.0224	0.0162	0.0172	0.0358	0.0190	0.0258	0.0109	0.0189	0.0130	0.0103	0.0114	0.0161	0.0190	0.0144	0.0316	0.0191	0.0160	0.0198	0.0226	0.0166	0.0224	0.0154	0.0325	0.0411	0.0259	0.0370	0.0455	0.0332	0.0271	0.1417	0.0361	0.0114	0.0247	0.0411	0.0363	0.0153	0.0113	0.0154	0.0116	0.0185	0.0153	0.0184	0.0193	0.0153	0.0154	0.0094	0.0111
0.0415	0.0406	0.0451	0.0417	0.0415	0.0578	0.0440	0.0459	0.0413	0.0437	0.0413	0.0409	0.0417	0.0413	0.0439	0.0413	0.0603	0.0426	0.0417	0.0429	0.0450	0.0418	0.0450	0.0419	0.0569	0.0488	0.0399	0.0605	0.0764	0.0480	0.0442	0.1550	0.0607	0.0427	0.0487	0.0620	0.0590	0.0415	0.0422	0.0425	0.0412	0.0428	0.0423	0.0448	0.0438	0.0417	0.0419	0.0433	0.0434
0.0164	0.0197	0.0227	0.0164	0.0161	0.0361	0.0193	0.0258	0.0107	0.0190	0.0132	0.0106	0.0115	0.0164	0.0192	0.0153	0.0314	0.0190	0.0164	0.0205	0.0228	0.0175	0.0227	0.0164	0.0321	0.0413	0.0260	0.0365	0.0466	0.0333	0.0258	0.1406	0.0379	0.0124	0.0263	0.0420	0.0371	0.0160	0.0117	0.0157	0.0135	0.0193	0.0167	0.0189	0.0200	0.0162	0.0163	0.0103	0.0128
0.0174	0.0212	0.0241	0.0173	0.0207	0.0388	0.0214	0.0248	0.0126	0.0212	0.0148	0.0124	0.0131	0.0181	0.0214	0.0167	0.0332	0.0192	0.0190	0.0215	0.0252	0.0187	0.0242	0.0126	0.0336	0.0408	0.0244	0.0387	0.0498	0.0347	0.0271	0.1400	0.0404	0.0132	0.0273	0.0387	0.0380	0.0125	0.0136	0.0197	0.0160	0.0165	0.0172	0.0205	0.0215	0.0125	0.0176	0.0135	0.0131
0.0100	0.0157	0.0190	0.0098	0.0118	0.0339	0.0151	0.0222	0.0140	0.0149	0.0165	0.0144	0.0140	0.0125	0.0150	0.0115	0.0398	0.0149	0.0119	0.0160	0.0190	0.0125	0.0189	0.0162	0.0324	0.0373	0.0316	0.0369	0.0468	0.0317	0.0236	0.1435	0.0325	0.0146	0.0207	0.0373	0.0277	0.0159	0.0133	0.0072	0.0113	0.0105	0.0106	0.0144	0.0147	0.0161	0.0067	0.0163	0.0189
0.0396	0.0434	0.0465	0.0395	0.0402	0.0597	0.0392	0.0500	0.0338	0.0390	0.0354	0.0346	0.0334	0.0400	0.0390	0.0393	0.0192	0.0430	0.0397	0.0431	0.0467	0.0409	0.0468	0.0439	0.0417	0.0623	0.0483	0.0246	0.0691	0.0561	0.0478	0.1200	0.0577	0.0315	0.0478	0.0248	0.0569	0.0441	0.0340	0.0377	0.0397	0.0413	0.0398	0.0393	0.0399	0.0443	0.0381	0.0367	0.0360
0.0518	0.0552	0.0588	0.0517	0.0537	0.0718	0.0514	0.0626	0.0466	0.0513	0.0468	0.0457	0.0456	0.0517	0.0514	0.0511	0.0262	0.0566	0.0525	0.0558	0.0590	0.0524	0.0589	0.0560	0.0559	0.0751	0.0594	0.0318	0.0811	0.0690	0.0597	0.1271	0.0722	0.0469	0.0602	0.0335	0.0726	0.0556	0.0455	0.0524	0.0537	0.0542	0.0519	0.0512	0.0513	0.0558	0.0500	0.0479	0.0476
0.0149	0.0142	0.0184	0.0146	0.0141	0.0374	0.0149	0.0187	0.0156	0.0145	0.0187	0.0154	0.0168	0.0114	0.0146	0.0108	0.0374	0.0121	0.0120	0.0151	0.0184	0.0123	0.0181	0.0046	0.0338	0.0377	0.0279	0.0314	0.0547	0.0287	0.0233	0.1457	0.0350	0.0179	0.0223	0.0337	0.0338	0.0048	0.0179	0.0155	0.0099	0.0134	0.0125	0.0148	0.0150	0.0040	0.0139	0.0173	0.0178
0.0223	0.0278	0.0219	0.0223	0.0245	0.0374	0.0212	0.0214	0.0260	0.0211	0.0255	0.0261	0.0257	0.0243	0.0212	0.0235	0.0475	0.0244	0.0246	0.0235	0.0211	0.0245	0.0218	0.0246	0.0399	0.0299	0.0339	0.0451	0.0614	0.0256	0.0280	0.1518	0.0403	0.0267	0.0329	0.0421	0.0416	0.0241	0.0260	0.0209	0.0244	0.0187	0.0234	0.0203	0.0209	0.0240	0.0198	0.0291	0.0255
0.0735	0.0728	0.0790	0.0733	0.0709	0.0959	0.0754	0.0794	0.0749	0.0754	0.0770	0.0746	0.0744	0.0719	0.0754	0.0719	0.1008	0.0717	0.0715	0.0751	0.0785	0.0724	0.0787	0.0713	0.0931	0.0909	0.0847	0.0979	0.0431	0.0842	0.0743	0.1740	0.0649	0.0742	0.0806	0.0953	0.0628	0.0712	0.0750	0.0718	0.0729	0.0702	0.0713	0.0742	0.0757	0.0711	0.0714	0.0771	0.0797
0.0529	0.0555	0.0633	0.0528	0.0579	0.0796	0.0557	0.0664	0.0572	0.0552	0.0576	0.0580	0.0593	0.0556	0.0551	0.0547	0.0392	0.0604	0.0550	0.0587	0.0626	0.0559	0.0632	0.0603	0.0618	0.0797	0.0698	0.0365	0.0949	0.0699	0.0599	0.1048	0.0390	0.0567	0.0639	0.0358	0.0322	0.0604	0.0591	0.0514	0.0575	0.0556	0.0538	0.0550	0.0554	0.0605	0.0523	0.0617	0.0598
0.0100	0.0106	0.0143	0.0099	0.0112	0.0333	0.0103	0.0174	0.0122	0.0102	0.0137	0.0111	0.0132	0.0070	0.0103	0.0021	0.0372	0.0117	0.0078	0.0111	0.0144	0.0079	0.0145	0.0106	0.0304	0.0371	0.0302	0.0322	0.0489	0.0288	0.0219	0.1445	0.0293	0.0130	0.0174	0.0325	0.0301	0.0106	0.0137	0.0110	0.0081	0.0130	0.0080	0.0105	0.0110	0.0110	0.0106	0.0163	0.0174
0.0146	0.0150	0.0189	0.0145	0.0144	0.0378	0.0152	0.0194	0.0166	0.0150	0.0191	0.0167	0.0170	0.0120	0.0151	0.0109	0.0413	0.0132	0.0126	0.0161	0.0187	0.0119	0.0189	0.0113	0.0342	0.0359	0.0298	0.0359	0.0553	0.0287	0.0222	0.1450	0.0348	0.0185	0.0212	0.0325	0.0344	0.0112	0.0171	0.0155	0.0138	0.0125	0.0132	0.0146	0.0146	0.0112	0.0146	0.0218	0.0217
0.0101	0.0107	0.0150	0.0101	0.0109	0.0336	0.0104	0.0182	0.0122	0.0103	0.0144	0.0130	0.0131	0.0078	0.0103	0.0074	0.0381	0.0125	0.0085	0.0119	0.0148	0.0081	0.0152	0.0126	0.0311	0.0370	0.0304	0.0317	0.0512	0.0286	0.0216	0.1433	0.0305	0.0152	0.0169	0.0328	0.0296	0.0122	0.0136	0.0106	0.0101	0.0132	0.0102	0.0105	0.0105	0.0125	0.0102	0.0172	0.0176
0.0102	0.0106	0.0150	0.0102	0.0108	0.0338	0.0105	0.0182	0.0125	0.0104	0.0145	0.0132	0.0133	0.0077	0.0104	0.0074	0.0381	0.0127	0.0086	0.0118	0.0148	0.0080	0.0152	0.0127	0.0316	0.0369	0.0305	0.0319	0.0515	0.0286	0.0217	0.1437	0.0309	0.0154	0.0172	0.0329	0.0298	0.0126	0.0138	0.0111	0.0102	0.0133	0.0102	0.0109	0.0110	0.0128	0.0103	0.0174	0.0181
0.0839	0.0857	0.0925	0.0835	0.0839	0.1071	0.0845	0.0935	0.0774	0.0839	0.0783	0.0777	0.0785	0.0846	0.0839	0.0838	0.0583	0.0855	0.0831	0.0879	0.0919	0.0852	0.0916	0.0874	0.0918	0.1080	0.0882	0.0640	0.1173	0.0967	0.0870	0.1042	0.0660	0.0796	0.0938	0.0611	0.0671	0.0866	0.0791	0.0818	0.0847	0.0848	0.0843	0.0867	0.0842	0.0874	0.0839	0.0807	0.0806
0.0143	0.0172	0.0222	0.0142	0.0173	0.0354	0.0182	0.0257	0.0085	0.0181	0.0112	0.0089	0.0097	0.0154	0.0182	0.0137	0.0352	0.0196	0.0147	0.0187	0.0224	0.0157	0.0222	0.0186	0.0322	0.0409	0.0262	0.0403	0.0471	0.0342	0.0248	0.1397	0.0363	0.0104	0.0235	0.0395	0.0333	0.0182	0.0094	0.0152	0.0167	0.0173	0.0140	0.0170	0.0179	0.0185	0.0143	0.0140	0.0137
0.0142	0.0181	0.0220	0.0142	0.0181	0.0350	0.0182	0.0256	0.0084	0.0181	0.0114	0.0090	0.0099	0.0150	0.0181	0.0134	0.0348	0.0195	0.0152	0.0188	0.0221	0.0152	0.0221	0.0183	0.0321	0.0406	0.0267	0.0401	0.0471	0.0350	0.0255	0.1385	0.0362	0.0095	0.0233	0.0392	0.0329	0.0182	0.0093	0.0147	0.0167	0.0169	0.0134	0.0173	0.0180	0.0185	0.0144	0.0139	0.0138
0.0163	0.0193	0.0234	0.0162	0.0192	0.0371	0.0133	0.0266	0.0107	0.0128	0.0095	0.0103	0.0109	0.0154	0.0131	0.0156	0.0315	0.0205	0.0171	0.0201	0.0224	0.0161	0.0232	0.0203	0.0315	0.0381	0.0233	0.0357	0.0485	0.0326	0.0229	0.1376	0.0335	0.0126	0.0267	0.0389	0.0361	0.0202	0.0121	0.0167	0.0187	0.0194	0.0168	0.0128	0.0131	0.0206	0.0163	0.0150	0.0095
0.0411	0.0421	0.0470	0.0410	0.0381	0.0625	0.0434	0.0488	0.0391	0.0434	0.0411	0.0393	0.0399	0.0401	0.0434	0.0404	0.0637	0.0415	0.0389	0.0425	0.0466	0.0410	0.0470	0.0425	0.0606	0.0626	0.0520	0.0649	0.0420	0.0558	0.0466	0.1535	0.0479	0.0402	0.0489	0.0650	0.0464	0.0422	0.0406	0.0405	0.0416	0.0421	0.0405	0.0429	0.0441	0.0423	0.0408	0.0421	0.0446
0.0160	0.0150	0.0193	0.0158	0.0155	0.0385	0.0155	0.0193	0.0171	0.0154	0.0201	0.0170	0.0183	0.0125	0.0155	0.0112	0.0387	0.0136	0.0128	0.0167	0.0194	0.0132	0.0194	0.0040	0.0337	0.0384	0.0296	0.0325	0.0545	0.0293	0.0245	0.1468	0.0347	0.0185	0.0223	0.0333	0.0346	0.0045	0.0188	0.0165	0.0101	0.0134	0.0122	0.0149	0.0156	0.0032	0.0151	0.0187	0.0182
0.0156	0.0179	0.0218	0.0153	0.0122	0.0359	0.0187	0.0253	0.0107	0.0187	0.0128	0.0109	0.0117	0.0155	0.0187	0.0158	0.0345	0.0182	0.0137	0.0167	0.0216	0.0165	0.0218	0.0200	0.0331	0.0407	0.0261	0.0396	0.0476	0.0335	0.0254	0.1385	0.0368	0.0126	0.0242	0.0423	0.0358	0.0196	0.0126	0.0159	0.0170	0.0199	0.0168	0.0187	0.0195	0.0199	0.0164	0.0143	0.0168
0.0133	0.0109	0.0168	0.0132	0.0042	0.0356	0.0128	0.0200	0.0152	0.0127	0.0172	0.0158	0.0161	0.0094	0.0127	0.0101	0.0397	0.0128	0.0083	0.0123	0.0160	0.0107	0.0169	0.0145	0.0329	0.0379	0.0299	0.0338	0.0521	0.0281	0.0222	0.1443	0.0309	0.0174	0.0191	0.0365	0.0322	0.0140	0.0170	0.0128	0.0114	0.0166	0.0127	0.0138	0.0139	0.0141	0.0136	0.0185	0.0215
0.0163	0.0194	0.0224	0.0162	0.0157	0.0367	0.0191	0.0258	0.0107	0.0190	0.0136	0.0112	0.0117	0.0158	0.0192	0.0164	0.0308	0.0189	0.0163	0.0203	0.0221	0.0170	0.0223	0.0167	0.0318	0.0409	0.0255	0.0363	0.0476	0.0338	0.0265	0.1410	0.0386	0.0131	0.0264	0.0430	0.0369	0.0165	0.0126	0.0165	0.0136	0.0201	0.0174	0.0196	0.0208	0.0165	0.0166	0.0103	0.0131
0.0145	0.0188	0.0221	0.0145	0.0172	0.0362	0.0192	0.0258	0.0100	0.0192	0.0127	0.0101	0.0107	0.0153	0.0192	0.0157	0.0301	0.0197	0.0164	0.0189	0.0225	0.0166	0.0221	0.0157	0.0320	0.0419	0.0249	0.0354	0.0486	0.0342	0.0262	0.1392	0.0397	0.0123	0.0260	0.0421	0.0363	0.0158	0.0114	0.0173	0.0149	0.0194	0.0165	0.0191	0.0200	0.0155	0.0160	0.0116	0.0125
0.0161	0.0161	0.0192	0.0161	0.0141	0.0389	0.0160	0.0185	0.0178	0.0159	0.0208	0.0180	0.0189	0.0122	0.0160	0.0131	0.0389	0.0119	0.0135	0.0170	0.0189	0.0137	0.0191	0.0102	0.0352	0.0383	0.0296	0.0334	0.0542	0.0296	0.0253	0.1459	0.0345	0.0205	0.0229	0.0352	0.0364	0.0095	0.0197	0.0161	0.0104	0.0159	0.0153	0.0163	0.0167	0.0098	0.0162	0.0165	0.0187
0.0166	0.0184	0.0242	0.0164	0.0199	0.0373	0.0198	0.0275	0.0110	0.0196	0.0126	0.0100	0.0117	0.0169	0.0196	0.0149	0.0368	0.0215	0.0174	0.0202	0.0244	0.0161	0.0241	0.0209	0.0341	0.0430	0.0295	0.0421	0.0462	0.0383	0.0284	0.1412	0.0369	0.0121	0.0264	0.0413	0.0344	0.0211	0.0114	0.0152	0.0192	0.0185	0.0155	0.0181	0.0185	0.0214	0.0171	0.0161	0.0171
0.0237	0.0258	0.0312	0.0236	0.0258	0.0469	0.0239	0.0329	0.0242	0.0237	0.0242	0.0246	0.0249	0.0243	0.0237	0.0235	0.0233	0.0268	0.0266	0.0238	0.0275	0.0311	0.0239	0.0310	0.0262	0.0369	0.0459	0.0358	0.0259	0.0617	0.0380	0.0299	0.1287	0.0409	0.0260	0.0323	0.0236	0.0410	0.0262	0.0244	0.0225	0.0248	0.0239	0.0224	0.0223	0.0231	0.0265	0.0228	0.0282	0.0263
0.0543	0.0571	0.0602	0.0543	0.0569	0.0735	0.0585	0.0629	0.0486	0.0581	0.0514	0.0487	0.0489	0.0545	0.0581	0.0533	0.0747	0.0579	0.0550	0.0569	0.0601	0.0546	0.0601	0.0588	0.0702	0.0789	0.0652	0.0800	0.0653	0.0743	0.0667	0.1842	0.0759	0.0483	0.0626	0.0799	0.0722	0.0584	0.0484	0.0537	0.0551	0.0564	0.0532	0.0548	0.0567	0.0588	0.0538	0.0521	0.0534
0.0628	0.0637	0.0716	0.0626	0.0655	0.0837	0.0637	0.0743	0.0654	0.0568	0.0631	0.0570	0.0575	0.0586	0.0632	0.0633	0.0627	0.0385	0.0686	0.0629	0.0662	0.0704	0.0634	0.0712	0.0678	0.0664	0.0872	0.0693	0.0434	0.0949	0.0776	0.0678	0.1041	0.0465	0.0590	0.0711	0.0425	0.0453	0.0676	0.0584	0.0625	0.0652	0.0659	0.0629	0.0617	0.0627	0.0680	0.0623	0.0594	0.0589
0.0301	0.0273	0.0354	0.0303	0.0294	0.0539	0.0308	0.0382	0.0316	0.0307	0.0338	0.0332	0.0336	0.0278	0.0306	0.0275	0.0590	0.0326	0.0271	0.0307	0.0351	0.0284	0.0353	0.0315	0.0521	0.0581	0.0479	0.0533	0.0712	0.0457	0.0380	0.1253	0.0154	0.0320	0.0360	0.0530	0.0121	0.0320	0.0339	0.0298	0.0302	0.0331	0.0288	0.0309	0.0309	0.0320	0.0298	0.0366	0.0379
0.0489	0.0507	0.0555	0.0489	0.0505	0.0388	0.0472	0.0591	0.0481	0.0471	0.0493	0.0481	0.0488	0.0487	0.0472	0.0475	0.0581	0.0519	0.0488	0.0531	0.0554	0.0477	0.0558	0.0526	0.0444	0.0690	0.0610	0.0583	0.0843	0.0642	0.0559	0.1655	0.0639	0.0499	0.0554	0.0565	0.0674	0.0523	0.0475	0.0459	0.0482	0.0495	0.0471	0.0474	0.0457	0.0526	0.0482	0.0501	0.0484
0.0339	0.0323	0.0385	0.0339	0.0339	0.0527	0.0343	0.0398	0.0344	0.0343	0.0373	0.0346	0.0360	0.0301	0.0341	0.0302	0.0562	0.0345	0.0311	0.0352	0.0378	0.0302	0.0387	0.0273	0.0507	0.0583	0.0491	0.0517	0.0727	0.0496	0.0438	0.1679	0.0526	0.0359	0.0406	0.0540	0.0513	0.0277	0.0347	0.0329	0.0292	0.0332	0.0295	0.0331	0.0333	0.0272	0.0323	0.0353	0.0361
0.0293	0.0312	0.0380	0.0293	0.0313	0.0544	0.0334	0.0408	0.0333	0.0332	0.0337	0.0331	0.0346	0.0310	0.0332	0.0302	0.0597	0.0340	0.0298	0.0337	0.0373	0.0318	0.0377	0.0352	0.0532	0.0570	0.0485	0.0570	0.0695	0.0478	0.0390	0.1265	0.0161	0.0328	0.0393	0.0576	0.0103	0.0354	0.0355	0.0263	0.0317	0.0316	0.0296	0.0349	0.0351	0.0355	0.0290	0.0361	0.0395
0.0155	0.0191	0.0231	0.0154	0.0194	0.0362	0.0194	0.0265	0.0094	0.0192	0.0127	0.0101	0.0104	0.0160	0.0193	0.0143	0.0321	0.0205	0.0166	0.0205	0.0230	0.0161	0.0232	0.0159	0.0318	0.0423	0.0261	0.0367	0.0467	0.0349	0.0273	0.1414	0.0377	0.0103	0.0245	0.0398	0.0349	0.0151	0.0111	0.0158	0.0126	0.0176	0.0154	0.0178	0.0187	0.0157	0.0150	0.0095	0.0096
0.0353	0.0396	0.0454	0.0352	0.0416	0.0613	0.0378	0.0489	0.0397	0.0377	0.0414	0.0407	0.0411	0.0383	0.0381	0.0377	0.0368	0.0220	0.0431	0.0384	0.0423	0.0453	0.0370	0.0458	0.0420	0.0426	0.0606	0.0545	0.0190	0.0777	0.0558	0.0463	0.1261	0.0550	0.0412	0.0452	0.0157	0.0522	0.0419	0.0399	0.0339	0.0392	0.0354	0.0344	0.0361	0.0365	0.0422	0.0346	0.0447	0.0411
0.1075	0.1044	0.1054	0.1074	0.1064	0.0877	0.1102	0.1088	0.1054	0.1099	0.1066	0.1060	0.1065	0.1065	0.1100	0.1058	0.1313	0.1100	0.1058	0.1051	0.1051	0.1062	0.1050	0.1074	0.1181	0.1173	0.1161	0.1311	0.1429	0.1123	0.1147	0.2245	0.1264	0.1070	0.1132	0.1330	0.1249	0.1067	0.1067	0.1060	0.1051	0.1078	0.1055	0.1109	0.1095	0.1074	0.1058	0.1061	0.1090
0.0398	0.0400	0.0440	0.0396	0.0373	0.0620	0.0365	0.0471	0.0422	0.0365	0.0433	0.0426	0.0419	0.0371	0.0367	0.0368	0.0222	0.0407	0.0368	0.0410	0.0438	0.0376	0.0446	0.0411	0.0440	0.0623	0.0561	0.0170	0.0748	0.0541	0.0479	0.1215	0.0533	0.0399	0.0448	0.0164	0.0571	0.0415	0.0414	0.0375	0.0372	0.0410	0.0380	0.0363	0.0365	0.0416	0.0378	0.0440	0.0439
0.1399	0.1367	0.1356	0.1400	0.1385	0.1174	0.1426	0.1376	0.1380	0.1427	0.1392	0.1386	0.1399	0.1394	0.1428	0.1387	0.1646	0.1407	0.1386	0.1383	0.1362	0.1393	0.1352	0.1386	0.1465	0.1450	0.1470	0.1647	0.1780	0.1397	0.1455	0.2151	0.1597	0.1400	0.1460	0.1648	0.1587	0.1379	0.1396	0.1387	0.1380	0.1389	0.1390	0.1431	0.1427	0.1386	0.1390	0.1386	0.1420
0.0786	0.0801	0.0863	0.0788	0.0819	0.1003	0.0835	0.0885	0.0735	0.0834	0.0755	0.0737	0.0753	0.0797	0.0835	0.0779	0.1010	0.0834	0.0794	0.0819	0.0868	0.0803	0.0866	0.0821	0.0993	0.1023	0.0882	0.1062	0.0463	0.0957	0.0874	0.1718	0.0669	0.0736	0.0889	0.1049	0.0616	0.0819	0.0751	0.0796	0.0816	0.0806	0.0793	0.0816	0.0830	0.0824	0.0796	0.0784	0.0790

FC21	HL38	CL33	HL39	CL34	CL35	CL36	CL37	CL38	CL39	CL40	FC22	CL41	CL42	CL43	HL40	HL41	HL42	HL43	CL44	CL45	HL44	FC23	HL45	CL46	CL47	CL48	CL49	CL50	CL51	CL52	CL53	CL54	FC24	CL55	CL56	CL57	CL58	CL59	CL60	CL61	CL62	CL63	CL64	FC25	CL65	HL46	BL10	BL11	BL12

[redacted]

0.0187 [redacted]

0.0133	0.0194	
0.0116	0.0143	0.0106	
0.0103	0.0231	0.0165	0.0145	
0.0147	0.0134	0.0188	0.0153	0.0166	
0.1699	0.1721	0.1698	0.1701	0.1702	0.1698	
0.0519	0.0525	0.0512	0.0565	0.0552	0.0495	0.1998	
0.0108	0.0160	0.0115	0.0096	0.0141	0.0166	0.1695	0.0548	
0.0925	0.0855	0.0897	0.0909	0.0954	0.0880	0.1862	0.1120	0.0904	
0.0398	0.0355	0.0417	0.0415	0.0423	0.0352	0.1597	0.0698	0.0420	0.1103	
0.0090	0.0207	0.0139	0.0117	0.0128	0.0152	0.1702	0.0543	0.0121	0.0931	0.0411	
0.3008	0.3040	0.3004	0.3000	0.3000	0.3010	0.2299	0.2841	0.3012	0.2957	0.2862	0.3013	
0.0386	0.0448	0.0418	0.0395	0.0429	0.0427	0.1820	0.0818	0.0388	0.1196	0.0690	0.0399	0.3151	
0.0253	0.0305	0.0261	0.0210	0.0287	0.0298	0.1620	0.0695	0.0256	0.0807	0.0545	0.0259	0.2998	0.0541	
0.0153	0.0122	0.0207	0.0191	0.0181	0.0117	0.1721	0.0494	0.0181	0.0867	0.0352	0.0176	0.3026	0.0437	0.0332	
0.0123	0.0167	0.0182	0.0169	0.0172	0.0122	0.1701	0.0497	0.0158	0.0898	0.0357	0.0145	0.3003	0.0416	0.0311	0.0119	
0.0162	0.0190	0.0146	0.0192	0.0186	0.0145	0.1703	0.0468	0.0180	0.0886	0.0355	0.0170	0.2996	0.0443	0.0320	0.0145	0.0110	
0.0101	0.0213	0.0101	0.0140	0.0141	0.0171	0.1697	0.0489	0.0128	0.0915	0.0389	0.0117	0.2990	0.0412	0.0263	0.0184	0.0160	0.0108	
0.0720	0.0806	0.0715	0.0756	0.0747	0.0790	0.2161	0.0907	0.0738	0.1534	0.0957	0.0740	0.2621	0.1043	0.0887	0.0760	0.0766	0.0736	0.0684	
0.0592	0.0584	0.0590	0.0635	0.0632	0.0550	0.2120	0.0696	0.0620	0.1291	0.0751	0.0616	0.2728	0.0896	0.0768	0.0546	0.0567	0.0550	0.0566	0.0327	
0.0171	0.0166	0.0146	0.0193	0.0192	0.0120	0.1707	0.0450	0.0191	0.0864	0.0353	0.0175	0.2993	0.0449	0.0326	0.0114	0.0127	0.0085	0.0128	0.0734	0.0514	
0.0301	0.0421	0.0330	0.0347	0.0342	0.0378	0.1521	0.0733	0.0336	0.0695	0.0621	0.0312	0.2869	0.0621	0.0286	0.0388	0.0361	0.0355	0.0298	0.0925	0.0806	0.0364	
0.0106	0.0133	0.0109	0.0048	0.0149	0.0158	0.1702	0.0562	0.0087	0.0904	0.0407	0.0119	0.2999	0.0387	0.0217	0.0183	0.0158	0.0182	0.0127	0.0751	0.0628	0.0185	0.0339	
0.0172	0.0168	0.0148	0.0195	0.0194	0.0122	0.1710	0.0452	0.0193	0.0863	0.0353	0.0177	0.2995	0.0451	0.0326	0.0114	0.0129	0.0087	0.0129	0.0736	0.0518	0.0022	0.0365	0.0186	
0.0163	0.0193	0.0202	0.0195	0.0182	0.0132	0.1701	0.0539	0.0185	0.0920	0.0377	0.0176	0.3014	0.0423	0.0331	0.0153	0.0110	0.0124	0.0179	0.0800	0.0611	0.0152	0.0383	0.0185	0.0154	
0.0076	0.0194	0.0133	0.0119	0.0112	0.0143	0.1705	0.0531	0.0114	0.0923	0.0401	0.0093	0.2999	0.0381	0.0252	0.0142	0.0129	0.0142	0.0096	0.0714	0.0604	0.0148	0.0302	0.0107	0.0150	0.0148	
0.0149	0.0169	0.0213	0.0177	0.0173	0.0132	0.1693	0.0528	0.0186	0.0919	0.0355	0.0185	0.3004	0.0449	0.0315	0.0153	0.0108	0.0141	0.0187	0.0788	0.0600	0.0171	0.0385	0.0167	0.0174	0.0103	0.0167	
0.0170	0.0167	0.0146	0.0193	0.0192	0.0121	0.1707	0.0449	0.0192	0.0862	0.0351	0.0174	0.2987	0.0452	0.0326	0.0114	0.0129	0.0085	0.0128	0.0733	0.0513	0.0022	0.0361	0.0185	0.0023	0.0152	0.0148	0.0169	
0.0106	0.0168	0.0118	0.0099	0.0148	0.0169	0.1696	0.0545	0.0071	0.0904	0.0421	0.0127	0.2999	0.0385	0.0251	0.0183	0.0161	0.0186	0.0134	0.0739	0.0622	0.0193	0.0334	0.0088	0.0195	0.0183	0.0110	0.0180	0.0192	
0.0085	0.0194	0.0122	0.0107	0.0094	0.0131	0.1699	0.0531	0.0110	0.0929	0.0402	0.0079	0.2994	0.0383	0.0245	0.0143	0.0132	0.0140	0.0099	0.0722	0.0609	0.0147	0.0310	0.0106	0.0148	0.0149	0.0062	0.0172	0.0148	0.0116	
0.0111	0.0169	0.0185	0.0173	0.0152	0.0124	0.1706	0.0495	0.0165	0.0897	0.0368	0.0151	0.2995	0.0415	0.0312	0.0106	0.0074	0.0098	0.0166	0.0763	0.0573	0.0128	0.0357	0.0162	0.0129	0.0102	0.0113	0.0104	0.0127	0.0163	0.0124	
0.0175	0.0097	0.0181	0.0165	0.0207	0.0127	0.1706	0.0513	0.0149	0.0846	0.0365	0.0181	0.3030	0.0441	0.0322	0.0093	0.0153	0.0176	0.0197	0.0803	0.0571	0.0151	0.0412	0.0164	0.0152	0.0178	0.0181	0.0182	0.0153	0.0153	0.0170	0.0156	
0.0707	0.0750	0.0737	0.0754	0.0689	0.0694	0.1568	0.1031	0.0746	0.1030	0.0935	0.0730	0.2966	0.1020	0.0708	0.0698	0.0663	0.0647	0.0717	0.1338	0.1147	0.0678	0.0484	0.0748	0.0678	0.0666	0.0714	0.0659	0.0676	0.0752	0.0702	0.0642	0.0731	
0.0358	0.0347	0.0388	0.0360	0.0368	0.0340	0.1568	0.0748	0.0375	0.1112	0.0556	0.0357	0.2855	0.0483	0.0499	0.0324	0.0352	0.0365	0.0383	0.0975	0.0816	0.0368	0.0591	0.0364	0.0372	0.0370	0.0335	0.0361	0.0371	0.0375	0.0323	0.0343	0.0347	0.0933	
0.0184	0.0055	0.0191	0.0150	0.0227	0.0126	0.1718	0.0522	0.0156	0.0860	0.0357	0.0196	0.3028	0.0443	0.0317	0.0110	0.0153	0.0179	0.0207	0.0792	0.0576	0.0153	0.0419	0.0132	0.0155	0.0186	0.0186	0.0171	0.0154	0.0158	0.0184	0.0160	0.0088	0.0741	0.0341	
0.0174	0.0098	0.0182	0.0165	0.0207	0.0126	0.1705	0.0514	0.0149	0.0845	0.0365	0.0181	0.3029	0.0443	0.0320	0.0091	0.0151	0.0175	0.0196	0.0802	0.0568	0.0148	0.0411	0.0163	0.0150	0.0176	0.0180	0.0178	0.0149	0.0152	0.0168	0.0154	0.0027	0.0732	0.0345	0.0087	
0.0133	0.0151	0.0188	0.0142	0.0169	0.0116	0.1701	0.0511	0.0174	0.0908	0.0351	0.0164	0.2997	0.0435	0.0281	0.0141	0.0096	0.0124	0.0175	0.0787	0.0587	0.0157	0.0377	0.0143	0.0159	0.0119	0.0153	0.0070	0.0156	0.0168	0.0148	0.0086	0.0161	0.0674	0.0343	0.0157	0.0160	
0.0174	0.0094	0.0180	0.0162	0.0205	0.0124	0.1709	0.0513	0.0147	0.0848	0.0363	0.0179	0.3034	0.0439	0.0319	0.0093	0.0150	0.0173	0.0194	0.0802	0.0569	0.0149	0.0410	0.0161	0.0150	0.0176	0.0179	0.0178	0.0149	0.0152	0.0167	0.0154	0.0025	0.0734	0.0344	0.0086	0.0026	0.0158	
0.0176	0.0099	0.0183	0.0166	0.0208	0.0128	0.1707	0.0515	0.0150	0.0848	0.0364	0.0183	0.3031	0.0438	0.0321	0.0093	0.0152	0.0177	0.0197	0.0804	0.0572	0.0150	0.0413	0.0163	0.0152	0.0179	0.0181	0.0181	0.0152	0.0154	0.0170	0.0155	0.0027	0.0736	0.0347	0.0088	0.0025	0.0160	0.0028	
0.0156	0.0164	0.0211	0.0177	0.0180	0.0124	0.1694	0.0534	0.0183	0.0918	0.0349	0.0180	0.3007	0.0445	0.0309	0.0157	0.0114	0.0132	0.0179	0.0801	0.0612	0.0166	0.0382	0.0163	0.0168	0.0092	0.0156	0.0044	0.0167	0.0178	0.0160	0.0110	0.0179	0.0668	0.0357	0.0168	0.0176	0.0083	0.0175	0.0177	
0.0094	0.0209	0.0140	0.0122	0.0127	0.0149	0.1701	0.0536	0.0126	0.0933	0.0411	0.0054	0.2998	0.0401	0.0260	0.0179	0.0151	0.0165	0.0110	0.0734	0.0607	0.0177	0.0316	0.0119	0.0177	0.0174	0.0096	0.0188	0.0178	0.0130	0.0077	0.0154	0.0184	0.0731	0.0360	0.0198	0.0183	0.0164	0.0182	0.0183	0.0181	
0.0091	0.0191	0.0130	0.0112	0.0109	0.0145	0.1702	0.0536	0.0112	0.0920	0.0400	0.0093	0.2992	0.0386	0.0254	0.0139	0.0127	0.0149	0.0109	0.0716	0.0609	0.0123	0.0310	0.0106	0.0124	0.0154	0.0069	0.0164	0.0125	0.0116	0.0059	0.0125	0.0175	0.0705	0.0331	0.0180	0.0173	0.0155	0.0172	0.0175	0.0161	0.0096	
0.0432	0.0546	0.0483	0.0467	0.0413	0.0481	0.1730	0.0857	0.0462	0.1283	0.0733	0.0438	0.3074	0.0750	0.0602	0.0516	0.0489	0.0510	0.0449	0.1081	0.0958	0.0516	0.0657	0.0463	0.0517	0.0491	0.0427	0.0502	0.0515	0.0455	0.0422	0.0492	0.0522	0.0403	0.0703	0.0537	0.0522	0.0500	0.0523	0.0524	0.0490	0.0431	0.0433	
0.0364	0.0388	0.0431	0.0396	0.0392	0.0341	0.1807	0.0741	0.0406	0.1135	0.0460	0.0392	0.3184	0.0650	0.0534	0.0362	0.0317	0.0358	0.0406	0.1010	0.0812	0.0388	0.0611	0.0388	0.0392	0.0301	0.0379	0.0283	0.0390	0.0402	0.0381	0.0318	0.0397	0.0887	0.0582	0.0381	0.0394	0.0312	0.0394	0.0395	0.0288	0.0397	0.0381	0.0724	
0.0130	0.0159	0.0171	0.0161	0.0152	0.0117	0.1703	0.0499	0.0157	0.0895	0.0362	0.0142	0.2995	0.0413	0.0295	0.0117	0.0087	0.0091	0.0154	0.0767	0.0578	0.0122	0.0360	0.0158	0.0123	0.0102	0.0117	0.0111	0.0122	0.0159	0.0107	0.0069	0.0144	0.0654	0.0326	0.0157	0.0141	0.0089	0.0140	0.0144	0.0105	0.0140	0.0121	0.0480	0.0332	
0.0111	0.0177	0.0124	0.0108	0.0152	0.0182	0.1696	0.0546	0.0080	0.0909	0.0425	0.0124	0.3006	0.0394	0.0264	0.0190	0.0167	0.0195	0.0141	0.0736	0.0621	0.0199	0.0339	0.0099	0.0201	0.0190	0.0114	0.0193	0.0199	0.0057	0.0122	0.0167	0.0166	0.0755	0.0382	0.0170	0.0165	0.0179	0.0163	0.0166	0.0191	0.0127	0.0120	0.0444	0.0411	0.0163	
0.0755	0.0750	0.0800	0.0779	0.0760	0.0701	0.2129	0.1206	0.0779	0.1480	0.0832	0.0759	0.4011	0.0635	0.0904	0.0715	0.0684	0.0725	0.0779	0.1499	0.1256	0.0761	0.1020	0.0770	0.0760	0.0663	0.0749	0.0667	0.0762	0.0783	0.0733	0.0692	0.0742	0.1343	0.0971	0.0741	0.0744	0.0685	0.0739	0.0736	0.0662	0.0763	0.0748	0.1139	0.0641	0.0688	0.0785	
0.1020	0.1018	0.1036	0.1017	0.1030	0.1008	0.1987	0.1339	0.1018	0.1650	0.1065	0.1022	0.3586	0.0818	0.1124	0.1023	0.0991	0.0995	0.1026	0.1661	0.1472	0.1015	0.1177	0.1005	0.1017	0.0997	0.1011	0.0987	0.1016	0.1019	0.1008	0.0990	0.1029	0.1510	0.1196	0.1011	0.1030	0.0991	0.1027	0.1021	0.0987	0.1022	0.1011	0.1381	0.1039	0.0986	0.1020	0.0610	
0.0581	0.0582	0.0599	0.0578	0.0599	0.0562	0.2006	0.0949	0.0587	0.1264	0.0754	0.0591	0.3395	0.0494	0.0685	0.0591	0.0555	0.0568	0.0596	0.1229	0.1038	0.0588	0.0765	0.0565	0.0590	0.0568	0.0574	0.0553	0.0590	0.0585	0.0575	0.0553	0.0594	0.1090	0.0771	0.0584	0.0593	0.0550	0.0591	0.0588	0.0547	0.0591	0.0578	0.0919	0.0661	0.0551	0.0595	0.0586	0.0681	
0.0924	0.0905	0.0976	0.0936	0.0933	0.0848	0.2095	0.1261	0.0954	0.1694	0.1147	0.0935	0.3507	0.0760	0.1089	0.0873	0.0897	0.0909	0.0943	0.1600	0.1370	0.0882	0.1163	0.0926	0.0883	0.0898	0.0909	0.0888	0.0884	0.0936	0.0900	0.0882	0.0903	0.0834	0.1112	0.0892	0.0905	0.0876	0.0904	0.0901	0.0881	0.0931	0.0908	0.0638	0.1126	0.0880	0.0953	0.0821	0.0939	0.0926
0.0809	0.0680	0.0769	0.0758	0.0832	0.0741	0.1534	0.1099	0.0793	0.1014	0.0955	0.0815	0.2924	0.1080	0.0732	0.0736	0.0773	0.0768	0.0797	0.1407	0.1173	0.0738	0.0602	0.0754	0.0739	0.0797	0.0808	0.0791	0.0738	0.0786	0.0801	0.0779	0.0710	0.0353	0.0936	0.0679	0.0709	0.0769	0.0711	0.0711	0.0785	0.0816	0.0804	0.0596	0.1024	0.0776	0.0795	0.1482	0.1592	0.1164
0.0798	0.0688	0.0800	0.0763	0.0812	0.0745	0.1674	0.1088	0.0779	0.1479	0.0739	0.0798	0.3049	0.1068	0.0941	0.0727	0.0764	0.0796	0.0825	0.1386	0.1161	0.0765	0.1040	0.0766	0.0766	0.0765	0.0801	0.0779	0.0765	0.0783	0.0795	0.0777	0.0697	0.0775	0.0923	0.0689	0.0694	0.0777	0.0699	0.0698	0.0776	0.0795	0.0794	0.0571	0.0862	0.0770	0.0776	0.1299	0.1454	0.1175
0.0135	0.0149	0.0185	0.0153	0.0179	0.0107	0.1697	0.0511	0.0162	0.0900	0.0336	0.0154	0.2993	0.0409	0.0292	0.0131	0.0085	0.0107	0.0154	0.0782	0.0585	0.0142	0.0367	0.0142	0.0144	0.0099	0.0129	0.0087	0.0143	0.0156	0.0138	0.0082	0.0155	0.0672	0.0338	0.0140	0.0153	0.0079	0.0151	0.0155	0.0076	0.0156	0.0138	0.0489	0.0295	0.0083	0.0168	0.0670	0.0975	0.0536
0.0179	0.0053	0.0182	0.0130	0.0209	0.0118	0.1713	0.0537	0.0160	0.0861	0.0353	0.0185	0.3054	0.0443	0.0289	0.0115	0.0157	0.0177	0.0201	0.0817	0.0584	0.0153	0.0398	0.0132	0.0154	0.0184	0.0184	0.0167	0.0154	0.0161	0.0173	0.0158	0.0083	0.0728	0.0334	0.0059	0.0084	0.0134	0.0083	0.0084	0.0160	0.0187	0.0181	0.0527	0.0382	0.0139	0.0172	0.0743	0.1026	0.0585
0.0137	0.0116	0.0190	0.0182	0.0176	0.0105	0.1716	0.0491	0.0172	0.0869	0.0343	0.0163	0.3054	0.0434	0.0323	0.0063	0.0114	0.0153	0.0176	0.0772	0.0542	0.0129	0.0381	0.0178	0.0131	0.0148	0.0148	0.0143	0.0131	0.0169	0.0147	0.0117	0.0090	0.0702	0.0331	0.0112	0.0089	0.0122	0.0089	0.0090	0.0150	0.0166	0.0153	0.0496	0.0353	0.0120	0.0179	0.0708	0.1028	0.0585
0.0160	0.0172	0.0158	0.0215	0.0203	0.0139	0.1697	0.0436	0.0191	0.0870	0.0358	0.0185	0.3031	0.0453	0.0340	0.0131	0.0137	0.0117	0.0138	0.0743	0.0503	0.0088	0.0358	0.0200	0.0089	0.0179	0.0179	0.0164	0.0089	0.0188	0.0174	0.0141	0.0156	0.0683	0.0393	0.0167	0.0155	0.0159	0.0152	0.0154	0.0170	0.0185	0.0174	0.0520	0.0378	0.0140	0.0192	0.0761	0.1026	0.0601
0.0339	0.0335	0.0345	0.0313	0.0381	0.0342	0.1554	0.0737	0.0323	0.1086	0.0580	0.0351	0.2833	0.0448	0.0470	0.0364	0.0335	0.0367	0.0362	0.0979	0.0806	0.0370	0.0574	0.0309	0.0375	0.0362	0.0341	0.0349	0.0373	0.0321	0.0337	0.0341	0.0347	0.0933	0.0135	0.0331	0.0345	0.0340	0.0343	0.0346	0.0345	0.0355	0.0335	0.0700	0.0564	0.0341	0.0326	0.1002	0.1196	0.0756
0.0931	0.0937	0.0947	0.0960	0.0934	0.0909	0.1530	0.1254	0.0963	0.1235	0.1129	0.0955	0.2764	0.1240	0.0908	0.0909	0.0896	0.0902	0.0922	0.1552	0.1328	0.0905	0.0732	0.0956	0.0902	0.0941	0.0946	0.0928	0.0902	0.0959	0.0934	0.0903	0.0924	0.0506	0.1120	0.0926	0.0926	0.0908	0.0927	0.0926	0.0938	0.0952	0.0937	0.0731	0.1168	0.0906	0.0969	0.1717	0.1793	0.1349
0.0107	0.0208	0.0146	0.0121	0.0126	0.0146	0.1705	0.0550	0.0146	0.0947	0.0409	0.0125	0.2983	0.0420	0.0259	0.0179	0.0157	0.0194	0.0144	0.0755	0.0629	0.0195	0.0345	0.0123	0.0197	0.0185	0.0118	0.0172	0.0196	0.0136	0.0101	0.0154	0.0205	0.0731	0.0355	0.0199	0.0204	0.0145	0.0204	0.0205	0.0177	0.0125	0.0115	0.0449	0.0386	0.0166	0.0149	0.0762	0.1047	0.0604
0.0483	0.0498	0.0457	0.0476	0.0525	0.0499	0.1371	0.0871	0.0470	0.0818	0.0744	0.0495	0.2755	0.0572	0.0448	0.0499	0.0486	0.0480	0.0476	0.1142	0.0951	0.0483	0.0277	0.0469	0.0483	0.0503	0.0480	0.0519	0.0484	0.0469	0.0476	0.0483	0.0483	0.0645	0.0402	0.0492	0.0481	0.0507	0.0480	0.0482	0.0510	0.0495	0.0482	0.0855	0.0736	0.0482	0.0473	0.1186	0.1293	0.0878
0.0074	0.0175	0.0113	0.0088	0.0102	0.0122	0.1701	0.0526	0.0113	0.0932	0.0388	0.0097	0.3011	0.0387	0.0234	0.0150	0.0131	0.0162	0.0114	0.0731	0.0604	0.0165	0.0310	0.0090	0.0167	0.0155	0.0087	0.0138	0.0165	0.0106	0.0078	0.0121	0.0177	0.0710	0.0333	0.0167	0.0175	0.0113	0.0174	0.0176	0.0146	0.0099	0.0087	0.0440	0.0353	0.0131	0.0117	0.0727	0.1012	0.0569
0.0148	0.0240	0.0197	0.0171	0.0190	0.0200	0.1758	0.0612	0.0150	0.1012	0.0466	0.0157	0.3078	0.0435	0.0314	0.0222	0.0202	0.0225	0.0168	0.0814	0.0691	0.0233	0.0371	0.0165	0.0232	0.0226	0.0143	0.0224	0.0232	0.0153	0.0146	0.0209	0.0230	0.0802	0.0410	0.0232	0.0231	0.0213	0.0228	0.0230	0.0217	0.0158	0.0154	0.0508	0.0445	0.0189	0.0164	0.0842	0.1110	0.0655
0.1167	0.1167	0.1128	0.1122	0.1195	0.1215	0.1569	0.1598	0.1145	0.1347	0.1442	0.1182	0.3162	0.1488	0.1064	0.1234	0.1223	0.1223	0.1151	0.1514	0.1652	0.1224	0.0952	0.1123	0.1225	0.1258	0.1171	0.1243	0.1220	0.1150	0.1167	0.1235	0.1201	0.0799	0.1423	0.1170	0.1204	0.1217	0.1202	0.1204	0.1238	0.1183	0.1169	0.0973	0.1425	0.1225	0.1165	0.1733	0.1799	0.1591
0.0157	0.0188	0.0148	0.0192	0.0188	0.0143	0.1709	0.0471	0.0180	0.0895	0.0355	0.0173	0.3002	0.0440	0.0321	0.0143	0.0109	0.0048	0.0112	0.0740	0.0552	0.0087	0.0355	0.0180	0.0087	0.0120	0.0138	0.0138	0.0086	0.0184	0.0139	0.0090	0.0178	0.0645	0.0362	0.0178	0.0176	0.0124	0.0176	0.0179	0.0131	0.0169	0.0148	0.0515	0.0351	0.0090	0.0191	0.0710	0.1000	0.0567
0.0158	0.0140	0.0205	0.0170	0.0189	0.0078	0.1702	0.0484	0.0177	0.0903	0.0355	0.0153	0.3020	0.0438	0.0310	0.0133	0.0137	0.0159	0.0182	0.0785	0.0548	0.0139	0.0388	0.0171	0.0140	0.0159	0.0165	0.0153	0.0139	0.0177	0.0150	0.0142	0.0135	0.0719	0.0358	0.0135	0.0133	0.0132	0.0132	0.0136	0.0145	0.0152	0.0164	0.0491	0.0361	0.0126	0.0172	0.0730	0.1008	0.0574
0.0092	0.0196	0.0140	0.0107	0.0128	0.0137	0.1708	0.0544	0.0124	0.0937	0.0404	0.0060	0.3028	0.0399	0.0251	0.0176	0.0148	0.0166	0.0110	0.0741	0.0618	0.0179	0.0315	0.0108	0.0178	0.0173	0.0093	0.0175	0.0178	0.0126	0.0081	0.0154	0.0183	0.0735	0.0353	0.0189	0.0182	0.0152	0.0180	0.0183	0.0172	0.0041	0.0095	0.0445	0.0387	0.0138	0.0131	0.0755	0.1018	0.0583
0.0158	0.0140	0.0208	0.0174	0.0192	0.0076	0.1702	0.0485	0.0175	0.0897	0.0351	0.0155	0.3021	0.0436	0.0312	0.0132	0.0136	0.0158	0.0182	0.0785	0.0543	0.0139	0.0388	0.0171	0.0140	0.0160	0.0162	0.0152	0.0139	0.0177	0.0152	0.0141	0.0136	0.0721	0.0360	0.0132	0.0136	0.0135	0.0135	0.0139	0.0145	0.0153	0.0165	0.0492	0.0358	0.0128	0.0173	0.0728	0.1008	0.0575
0.0065	0.0188	0.0125	0.0122	0.0107	0.0154	0.1693	0.0518	0.0099	0.0927	0.0404	0.0100	0.2990	0.0387	0.0260	0.0149	0.0128	0.0159	0.0108	0.0718	0.0598	0.0169	0.0308	0.0110	0.0171	0.0164	0.0086	0.0150	0.0168	0.0104	0.0085	0.0121	0.0174	0.0706	0.0354	0.0181	0.0174	0.0139	0.0172	0.0175	0.0158	0.0101	0.0088	0.0435	0.0367	0.0138	0.0108	0.0747	0.1018	0.0581
0.0120	0.0172	0.0136	0.0104	0.0163	0.0171	0.1701	0.0561	0.0090	0.0915	0.0424	0.0126	0.3010	0.0406	0.0261	0.0204	0.0180	0.0206	0.0148	0.0755	0.0631	0.0214	0.0349	0.0100	0.0215	0.0211	0.0129	0.0198	0.0213	0.0095	0.0131	0.0188	0.0175	0.0764	0.0388	0.0171	0.0176	0.0185	0.0175	0.0178	0.0195	0.0129	0.0132	0.0463	0.0412	0.0180	0.0086	0.0796	0.1024	0.0595
0.0190	0.0112	0.0198	0.0178	0.0200	0.0133	0.1715	0.0523	0.0164	0.0857	0.0379	0.0195	0.3041	0.0453	0.0337	0.0097	0.0167	0.0177	0.0216	0.0806	0.0585	0.0152	0.0417	0.0179	0.0154	0.0189	0.0183	0.0188	0.0153	0.0168	0.0171	0.0158	0.0048	0.0723	0.0348	0.0104	0.0046	0.0175	0.0047	0.0049	0.0189	0.0200	0.0173	0.0529	0.0403	0.0146	0.0182	0.0743	0.1030	0.0596
0.0173	0.0095	0.0180	0.0161	0.0205	0.0123	0.1706	0.0516	0.0147	0.0847	0.0364	0.0179	0.3043	0.0440	0.0323	0.0094	0.0152	0.0175	0.0197	0.0804	0.0571	0.0150	0.0410	0.0163	0.0151	0.0178	0.0180	0.0180	0.0150	0.0154	0.0168	0.0155	0.0026	0.0731	0.0347	0.0088	0.0026	0.0160	0.0026	0.0027	0.0177	0.0184	0.0174	0.0523	0.0393	0.0143	0.0166	0.0741	0.1027	0.0589
0.0145	0.0252	0.0199	0.0176	0.0175	0.0210	0.1755	0.0588	0.0183	0.1000	0.0471	0.0173	0.3009	0.0460	0.0320	0.0205	0.0200	0.0223	0.0182	0.0784	0.0670	0.0229	0.0384	0.0168	0.0231	0.0232	0.0142	0.0222	0.0229	0.0176	0.0143	0.0187	0.0256	0.0778	0.0408	0.0244	0.0255	0.0205	0.0254	0.0256	0.0225	0.0174	0.0148	0.0517	0.0430	0.0197	0.0187	0.0832	0.1084	0.0651
0.0143	0.0252	0.0200	0.0177	0.0176	0.0210	0.1751	0.0587	0.0184	0.1001	0.0470	0.0172	0.3007	0.0462	0.0319	0.0204	0.0199	0.0223	0.0182	0.0781	0.0668	0.0229	0.0385	0.0169	0.0230	0.0233	0.0143	0.0222	0.0229	0.0177	0.0144	0.0186	0.0256	0.0774	0.0407	0.0245	0.0255	0.0204	0.0255	0.0257	0.0224	0.0176	0.0149	0.0518	0.0428	0.0197	0.0187	0.0835	0.1083	0.0648
0.0081	0.0208	0.0144	0.0127	0.0083	0.0144	0.1694	0.0537	0.0120	0.0946	0.0408	0.0103	0.3015	0.0404	0.0265	0.0167	0.0145	0.0176	0.0116	0.0731	0.0613	0.0184	0.0319	0.0125	0.0187	0.0143	0.0099	0.0136	0.0185	0.0123	0.0086	0.0145	0.0188	0.0697	0.0371	0.0200	0.0186	0.0145	0.0185	0.0188	0.0141	0.0107	0.0101	0.0427	0.0351	0.0144	0.0128	0.0726	0.1020	0.0587
0.0099	0.0158	0.0123	0.0101	0.0151	0.0166	0.1691	0.0545	0.0077	0.0905	0.0430	0.0120	0.3006	0.0389	0.0256	0.0186	0.0164	0.0190	0.0131	0.0738	0.0620	0.0198	0.0336	0.0093	0.0200	0.0195	0.0112	0.0191	0.0198	0.0090	0.0114	0.0170	0.0156	0.0750	0.0379	0.0162	0.0156	0.0177	0.0154	0.0158	0.0187	0.0122	0.0115	0.0465	0.0408	0.0158	0.0094	0.0784	0.1020	0.0592
0.0153	0.0108	0.0172	0.0150	0.0198	0.0116	0.1696	0.0497	0.0125	0.0847	0.0382	0.0168	0.3017	0.0414	0.0312	0.0129	0.0135	0.0159	0.0181	0.0801	0.0568	0.0144	0.0392	0.0143	0.0145	0.0166	0.0166	0.0164	0.0144	0.0138	0.0166	0.0142	0.0101	0.0721	0.0381	0.0111	0.0102	0.0150	0.0100	0.0104	0.0160	0.0172	0.0167	0.0516	0.0378	0.0132	0.0145	0.0753	0.1009	0.0573

0.0721	0.0695	0.0781	0.0739	0.0741	0.0699	0.1682	0.1038	0.0753	0.1494	0.0684	0.0738	0.3069	0.1026	0.0881	0.0686	0.0700	0.0739	0.0761	0.1333	0.1128	0.0739	0.0964	0.0735	0.0741	0.0728	0.0742	0.0695	0.0738	0.0750	0.0736	0.0708	0.0703	0.0715	0.0896	0.0695	0.0700	0.0688	0.0703	0.0702	0.0704	0.0736	0.0731	0.0522	0.0774	0.0700	0.0749	0.1224	0.1419	0.1131
0.0584	0.0546	0.0575	0.0586	0.0578	0.0523	0.1431	0.0899	0.0574	0.0823	0.0777	0.0600	0.2748	0.0828	0.0546	0.0557	0.0560	0.0551	0.0567	0.1197	0.0976	0.0533	0.0357	0.0576	0.0534	0.0559	0.0584	0.0544	0.0531	0.0573	0.0581	0.0563	0.0535	0.0655	0.0793	0.0541	0.0533	0.0572	0.0534	0.0536	0.0545	0.0596	0.0583	0.0905	0.0772	0.0562	0.0585	0.1059	0.1198	0.0908
0.1548	0.1534	0.1526	0.1509	0.1535	0.1547	0.1754	0.1914	0.1536	0.1826	0.1757	0.1551	0.3185	0.1207	0.1469	0.1588	0.1558	0.1550	0.1553	0.2205	0.2021	0.1552	0.1315	0.1510	0.1555	0.1558	0.1559	0.1528	0.1552	0.1525	0.1548	0.1561	0.1552	0.1061	0.1380	0.1539	0.1551	0.1535	0.1552	0.1548	0.1530	0.1557	0.1554	0.1278	0.1797	0.1552	0.1515	0.1508	0.1415	0.1475
0.0175	0.0114	0.0190	0.0150	0.0213	0.0112	0.1706	0.0505	0.0161	0.0873	0.0382	0.0179	0.3017	0.0439	0.0308	0.0156	0.0158	0.0184	0.0211	0.0805	0.0578	0.0164	0.0404	0.0153	0.0166	0.0187	0.0189	0.0168	0.0164	0.0158	0.0179	0.0162	0.0123	0.0741	0.0385	0.0119	0.0122	0.0151	0.0122	0.0126	0.0166	0.0180	0.0184	0.0518	0.0380	0.0151	0.0151	0.0758	0.1010	0.0579
0.0119	0.0166	0.0136	0.0101	0.0161	0.0167	0.1702	0.0557	0.0089	0.0923	0.0411	0.0129	0.3005	0.0407	0.0258	0.0204	0.0177	0.0204	0.0152	0.0749	0.0635	0.0211	0.0350	0.0090	0.0213	0.0202	0.0128	0.0182	0.0210	0.0079	0.0131	0.0182	0.0175	0.0762	0.0378	0.0161	0.0176	0.0172	0.0172	0.0176	0.0181	0.0137	0.0132	0.0460	0.0398	0.0175	0.0070	0.0786	0.1021	0.0588
0.0113	0.0155	0.0127	0.0091	0.0155	0.0157	0.1740	0.0577	0.0083	0.0943	0.0412	0.0127	0.3152	0.0404	0.0266	0.0191	0.0170	0.0202	0.0149	0.0763	0.0639	0.0202	0.0354	0.0079	0.0203	0.0195	0.0122	0.0176	0.0203	0.0070	0.0123	0.0173	0.0163	0.0785	0.0374	0.0148	0.0163	0.0169	0.0162	0.0162	0.0173	0.0132	0.0123	0.0475	0.0393	0.0169	0.0062	0.0794	0.1054	0.0600
0.0094	0.0197	0.0155	0.0136	0.0123	0.0131	0.1701	0.0541	0.0106	0.0937	0.0401	0.0101	0.3043	0.0399	0.0278	0.0173	0.0149	0.0173	0.0123	0.0731	0.0602	0.0178	0.0324	0.0132	0.0179	0.0174	0.0103	0.0175	0.0177	0.0119	0.0101	0.0153	0.0184	0.0732	0.0370	0.0191	0.0184	0.0167	0.0181	0.0185	0.0171	0.0105	0.0099	0.0426	0.0386	0.0150	0.0101	0.0750	0.1025	0.0591
0.0112	0.0153	0.0134	0.0092	0.0161	0.0158	0.1700	0.0560	0.0089	0.0910	0.0416	0.0126	0.3019	0.0401	0.0252	0.0197	0.0172	0.0199	0.0144	0.0755	0.0634	0.0208	0.0349	0.0085	0.0211	0.0206	0.0122	0.0186	0.0207	0.0093	0.0124	0.0181	0.0168	0.0764	0.0377	0.0151	0.0168	0.0172	0.0166	0.0169	0.0182	0.0134	0.0126	0.0476	0.0396	0.0171	0.0104	0.0788	0.1025	0.0593
0.0149	0.0138	0.0214	0.0176	0.0192	0.0098	0.1702	0.0477	0.0166	0.0894	0.0362	0.0166	0.3024	0.0439	0.0318	0.0126	0.0130	0.0169	0.0187	0.0775	0.0541	0.0148	0.0385	0.0166	0.0150	0.0169	0.0166	0.0144	0.0148	0.0169	0.0165	0.0137	0.0148	0.0713	0.0367	0.0132	0.0146	0.0132	0.0146	0.0148	0.0148	0.0167	0.0171	0.0499	0.0349	0.0137	0.0163	0.0730	0.1011	0.0579
0.0781	0.0850	0.0810	0.0826	0.0792	0.0803	0.1386	0.1128	0.0823	0.1163	0.0814	0.0805	0.2675	0.0940	0.0774	0.0800	0.0784	0.0775	0.0783	0.1371	0.1204	0.0783	0.0571	0.0815	0.0785	0.0806	0.0783	0.0800	0.0779	0.0822	0.0783	0.0777	0.0844	0.0916	0.0682	0.0844	0.0842	0.0795	0.0845	0.0844	0.0806	0.0810	0.0786	0.1140	0.0883	0.0786	0.0817	0.1380	0.1412	0.1159
0.0323	0.0315	0.0338	0.0318	0.0364	0.0316	0.1783	0.0713	0.0312	0.0965	0.0550	0.0337	0.2969	0.0589	0.0481	0.0320	0.0276	0.0298	0.0349	0.0980	0.0780	0.0331	0.0555	0.0314	0.0331	0.0292	0.0323	0.0292	0.0330	0.0314	0.0324	0.0269	0.0305	0.0859	0.0544	0.0311	0.0305	0.0284	0.0303	0.0306	0.0287	0.0340	0.0322	0.0681	0.0513	0.0276	0.0320	0.0932	0.1171	0.0727
0.0161	0.0103	0.0179	0.0154	0.0205	0.0108	0.1695	0.0504	0.0136	0.0860	0.0379	0.0181	0.3004	0.0426	0.0310	0.0137	0.0141	0.0171	0.0195	0.0797	0.0569	0.0146	0.0392	0.0146	0.0148	0.0170	0.0169	0.0161	0.0146	0.0136	0.0175	0.0149	0.0114	0.0726	0.0383	0.0102	0.0114	0.0150	0.0113	0.0114	0.0156	0.0187	0.0177	0.0517	0.0374	0.0141	0.0146	0.0753	0.1015	0.0576
0.0093	0.0149	0.0100	0.0075	0.0142	0.0146	0.1699	0.0553	0.0087	0.0906	0.0400	0.0117	0.2995	0.0382	0.0233	0.0175	0.0152	0.0176	0.0121	0.0747	0.0626	0.0181	0.0330	0.0060	0.0183	0.0168	0.0095	0.0161	0.0182	0.0081	0.0101	0.0147	0.0159	0.0741	0.0352	0.0137	0.0158	0.0142	0.0157	0.0160	0.0156	0.0118	0.0101	0.0453	0.0372	0.0153	0.0091	0.0760	0.1007	0.0567
0.0185	0.0031	0.0195	0.0148	0.0232	0.0133	0.1723	0.0525	0.0161	0.0863	0.0355	0.0206	0.3044	0.0445	0.0304	0.0119	0.0165	0.0189	0.0212	0.0806	0.0582	0.0162	0.0420	0.0131	0.0164	0.0192	0.0191	0.0166	0.0163	0.0164	0.0193	0.0166	0.0095	0.0752	0.0346	0.0055	0.0094	0.0148	0.0094	0.0096	0.0162	0.0208	0.0188	0.0546	0.0385	0.0156	0.0175	0.0748	0.1017	0.0585
0.0151	0.0121	0.0165	0.0155	0.0180	0.0115	0.1703	0.0495	0.0138	0.0847	0.0379	0.0172	0.3002	0.0419	0.0309	0.0109	0.0122	0.0141	0.0175	0.0787	0.0569	0.0115	0.0381	0.0142	0.0117	0.0153	0.0139	0.0156	0.0116	0.0132	0.0143	0.0116	0.0102	0.0706	0.0366	0.0114	0.0099	0.0144	0.0098	0.0101	0.0152	0.0173	0.0143	0.0504	0.0375	0.0113	0.0137	0.0746	0.1004	0.0566
0.0496	0.0448	0.0482	0.0491	0.0531	0.0460	0.1564	0.0821	0.0477	0.0704	0.0692	0.0522	0.2908	0.0773	0.0443	0.0471	0.0477	0.0467	0.0491	0.1102	0.0871	0.0445	0.0286	0.0486	0.0445	0.0506	0.0503	0.0500	0.0442	0.0487	0.0509	0.0479	0.0449	0.0623	0.0688	0.0451	0.0448	0.0488	0.0447	0.0449	0.0499	0.0522	0.0504	0.0856	0.0733	0.0473	0.0492	0.1167	0.1289	0.0872
0.0113	0.0154	0.0116	0.0084	0.0134	0.0169	0.1701	0.0563	0.0094	0.0898	0.0419	0.0113	0.3029	0.0390	0.0247	0.0153	0.0169	0.0182	0.0137	0.0746	0.0630	0.0186	0.0338	0.0077	0.0187	0.0188	0.0106	0.0190	0.0186	0.0096	0.0094	0.0162	0.0123	0.0733	0.0349	0.0143	0.0121	0.0168	0.0120	0.0123	0.0188	0.0119	0.0101	0.0459	0.0403	0.0149	0.0103	0.0765	0.1015	0.0585
0.0182	0.0102	0.0189	0.0169	0.0197	0.0129	0.1714	0.0521	0.0158	0.0854	0.0371	0.0189	0.3045	0.0448	0.0326	0.0086	0.0161	0.0168	0.0203	0.0800	0.0581	0.0144	0.0416	0.0167	0.0146	0.0179	0.0171	0.0182	0.0145	0.0161	0.0162	0.0148	0.0043	0.0722	0.0341	0.0097	0.0041	0.0164	0.0041	0.0042	0.0178	0.0193	0.0165	0.0525	0.0398	0.0133	0.0174	0.0747	0.1037	0.0594
0.3874	0.3863	0.3861	0.3845	0.3841	0.3861	0.2578	0.3682	0.3867	0.3417	0.3791	0.3872	0.1329	0.4019	0.3766	0.3871	0.3863	0.3870	0.3862	0.3394	0.3535	0.3870	0.3617	0.3858	0.3868	0.3864	0.3870	0.3851	0.3862	0.3862	0.3864	0.3870	0.3867	0.3365	0.3618	0.3866	0.3867	0.3853	0.3870	0.3867	0.3857	0.3861	0.3861	0.3588	0.3931	0.3864	0.3863	0.4450	0.4095	0.4195
0.0124	0.0226	0.0127	0.0151	0.0143	0.0179	0.1702	0.0514	0.0160	0.0939	0.0398	0.0153	0.3000	0.0444	0.0276	0.0208	0.0180	0.0158	0.0098	0.0711	0.0583	0.0159	0.0321	0.0143	0.0160	0.0216	0.0141	0.0188	0.0158	0.0153	0.0133	0.0183	0.0230	0.0720	0.0399	0.0219	0.0228	0.0178	0.0226	0.0229	0.0195	0.0152	0.0134	0.0470	0.0405	0.0191	0.0165	0.0803	0.1055	0.0614
0.0443	0.0435	0.0462	0.0432	0.0470	0.0465	0.1913	0.0857	0.0420	0.1241	0.0729	0.0462	0.3273	0.0666	0.0600	0.0458	0.0448	0.0457	0.0464	0.1121	0.0947	0.0466	0.0691	0.0425	0.0467	0.0474	0.0438	0.0472	0.0466	0.0427	0.0435	0.0440	0.0445	0.1049	0.0502	0.0450	0.0445	0.0453	0.0443	0.0447	0.0468	0.0466	0.0434	0.0828	0.0707	0.0428	0.0431	0.1183	0.1360	0.0894
0.0552	0.0538	0.0556	0.0546	0.0572	0.0527	0.1870	0.0928	0.0540	0.1069	0.0754	0.0579	0.3126	0.0829	0.0549	0.0557	0.0506	0.0518	0.0576	0.1201	0.1000	0.0553	0.0561	0.0537	0.0556	0.0507	0.0560	0.0496	0.0552	0.0538	0.0563	0.0500	0.0542	0.0848	0.0775	0.0531	0.0541	0.0505	0.0540	0.0543	0.0496	0.0582	0.0562	0.0908	0.0722	0.0505	0.0548	0.1163	0.1367	0.0922
0.0099	0.0163	0.0107	0.0091	0.0139	0.0159	0.1697	0.0546	0.0068	0.0902	0.0414	0.0112	0.3018	0.0388	0.0248	0.0176	0.0150	0.0176	0.0124	0.0733	0.0614	0.0180	0.0328	0.0090	0.0182	0.0181	0.0102	0.0181	0.0180	0.0077	0.0102	0.0158	0.0149	0.0744	0.0367	0.0161	0.0149	0.0166	0.0146	0.0150	0.0178	0.0118	0.0103	0.0458	0.0401	0.0145	0.0083	0.0779	0.1015	0.0580
0.0858	0.0899	0.0874	0.0897	0.0840	0.0831	0.1516	0.1171	0.0891	0.1200	0.0850	0.0876	0.2897	0.1170	0.0855	0.0858	0.0801	0.0813	0.0868	0.1480	0.1261	0.0837	0.0643	0.0896	0.0836	0.0811	0.0873	0.0812	0.0833	0.0896	0.0868	0.0805	0.0884	0.0269	0.1099	0.0895	0.0881	0.0819	0.0885	0.0883	0.0820	0.0874	0.0865	0.0540	0.0859	0.0817	0.0893	0.1356	0.1434	0.1207
0.0409	0.0441	0.0415	0.0436	0.0421	0.0384	0.1534	0.0754	0.0438	0.0702	0.0620	0.0414	0.2878	0.0694	0.0373	0.0396	0.0362	0.0325	0.0395	0.1027	0.0838	0.0364	0.0196	0.0430	0.0364	0.0368	0.0392	0.0381	0.0362	0.0441	0.0374	0.0338	0.0424	0.0470	0.0587	0.0437	0.0423	0.0359	0.0422	0.0424	0.0378	0.0411	0.0397	0.0759	0.0606	0.0327	0.0447	0.1002	0.1177	0.0774
0.1214	0.1240	0.1225	0.1231	0.1229	0.1196	0.1876	0.1595	0.1247	0.1390	0.1459	0.1220	0.3447	0.0893	0.1108	0.1217	0.1210	0.1222	0.1216	0.1853	0.1662	0.1220	0.0985	0.1233	0.1220	0.1234	0.1226	0.1214	0.1220	0.1244	0.1209	0.1215	0.1241	0.1353	0.1049	0.1231	0.1242	0.1201	0.1240	0.1237	0.1224	0.1226	0.1220	0.1573	0.1415	0.1218	0.1235	0.0935	0.1047	0.1143
0.0102	0.0151	0.0115	0.0072	0.0142	0.0147	0.1702	0.0555	0.0092	0.0907	0.0408	0.0113	0.3002	0.0394	0.0225	0.0182	0.0163	0.0181	0.0123	0.0752	0.0629	0.0189	0.0338	0.0075	0.0192	0.0184	0.0109	0.0174	0.0190	0.0087	0.0102	0.0161	0.0152	0.0750	0.0358	0.0153	0.0151	0.0145	0.0150	0.0152	0.0168	0.0114	0.0114	0.0460	0.0392	0.0151	0.0102	0.0775	0.1022	0.0576
0.0413	0.0424	0.0434	0.0418	0.0451	0.0414	0.1724	0.0716	0.0408	0.1084	0.0628	0.0413	0.3043	0.0683	0.0545	0.0422	0.0420	0.0434	0.0432	0.0953	0.0765	0.0437	0.0604	0.0416	0.0437	0.0442	0.0414	0.0444	0.0436	0.0404	0.0414	0.0422	0.0414	0.0951	0.0599	0.0414	0.0415	0.0426	0.0414	0.0416	0.0439	0.0412	0.0416	0.0737	0.0645	0.0417	0.0407	0.1060	0.1208	0.0819
0.0102	0.0160	0.0129	0.0089	0.0147	0.0151	0.1698	0.0559	0.0088	0.0904	0.0408	0.0120	0.3002	0.0400	0.0245	0.0188	0.0164	0.0191	0.0125	0.0760	0.0626	0.0197	0.0335	0.0078	0.0200	0.0196	0.0108	0.0179	0.0198	0.0093	0.0112	0.0168	0.0161	0.0755	0.0368	0.0157	0.0161	0.0164	0.0159	0.0161	0.0174	0.0125	0.0117	0.0461	0.0395	0.0163	0.0107	0.0784	0.1027	0.0579
0.0125	0.0144	0.0139	0.0114	0.0159	0.0186	0.1714	0.0569	0.0103	0.0905	0.0412	0.0133	0.3022	0.0413	0.0268	0.0157	0.0187	0.0200	0.0141	0.0743	0.0633	0.0213	0.0359	0.0117	0.0214	0.0209	0.0132	0.0213	0.0213	0.0111	0.0122	0.0184	0.0123	0.0769	0.0353	0.0146	0.0123	0.0186	0.0123	0.0124	0.0207	0.0134	0.0135	0.0483	0.0429	0.0174	0.0120	0.0769	0.1039	0.0608
0.0132	0.0156	0.0189	0.0156	0.0166	0.0110	0.1700	0.0510	0.0171	0.0910	0.0349	0.0154	0.2991	0.0425	0.0300	0.0125	0.0078	0.0116	0.0170	0.0779	0.0579	0.0143	0.0372	0.0148	0.0145	0.0109	0.0145	0.0092	0.0143	0.0167	0.0132	0.0077	0.0161	0.0656	0.0343	0.0144	0.0158	0.0075	0.0158	0.0161	0.0096	0.0161	0.0138	0.0482	0.0301	0.0093	0.0178	0.0674	0.0989	0.0548
0.0330	0.0435	0.0358	0.0363	0.0357	0.0385	0.1490	0.0755	0.0368	0.0769	0.0619	0.0358	0.2879	0.0649	0.0290	0.0405	0.0383	0.0389	0.0335	0.0944	0.0821	0.0397	0.0162	0.0351	0.0400	0.0382	0.0341	0.0361	0.0395	0.0365	0.0338	0.0381	0.0440	0.0544	0.0596	0.0427	0.0439	0.0384	0.0440	0.0440	0.0366	0.0361	0.0342	0.0689	0.0577	0.0398	0.0378	0.1013	0.1186	0.0779
0.0441	0.0563	0.0477	0.0491	0.0480	0.0526	0.1559	0.0866	0.0481	0.0792	0.0751	0.0474	0.2913	0.0774	0.0419	0.0534	0.0500	0.0505	0.0447	0.1042	0.0934	0.0512	0.0240	0.0480	0.0512	0.0540	0.0463	0.0522	0.0509	0.0479	0.0461	0.0505	0.0555	0.0648	0.0698	0.0566	0.0556	0.0511	0.0555	0.0556	0.0532	0.0474	0.0462	0.0806	0.0758	0.0514	0.0484	0.1208	0.1329	0.0915
0.0167	0.0089	0.0182	0.0164	0.0209	0.0130	0.1708	0.0515	0.0134	0.0842	0.0358	0.0180	0.3054	0.0432	0.0324	0.0096	0.0142	0.0169	0.0188	0.0802	0.0571	0.0149	0.0405	0.0153	0.0150	0.0173	0.0174	0.0171	0.0149	0.0143	0.0171	0.0153	0.0042	0.0739	0.0348	0.0084	0.0041	0.0161	0.0041	0.0043	0.0169	0.0187	0.0170	0.0528	0.0388	0.0145	0.0153	0.0744	0.1024	0.0590
0.0251	0.0242	0.0254	0.0281	0.0299	0.0236	0.1771	0.0551	0.0287	0.0974	0.0409	0.0277	0.3014	0.0550	0.0412	0.0202	0.0202	0.0178	0.0226	0.0797	0.0600	0.0206	0.0459	0.0272	0.0208	0.0233	0.0263	0.0217	0.0206	0.0281	0.0267	0.0204	0.0239	0.0755	0.0435	0.0230	0.0237	0.0205	0.0238	0.0238	0.0223	0.0281	0.0269	0.0621	0.0430	0.0216	0.0294	0.0814	0.1104	0.0670
0.0739	0.0704	0.0799	0.0757	0.0764	0.0719	0.1699	0.1056	0.0773	0.1509	0.0698	0.0758	0.3075	0.1048	0.0899	0.0695	0.0716	0.0756	0.0782	0.1347	0.1147	0.0758	0.0986	0.0753	0.0758	0.0747	0.0760	0.0715	0.0756	0.0767	0.0755	0.0725	0.0711	0.0723	0.0902	0.0704	0.0710	0.0704	0.0713	0.0713	0.0721	0.0756	0.0747	0.0528	0.0795	0.0719	0.0769	0.1242	0.1435	0.1147
0.0582	0.0613	0.0599	0.0616	0.0600	0.0558	0.1508	0.0906	0.0604	0.0909	0.0545	0.0584	0.2837	0.0866	0.0566	0.0583	0.0527	0.0515	0.0563	0.1177	0.0975	0.0554	0.0360	0.0606	0.0554	0.0510	0.0576	0.0522	0.0549	0.0607	0.0569	0.0528	0.0603	0.0623	0.0801	0.0606	0.0600	0.0543	0.0602	0.0602	0.0520	0.0581	0.0578	0.0897	0.0566	0.0526	0.0606	0.0995	0.1117	0.0908
0.0120	0.0130	0.0168	0.0158	0.0159	0.0082	0.1689	0.0473	0.0151	0.0865	0.0348	0.0137	0.3014	0.0404	0.0295	0.0100	0.0099	0.0122	0.0138	0.0779	0.0546	0.0105	0.0352	0.0151	0.0107	0.0117	0.0116	0.0127	0.0106	0.0143	0.0120	0.0101	0.0107	0.0688	0.0343	0.0128	0.0105	0.0106	0.0105	0.0106	0.0122	0.0135	0.0135	0.0458	0.0348	0.0084	0.0155	0.0704	0.1011	0.0559
0.0174	0.0138	0.0214	0.0200	0.0210	0.0125	0.1722	0.0507	0.0192	0.0877	0.0362	0.0167	0.3042	0.0451	0.0340	0.0097	0.0141	0.0167	0.0199	0.0795	0.0573	0.0144	0.0401	0.0200	0.0145	0.0172	0.0169	0.0183	0.0145	0.0189	0.0163	0.0147	0.0112	0.0730	0.0338	0.0126	0.0110	0.0163	0.0111	0.0111	0.0177	0.0170	0.0170	0.0529	0.0393	0.0137	0.0197	0.0740	0.1041	0.0613
0.0136	0.0136	0.0177	0.0169	0.0180	0.0087	0.1694	0.0482	0.0156	0.0870	0.0360	0.0140	0.2996	0.0405	0.0307	0.0097	0.0099	0.0126	0.0155	0.0776	0.0550	0.0102	0.0357	0.0159	0.0103	0.0123	0.0122	0.0140	0.0102	0.0150	0.0117	0.0105	0.0123	0.0694	0.0341	0.0124	0.0122	0.0130	0.0122	0.0124	0.0133	0.0138	0.0128	0.0482	0.0352	0.0099	0.0157	0.0714	0.0997	0.0566
0.0135	0.0138	0.0180	0.0172	0.0181	0.0091	0.1698	0.0483	0.0156	0.0870	0.0360	0.0144	0.3000	0.0405	0.0307	0.0096	0.0103	0.0127	0.0156	0.0777	0.0549	0.0105	0.0359	0.0161	0.0107	0.0124	0.0124	0.0142	0.0106	0.0152	0.0122	0.0104	0.0125	0.0695	0.0347	0.0126	0.0123	0.0132	0.0123	0.0125	0.0135	0.0140	0.0134	0.0482	0.0353	0.0100	0.0158	0.0719	0.1002	0.0568
0.0778	0.0858	0.0806	0.0797	0.0778	0.0822	0.1747	0.1246	0.0813	0.1051	0.0823	0.0784	0.3458	0.1117	0.0720	0.0853	0.0835	0.0839	0.0775	0.1442	0.1284	0.0844	0.0546	0.0792	0.0842	0.0840	0.0779	0.0850	0.0839	0.0811	0.0778	0.0847	0.0873	0.0986	0.1043	0.0857	0.0872	0.0837	0.0871	0.0872	0.0838	0.0786	0.0780	0.1120	0.0610	0.0846	0.0815	0.1030	0.1334	0.1060
0.0093	0.0208	0.0141	0.0121	0.0126	0.0147	0.1704	0.0541	0.0124	0.0934	0.0412	0.0052	0.3005	0.0401	0.0260	0.0179	0.0148	0.0164	0.0109	0.0737	0.0608	0.0176	0.0312	0.0119	0.0177	0.0172	0.0093	0.0186	0.0176	0.0128	0.0078	0.0152	0.0183	0.0728	0.0359	0.0197	0.0182	0.0163	0.0181	0.0183	0.0180	0.0026	0.0094	0.0435	0.0394	0.0139	0.0124	0.0761	0.1029	0.0596
0.0089	0.0207	0.0140	0.0118	0.0118	0.0145	0.1699	0.0544	0.0124	0.0927	0.0415	0.0071	0.2999	0.0397	0.0257	0.0177	0.0152	0.0164	0.0108	0.0739	0.0614	0.0176	0.0309	0.0117	0.0177	0.0162	0.0090	0.0178	0.0176	0.0130	0.0079	0.0151	0.0184	0.0722	0.0361	0.0197	0.0182	0.0160	0.0181	0.0184	0.0170	0.0047	0.0095	0.0429	0.0386	0.0136	0.0133	0.0755	0.1027	0.0587
0.0108	0.0209	0.0099	0.0146	0.0153	0.0179	0.1694	0.0502	0.0127	0.0918	0.0396	0.0131	0.3011	0.0408	0.0273	0.0192	0.0164	0.0130	0.0077	0.0695	0.0579	0.0130	0.0301	0.0135	0.0133	0.0187	0.0110	0.0185	0.0130	0.0130	0.0120	0.0169	0.0207	0.0714	0.0384	0.0207	0.0205	0.0180	0.0203	0.0206	0.0184	0.0133	0.0109	0.0461	0.0408	0.0157	0.0134	0.0782	0.1029	0.0599
0.0391	0.0408	0.0451	0.0410	0.0425	0.0396	0.1560	0.0772	0.0412	0.1182	0.0513	0.0402	0.2913	0.0692	0.0549	0.0408	0.0408	0.0439	0.0426	0.1008	0.0845	0.0437	0.0627	0.0403	0.0441	0.0438	0.0407	0.0412	0.0437	0.0412	0.0402	0.0416	0.0420	0.0709	0.0602	0.0405	0.0419	0.0404	0.0420	0.0422	0.0411	0.0402	0.0396	0.0459	0.0527	0.0409	0.0404	0.0958	0.1177	0.0825
0.0179	0.0102	0.0187	0.0169	0.0208	0.0129	0.1706	0.0517	0.0152	0.0848	0.0368	0.0183	0.3033	0.0440	0.0325	0.0097	0.0155	0.0177	0.0199	0.0808	0.0573	0.0153	0.0410	0.0166	0.0154	0.0179	0.0184	0.0182	0.0154	0.0156	0.0172	0.0158	0.0032	0.0732	0.0349	0.0090	0.0029	0.0163	0.0030	0.0029	0.0179	0.0183	0.0176	0.0519	0.0398	0.0145	0.0169	0.0740	0.1025	0.0593
0.0110	0.0185	0.0170	0.0130	0.0153	0.0142	0.1701	0.0539	0.0122	0.0940	0.0396	0.0116	0.3003	0.0414	0.0268	0.0188	0.0163	0.0194	0.0140	0.0730	0.0606	0.0190	0.0343	0.0121	0.0192	0.0199	0.0121	0.0179	0.0192	0.0124	0.0118	0.0173	0.0197	0.0752	0.0367	0.0178	0.0196	0.0166	0.0194	0.0197	0.0173	0.0115	0.0114	0.0449	0.0389	0.0165	0.0113	0.0772	0.1027	0.0600
0.0157	0.0132	0.0215	0.0176	0.0201	0.0088	0.1705	0.0487	0.0167	0.0894	0.0346	0.0162	0.3023	0.0431	0.0317	0.0127	0.0132	0.0166	0.0187	0.0783	0.0542	0.0134	0.0389	0.0166	0.0137	0.0168	0.0165	0.0148	0.0136	0.0171	0.0163	0.0142	0.0139	0.0725	0.0360	0.0125	0.0138	0.0138	0.0138	0.0140	0.0143	0.0165	0.0159	0.0500	0.0359	0.0134	0.0163	0.0732	0.1013	0.0583
0.0113	0.0156	0.0132	0.0092	0.0159	0.0163	0.1706	0.0558	0.0082	0.0913	0.0414	0.0127	0.3006	0.0395	0.0249	0.0192	0.0171	0.0197	0.0139	0.0748	0.0628	0.0204	0.0351	0.0081	0.0205	0.0201	0.0122	0.0182	0.0203	0.0083	0.0122	0.0176	0.0164	0.0759	0.0368	0.0149	0.0164	0.0167	0.0161	0.0164	0.0179	0.0130	0.0124	0.0473	0.0398	0.0166	0.0094	0.0785	0.1027	0.0590
0.0101	0.0165	0.0124	0.0102	0.0149	0.0166	0.1695	0.0548	0.0075	0.0901	0.0427	0.0120	0.3021	0.0390	0.0259	0.0181	0.0163	0.0189	0.0130	0.0742	0.0620	0.0197	0.0336	0.0090	0.0198	0.0193	0.0112	0.0191	0.0197	0.0085	0.0113	0.0169	0.0155	0.0751	0.0379	0.0160	0.0153	0.0177	0.0153	0.0155	0.0186	0.0119	0.0114	0.0464	0.0405	0.0160	0.0090	0.0784	0.1022	0.0594
0.0185	0.0029	0.0196	0.0147	0.0231	0.0136	0.1722	0.0526	0.0162	0.0861	0.0355	0.0206	0.3037	0.0447	0.0305	0.0119	0.0164	0.0190	0.0212	0.0807	0.0583	0.0164	0.0420	0.0131	0.0166	0.0194	0.0193	0.0167	0.0165	0.0166	0.0194	0.0167	0.0096	0.0752	0.0345	0.0054	0.0094	0.0148	0.0094	0.0096	0.0165	0.0208	0.0191	0.0547	0.0388	0.0158	0.0175	0.0749	0.1019	0.0582
0.0110	0.0229	0.0168	0.0157	0.0089	0.0163	0.1707	0.0560	0.0145	0.0946	0.0418	0.0129	0.2999	0.0425	0.0284	0.0186	0.0169	0.0179	0.0130	0.0752	0.0635	0.0183	0.0339	0.0145	0.0183	0.0182	0.0093	0.0189	0.0183	0.0140	0.0090	0.0161	0.0212	0.0715	0.0370	0.0225	0.0211	0.0186	0.0210	0.0212	0.0177	0.0123	0.0100	0.0372	0.0406	0.0151	0.0147	0.0766	0.1039	0.0596
0.0244	0.0270	0.0262	0.0270	0.0281	0.0238	0.1607	0.0591	0.0273	0.0781	0.0433	0.0247	0.2947	0.0529	0.0262	0.0235	0.0234	0.0217	0.0235	0.0835	0.0657	0.0229	0.0239	0.0264	0.0231	0.0252	0.0237	0.0251	0.0228	0.0271	0.0239	0.0229	0.0261	0.0580	0.0457	0.0259	0.0261	0.0238	0.0260	0.0263	0.0246	0.0246	0.0243	0.0599	0.0450	0.0231	0.0277	0.0836	0.1056	0.0631
0.0486	0.0587	0.0539	0.0507	0.0494	0.0543	0.1891	0.0949	0.0519	0.1354	0.0804	0.0494	0.3231	0.0390	0.0654	0.0560	0.0544	0.0564	0.0517	0.1150	0.1031	0.0564	0.0717	0.0493	0.0563	0.0543	0.0483	0.0544	0.0563	0.0513	0.0476	0.0538	0.0588	0.0860	0.0754	0.0575	0.0587	0.0548	0.0587	0.0583	0.0536	0.0498	0.0483	0.0593	0.0767	0.0532	0.0519	0.0698	0.0849	0.0592
0.0576	0.0688	0.0593	0.0605	0.0584	0.0637	0.1506	0.0986	0.0605	0.0930	0.0630	0.0575	0.2840	0.0885	0.0546	0.0661	0.0628	0.0621	0.0564	0.1184	0.1053	0.0627	0.0343	0.0597	0.0628	0.0645	0.0575	0.0653	0.0623	0.0610	0.0566	0.0633	0.0679	0.0746	0.0826	0.0683	0.0679	0.0637	0.0680	0.0679	0.0647	0.0576	0.0571	0.0889	0.0694	0.0635	0.0604	0.1122	0.1192	0.0978
0.0336	0.0331	0.0383	0.0364	0.0356	0.0285	0.1666	0.0664	0.0355	0.1080	0.0318	0.0336	0.2982	0.0613	0.0504	0.0295	0.0301	0.0330	0.0351	0.0963	0.0732	0.0306	0.0567	0.0351	0.0309	0.0299	0.0325	0.0305	0.0306	0.0359	0.0315	0.0311	0.0319	0.0885	0.0541	0.0324	0.0315	0.0325	0.0318	0.0317	0.0297	0.0334	0.0328	0.0658	0.0316	0.0304	0.0356	0.0713	0.0946	0.0717
0.0484	0.0523	0.0483	0.0486	0.0500	0.0472	0.1577	0.0838	0.0515	0.1101	0.0711	0.0487	0.2839	0.0618	0.0556	0.0499	0.0487	0.0452	0.0469	0.1092	0.0928	0.0457	0.0559	0.0486	0.0457	0.0507	0.0471	0.0497	0.0456	0.0507	0.0462	0.0471	0.0523	0.0907	0.0355	0.0514	0.0522	0.0472	0.0522	0.0525	0.0489	0.0487	0.0475	0.0840	0.0727	0.0459	0.0520	0.1189	0.1274	0.0868
0.0351	0.0300	0.0365	0.0342	0.0372	0.0321	0.1877	0.0707	0.0327	0.1061	0.0574	0.0364	0.3196	0.0607	0.0497	0.0297	0.0336	0.0343	0.0374	0.0991	0.0780	0.0332	0.0588	0.0333	0.0332	0.0356	0.0340	0.0359	0.0331	0.0330	0.0336	0.0325	0.0272	0.0915	0.0500	0.0299	0.0269	0.0338	0.0268	0.0272	0.0351	0.0368	0.0340	0.0710	0.0578	0.0315	0.0340	0.0970	0.1214	0.0772
0.0337	0.0351	0.0394	0.0360	0.0344	0.0300	0.1657	0.0694	0.0364	0.1117	0.0298	0.0350	0.2970	0.0624	0.0494	0.0341	0.0293	0.0314	0.0357	0.0973	0.0772	0.0349	0.0573	0.0348	0.0350	0.0276	0.0337	0.0260	0.0348	0.0357	0.0331	0.0293	0.0356	0.0857	0.0540	0.0350	0.0353	0.0282	0.0353	0.0354	0.0252	0.0346	0.0343	0.0635	0.0298	0.0288	0.0360	0.0674	0.0928	0.0683
0.0100	0.0167	0.0102	0.0078	0.0125	0.0159	0.1704	0.0561	0.0092	0.0911	0.0421	0.0106	0.2996	0.0381	0.0242	0.0182	0.0160	0.0178	0.0125	0.0756	0.0625	0.0183	0.0329	0.0083	0.0185	0.0176	0.0100	0.0181	0.0184	0.0096	0.0085	0.0153	0.0155	0.0731	0.0362	0.0165	0.0154	0.0160	0.0153	0.0155	0.0179	0.0106	0.0100	0.0450	0.0399	0.0146	0.0099	0.0765	0.1016	0.0577
0.0405	0.0435	0.0414	0.0431	0.0414	0.0379	0.1533	0.0748	0.0430	0.0702	0.0615	0.0409	0.2863	0.0686	0.0370	0.0389	0.0359	0.0324	0.0389	0.1015	0.0832	0.0362	0.0195	0.0427	0.0363	0.0361	0.0387	0.0379	0.0361	0.0432	0.0365	0.0334	0.0418	0.0465	0.0582	0.0430	0.0416	0.0357	0.0417	0.0419	0.0374	0.0405	0.0391	0.0749	0.0597	0.0325	0.0440	0.0998	0.1178	0.0764
0.1063	0.1084	0.1086	0.1048	0.1079	0.1062	0.2009	0.1181	0.1063	0.1632	0.1293	0.1069	0.2561	0.1213	0.1192	0.1082	0.1067	0.1090	0.1098	0.1682	0.1517	0.1096	0.1310	0.1057	0.1097	0.1091	0.1064	0.1081	0.1095	0.1060	0.1056	0.1058	0.1073	0.1688	0.0915	0.1083	0.1074	0.1048	0.1073	0.1074	0.1081	0.1069	0.1061	0.1429	0.1291	0.1046	0.1068	0.1781	0.1741	0.1479
0.0403	0.0407	0.0441	0.0441	0.0398	0.0350	0.1547	0.0728	0.0452	0.0698	0.0603	0.0435	0.2887	0.0712	0.0379	0.0382	0.0385	0.0391	0.0417	0.1023	0.0799	0.0367	0.0191	0.0433	0.0368	0.0390	0.0421	0.0368	0.0364	0.0447	0.0416	0.0383	0.0418	0.0474	0.0618	0.0401	0.0415	0.0388	0.0417	0.0417	0.0375	0.0438	0.0422	0.0726	0.0585	0.0402	0.0459	0.1010	0.1192	0.0780
0.1392	0.1390	0.1418	0.1380	0.1413	0.1391	0.2207	0.1498	0.1390	0.1803	0.1598	0.1399	0.2943	0.1547	0.1511	0.1394	0.1397	0.1423	0.1426	0.2005	0.1828	0.1426	0.1651	0.1385	0.1428	0.1426	0.1395	0.1409	0.1429	0.1396	0.1390	0.1391	0.1387	0.2037	0.1223	0.1391	0.1388	0.1376	0.1386	0.1387	0.1409	0.1402	0.1394	0.1775	0.1585	0.1377	0.1398	0.1827	0.1942	0.1764
0.0748	0.0830	0.0793	0.0762	0.0763	0.0797	0.1804	0.1169	0.0771	0.1617	0.0807	0.0745	0.3158	0.1055	0.0915	0.0809	0.0795	0.0823	0.0770	0.1386	0.1256	0.0833	0.0981	0.0759	0.0833	0.0797	0.0741	0.0817	0.0832	0.0773	0.0736	0.0807	0.0823	0.0806	0.1002	0.0831	0.0825	0.0811	0.0823	0.0827	0.0810	0.0743	0.0740	0.0512	0.0853	0.0796	0.0769	0.1304	0.1477	0.1220

BL13	BL14	HL47	CL66	CL67	FC26	CL68	CL69	CL70	CL71	CL72	CL73	CL74	CL75	CL76	FC27	FC3	FC28	FC29	FC30	FC31	FC32	FC33	FC34	FC35	DS3	DS4	FC4	DS5	DS6	DS7	DS8	DS9	DS10	DS11	DS12	HL1	HL2	FC5	HL3	HL4	HL5	HL6	HL7	HL8	BL1	HL9	CL1	BL2	FC6

0.0982																										
0.0986	0.0547																									
0.0864	0.0766	0.0778																								
0.0900	0.0668	0.0685	0.0139																							
0.0887	0.0734	0.0723	0.0127	0.0107																						
0.0905	0.0756	0.0780	0.0147	0.0164	0.0126																					
0.1113	0.0914	0.0906	0.0317	0.0334	0.0363	0.0380																				
0.1190	0.0432	0.0894	0.0905	0.0932	0.0906	0.0921	0.1103																			
0.0923	0.0790	0.0817	0.0152	0.0185	0.0163	0.0201	0.0337	0.0909																		
0.1311	0.0707	0.1137	0.0478	0.0487	0.0504	0.0485	0.0348	0.0931	0.0507																	
0.0897	0.0785	0.0796	0.0118	0.0155	0.0135	0.0168	0.0321	0.0921	0.0064	0.0483																
0.1010	0.0880	0.0878	0.0192	0.0228	0.0218	0.0229	0.0403	0.0991	0.0178	0.0560	0.0146															
0.1525	0.0654	0.1199	0.1217	0.1205	0.1219	0.1268	0.1387	0.0911	0.1143	0.1211	0.1164	0.1315														
0.0910	0.0768	0.0798	0.0100	0.0177	0.0153	0.0106	0.0365	0.0913	0.0189	0.0473	0.0157	0.0220	0.1237													
0.0876	0.0759	0.0750	0.0119	0.0128	0.0126	0.0128	0.0355	0.0930	0.0170	0.0514	0.0143	0.0207	0.1234	0.0160												
0.0936	0.0815	0.0804	0.0145	0.0176	0.0161	0.0191	0.0351	0.0964	0.0119	0.0504	0.0089	0.0157	0.1173	0.0169	0.0143											
0.0876	0.0760	0.0751	0.0119	0.0127	0.0125	0.0127	0.0354	0.0927	0.0175	0.0514	0.0147	0.0205	0.1232	0.0160	0.0030	0.0145										
0.0920	0.0803	0.0800	0.0134	0.0178	0.0138	0.0158	0.0333	0.0924	0.0098	0.0482	0.0068	0.0147	0.1173	0.0154	0.0160	0.0100	0.0159									
0.0959	0.0794	0.0786	0.0172	0.0166	0.0195	0.0211	0.0334	0.0969	0.0154	0.0487	0.0122	0.0172	0.1153	0.0206	0.0161	0.0126	0.0157	0.0120								
0.0892	0.0723	0.0709	0.0169	0.0097	0.0102	0.0168	0.0360	0.0940	0.0221	0.0493	0.0190	0.0243	0.1214	0.0180	0.0147	0.0194	0.0150	0.0184	0.0189							
0.0907	0.0712	0.0696	0.0154	0.0083	0.0085	0.0154	0.0345	0.0928	0.0206	0.0482	0.0176	0.0228	0.1201	0.0179	0.0131	0.0178	0.0133	0.0172	0.0173	0.0043						
0.0973	0.0872	0.0875	0.0199	0.0243	0.0212	0.0239	0.0402	0.0939	0.0157	0.0555	0.0129	0.0128	0.1240	0.0217	0.0221	0.0163	0.0220	0.0138	0.0192	0.0256	0.0253					
0.0974	0.0871	0.0877	0.0200	0.0244	0.0210	0.0239	0.0400	0.0938	0.0159	0.0556	0.0129	0.0128	0.1237	0.0217	0.0222	0.0164	0.0222	0.0139	0.0192	0.0254	0.0253	0.0020				
0.0921	0.0823	0.0797	0.0153	0.0193	0.0145	0.0183	0.0361	0.0946	0.0112	0.0505	0.0082	0.0167	0.1182	0.0173	0.0164	0.0105	0.0166	0.0081	0.0136	0.0195	0.0184	0.0161	0.0162			
0.0951	0.0795	0.0783	0.0167	0.0162	0.0175	0.0200	0.0326	0.0968	0.0147	0.0469	0.0116	0.0161	0.1141	0.0189	0.0176	0.0118	0.0171	0.0106	0.0084	0.0171	0.0153	0.0180	0.0181	0.0123		
0.0894	0.0742	0.0724	0.0139	0.0108	0.0122	0.0139	0.0326	0.0940	0.0198	0.0468	0.0166	0.0208	0.1206	0.0159	0.0118	0.0170	0.0117	0.0156	0.0135	0.0116	0.0098	0.0233	0.0234	0.0173	0.0115	

0.0946	0.0679	0.0291	0.0693	0.0688	0.0682	0.0722	0.0904	0.0809	0.0735	0.1112	0.0707	0.0798	0.1184	0.0738	0.0696	0.0735	0.0694	0.0721	0.0747	0.0711	0.0699	0.0782	0.0781	0.0727	0.0750	0.0721																							
0.1175	0.0722	0.1161	0.0549	0.0545	0.0557	0.0531	0.0757	0.0923	0.0598	0.0466	0.0582	0.0649	0.1190	0.0549	0.0549	0.0591	0.0544	0.0578	0.0582	0.0546	0.0533	0.0658	0.0657	0.0580	0.0571	0.0517	0.1136																						
0.0864	0.0979	0.1421	0.1537	0.1518	0.1580	0.1545	0.1333	0.1206	0.1548	0.1106	0.1531	0.1606	0.1477	0.1549	0.1536	0.1568	0.1537	0.1541	0.1513	0.1553	0.1553	0.1625	0.1628	0.1543	0.1527	0.1536	0.1384	0.1324																					
0.0895	0.0745	0.0741	0.0141	0.0111	0.0148	0.0160	0.0334	0.0950	0.0194	0.0491	0.0165	0.0229	0.1204	0.0187	0.0072	0.0171	0.0073	0.0179	0.0140	0.0136	0.0120	0.0238	0.0240	0.0188	0.0143	0.0097	0.0719	0.0536	0.1508																				
0.0948	0.0794	0.0790	0.0160	0.0163	0.0187	0.0204	0.0323	0.0968	0.0143	0.0487	0.0109	0.0168	0.1147	0.0202	0.0154	0.0127	0.0155	0.0117	0.0079	0.0187	0.0171	0.0186	0.0187	0.0131	0.0098	0.0149	0.0740	0.0587	0.1512	0.0134																			
0.1000	0.0822	0.0808	0.0151	0.0154	0.0180	0.0201	0.0321	0.1049	0.0134	0.0505	0.0102	0.0171	0.1193	0.0199	0.0149	0.0123	0.0148	0.0112	0.0073	0.0175	0.0159	0.0185	0.0186	0.0127	0.0094	0.0141	0.0758	0.0607	0.1567	0.0126	0.0016																		
0.0937	0.0828	0.0806	0.0151	0.0191	0.0164	0.0183	0.0360	0.0967	0.0136	0.0503	0.0107	0.0155	0.1182	0.0174	0.0132	0.0108	0.0130	0.0092	0.0101	0.0192	0.0181	0.0175	0.0176	0.0097	0.0109	0.0156	0.0734	0.0587	0.1550	0.0154	0.0105	0.0105																	
0.0946	0.0789	0.0794	0.0155	0.0154	0.0189	0.0207	0.0327	0.0974	0.0142	0.0479	0.0111	0.0160	0.1138	0.0200	0.0161	0.0121	0.0154	0.0117	0.0081	0.0182	0.0165	0.0180	0.0181	0.0133	0.0071	0.0130	0.0745	0.0572	0.1532	0.0132	0.0087	0.0081	0.0116																
0.0880	0.0762	0.0759	0.0126	0.0132	0.0120	0.0121	0.0351	0.0919	0.0169	0.0509	0.0140	0.0203	0.1243	0.0165	0.0066	0.0161	0.0060	0.0147	0.0146	0.0160	0.0146	0.0212	0.0211	0.0161	0.0164	0.0111	0.0688	0.0544	0.1533	0.0091	0.0149	0.0144	0.0132	0.0151															
0.1607	0.1025	0.1099	0.0792	0.0836	0.0810	0.0784	0.0674	0.1086	0.0802	0.0423	0.0781	0.0777	0.1473	0.0775	0.0807	0.0813	0.0803	0.0783	0.0824	0.0840	0.0844	0.0716	0.0717	0.0785	0.0822	0.0820	0.0974	0.0787	0.1411	0.0827	0.0820	0.0844	0.0806	0.0828	0.0794														
0.1101	0.0936	0.0930	0.0255	0.0314	0.0314	0.0345	0.0488	0.1131	0.0356	0.0650	0.0326	0.0414	0.1401	0.0290	0.0333	0.0340	0.0333	0.0314	0.0336	0.0316	0.0305	0.0399	0.0400	0.0335	0.0316	0.0289	0.0907	0.0723	0.1715	0.0310	0.0331	0.0343	0.0341	0.0327	0.0330	0.0999													
0.0891	0.0730	0.0728	0.0133	0.0103	0.0127	0.0143	0.0326	0.0936	0.0196	0.0475	0.0162	0.0219	0.1199	0.0171	0.0117	0.0179	0.0112	0.0161	0.0139	0.0127	0.0111	0.0238	0.0240	0.0178	0.0121	0.0081	0.0716	0.0523	0.1524	0.0087	0.0140	0.0128	0.0158	0.0126	0.0113	0.0825	0.0294												
0.0920	0.0762	0.0775	0.0128	0.0139	0.0165	0.0194	0.0296	0.0951	0.0099	0.0457	0.0065	0.0159	0.1129	0.0168	0.0165	0.0108	0.0165	0.0088	0.0097	0.0174	0.0158	0.0149	0.0150	0.0113	0.0088	0.0140	0.0734	0.0562	0.1514	0.0146	0.0082	0.0071	0.0116	0.0081	0.0163	0.0801	0.0297	0.0139											
0.0905	0.0682	0.0692	0.0145	0.0054	0.0114	0.0170	0.0334	0.0942	0.0204	0.0497	0.0172	0.0238	0.1177	0.0185	0.0139	0.0194	0.0137	0.0186	0.0172	0.0112	0.0096	0.0249	0.0251	0.0206	0.0157	0.0105	0.0697	0.0544	0.1536	0.0113	0.0166	0.0151	0.0196	0.0153	0.0135	0.0846	0.0313	0.0102	0.0144										
0.0878	0.0733	0.0726	0.0131	0.0114	0.0108	0.0137	0.0326	0.0924	0.0184	0.0467	0.0157	0.0218	0.1211	0.0143	0.0125	0.0172	0.0123	0.0151	0.0158	0.0103	0.0099	0.0216	0.0216	0.0164	0.0144	0.0090	0.0725	0.0522	0.1535	0.0108	0.0149	0.0141	0.0162	0.0147	0.0127	0.0795	0.0283	0.0090	0.0129	0.0116									
0.1271	0.0581	0.1027	0.0480	0.0440	0.0470	0.0454	0.0618	0.0806	0.0532	0.0388	0.0505	0.0571	0.1086	0.0467	0.0477	0.0522	0.0471	0.0503	0.0493	0.0460	0.0447	0.0577	0.0577	0.0522	0.0475	0.0429	0.1024	0.0413	0.1247	0.0455	0.0502	0.0513	0.0515	0.0494	0.0472	0.0711	0.0622	0.0429	0.0479	0.0446	0.0428								
0.0937	0.0767	0.0752	0.0168	0.0137	0.0150	0.0207	0.0331	0.0962	0.0140	0.0471	0.0107	0.0176	0.1142	0.0181	0.0186	0.0113	0.0189	0.0114	0.0115	0.0125	0.0121	0.0179	0.0179	0.0117	0.0094	0.0149	0.0730	0.0580	0.1538	0.0169	0.0113	0.0105	0.0126	0.0106	0.0189	0.0817	0.0318	0.0162	0.0088	0.0152	0.0140	0.0494							
0.0901	0.0719	0.0701	0.0158	0.0090	0.0095	0.0165	0.0354	0.0940	0.0213	0.0490	0.0182	0.0241	0.1212	0.0170	0.0140	0.0188	0.0143	0.0179	0.0183	0.0038	0.0038	0.0245	0.0246	0.0186	0.0163	0.0108	0.0704	0.0542	0.1568	0.0130	0.0181	0.0171	0.0184	0.0172	0.0155	0.0832	0.0311	0.0121	0.0161	0.0100	0.0093	0.0453	0.0112						
0.4056	0.3212	0.3233	0.3875	0.3921	0.3891	0.3931	0.3614	0.3075	0.3846	0.3565	0.3889	0.4010	0.2808	0.3892	0.3883	0.3883	0.3882	0.3866	0.3871	0.3867	0.3873	0.3883	0.3873	0.3860	0.3866	0.3878	0.3298	0.3485	0.3347	0.3871	0.3863	0.4016	0.3891	0.3877	0.3887	0.3218	0.3850	0.3869	0.3874	0.3879	0.3868	0.3526	0.3878	0.3879					
0.0963	0.0782	0.0825	0.0177	0.0213	0.0194	0.0155	0.0369	0.0896	0.0127	0.0507	0.0107	0.0196	0.1156	0.0151	0.0196	0.0145	0.0193	0.0127	0.0164	0.0239	0.0230	0.0190	0.0189	0.0133	0.0165	0.0216	0.0741	0.0585	0.1529	0.0215	0.0159	0.0157	0.0152	0.0154	0.0184	0.0788	0.0381	0.0215	0.0135	0.0222	0.0200	0.0508	0.0162	0.0233	0.3861				
0.1272	0.1117	0.1099	0.0440	0.0453	0.0470	0.0499	0.0463	0.1307	0.0486	0.0668	0.0451	0.0526	0.1588	0.0459	0.0471	0.0469	0.0471	0.0440	0.0440	0.0447	0.0442	0.0508	0.0514	0.0462	0.0422	0.0419	0.1076	0.0893	0.1720	0.0446	0.0440	0.0457	0.0466	0.0442	0.0472	0.0977	0.0606	0.0431	0.0420	0.0438	0.0414	0.0783	0.0427	0.0434	0.4215	0.0508			
0.1329	0.0943	0.1158	0.0489	0.0534	0.0544	0.0563	0.0717	0.1052	0.0578	0.0668	0.0542	0.0535	0.1408	0.0517	0.0552	0.0575	0.0545	0.0549	0.0554	0.0552	0.0540	0.0501	0.0504	0.0552	0.0545	0.0520	0.1115	0.0697	0.1682	0.0533	0.0546	0.0555	0.0567	0.0543	0.0541	0.0857	0.0658	0.0516	0.0522	0.0534	0.0511	0.0619	0.0551	0.0546	0.3932	0.0573	0.0858		
0.0951	0.0789	0.0776	0.0158	0.0152	0.0163	0.0186	0.0320	0.0955	0.0139	0.0465	0.0106	0.0155	0.1144	0.0177	0.0173	0.0114	0.0167	0.0100	0.0090	0.0162	0.0146	0.0175	0.0176	0.0115	0.0076	0.0130	0.0746	0.0573	0.1532	0.0153	0.0089	0.0082	0.0105	0.0087	0.0169	0.0816	0.0311	0.0134	0.0083	0.0163	0.0130	0.0475	0.0088	0.0155	0.3878	0.0156	0.0424	0.0534	
0.1049	0.0535	0.0673	0.0812	0.0886	0.0849	0.0837	0.1096	0.0689	0.0879	0.0815	0.0865	0.0972	0.1012	0.0810	0.0858	0.0885	0.0851	0.0867	0.0897	0.0889	0.0884	0.0943	0.0944	0.0851	0.0894	0.0862	0.0620	0.0787	0.1254	0.0876	0.0908	0.0950	0.0868	0.0898	0.0845	0.0792	0.1013	0.0863	0.0886	0.0899	0.0855	0.0768	0.0890	0.0885	0.3250	0.0867	0.1239	0.0981	0.0888
0.1176	0.0598	0.1056	0.0356	0.0423	0.0401	0.0390	0.0615	0.0744	0.0429	0.0329	0.0403	0.0484	0.1041	0.0314	0.0401	0.0412	0.0405	0.0413	0.0456	0.0424	0.0424	0.0475	0.0476	0.0418	0.0441	0.0413	0.0985	0.0381	0.1285	0.0429	0.0455	0.0469	0.0429	0.0453	0.0423	0.0621	0.0543	0.0422	0.0426	0.0438	0.0391	0.0241	0.0424	0.0412	0.3601	0.0437	0.0727	0.0563	0.0428
0.1140	0.1448	0.1915	0.1196	0.1237	0.1208	0.1224	0.1074	0.1606	0.1218	0.0850	0.1183	0.1324	0.1710	0.1226	0.1207	0.1227	0.1205	0.1216	0.1230	0.1245	0.1243	0.1278	0.1276	0.1215	0.1243	0.1247	0.1822	0.1054	0.0921	0.1218	0.1223	0.1263	0.1209	0.1239	0.1202	0.1107	0.1442	0.1246	0.1221	0.1238	0.1243	0.1147	0.1230	0.1253	0.3884	0.1214	0.1451	0.1423	0.1247
0.0927	0.0765	0.0774	0.0146	0.0131	0.0169	0.0201	0.0311	0.0956	0.0115	0.0470	0.0084	0.0162	0.1135	0.0183	0.0154	0.0103	0.0157	0.0110	0.0095	0.0169	0.0150	0.0167	0.0169	0.0116	0.0089	0.0134	0.0734	0.0576	0.1511	0.0134	0.0092	0.0083	0.0118	0.0084	0.0165	0.0813	0.0311	0.0139	0.0067	0.0147	0.0139	0.0482	0.0091	0.0157	0.3871	0.0142	0.0424	0.0537	0.0079
0.1180	0.0984	0.0982	0.0416	0.0430	0.0419	0.0444	0.0591	0.1119	0.0439	0.0727	0.0417	0.0466	0.1393	0.0438	0.0411	0.0414	0.0409	0.0418	0.0409	0.0430	0.0415	0.0457	0.0457	0.0429	0.0408	0.0416	0.0932	0.0792	0.1715	0.0412	0.0410	0.0432	0.0413	0.0413	0.0413	0.0977	0.0542	0.0414	0.0411	0.0422	0.0419	0.0721	0.0411	0.0424	0.3835	0.0456	0.0722	0.0775	0.0408
0.0940	0.0776	0.0787	0.0150	0.0145	0.0178	0.0199	0.0319	0.0954	0.0131	0.0473	0.0099	0.0159	0.1137	0.0186	0.0164	0.0114	0.0156	0.0113	0.0089	0.0175	0.0160	0.0173	0.0173	0.0125	0.0086	0.0140	0.0743	0.0574	0.1523	0.0147	0.0095	0.0087	0.0118	0.0073	0.0151	0.0818	0.0316	0.0139	0.0075	0.0155	0.0141	0.0484	0.0102	0.0164	0.3870	0.0137	0.0438	0.0539	0.0085
0.0965	0.0767	0.0757	0.0187	0.0132	0.0146	0.0220	0.0350	0.0965	0.0157	0.0489	0.0126	0.0184	0.1147	0.0200	0.0193	0.0133	0.0196	0.0125	0.0122	0.0141	0.0123	0.0204	0.0205	0.0134	0.0096	0.0152	0.0741	0.0596	0.1548	0.0164	0.0128	0.0118	0.0129	0.0108	0.0199	0.0845	0.0330	0.0158	0.0110	0.0141	0.0164	0.0504	0.0087	0.0128	0.3864	0.0182	0.0445	0.0575	0.0098
0.0877	0.0762	0.0770	0.0076	0.0142	0.0121	0.0150	0.0328	0.0886	0.0126	0.0492	0.0113	0.0207	0.1206	0.0115	0.0127	0.0151	0.0125	0.0135	0.0181	0.0174	0.0159	0.0189	0.0189	0.0150	0.0173	0.0144	0.0688	0.0551	0.1539	0.0147	0.0170	0.0163	0.0159	0.0166	0.0117	0.0781	0.0277	0.0144	0.0138	0.0151	0.0134	0.0484	0.0170	0.0164	0.3860	0.0164	0.0455	0.0493	0.0162
0.1168	0.0622	0.1040	0.0372	0.0425	0.0400	0.0386	0.0569	0.0763	0.0345	0.0353	0.0316	0.0411	0.0978	0.0383	0.0408	0.0354	0.0405	0.0333	0.0373	0.0451	0.0439	0.0394	0.0394	0.0315	0.0372	0.0421	0.0951	0.0401	0.1344	0.0425	0.0370	0.0378	0.0361	0.0367	0.0397	0.0605	0.0579	0.0418	0.0337	0.0431	0.0412	0.0334	0.0370	0.0443	0.3643	0.0322	0.0727	0.0586	0.0366
0.1324	0.0691	0.1144	0.0520	0.0559	0.0522	0.0512	0.0665	0.0816	0.0462	0.0443	0.0458	0.0528	0.1074	0.0510	0.0545	0.0478	0.0543	0.0452	0.0495	0.0570	0.0555	0.0526	0.0526	0.0468	0.0478	0.0534	0.1044	0.0500	0.1305	0.0556	0.0498	0.0514	0.0479	0.0496	0.0534	0.0697	0.0703	0.0546	0.0478	0.0558	0.0522	0.0189	0.0493	0.0562	0.3493	0.0454	0.0840	0.0703	0.0472
0.0919	0.0723	0.0705	0.0147	0.0090	0.0089	0.0147	0.0334	0.0942	0.0208	0.0478	0.0177	0.0225	0.1204	0.0172	0.0137	0.0182	0.0134	0.0163	0.0165	0.0060	0.0038	0.0246	0.0245	0.0185	0.0143	0.0093	0.0707	0.0528	0.1566	0.0119	0.0158	0.0150	0.0174	0.0156	0.0138	0.0839	0.0299	0.0103	0.0150	0.0089	0.0091	0.0446	0.0128	0.0056	0.3888	0.0224	0.0439	0.0535	0.0141
0.1014	0.0818	0.0860	0.0194	0.0233	0.0203	0.0212	0.0459	0.0911	0.0277	0.0582	0.0246	0.0236	0.1296	0.0176	0.0253	0.0273	0.0251	0.0262	0.0295	0.0254	0.0240	0.0199	0.0197	0.0270	0.0287	0.0258	0.0785	0.0649	0.1651	0.0272	0.0288	0.0288	0.0280	0.0283	0.0242	0.0765	0.0401	0.0261	0.0266	0.0238	0.0260	0.0567	0.0268	0.0243	0.3869	0.0240	0.0582	0.0495	0.0277
0.0954	0.0667	0.0275	0.0711	0.0693	0.0693	0.0737	0.0922	0.0816	0.0750	0.1126	0.0727	0.0813	0.1179	0.0755	0.0715	0.0752	0.0714	0.0741	0.0768	0.0720	0.0711	0.0801	0.0801	0.0744	0.0769	0.0739	0.0054	0.1158	0.1384	0.0738	0.0761	0.0775	0.0754	0.0765	0.0708	0.0987	0.0927	0.0736	0.0752	0.0703	0.0744	0.1040	0.0747	0.0713	0.3282	0.0759	0.1094	0.1134	0.0766
0.1348	0.0778	0.0900	0.0501	0.0595	0.0579	0.0556	0.0784	0.0941	0.0608	0.0506	0.0584	0.0656	0.1254	0.0503	0.0568	0.0585	0.0563	0.0582	0.0617	0.0611	0.0601	0.0661	0.0662	0.0569	0.0613	0.0583	0.0864	0.0518	0.1520	0.0591	0.0618	0.0631	0.0583	0.0617	0.0576	0.0504	0.0706	0.0588	0.0595	0.0608	0.0573	0.0477	0.0606	0.0602	0.3497	0.0596	0.0909	0.0704	0.0604
0.0854	0.0742	0.0733	0.0095	0.0113	0.0065	0.0109	0.0340	0.0907	0.0154	0.0481	0.0121	0.0187	0.1229	0.0119	0.0094	0.0133	0.0093	0.0125	0.0172	0.0121	0.0106	0.0201	0.0201	0.0132	0.0153	0.0101	0.0698	0.0530	0.1545	0.0122	0.0164	0.0157	0.0141	0.0163	0.0107	0.0792	0.0291	0.0103	0.0138	0.0125	0.0079	0.0443	0.0149	0.0110	0.3896	0.0175	0.0446	0.0529	0.0141
0.0909	0.0750	0.0735	0.0147	0.0128	0.0095	0.0159	0.0380	0.0931	0.0202	0.0522	0.0173	0.0224	0.1246	0.0170	0.0132	0.0167	0.0133	0.0179	0.0202	0.0124	0.0110	0.0244	0.0244	0.0185	0.0189	0.0134	0.0699	0.0569	0.1594	0.0155	0.0206	0.0199	0.0175	0.0198	0.0142	0.0836	0.0344	0.0146	0.0194	0.0133	0.0140	0.0485	0.0169	0.0115	0.3878	0.0231	0.0483	0.0576	0.0182
0.0860	0.0749	0.0739	0.0108	0.0127	0.0097	0.0114	0.0331	0.0910	0.0162	0.0476	0.0136	0.0185	0.1229	0.0124	0.0096	0.0138	0.0099	0.0138	0.0172	0.0136	0.0121	0.0203	0.0204	0.0154	0.0157	0.0103	0.0703	0.0527	0.1546	0.0126	0.0170	0.0162	0.0139	0.0170	0.0103	0.0795	0.0299	0.0110	0.0150	0.0131	0.0100	0.0447	0.0163	0.0130	0.3875	0.0191	0.0447	0.0537	0.0148
0.0865	0.0748	0.0738	0.0109	0.0130	0.0100	0.0115	0.0335	0.0914	0.0166	0.0475	0.0138	0.0188	0.1232	0.0125	0.0095	0.0142	0.0098	0.0138	0.0173	0.0139	0.0124	0.0203	0.0205	0.0155	0.0160	0.0103	0.0701	0.0525	0.1551	0.0126	0.0173	0.0164	0.0141	0.0172	0.0105	0.0796	0.0301	0.0111	0.0150	0.0131	0.0103	0.0445	0.0165	0.0129	0.3877	0.0195	0.0445	0.0535	0.0150
0.1751	0.1075	0.1251	0.0812	0.0863	0.0847	0.0860	0.1065	0.1250	0.0797	0.0795	0.0774	0.0896	0.1176	0.0835	0.0836	0.0782	0.0830	0.0795	0.0806	0.0884	0.0872	0.0859	0.0858	0.0799	0.0814	0.0867	0.1163	0.0812	0.1904	0.0860	0.0806	0.0844	0.0790	0.0795	0.0840	0.0773	0.1058	0.0862	0.0788	0.0864	0.0859	0.0770	0.0802	0.0873	0.3730	0.0759	0.1251	0.1053	0.0811
0.0938	0.0817	0.0799	0.0154	0.0186	0.0164	0.0185	0.0354	0.0955	0.0123	0.0497	0.0096	0.0159	0.1191	0.0168	0.0149	0.0038	0.0151	0.0099	0.0128	0.0196	0.0182	0.0172	0.0174	0.0104	0.0119	0.0171	0.0739	0.0595	0.1561	0.0178	0.0135	0.0128	0.0102	0.0130	0.0166	0.0810	0.0337	0.0184	0.0114	0.0204	0.0170	0.0520	0.0114	0.0189	0.3879	0.0149	0.0462	0.0575	0.0115
0.0926	0.0814	0.0799	0.0150	0.0184	0.0164	0.0189	0.0356	0.0954	0.0125	0.0494	0.0096	0.0158	0.1185	0.0166	0.0156	0.0053	0.0159	0.0098	0.0136	0.0192	0.0182	0.0170	0.0171	0.0094	0.0118	0.0169	0.0740	0.0585	0.1559	0.0185	0.0143	0.0137	0.0104	0.0128	0.0174	0.0807	0.0333	0.0183	0.0111	0.0203	0.0172	0.0515	0.0110	0.0182	0.3863	0.0147	0.0459	0.0568	0.0115
0.0951	0.0799	0.0822	0.0166	0.0202	0.0180	0.0139	0.0367	0.0943	0.0154	0.0481	0.0118	0.0169	0.1159	0.0129	0.0186	0.0131	0.0189	0.0113	0.0144	0.0214	0.0204	0.0190	0.0191	0.0126	0.0127	0.0181	0.0758	0.0572	0.1542	0.0206	0.0143	0.0142	0.0118	0.0144	0.0186	0.0788	0.0356	0.0187	0.0128	0.0209	0.0181	0.0491	0.0138	0.0206	0.3875	0.0106	0.0472	0.0572	0.0123
0.0925	0.0703	0.0511	0.0397	0.0408	0.0402	0.0436	0.0604	0.0857	0.0410	0.0781	0.0383	0.0455	0.1141	0.0439	0.0384	0.0396	0.0377	0.0393	0.0397	0.0433	0.0422	0.0455	0.0458	0.0402	0.0405	0.0419	0.0349	0.0834	0.1429	0.0398	0.0395	0.0397	0.0373	0.0393	0.0376	0.0868	0.0609	0.0407	0.0398	0.0406	0.0422	0.0747	0.0404	0.0423	0.3565	0.0419	0.0752	0.0812	0.0406
0.0899	0.0709	0.0698	0.0155	0.0082	0.0093	0.0154	0.0347	0.0923	0.0207	0.0482	0.0178	0.0229	0.1207	0.0177	0.0136	0.0183	0.0139	0.0175	0.0176	0.0044	0.0030	0.0258	0.0258	0.0189	0.0157	0.0104	0.0702	0.0535	0.1548	0.0126	0.0178	0.0164	0.0185	0.0170	0.0147	0.0844	0.0305	0.0115	0.0160	0.0099	0.0103	0.0448	0.0123	0.0044	0.3870	0.0229	0.0446	0.0546	0.0151
0.0934	0.0815	0.0814	0.0149	0.0184	0.0181	0.0194	0.0352	0.0958	0.0135	0.0513	0.0108	0.0159	0.1171	0.0195	0.0123	0.0112	0.0116	0.0113	0.0100	0.0212	0.0196	0.0175	0.0178	0.0123	0.0109	0.0166	0.0728	0.0591	0.1537	0.0132	0.0100	0.0094	0.0067	0.0096	0.0119	0.0808	0.0359	0.0152	0.0116	0.0180	0.0173	0.0523	0.0143	0.0202	0.3869	0.0144	0.0471	0.0575	0.0118
0.0877	0.0758	0.0750	0.0115	0.0128	0.0125	0.0125	0.0346	0.0937	0.0179	0.0512	0.0151	0.0203	0.1233	0.0163	0.0048	0.0160	0.0043	0.0157	0.0147	0.0154	0.0139	0.0221	0.0223	0.0169	0.0165	0.0111	0.0692	0.0547	0.1537	0.0083	0.0150	0.0146	0.0119	0.0147	0.0043	0.0801	0.0333	0.0104	0.0161	0.0128	0.0120	0.0465	0.0190	0.0146	0.3893	0.0191	0.0473	0.0548	0.0169
0.0946	0.0783	0.0783	0.0154	0.0149	0.0184	0.0200	0.0317	0.0965	0.0137	0.0477	0.0107	0.0153	0.1143	0.0193	0.0162	0.0120	0.0160	0.0114	0.0087	0.0179	0.0161	0.0177	0.0179	0.0130	0.0083	0.0124	0.0733	0.0578	0.1529	0.0145	0.0080	0.0070	0.0116	0.0070	0.0152	0.0818	0.0324	0.0134	0.0076	0.0151	0.0149	0.0491	0.0102	0.0167	0.3868	0.0154	0.0430	0.0539	0.0085
0.0956	0.0800	0.0782	0.0165	0.0164	0.0177	0.0194	0.0327	0.0972	0.0148	0.0469	0.0115	0.0159	0.1148	0.0186	0.0176	0.0120	0.0172	0.0107	0.0082	0.0171	0.0154	0.0181	0.0182	0.0122	0.0044	0.0121	0.0748	0.0571	0.1541	0.0148	0.0095	0.0089	0.0109	0.0069	0.0164	0.0820	0.0319	0.0126	0.0088	0.0160	0.0142	0.0481	0.0094	0.0162	0.3885	0.0162	0.0429	0.0545	0.0076
0.0907	0.0681	0.0691	0.0148	0.0051	0.0114	0.0169	0.0337	0.0936	0.0206	0.0501	0.0173	0.0240	0.1175	0.0187	0.0140	0.0196	0.0139	0.0185	0.0172	0.0113	0.0095	0.0249	0.0251	0.0207	0.0159	0.0108	0.0693	0.0547	0.1537	0.0114	0.0165	0.0152	0.0198	0.0154	0.0134	0.0847	0.0314	0.0103	0.0147	0.0026	0.0119	0.0445	0.0151	0.0099	0.3868	0.0223	0.0434	0.0539	0.0163
0.0928	0.0829	0.0820	0.0171	0.0217	0.0180	0.0202	0.0377	0.0941	0.0133	0.0520	0.0125	0.0180	0.1188	0.0182	0.0189	0.0125	0.0187	0.0120	0.0160	0.0206	0.0210	0.0182	0.0182	0.0120	0.0146	0.0194	0.0755	0.0582	0.1552	0.0213	0.0159	0.0156	0.0125	0.0155	0.0194	0.0802	0.0360	0.0202	0.0134	0.0224	0.0166	0.0526	0.0141	0.0198	0.3844	0.0140	0.0472	0.0583	0.0137
0.1012	0.0661	0.0871	0.0213	0.0260	0.0233	0.0239	0.0463	0.0811	0.0269	0.0391	0.0239	0.0313	0.1095	0.0219	0.0248	0.0237	0.0245	0.0248	0.0279	0.0268	0.0263	0.0310	0.0312	0.0258	0.0274	0.0263	0.0806	0.0445	0.1447	0.0266	0.0277	0.0275	0.0256	0.0273	0.0252	0.0677	0.0419	0.0263	0.0255	0.0266	0.0252	0.0379	0.0252	0.0259	0.3740	0.0260	0.0581	0.0476	0.0267
0.0517	0.0995	0.0989	0.0529	0.0570	0.0557	0.0572	0.0744	0.1127	0.0512	0.0892	0.0476	0.0526	0.1392	0.0560	0.0555	0.0493	0.0558	0.0487	0.0525	0.0587	0.0587	0.0541	0.0542	0.0477	0.0514	0.0568	0.0931	0.0925	0.1160	0.0579	0.0524	0.0525	0.0505	0.0519	0.0553	0.1199	0.0738	0.0572	0.0495	0.0581	0.0557	0.0924	0.0500	0.0586	0.4063	0.0528	0.0875	0.0936	0.0511
0.1447	0.0826	0.0988	0.0616	0.0668	0.0655	0.0630	0.0809	0.0988	0.0592	0.0540	0.0581	0.0653	0.1216	0.0614	0.0644	0.0582	0.0642	0.0579	0.0612	0.0685	0.0678	0.0656	0.0655	0.0591	0.0607	0.0658	0.0933	0.0605	0.1498	0.0666	0.0617	0.0632	0.0581	0.0613	0.0650	0.0538	0.0812	0.0663	0.0592	0.0690	0.0649	0.0460	0.0598	0.0678	0.3456	0.0585	0.0970	0.0828	0.0603
0.1070	0.0958	0.0633	0.0302	0.0322	0.0298	0.0312	0.0529	0.1137	0.0364	0.0683	0.0338	0.0400	0.1457	0.0319	0.0290	0.0341	0.0287	0.0330	0.0367	0.0328	0.0317	0.0405	0.0406	0.0322	0.0359	0.0301	0.0611	0.0710	0.1750	0.0321	0.0363	0.0363	0.0333	0.0366	0.0292	0.0700	0.0492	0.0312	0.0348	0.0326	0.0302	0.0651	0.0351	0.0317	0.3762	0.0385	0.0657	0.0726	0.0354
0.1258	0.0978	0.1155	0.0461	0.0510	0.0503	0.0496	0.0370	0.1122	0.0487	0.0400	0.0463	0.0568	0.1417	0.0455	0.0489	0.0482	0.0489	0.0489	0.0519	0.0526	0.0524	0.0537	0.0538	0.0499	0.0514	0.0512	0.1073	0.0755	0.1391	0.0511	0.0508	0.0534	0.0511	0.0511	0.0505	0.0661	0.0684	0.0512	0.0483	0.0520	0.0495	0.0681	0.0501	0.0516	0.3664	0.0481	0.0655	0.0831	0.0501
0.1096	0.0933	0.0917	0.0331	0.0292	0.0304	0.0348	0.0489	0.1142	0.0383	0.0641	0.0354	0.0408	0.1428	0.0341	0.0328	0.0364	0.0328	0.0348	0.0351	0.0269	0.0266	0.0413	0.0417	0.0360	0.0333	0.0290	0.0914	0.0735	0.1710	0.0313	0.0348	0.0339	0.0362	0.0343	0.0332	0.0978	0.0486	0.0302	0.0326	0.0298	0.0278	0.0635	0.0303	0.0253	0.4081	0.0406	0.0189	0.0722	0.0330
0.1082	0.0977	0.0676	0.0259	0.0343	0.0331	0.0342	0.0530	0.1133	0.0355	0.0697	0.0328	0.0403	0.1457	0.0311	0.0306	0.0343	0.0303	0.0338	0.0367	0.0363	0.0354	0.0408	0.0409	0.0333	0.0370	0.0336	0.0596	0.0721	0.1720	0.0335	0.0351	0.0353	0.0340	0.0357	0.0313	0.0694	0.0468	0.0335	0.0337	0.0348	0.0332	0.0680	0.0368	0.0353	0.3756	0.0371	0.0667	0.0685	0.0360
0.0932	0.0772	0.0767	0.0152	0.0142	0.0167	0.0192	0.0318	0.0961	0.0121	0.0457	0.0088	0.0169	0.1148	0.0173	0.0177	0.0108	0.0180	0.0099	0.0110	0.0169	0.0154	0.0179	0.0180	0.0103	0.0093	0.0145	0.0754	0.0578	0.1518	0.0160	0.0109	0.0101	0.0119	0.0103	0.0182	0.0820	0.0275	0.0150	0.0077	0.0164	0.0140	0.0480	0.0082	0.0160	0.3880	0.0153	0.0426	0.0541	0.0083
0.1163	0.0593	0.1046	0.0348	0.0415	0.0395	0.0386	0.0607	0.0742	0.0423	0.0325	0.0400	0.0474	0.1039	0.0314	0.0393	0.0407	0.0397	0.0406	0.0449	0.0419	0.0419	0.0469	0.0469	0.0413	0.0435	0.0404	0.0974	0.0376	0.1293	0.0425	0.0450	0.0461	0.0421	0.0446	0.0410	0.0619	0.0536	0.0416	0.0421	0.0431	0.0388	0.0246	0.0418	0.0408	0.3597	0.0430	0.0715	0.0554	0.0425
0.1908	0.1742	0.1695	0.1069	0.1086	0.1079	0.1106	0.0921	0.1821	0.1076	0.1176	0.1051	0.1092	0.2042	0.1099	0.1059	0.1071	0.1056	0.1056	0.1072	0.1081	0.1073	0.1031	0.1033	0.1071	0.1065	0.1072	0.1645	0.1520	0.2155	0.1059	0.1063	0.1122	0.1073	0.1072	0.1065	0.1310	0.1261	0.1075	0.1059	0.1084	0.1057	0.1420	0.1067	0.1074	0.3096	0.1112	0.1227	0.1318	0.1057
0.1132	0.0579	0.1017	0.0381	0.0396	0.0378	0.0363	0.0606	0.0736	0.0415	0.0336	0.0394	0.0491	0.1041	0.0385	0.0377	0.0426	0.0375	0.0412	0.0455	0.0421	0.0413	0.0467	0.0467	0.0397	0.0450	0.0394	0.0962	0.0316	0.1320	0.0397	0.0447	0.0453	0.0431	0.0444	0.0370	0.0593	0.0586	0.0393	0.0418	0.0406	0.0388	0.0280	0.0454	0.0417	0.3604	0.0406	0.0749	0.0551	0.0446
0.2266	0.2069	0.2019	0.1398	0.1379	0.1388	0.1426	0.1252	0.2168	0.1406	0.1483	0.1380	0.1381	0.1935	0.1426	0.1380	0.1395	0.1383	0.1392	0.1399	0.1391	0.1385	0.1327	0.1330	0.1401	0.1394	0.1399	0.1971	0.1869	0.2515	0.1387	0.1390	0.1401	0.1390	0.1397	0.1388	0.1605	0.1594	0.1404	0.1384	0.1390	0.1389	0.1758	0.1388	0.1386	0.2942	0.1441	0.1565	0.1611	0.1384
0.1054	0.0809	0.0481	0.0795	0.0814	0.0797	0.0832	0.1000	0.0967	0.0767	0.1175	0.0747	0.0810	0.1219	0.0818	0.0801	0.0746	0.0805	0.0755	0.0783	0.0831	0.0822	0.0819	0.0818	0.0744	0.0776	0.0825	0.0452	0.1240	0.1560	0.0834	0.0779	0.0797	0.0752	0.0780	0.0817	0.1176	0.0997	0.0829	0.0759	0.0836	0.0816	0.1176	0.0747	0.0825	0.3554	0.0787	0.1153	0.1155	0.0765

HL10	BL3	HL11	HL12	HL13	HL14	HL15	HL16	HL17	HL18	FC7	HL19	HL20	HL21	HL22	HL23	HL24	HL25	BL4	DS12.1	DS13	FC8	DS14	DS15	DS16	DS17	FC15	DS19	HL26	CL2	CL3	CL4	FC9	HL27	BL5	CL1	CL6	BL6	BL7	BL8	HL28	HL29	BL9

0.0637	
0.1520	0.1048	
0.0897	0.0427	0.1229	
0.1071	0.0655	0.1426	0.0409	
0.0894	0.0440	0.1237	0.0075	0.0414	
0.0918	0.0446	0.1239	0.0067	0.0413	0.0111	
0.0801	0.0363	0.1197	0.0146	0.0420	0.0157	0.0189	
0.0691	0.0251	0.1040	0.0350	0.0635	0.0352	0.0390	0.0367	
0.0798	0.0276	0.1141	0.0478	0.0746	0.0485	0.0500	0.0505	0.0280	
0.0891	0.0425	0.1244	0.0152	0.0415	0.0152	0.0127	0.0152	0.0432	0.0549	
0.0905	0.0451	0.1285	0.0273	0.0483	0.0275	0.0266	0.0191	0.0464	0.0600	0.0234	
0.0624	0.1004	0.1838	0.0751	0.0947	0.0761	0.0751	0.0708	0.0971	0.1062	0.0720	0.0796	
0.0417	0.0337	0.1215	0.0610	0.0800	0.0617	0.0630	0.0533	0.0412	0.0509	0.0595	0.0624	0.0881	
0.0832	0.0368	0.1220	0.0139	0.0416	0.0148	0.0163	0.0112	0.0388	0.0508	0.0107	0.0234	0.0714	0.0544	
0.0881	0.0408	0.1234	0.0186	0.0417	0.0198	0.0162	0.0157	0.0434	0.0550	0.0116	0.0231	0.0710	0.0591	0.0109	
0.0844	0.0372	0.1220	0.0154	0.0413	0.0163	0.0175	0.0108	0.0388	0.0509	0.0119	0.0235	0.0721	0.0545	0.0071	0.0099	
0.0839	0.0373	0.1223	0.0156	0.0413	0.0165	0.0178	0.0111	0.0386	0.0511	0.0120	0.0236	0.0723	0.0543	0.0071	0.0095	0.0027	
0.0747	0.0655	0.1310	0.0798	0.1066	0.0787	0.0818	0.0816	0.0611	0.0719	0.0866	0.0919	0.1181	0.0455	0.0843	0.0865	0.0842	0.0845	
0.0877	0.0407	0.1228	0.0111	0.0411	0.0122	0.0130	0.0157	0.0356	0.0473	0.0184	0.0280	0.0760	0.0579	0.0133	0.0166	0.0135	0.0138	0.0785	
0.0865	0.0403	0.1227	0.0110	0.0417	0.0119	0.0131	0.0157	0.0348	0.0473	0.0186	0.0278	0.0761	0.0574	0.0130	0.0168	0.0137	0.0137	0.0782	0.0047	
0.0863	0.0409	0.1220	0.0140	0.0438	0.0142	0.0158	0.0178	0.0340	0.0456	0.0194	0.0235	0.0774	0.0576	0.0155	0.0199	0.0152	0.0153	0.0784	0.0132	0.0126	
0.0724	0.0690	0.1492	0.0398	0.0651	0.0397	0.0413	0.0400	0.0629	0.0739	0.0418	0.0502	0.0358	0.0710	0.0399	0.0414	0.0405	0.0404	0.0949	0.0400	0.0406	0.0422	
0.0881	0.0425	0.1242	0.0153	0.0418	0.0161	0.0124	0.0161	0.0443	0.0557	0.0046	0.0240	0.0712	0.0602	0.0106	0.0113	0.0124	0.0126	0.0875	0.0181	0.0183	0.0206	0.0422	
0.0880	0.0444	0.1201	0.0111	0.0414	0.0104	0.0141	0.0155	0.0356	0.0489	0.0186	0.0277	0.0748	0.0603	0.0153	0.0187	0.0158	0.0159	0.0777	0.0113	0.0120	0.0139	0.0346	0.0199	
0.0849	0.0417	0.1201	0.0162	0.0414	0.0150	0.0196	0.0124	0.0406	0.0544	0.0133	0.0249	0.0712	0.0566	0.0098	0.0134	0.0098	0.0098	0.0836	0.0162	0.0168	0.0181	0.0371	0.0141	0.0102	
0.0901	0.0450	0.1244	0.0077	0.0415	0.0079	0.0111	0.0162	0.0363	0.0492	0.0153	0.0276	0.0752	0.0617	0.0162	0.0196	0.0162	0.0163	0.0801	0.0125	0.0125	0.0139	0.0390	0.0165	0.0101	0.0150	
0.0894	0.0439	0.1249	0.0088	0.0409	0.0078	0.0096	0.0172	0.0369	0.0481	0.0143	0.0284	0.0768	0.0609	0.0151	0.0187	0.0157	0.0158	0.0811	0.0119	0.0114	0.0131	0.0404	0.0155	0.0109	0.0164	0.0080	
0.0902	0.0440	0.1238	0.0149	0.0422	0.0157	0.0142	0.0153	0.0432	0.0558	0.0090	0.0237	0.0702	0.0614	0.0128	0.0135	0.0133	0.0133	0.0858	0.0206	0.0204	0.0209	0.0406	0.0099	0.0181	0.0129	0.0152	0.0162	
0.0848	0.0421	0.1246	0.0141	0.0448	0.0139	0.0165	0.0166	0.0374	0.0474	0.0203	0.0300	0.0776	0.0601	0.0145	0.0208	0.0159	0.0164	0.0755	0.0123	0.0114	0.0145	0.0424	0.0211	0.0143	0.0192	0.0151	0.0142	0.0227	
0.0718	0.0268	0.1090	0.0261	0.0495	0.0267	0.0272	0.0232	0.0291	0.0397	0.0259	0.0301	0.0826	0.0425	0.0229	0.0251	0.0233	0.0233	0.0675	0.0243	0.0241	0.0247	0.0486	0.0262	0.0262	0.0247	0.0274	0.0268	0.0267	0.0275	
0.1034	0.0815	0.1067	0.0504	0.0782	0.0509	0.0533	0.0537	0.0727	0.0875	0.0586	0.0657	0.0949	0.0975	0.0528	0.0580	0.0537	0.0541	0.1237	0.0495	0.0491	0.0515	0.0627	0.0585	0.0513	0.0558	0.0520	0.0516	0.0582	0.0505	0.0640	
0.0526	0.0392	0.1252	0.0608	0.0840	0.0606	0.0629	0.0628	0.0416	0.0408	0.0672	0.0730	0.0954	0.0229	0.0620	0.0670	0.0623	0.0623	0.0386	0.0577	0.0573	0.0576	0.0745	0.0673	0.0600	0.0643	0.0618	0.0607	0.0689	0.0569	0.0487	0.0989	
0.0682	0.0573	0.1432	0.0360	0.0590	0.0362	0.0377	0.0309	0.0567	0.0717	0.0312	0.0438	0.0630	0.0374	0.0271	0.0319	0.0259	0.0261	0.0678	0.0335	0.0328	0.0358	0.0458	0.0318	0.0350	0.0286	0.0363	0.0357	0.0328	0.0345	0.0420	0.0733	0.0462	
0.1077	0.0569	0.1095	0.0480	0.0707	0.0499	0.0523	0.0474	0.0565	0.0691	0.0529	0.0563	0.1090	0.0781	0.0477	0.0508	0.0481	0.0479	0.1052	0.0488	0.0483	0.0472	0.0760	0.0527	0.0495	0.0497	0.0499	0.0515	0.0520	0.0503	0.0518	0.0881	0.0807	0.0691	
0.1084	0.0597	0.1423	0.0328	0.0609	0.0340	0.0330	0.0340	0.0617	0.0730	0.0274	0.0447	0.0933	0.0782	0.0299	0.0327	0.0310	0.0309	0.1092	0.0365	0.0359	0.0377	0.0610	0.0273	0.0376	0.0331	0.0336	0.0332	0.0295	0.0371	0.0441	0.0756	0.0852	0.0505	0.0662	
0.0646	0.0550	0.1415	0.0348	0.0590	0.0351	0.0386	0.0274	0.0559	0.0716	0.0347	0.0405	0.0615	0.0349	0.0295	0.0357	0.0304	0.0304	0.0656	0.0348	0.0338	0.0365	0.0448	0.0353	0.0341	0.0307	0.0354	0.0363	0.0351	0.0327	0.0413	0.0731	0.0459	0.0110	0.0679	0.0535	
0.0887	0.0416	0.1241	0.0081	0.0421	0.0093	0.0100	0.0157	0.0358	0.0488	0.0155	0.0285	0.0775	0.0594	0.0140	0.0188	0.0150	0.0151	0.0812	0.0103	0.0102	0.0131	0.0420	0.0154	0.0138	0.0182	0.0102	0.0093	0.0165	0.0137	0.0265	0.0493	0.0590	0.0348	0.0497	0.0335	0.0358	
0.0633	0.0036	0.1043	0.0423	0.0646	0.0439	0.0439	0.0358	0.0250	0.0280	0.0421	0.0446	0.0990	0.0338	0.0363	0.0403	0.0363	0.0363	0.0653	0.0403	0.0398	0.0403	0.0677	0.0417	0.0437	0.0408	0.0440	0.0431	0.0433	0.0414	0.0263	0.0812	0.0391	0.0563	0.0568	0.0587	0.0539	0.0411	
0.1824	0.1359	0.1815	0.1049	0.1224	0.1063	0.1076	0.1060	0.1336	0.1429	0.1083	0.1108	0.1664	0.1511	0.1069	0.1094	0.1071	0.1072	0.1802	0.1069	0.1073	0.1098	0.1335	0.1079	0.1071	0.1066	0.1067	0.1075	0.1085	0.1091	0.1183	0.1468	0.1556	0.1257	0.1092	0.1223	0.1257	0.1067	0.1342	
0.0612	0.0211	0.0986	0.0431	0.0661	0.0434	0.0474	0.0364	0.0221	0.0330	0.0412	0.0467	0.0981	0.0359	0.0367	0.0414	0.0363	0.0364	0.0607	0.0435	0.0421	0.0416	0.0671	0.0417	0.0431	0.0376	0.0443	0.0451	0.0406	0.0408	0.0282	0.0810	0.0424	0.0530	0.0589	0.0601	0.0532	0.0443	0.0210	0.1347	
0.2176	0.1697	0.1971	0.1382	0.1545	0.1388	0.1388	0.1391	0.1672	0.1768	0.1386	0.1390	0.1992	0.1867	0.1390	0.1400	0.1399	0.1407	0.1864	0.1403	0.1409	0.1426	0.1662	0.1394	0.1394	0.1377	0.1390	0.1398	0.1390	0.1424	0.1512	0.1823	0.1915	0.1593	0.1414	0.1550	0.1594	0.1400	0.1691	0.0318	0.1688	
0.0617	0.1082	0.1939	0.0770	0.1025	0.0771	0.0781	0.0798	0.0985	0.1123	0.0824	0.0917	0.0451	0.0860	0.0775	0.0818	0.0795	0.0792	0.1128	0.0745	0.0737	0.0772	0.0576	0.0825	0.0768	0.0812	0.0780	0.0774	0.0833	0.0757	0.0886	0.0944	0.0871	0.0629	0.1166	0.1017	0.0637	0.0760	0.1076	0.1742	0.1060	0.2083	
