# Supplementary material for: ddRAD sequencing-based genotyping for population structure analysis in cultivated tomato provides new insights into the genomic diversity of Mediterranean ‘da serbo’ type long shelf-life germplasm
Source: Hortic Res. 2020 Sep 1;7:134. doi: 10.1038/s41438-020-00353-6 (PMC7459340; doi:10.1038/s41438-020-00353-6)
Supplement: Supplementary file 6 — Supplementary Table 6 [file 41438_2020_353_MOESM6_ESM.pdf]

Supplementary Table 6: Tomato sub set representing the entire genetic diversity of 288 cultivated genotypes

Sample ID	Biological status/Tipology	Provenance	Admixture Cluster
BL1	Breeding line	Peru	K2
BL2	Breeding line	na	Admixted
BL10	Breeding line	na	K3
BL11	Breeding line	na	Admixted
BL12	Breeding line	na	K3
BL13	Breeding line	na	K2
BL4	Breeding line	na	K5
BL5	Breeding line	na	Admixted
BL9	Breeding line	na	K2
CL1	Cultivars	na	Admixted
CL2	Cultivars	na	K6
CL4	Cultivars	na	K6
CL8	Cultivars	Italy	Admixted
CL9	Cultivars	USA	K5
CL14	Cultivars	Ecuador	Admixted
CL16	Cultivars	na	K6
CL15	Cultivars	Mexico	K4
CL17	Cultivars	Philippines	K4
CL18	Cultivars	Philippines	K1
CL19	Cultivars	China	K5
CL22	Cultivars	Spain	K3
CL36	Cultivars	Perù	K5
CL37	Cultivars	Perù	K1
CL39	Cultivars	Perù	K5
CL40	Cultivars	na	Admixted
CL42	Cultivars	Argentina	K6
CL43	Cultivars	Ecuador	K6
CL44	Cultivars	Peru	K1
CL55	Cultivars	France	K4
CL70	Cultivars	USA	K5
CL72	Cultivars	Argentina	K5
CL75	Cultivars	Canada	K5
DS5	Da serbo landraces	Italy	K5
DS6	Da serbo landraces	Italy	K5
DS14	Da serbo landraces	Italy	Admixted
DS29	Da serbo landraces	Spain	K1
DS30	Da serbo landraces	Spain	Admixted
DS32	Da serbo landraces	Spain	K6
DS34	Da serbo landraces	Spain	K4
DS35	Da serbo landraces	Spain	K6
DS39	Da serbo landraces	Spain	K4
FC25	Fresh consumption landraces	Italy	K3
FC34	Fresh consumption landraces	Italy	K6
FC42	Fresh consumption landraces	Spain	K2
FC44	Fresh consumption landraces	Spain	K2
FC53	Fresh consumption landraces	Spain	Admixted
HL1	Heirloom	na	K5
HL2	Heirloom	na	K3
HL10	Heirloom	na	K2
HL11	Heirloom	France	K5
HL13	Heirloom	USA	Admixted
HL18	Heirloom	USA	K5
HL19	Heirloom	Russia	Admixted

HL29	Heirloom	USA	K4
HL30	Heirloom	Israel	Admixted
HL31	Heirloom	USA	K6
HL32	Heirloom	USA	K2
HL47	Heirloom	Poland	K2

na = not available
