# Supplementary material for: ddRAD sequencing-based genotyping for population structure analysis in cultivated tomato provides new insights into the genomic diversity of Mediterranean ‘da serbo’ type long shelf-life germplasm
Source: Hortic Res. 2020 Sep 1;7:134. doi: 10.1038/s41438-020-00353-6 (PMC7459340; doi:10.1038/s41438-020-00353-6)
Supplement: Supplementary file 7 — Supplementary Table 7 [file 41438_2020_353_MOESM7_ESM.pdf]

Supplementary Table 7: List of 53 highly frequent SNPs in LSL 'da serbo' tomato genotypes, with related MAF, localization gene description and biological role

Chr	SNP positions	MAF in "da serbo"	MAF in other types	SNP localization	Gene ID	Gene Description	Biological role*
ch01	4325265	0.413	0.1657	upstream_gene_variant	Solyc01g009840.3	Transcription factor GRAS	RNA biosynthesis (15)
ch01	4451418	0.4274	0.1398	intergenic_region	Solyc01g009890.1-Solyc01g009900.3	-	
ch01	4451469	0.4083	0.09938	intergenic_region	Solyc01g009890.1-Solyc01g009900.3	-	
ch01	86095937	0.4236	0.1564	upstream_gene_variant	Solyc01g105720.2	hypothetical protein	not assigned (35)
ch02	4841338	0.4151	0.1678	downstream_gene_variant	Solyc02g050320.2	Unknown protein	not assigned (35)
ch02	14386391	0.4133	0.1906	intergenic_region	Solyc02g014220.3-Solyc02g014230.1	-	
ch02	17613037	0.4133	0.1943	intergenic_region	Solyc02g150107.1-Solyc02g014860.3	-	
ch02	20344797	0.4133	0.1934	intergenic_region	Solyc02g021190.1-Solyc02g021210.1	-	
ch03	54108138	0.4914	0.1754	upstream_gene_variant	Solyc03g097220.1	Unknown protein	not assigned (35)
ch04	62994007	0.4722	0.05866	downstream_gene_variant	Solyc04g080960.4	pre-pro-cysteine proteinase	protein homeostasis (19)
ch05	53469416	0.4083	0.09333	upstream_gene_variant	Solyc05g041910.3	Cytochrome b561 and DOMON domain-containing protein	not assigned (35)
ch05	59528689	0.4478	0.1766	upstream_gene_variant	Solyc05g050160.1	Unknown protein	not assigned (35)
ch05	59690899	0.4776	0.1741	intergenic_region	Solyc05g050250.1-Solyc05g050280.3	-	
ch05	59736919	0.4194	0.1929	downstream_gene_variant	Solyc05g050280.3	Auxin-responsive GH3 family protein (*JAR1*)	phytohormone metabolism (11)
ch05	59756703	0.4811	0.1538	downstream_gene_variant	Solyc05g050290.3	Auxin-responsive GH3 family protein (*JAR1*)	phytohormone metabolism (11)
ch07	58437680	0.4766	0.1323	upstream_gene_variant	Solyc07g045450.1	hypothetical protein	not assigned (35)
ch07	58530652	0.5	0.1183	upstream_gene_variant	Solyc07g045525.1	Retrovirus-related Pol polyprotein from transposon TNT 1-94	not assigned (35)
ch07	58530665	0.5	0.1071	upstream_gene_variant	Solyc07g045525.1	Retrovirus-related Pol polyprotein from transposon TNT 1-94	not assigned (35)
ch09	6508500	0.4776	0.1842	intergenic_region	Solyc09g014643.1-Solyc09g014644.1	-	
ch09	7564619	0.4851	0.1617	intergenic_region	Solyc09g015033.1-Solyc09g015037.1	-	
ch09	16093836	0.44	0.1639	upstream_gene_variant	Solyc09g018630.3	Bis(5'-adenosyl)-triphosphatase	not assigned (35)
ch09	23757250	0.4643	0.1667	intergenic_region	Solyc09g042630.1-Solyc09g042650.2	-	
ch09	26237541	0.4521	0.174	intergenic_region	Solyc09g031780.3-Solyc09g031770.3	-	
ch09	31256826	0.4412	0.1609	intergenic_region	Solyc09g047930.1-Solyc09g047935.1	-	
ch09	33203810	0.4521	0.1732	intergenic_region	Solyc09g055180.4-Solyc09g055185.1	-	
ch09	35053891	0.4621	0.1871	intergenic_region	Solyc09g055265.1-Solyc09g055310.3	-	
ch09	35301794	0.4783	0.1685	intergenic_region	Solyc09g055310.3-Solyc09g055320.1	-	
ch09	37679354	0.4521	0.1685	intergenic_region	Solyc09g055540.2-Solyc09g055545.1	-	
ch09	37747609	0.4224	0.1506	intergenic_region	Solyc09g055540.2-Solyc09g055545.1	-	
ch09	37747620	0.4224	0.1506	intergenic_region	Solyc09g055540.2-Solyc09g055545.1	-	
ch09	49238734	0.4559	0.1598	intergenic_region	Solyc09g059070.3-Solyc09g059080.1	-	
ch09	52182206	0.4701	0.132	downstream_gene_variant	Solyc09g059820.2	E3 ubiquitin-protein ligase-like protein	not assigned (35)
ch09	60185310	0.4386	0.08276	intron_variant	Solyc09g065810.3	Guanylate-binding protein	not assigned (35)
ch09	60290341	0.5	0.1768	downstream_gene_variant	Solyc09g065890.4	Phospholipase A1-Igamma2	lipid metabolism (5)
ch09	60290575	0.5	0.1602	downstream_gene_variant	Solyc09g065890.4	Phospholipase A1-Igamma2	lipid metabolism (5)
ch09	63392122	0.4044	0.07485	3' UTR_variant	Solyc09g075480.3	Kinesin-4	cytoskeleton organization (20)
ch09	65557283	0.4697	0.1953	synonimus_variant	Solyc09g089740.3	2-oxoglutarate (2OG) and Fe(II)-dependent oxygenase superfamily protein	not assigned (35)
ch09	68036060	0.4444	0.181	upstream_gene_variant	Solyc09g098070.3	MACPF domain-containing protein	not assigned (35)
ch09	68368794	0.4795	0.1854	downstream_gene_variant	Solyc09g098490.4	clathrin interactor EPSIN 2-like	not assigned (35)
ch09	68390847	0.4615	0.1281	intron_variant	Solyc09g098520.4	CMP-sialic acid transporter 2	solute transport (24)
ch09	68394358	0.4577	0.1831	intron_variant	Solyc09g098520.4	CMP-sialic acid transporter 2	solute transport (24)
ch09	68396924	0.4133	0.1389	intron_variant	Solyc09g098520.4	CMP-sialic acid transporter 2	solute transport (24)
ch10	47373891	0.4655	0.1964	intergenic_region	Solyc10g050250.1-Solyc10g050310.1	-	
ch10	58818571	0.4167	0.1944	downstream_gene_variant	Solyc10g076770.1	Unknown protein	not assigned (35)
ch10	62114730	0.4375	0.1509	intron_variant	Solyc10g083063.1	Unknown protein	not assigned (35)
ch10	62539333	0.4054	0.1602	upstream_gene_variant	Solyc10g083600.2	Ribosome production factor 2-like protein (ARPF2-ARRS1 complex)	protein biosyntesis (17)
ch10	62554377	0.4085	0.1629	intron_variant	Solyc10g083610.2	ethylene-inducible CTR1-like protein kinase	etylene perception (11.18)
ch10	62554444	0.4085	0.1313	intron_variant	Solyc10g083610.2	ethylene-inducible CTR1-like protein kinase	etylene perception (11.18)
ch10	62575654	0.4365	0.1258	intron_variant	Solyc10g083630.2	DNA demethylase 2	Chromatin organisation.DNA methylation.ROS1-mediated DNA demethylation (12, 14)
ch11	2232674	0.4733	0.1333	upstream_gene_variant	Solyc11g007990.2	chloroplast malate dehydrogenase (mdh)	Photosynthesis (1)
ch11	6625878	0.4918	0.1209	downstream_gene_variant	Solyc11g013490.2	Hexosyltransferase non-classical Arabinogalactan-protein	cell wall organization (21)
ch11	7839534	0.4122	0.1742	intergenic_region	Solyc11g017000.2-Solyc11g017010.2	-	
ch11	49032081	0.4653	0.1882	intergenic_region	Solyc11g065530.1-Solyc11g065540.1	-	

* in brackets, the related bin assigned by MapMan
