# Supplementary material for: ddRAD sequencing-based genotyping for population structure analysis in cultivated tomato provides new insights into the genomic diversity of Mediterranean ‘da serbo’ type long shelf-life germplasm
Source: Hortic Res. 2020 Sep 1;7:134. doi: 10.1038/s41438-020-00353-6 (PMC7459340; doi:10.1038/s41438-020-00353-6)
Supplement: Supplementary file 8 — Supplementary Table 8 [file 41438_2020_353_MOESM8_ESM.pdf]

**Supplementary Table 8:** Bin number, involved pathway, and the number of genes identified at high MAF values in long shelf life ('da serbo') genotypes.

| Bin | Pathway | Number of genes |
|---|---|---|
| 1 | Photosynthesis | 1 |
| 2 | Cellular respiration | - |
| 3 | Carbohydrate metabolism | - |
| 4 | Amino acid metabolism | - |
| 5 | Lipid metabolism | 1 |
| 6 | Nucleotide metabolism | - |
| 7 | Coenzyme metabolism | - |
| 8 | Polyamine metabolism | - |
| 9 | Secondary metabolism | - |
| 10 | Redox homeostasis | - |
| 11 | Phytohormone action | 2 |
| 12 | Chromatin organisation | 1 |
| 13 | Cell cycle organisation | - |
| 14 | DNA damage response | 1 |
| 15 | RNA biosynthesis | 1 |
| 16 | RNA processing | - |
| 17 | Protein biosynthesis | 1 |
| 18 | Protein modification | 1 |
| 19 | Protein homeostasis | 1 |
| 20 | Cytoskeleton organisation | 1 |
| 21 | Cell wall organisation | 1 |
| 22 | Vesicle trafficking | - |
| 23 | Protein translocation | - |
| 24 | Solute transport | 1 |
| 25 | Nutrient uptake | - |
| 26 | External stimuli response | - |
| 27 | Multi process regulation | - |
| 35 | not assigned | 15 |
| 50 | Enzyme classification | - |
