# Supplementary material for: ddRAD sequencing-based genotyping for population structure analysis in cultivated tomato provides new insights into the genomic diversity of Mediterranean ‘da serbo’ type long shelf-life germplasm
Source: Hortic Res. 2020 Sep 1;7:134. doi: 10.1038/s41438-020-00353-6 (PMC7459340; doi:10.1038/s41438-020-00353-6)
Supplement: Supplementary file 9 — Supplementary Figures [file 41438_2020_353_MOESM9_ESM.pdf]

Supplementary Figures

ddRAD sequencing-based genotyping for population structure analysis in cultivated tomato provides new insights into the genomic diversity of Mediterranean 'da serbo' type long shelf-life germplasm

Authors:
Salvatore Esposito, Teodoro Cardi, Gabriele Campanelli, Sara Sestili, María José Diez, Salvador Soler, Jaime Prohens, Pasquale Tripodi

Corresponding author. Pasquale Tripodi, pasquale.tripodi@crea.gov.it

This PDF file includes 6 supplementary Figures:

Supplementary Figure 1: Bar charts illustrating the distribution of master tag coverage at each position, along the 12 tomato chromosomes.

Supplementary Figure 2: Bar chart illustrating the total number of transitions and transversion events found among 288 tomato genotypes using the genome reference SL4.0. BL = Breeding lines; CL = cultivars; DS = 'da serbo'; FC = landraces for fresh consumption; HL = Heirlooms. The numbers on the ordinate axis represent the thousands of SNPs.

Supplementary Figure 3: Number of SNPs detected (A) and the observed heterozygosity in percentage (B) for the collection studied. Color legend indicates the biological status of each accession.

Supplementary Figure 4: (A) Cross Validation error for different admixture models (K=3 to 12), and (B) best K analysis using the non-parametric test Awclust. The optimal values are indicated by a red arrow.

Supplementary Figure 5: Detail of hierarchical clustering of 288 *S. lycopersicum* accessions and derived subgroups obtained at minimum variance cluster <0.1, using the AWclust software**.**

Supplementary Figure 6: MDS plot showing the distribution of 58 genotypes composing the mini-core set (red dots).

Figure S1

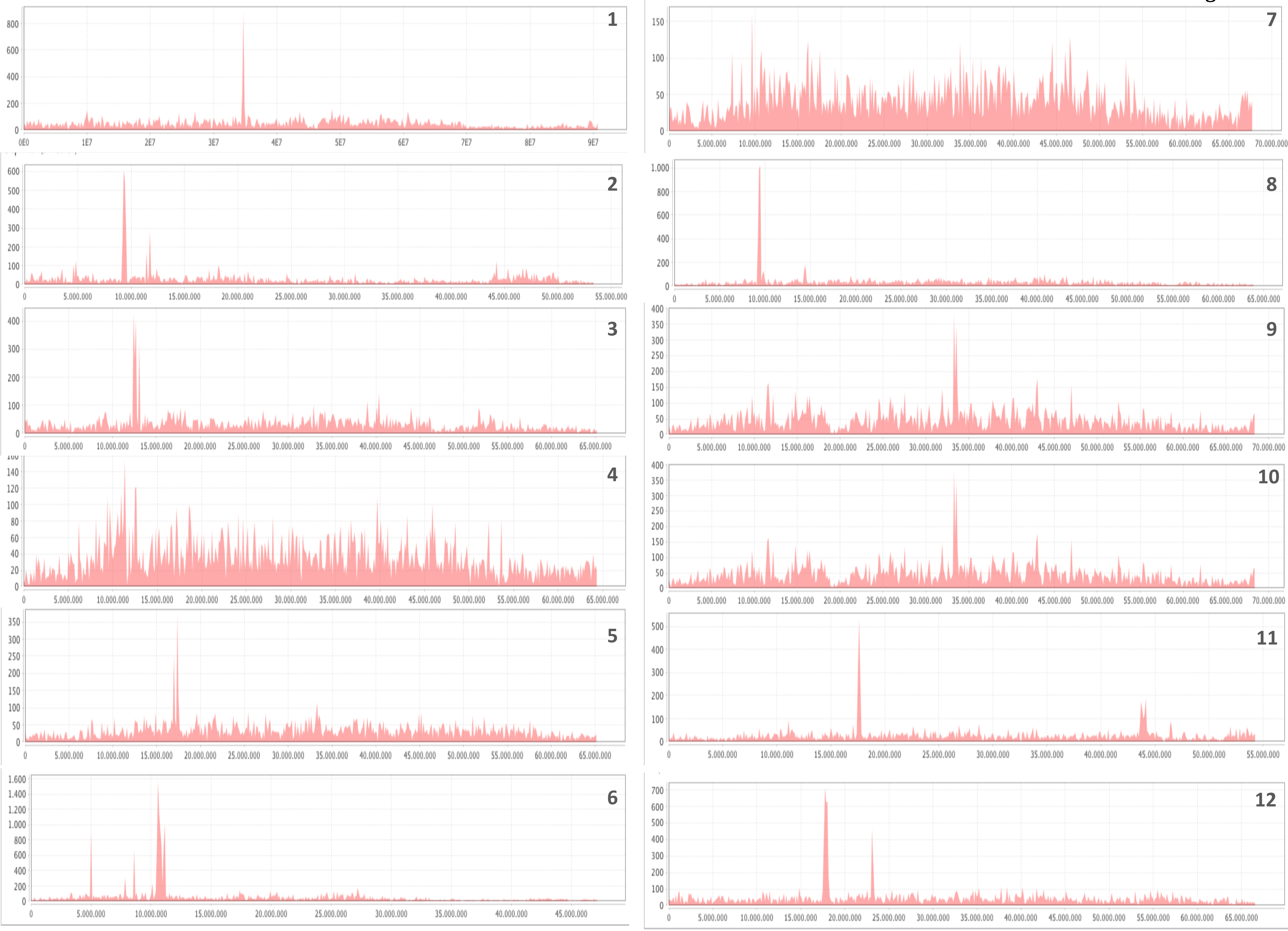

1
0 200 400 600 800
0E0 1E7 2E7 3E7 4E7 5E7 6E7 7E7 8E7 9E7
2
0 100 200 300 400 500 600
0 5.000.000 10.000.000 15.000.000 20.000.000 25.000.000 30.000.000 35.000.000 40.000.000 45.000.000 50.000.000 55.000.000
3
0 100 200 300 400
0 5.000.000 10.000.000 15.000.000 20.000.000 25.000.000 30.000.000 35.000.000 40.000.000 45.000.000 50.000.000 55.000.000 60.000.000 65.000.000
4
0 20 40 60 80 100 120 140
0 5.000.000 10.000.000 15.000.000 20.000.000 25.000.000 30.000.000 35.000.000 40.000.000 45.000.000 50.000.000 55.000.000 60.000.000 65.000.000
5
0 50 100 150 200 250 300 350
0 5.000.000 10.000.000 15.000.000 20.000.000 25.000.000 30.000.000 35.000.000 40.000.000 45.000.000 50.000.000 55.000.000 60.000.000 65.000.000
6
0 200 400 600 800 1.000 1.200 1.400 1.600
0 5.000.000 10.000.000 15.000.000 20.000.000 25.000.000 30.000.000 35.000.000 40.000.000 45.000.000
7
0 50 100 150
0 5.000.000 10.000.000 15.000.000 20.000.000 25.000.000 30.000.000 35.000.000 40.000.000 45.000.000 50.000.000 55.000.000 60.000.000 65.000.000 70.000.000
8
0 200 400 600 800 1.000
0 5.000.000 10.000.000 15.000.000 20.000.000 25.000.000 30.000.000 35.000.000 40.000.000 45.000.000 50.000.000 55.000.000 60.000.000 65.000.000
9
0 50 100 150 200 250 300 350 400
0 5.000.000 10.000.000 15.000.000 20.000.000 25.000.000 30.000.000 35.000.000 40.000.000 45.000.000 50.000.000 55.000.000 60.000.000 65.000.000 70.000.000
10
0 50 100 150 200 250 300 350 400
0 5.000.000 10.000.000 15.000.000 20.000.000 25.000.000 30.000.000 35.000.000 40.000.000 45.000.000 50.000.000 55.000.000 60.000.000 65.000.000 70.000.000
11
0 100 200 300 400 500
0 5.000.000 10.000.000 15.000.000 20.000.000 25.000.000 30.000.000 35.000.000 40.000.000 45.000.000 50.000.000 55.000.000
12
0 100 200 300 400 500 600 700
0 5.000.000 10.000.000 15.000.000 20.000.000 25.000.000 30.000.000 35.000.000 40.000.000 45.000.000 50.000.000 55.000.000 60.000.000 65.000.000

Figure S2

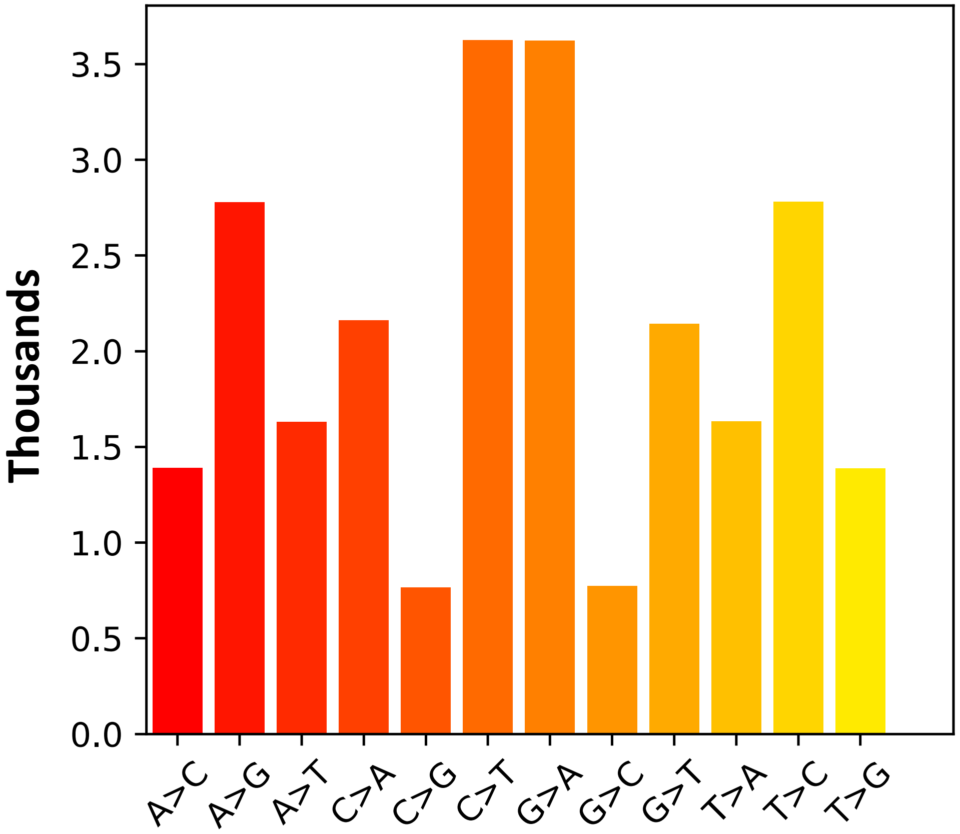

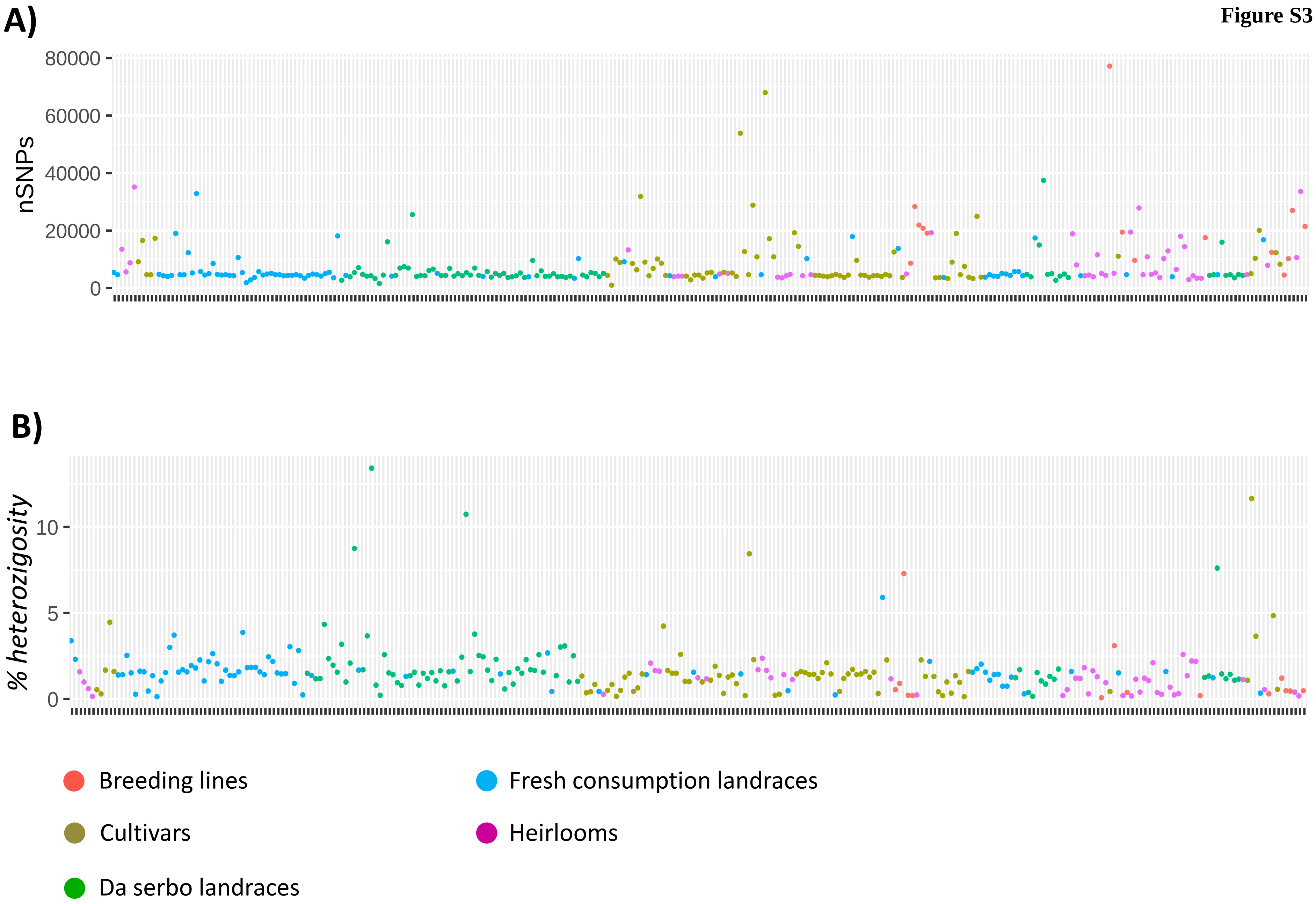
A)
nSNPs
80000
60000
40000
20000
0
B)
% heterozigosity
10
5
0
Breeding lines
Cultivars
Da serbo landraces
Fresh consumption landraces
Heirlooms

Figure S4

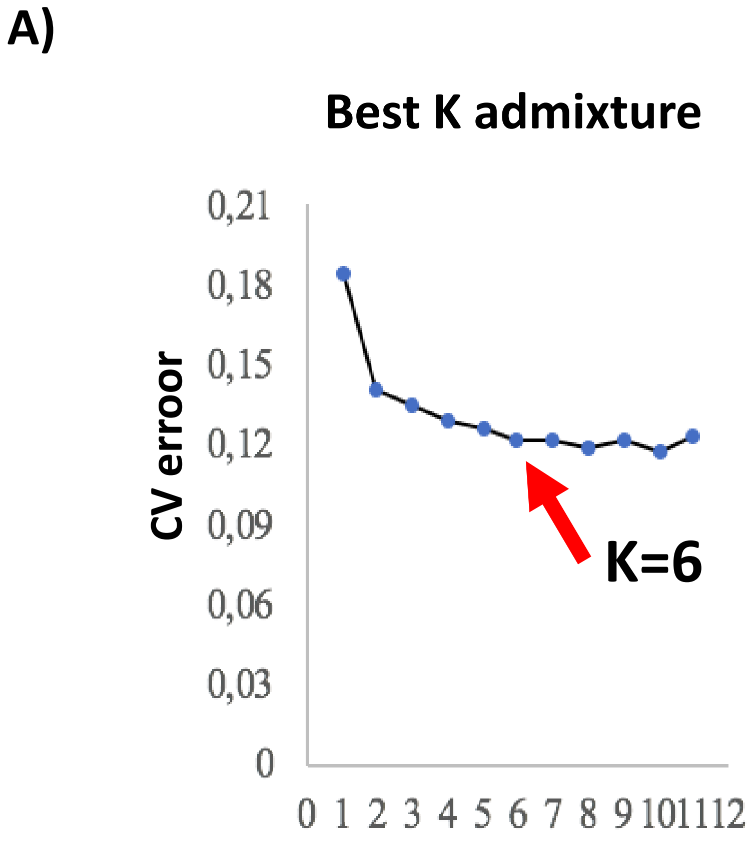
A)
Best K admixture
CV erroor
0,21
0,18
0,15
0,12
0,09
0,06
0,03
0
0 1 2 3 4 5 6 7 8 9 10 11 12
K=6

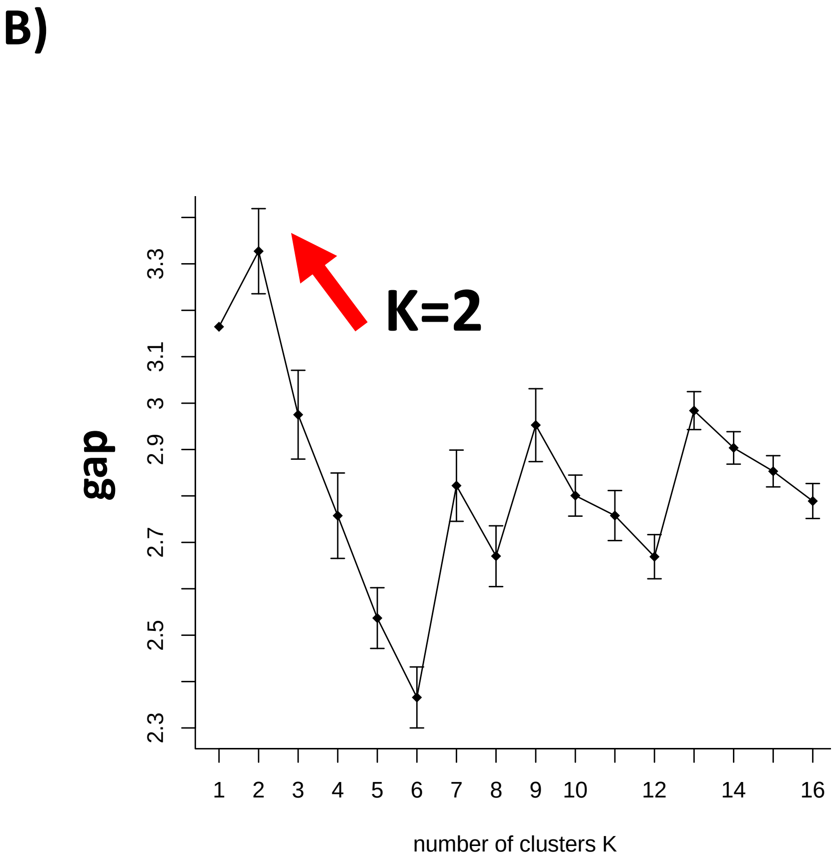
B)
gap
3.3
3.1
3
2.9
2.7
2.5
2.3
1 2 3 4 5 6 7 8 9 10 12 14 16
number of clusters K
K=2

Figure S5

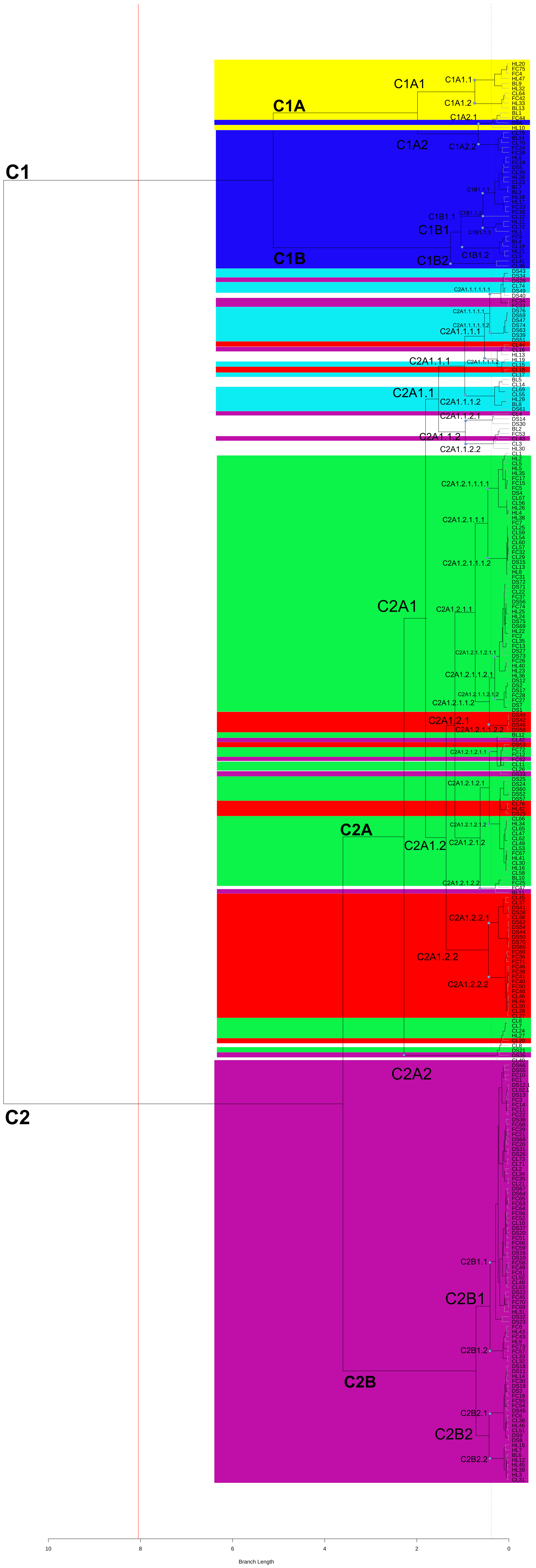
C1
C1A
C1A1
C1A1.1
C1A1.2
C1A2.1
C1A2
C1A2.2
C1B
C1B1
C1B1.1
C1B1.2
C1B2
C2A1.1.1.1.1
C2A1.1.1.1
C2A1.1.1
C2A1.1
C2A1.1.1.2
C2A1.1.2.1
C2A1.1.2
C2A1.1.2.2
C2A1.2.1.1.1.1
C2A1.2.1.1.1
C2A1.2.1.1.1.2
C2A1
C2A1.2.1.1
C2A1.2.1.1.2.1
C2A1.2.1.1.2
C2A1.2.1
C2A1.2.1.2.1
C2A1.2.1.2
C2A
C2A1.2
C2A1.2.2.1
C2A1.2.2
C2A1.2.2.2
C2A2
C2
C2B1.1
C2B1
C2B1.2
C2B
C2B2.1
C2B2
C2B2.2
10
8
6
4
2
0
Branch Length

Figure S6

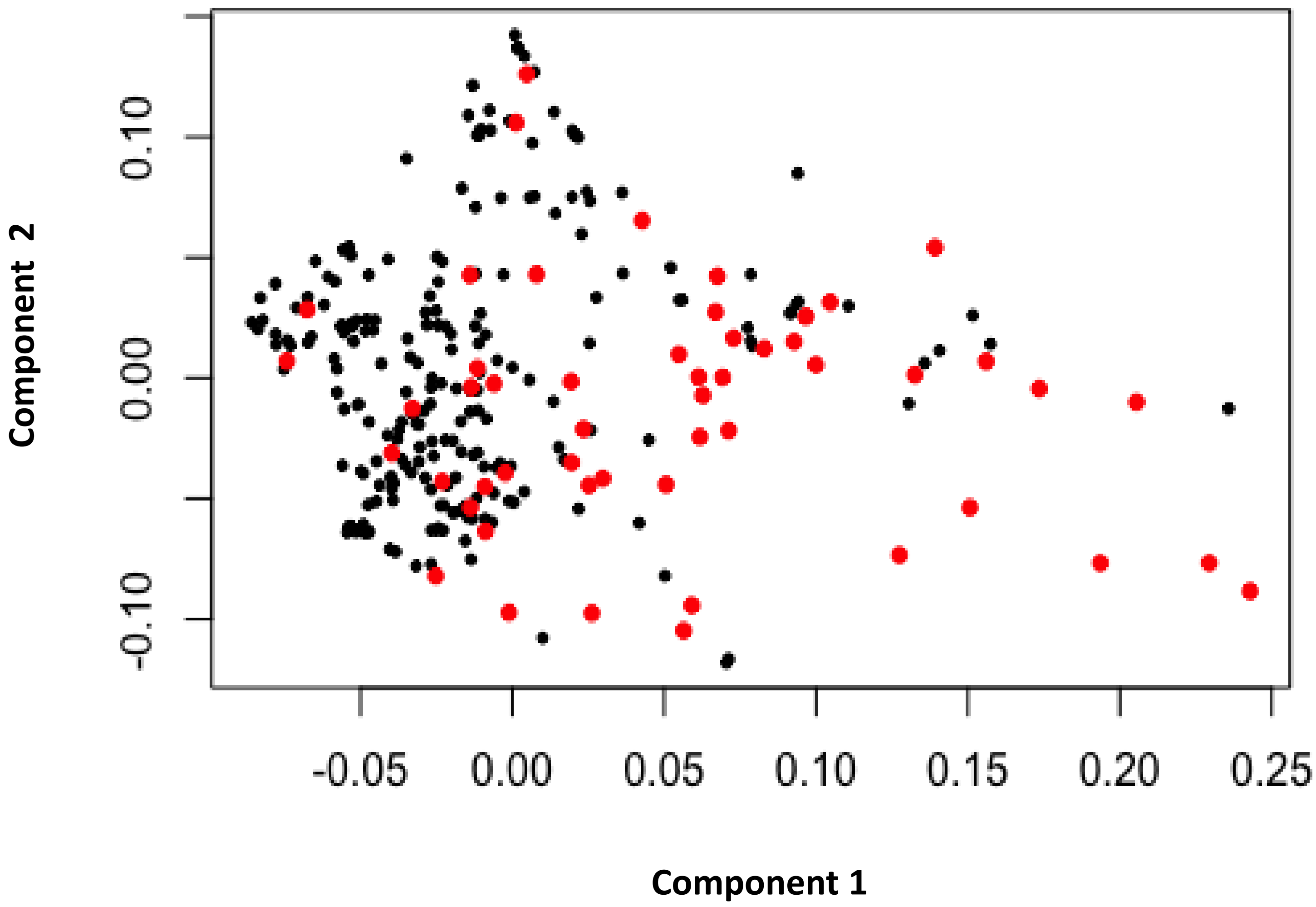

0.10
0.00
-0.10
-0.05
0.00
0.05
0.10
0.15
0.20
0.25
Component 1
Component 2
